# Sampling bias corrections for discrete and Gaussian partial information decompositions

**DOI:** 10.1016/j.patter.2026.101619

**Published:** 2026-07-23

**Authors:** Gabriel Matías Lorenz, Nicola Marie Engel, Loren Koçillari, Marco Celotto, Davide Orsenigo, Sebastiano Curreli, Simone Blanco Malerba, Andreas K. Engel, Christoph Kayser, Tommaso Fellin, Andrea I. Luppi, Stefano Panzeri

**Affiliations:** 1 Institute for Neural Information Processing, Center for Molecular Neurobiology, University Medical Center Hamburg-Eppendorf (UKE), 20251 Hamburg, Germany; 2 Department of Neurophysiology and Pathophysiology, University Medical Center Hamburg-Eppendorf (UKE), 20246 Hamburg, Germany; 3 Picower Institute for Learning and Memory, Massachusetts Institute of Technology, Cambridge, MA 02139, USA; 4 Department of Psychology, University of Torino, 10124 Turin, Italy; 5 Optical Approaches to Brain Function Laboratory, Istituto Italiano di Tecnologia, 16163 Genova, Italy; 6 Department of Cognitive Neuroscience, Bielefeld University, 33501 Bielefeld, Germany; 7 Department of Psychiatry, University of Oxford, Oxford OX3 7JX, UK; 8 St. John’s College, University of Cambridge, Cambridge CB2 1TP, UK; 9 Division of Information Engineering, University of Cambridge, Cambridge CB2 1PZ, UK

**Keywords:** information theory, neural coding, synergy, redundancy, brain communication, limited-sampling bias

## Abstract

Partial information decomposition (PID) has emerged as a principled way to decompose the information carried by neural activity into components identifying whether interactions among neurons or brain areas generate synergistic or redundant information. Here, we demonstrate that empirical measures of synergy and redundancy based on either Gaussian or discrete probability estimators suffer from a substantial limited-sampling estimation bias. This bias is much larger for synergy than for redundancy. The gap between them increases with the number of parameters specifying the probability distributions. We develop procedures that effectively correct for the bias and provide rules of thumb for the sample sizes required to obtain unbiased estimates. We show that, when used on empirical brain datasets, they successfully remove large synergy biases across species, recording modalities, and experimental designs. Our bias corrections extend the range of neuroscience questions and experimental designs addressable with PID and allow accurate comparisons between synergy and redundancy.

## Highlights

• Partial information decompositions suffer from major limited-sampling bias
• The bias is much larger for information synergy than for redundancy
• New bias-correction methods are developed and validated
• These methods work effectively on simulated and real brain data

## The bigger picture

Cognitive functions emerge because neurons interact and communicate. Recent work has used the formal principles of information theory to uncover how the interactions between neurons or brain areas contribute to information relevant for cognition.

This work has uncovered two types of interactions. Redundant interactions share the same information among different neurons, and this redundancy can make brain function more reliable, because the same information is available in more than one place. Synergistic interactions occur when a group of neurons carries a message that cannot be found in any member of the group alone. This synergy may allow the brain to represent complex aspects of the world and to use combinations of signals in flexible ways.

A central challenge is that these forms of cooperation must be inferred from experimental recordings. These datasets are limited, because animals and people can perform only so many trials, clinical datasets are especially difficult to collect, and recordings from many brain areas create many quantities that must be estimated at once.

We show how limited data affect estimates of redundancy and synergy, and we provide corrections that make these estimates more reliable. We also give guidance on how much data are needed for different types of analysis, helping researchers to plan experiments as well as analyze completed datasets. By reducing misleading effects of small sample sizes, these tools make it possible to compare redundancy and synergy more fairly across experiments, species, recording methods, and brain systems. They also open the door to studying these forms of neural cooperation in settings where data are precious, such as patient studies, disease models, and recordings from large-scale brain networks.

## Graphical abstract

**Figure:**
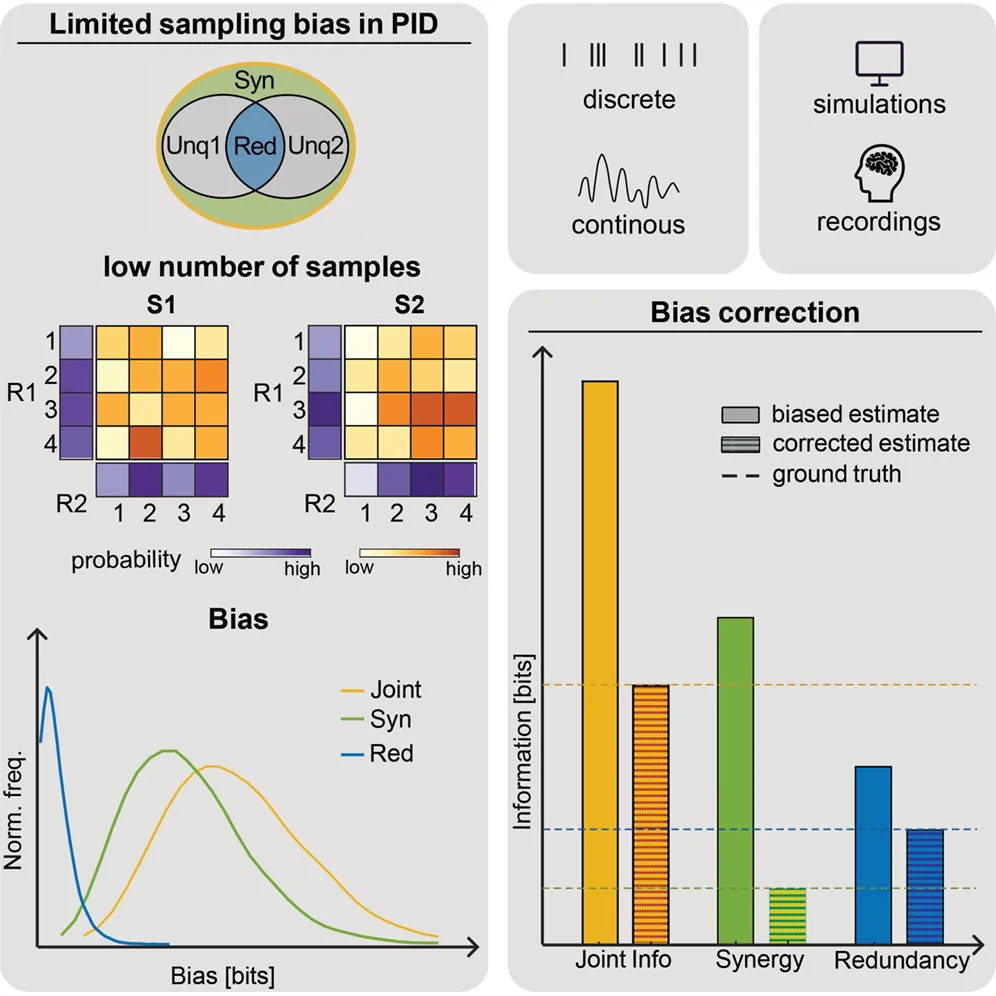

## Teaser

Lorenz, Engel et al. characterize the limited-sampling bias in partial information decomposition calculations. The bias is substantial when computed on empirical brain datasets, but the bias-correction methods developed here are demonstrated to work highly effectively to produce unbiased estimates of information synergy and redundancy.

## Introduction

The brain is a complex system whose behavior emerges from the interactions between neurons or brain areas.^1^ Because of this, the study of how interactions between neurons or brain areas shape information processing has fascinated neuroscientists for decades.^2^^,^^3^^,^^4^^,^^5^^,^^6^ Information theory has been a prominent tool in this research as it is uniquely positioned for investigating neural information processing.^7^^,^^8^^,^^9^ Shannon information can capture all ways (linear and non-linear) in which systems carry information and is thus equally applicable across species and recording modalities,^10^^,^^11^^,^^12^^,^^13^^,^^14^ real data, and neural network models.^15^^,^^16^^,^^17^^,^^18^

While earlier work has concentrated on understanding whether correlations between the activity of different neurons or brain areas increase or decrease information,^3^^,^^4^^,^^19^^,^^20^^,^^21^ recent advances in information theory based on partial information decomposition (PID)^22^ have enabled neuroscientists to formulate questions about the unique information carried by each individual neuron or brain area, the synergistic information generated by neural interactions (that is, new information that emerges specifically from and is found only in the interactions between neurons or areas), or redundant information present when different neurons or areas carry the same information.^23^ These PID tools have been developed and used both in forms suitable for discrete neural responses (usually applied for the spiking activity of neurons) and in Gaussian-distributed neural responses (usually applied to aggregate measures of neural activity).

Earlier applications of information theory to neuroscience have recognized that Shannon information measures from neural activity suffer from a prominent limited-sampling bias.^24^^,^^25^^,^^26^ This bias not only affects the accuracy of the estimate but can also skew comparisons between the information carried by simpler (less biased) and more complex (more biased) neural representations defined by a larger number of parameters. For example, the information about a stimulus carried jointly by two neurons is more biased than the information carried by each neuron individually. This would artificially inflate emergent effects if this bias is not corrected.^27^ The information-theoretic neuroscience literature has long recognized the importance of correcting for the limited-sampling bias and has provided effective tools for correcting the Shannon information sampling bias.^24^^,^^25^^,^^26^^,^^27^^,^^28^ However, to date the problem of whether and how synergy or redundancy components of PID are biased has not been studied systematically. While the problem of null models for information-theoretic quantities is gaining recognition,^29^ to our knowledge only two studies have investigated PID sampling bias, specifically for a Gaussian PID^30^ and for a PID with a mix of discrete and Gaussian variables.^31^ Here, we study systematically the sampling bias of both discrete and Gaussian estimators of PID with two or three sources. We find that synergy is far more biased than redundancy, especially in the discrete case. Using extensive numerical simulations accompanied by analytical considerations, we study and provide an understanding of the origin and properties of the bias. Based on this understanding, we provide effective methods that reduce this problem to provide accurate measures. Finally, we test our bias corrections on empirical neuroscience datasets, from the brain of mice, monkeys, and humans, of spikes recorded with either two-photon calcium imaging or electrophysiology and of functional MRI (fMRI) or magnetoencephalography (MEG) signals.

## Results

### Background: PID and its neuroscience use

For completeness, we first summarize the PID concepts we use. We focus this summary on the discrete PID, but the extension to Gaussian PID is straightforward (full details of discrete and Gaussian PIDs are given in methods). PID decomposes the information jointly carried by a set of source variables $\vec{R}$ (here, the activity of a set of simultaneously recorded neurons or brain areas, henceforth simply neural units) about a target *S* (here, a sensory stimulus or the activity of another simultaneously recorded neural unit) into components (non-negative for the PIDs we consider here) that capture information about the target that is either redundantly encoded across sources, uniquely encoded by each individual source, or synergistically encoded by the combination of sources, such that it is not available from any of them alone.^22^ We focus this introduction on the case of two source variables (that is, $\vec{R}=\left(R_{1},R_{2}\right)$), which has received the most attention and which has the most established theoretical foundations.^30^^,^^32^^,^^33^^,^^34^^,^^35^^,^^36^^,^^37^ Unless otherwise stated, all our results refer to the two-source PID, while the three-source PID is considered separately in later sections. We consider the Shannon information *I*(*S*;*R*_1_,*R*_2_) about the target variable *S* carried jointly by the activity of two neural units *R*_1_ and *R*_2_ and the Shannon information *I*(*S*;*R*_*i*_) that each of the two neural units (*i* = 1,2) carries about *S*. *I*(*S*;*R*_1_,*R*_2_) and *I*(*S*;*R*_*i*_) are computed from the probability distributions *p*(*s*, *r*_1_, *r*_2_) and *p*(*s*,*r*_*i*_) (*i* = 1, 2),^38^^,^^39^ as follows:

(Equation 1) $$I\left(S;R_{i}\right)=\sum_{s\in S,r_{i}\in R_{i}}p\left(s,r_{i}\right)\log\frac{p\left(s,r_{i}\right)}{p\left(s\right)p\left(r_{i}\right)}\text{,}$$

(Equation 2) $$I\left(S;R_{1},R_{2}\right)=\sum_{s\in S,r_{1}\in R_{1},r_{2}\in R_{2}}p\left(s,r_{1},r_{2}\right)\log\frac{p\left(s,r_{1},r_{2}\right)}{p\left(s\right)p\left(r_{1},r_{2}\right)}\text{.}$$

PID decomposes *I*(*S*;*R*_1_,*R*_2_) into the sum of four components, subject to three constraints involving the two single-source mutual information *I*(*S*;*R*_1_) and *I*(*S*;*R*_2_):

(Equation 3) $$I\left(S;R_{1},R_{2}\right)=Red\left(S:R_{1};R_{2}\right)+Unq\left(S:R_{1}\setminus R_{2}\right)+Unq\left(S:R_{2}\setminus R_{1}\right)+Syn\left(S:R_{1};R_{2}\right)\text{,}$$

(Equation 4) $$I\left(S;R_{1}\right)=Red\left(S:R_{1};R_{2}\right)+Unq\left(S:R_{1}\setminus R_{2}\right)\text{,}$$

(Equation 5) $$I\left(S;R_{2}\right)=Red\left(S:R_{1};R_{2}\right)+Unq\left(S:R_{2}\setminus R_{1}\right)\text{,}$$

where *Red*(*S*:*R*_1_;*R*_2_) is the redundant (or shared) information that both *R*_1_ and *R*_2_ encode about *S*; *Unq*(*S*:*R*_1_\*R*_2_) and *Unq*(*S*:*R*_2_\*R*_1_) are the unique information about *S* provided by one neural unit but not by the other; and *Syn*(*S*:*R*_1_;*R*_2_) is the synergistic information about *S* encoded jointly by *R*_1_ and *R*_2_. Equations 3, 4, and 5 imply that all four components contribute to the joint information, whereas only unique and redundant components contribute to single-neural unit information. Because the four PID components satisfy three linear constraints (Equations 3, 4, and 5), determining the value of one of the four components and the Shannon information values is sufficient to compute the other three PID terms (see equations of all components as a function of synergy in Equation 16).

Several definitions of PID have been proposed, which differ in their theoretical properties, including in whether redundancy and unique information do or not depend only on source-target pairwise marginals and in whether individual terms are or are not required to be non-negative.^40^^,^^41^^,^^42^^,^^43^^,^^44^^,^^45^ We consider in our study some of those that satisfy the two properties of non-negativity of each component and symmetry of redundancy and synergy under permutation of *R*_1_,*R*_2_, as the resulting components are clearly interpretable as fractions of the joint mutual information *I*(*S*;*R*_1_,*R*_2_) and are invariant to arbitrary reordering of the sources. In this section, we summarize the PID that we will use the most in our simulations unless otherwise stated, that is, the BROJA (Bertschinger-Rauh-Olbrich-Jost-Ay) PID (defined in Bertschinger et al.^33^ for discrete PID and in Venkatesh et al.,^30^ termed gPID, for Gaussian PID). It satisfies many desirable properties including additivity of all PID components for independent systems of sources and targets (proven for discrete^33^^,^^46^ and empirically validated for Gaussian PID^30^) and has been widely applied to neural data.^13^^,^^36^^,^^37^^,^^47^^,^^48^ The BROJA PID defines the union information, that is, the target information in the joint source space that cannot be possibly attributed to synergistic interactions or, equivalently, the total target information that can be extracted from a single source, as

(Equation 6) $$Union\left(S:R_{1};R_{2}\right)=\min_{q\in \Delta_{P}}I_{q}\left(S;R_{1},R_{2}\right)\text{,}$$

where Δ_*P*_ is the set of all joint probability distributions *q*(*s*,*r*_1_,*r*_2_) that have the same pairwise marginals *q*(*s*,*r*_1_) = *p*(*s*,*r*_1_) and *q*(*s*,*r*_2_) = *p*(*s*,*r*_2_) as the original distribution *p*(*s*,*r*_1_,*r*_2_), and *I*_*q*_(*S*;*R*_1_,*R*_2_) is the joint information computed for distribution *q*(*s*,*r*_1_,*r*_2_). The synergy is then defined as the difference between the joint and the union information:

(Equation 7) $$Syn\left(S:R_{1};R_{2}\right)=I\left(S;R_{1},R_{2}\right)-Union\left(S:R_{1};R_{2}\right)\text{.}$$

Other PID definitions that satisfy non-negativity and symmetry but do not satisfy additivity were also successfully applied to neuroscience and were used for both discrete and Gaussian calculations.^35^^,^^49^^,^^50^^,^^51^^,^^52^ These include the original PID termed *I*_*min*_^22^ (quantifying redundancy as the similarity between *R*_1_ and *R*_2_ in discriminating individual values of *S*), and the PID termed minimum mutual information *I*_*MMI*_^34^ (quantifying redundancy as the minimum between the mutual information individually carried by *R*_1_ and *R*_2_, thus capturing the amount but not the content of information carried by each neural unit^41^).

An approach extensively used in neuroscience for both Shannon information^3^^,^^10^^,^^13^^,^^48^^,^^53^^,^^54^^,^^55^^,^^56^^,^^57^^,^^58^^,^^59^^,^^60^^,^^61^^,^^62^^,^^63^^,^^64^ and PID^13^^,^^35^^,^^36^^,^^47^^,^^48^^,^^49^^,^^50^^,^^64^^,^^65^^,^^66^^,^^67^^,^^68^^,^^69^ is the discrete approach, in which all the variables are discrete or are discretized. The discrete PID can be computed by simply estimating the probabilities as the empirical occurrences across experimental samples (maximum-likelihood estimators) and plugging them into the PID equations (the so-called plugin approach^64^). The discrete approach is suitable for many neuroscience applications because traditional quantifications of neural spiking activity naturally lead to discrete variables. When focusing on spike count codes, the neural response is described by the number of spikes emitted by the neuron in the time window of interest.^53^^,^^54^^,^^56^^,^^57^^,^^58^^,^^70^^,^^71^ When investigating whether the timing of spikes encodes additional information above and beyond that present in spike counts, the most established approach^10^^,^^59^^,^^60^^,^^72^^,^^73^ is to discretize the neural response time window of interest into a number of small time bins and then turn the spike train into a binary word. In addition, in most experiments, often only a discrete number of different stimulus conditions is presented, usually with uniform probability, and thus the target *S* is often a discrete variable. In such conditions, as we show in Figure S13 and Note S5, the Gaussian PID may give inaccurate information values and PID components that are not credible (e.g., finding synergy or redundancy that clearly should not be present in the distributions). Because it is simple and does not require assumptions on the probability distributions, discretization of neural responses for computing information has also been used to compute information from continuous-valued non-spiking aggregate measures of neural activity such as local field potentials (LFPs), electroencephalography (EEG), or fMRI.^69^^,^^74^^,^^75^^,^^76^^,^^77^

Another approach extensively used in neuroscience is the Gaussian PID, which assumes Gaussian multivariate distributions for sources and target.^30^^,^^34^^,^^78^ The Gaussian PID can be computed from the covariance matrix, as this matrix fully defines the Gaussian probability distributions. The Gaussian PID is thus empirically computed from real data by estimating covariance matrices from the data and inserting them into the PID equations (see methods). Gaussian PIDs are well suited to analyze the activity of continuous-valued non-spiking aggregate measures of neural activity such as LFP, EEG, or fMRI.^49^^,^^51^

### Bias of synergy and redundancy for discrete and Gaussian estimators

Calculations of information and PID require the accurate estimation of the target-source joint probabilities. With an infinite amount of data, the true probabilities (or the parameters defining them) could be measured exactly. However, any real neuroscience experiment will only yield a finite number of samples *N* that can be used to estimate the probabilities. For example, in some neuroscience experiments a sensory stimulus (the target) will be presented in a given trial, and the activity of different neural units (sources) will be recorded. If the neural activity is quantified as a single number per trial (for example, the spike count),^56^^,^^70^ the number of samples *N* to compute the probabilities will equal the number of trials. If several samples of neural activity can be taken in each stimulus presentation trial,^63^^,^^79^ the number of samples will be equal to the number of trials times the number of samples per trial. In other cases, such as when computing information between the activity of some neural population (the target) and the activity of another neural population (the sources) in resting-state conditions, a trial structure may be absent, and the number of samples equals the number of time points at which activity of the different populations can be sampled during the experiment.^35^^,^^49^

**Figure 1:**
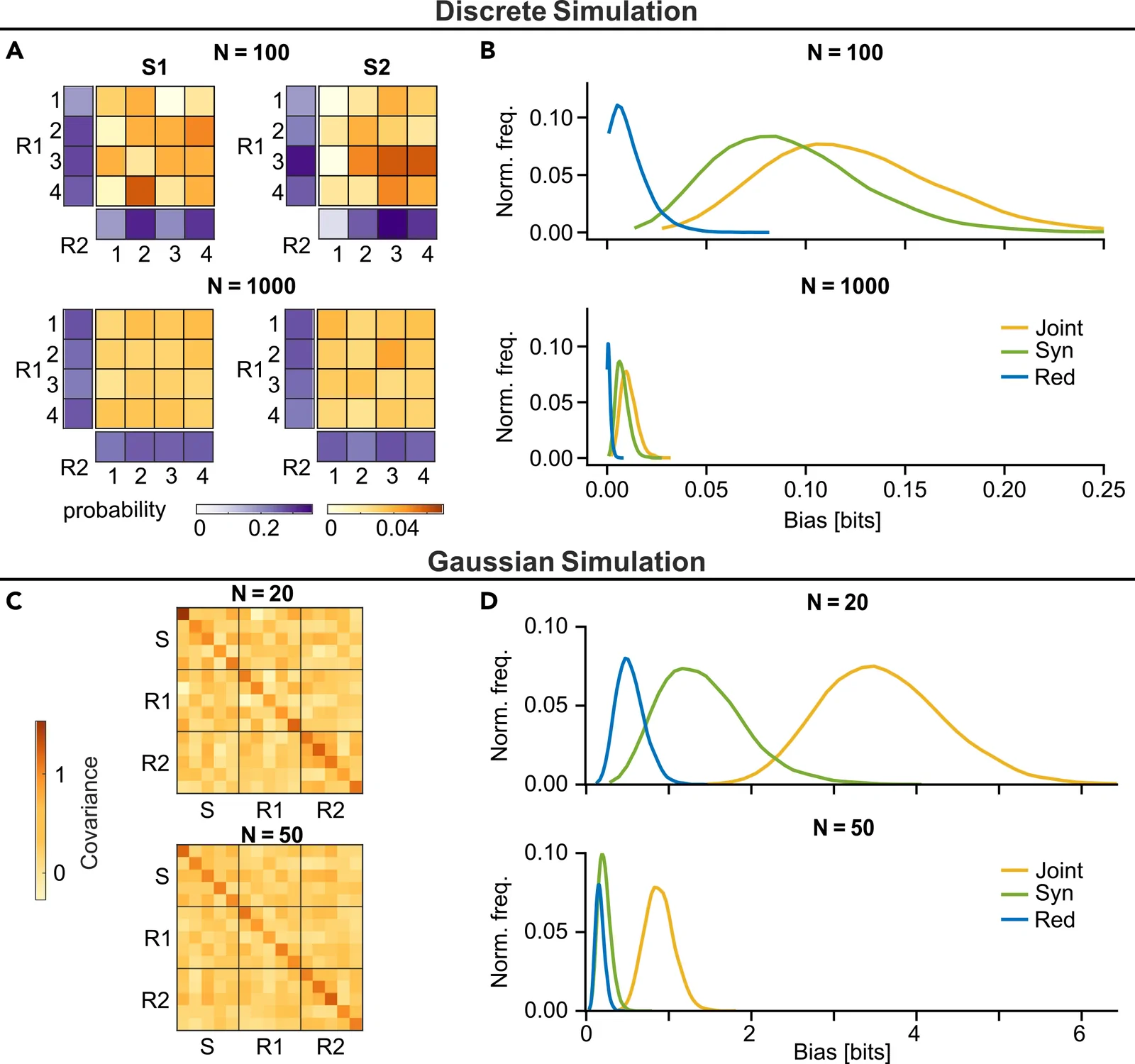
Illustration of the origin of the limited-sampling bias (A) Illustration of the bias for the discrete BROJA PID. Two uninformative neural units (in this example, two spiking neurons) respond with spike counts uniformly distributed from 1 to 4 (i.e., *R* = 4 bins) independently of which of *S* = 2 stimuli (target) is presented. Empirical stimulus-specific joint response probabilities are estimated from *N* = 100 (top) and *N* = 1,000 (bottom) samples and plotted with an orange color scale. Marginal distributions are shown at the margins (blue color scale). Deviations from uniformity are evident and are stronger for joint than for marginal distributions and for lower *N*. (B) Distribution (over 5,000 simulations) of the plugin (i.e., not bias-corrected) joint information, synergy (*Syn*), and redundancy (*Red*) values obtained with 100 (top) and 1,000 (bottom) samples respectively. (C) Illustration of bias for the Gaussian gPID. Empirical covariance matrices are estimated from *N* = 20 (top) and *N* = 50 (bottom) samples and plotted with an orange color scale. Presented stimuli (*S*) and responses of the two uninformative neural units (*R*_1_ and *R*_2_) are five-dimensional independent and identically distributed Gaussian variables with zero mean and unit variance. Deviations from diagonal covariance matrices are evident and stronger for lower *N*. (D) Distribution (over 5,000 simulations) of the plugin (i.e., not bias-corrected) joint information, synergy (*Syn*), and redundancy (*Red*) values obtained with 20 (top) and 50 (bottom) samples, respectively. For details, see Note S3.

When real-data probabilities are estimated from the finite number of available samples *N*, both the probabilities to be estimated for the discrete PID and the covariance matrices needed for the Gaussian PID have finite sampling fluctuations around their true values, which make the distributions of sources typically more dependent on the target than they really are (Figures 1A and 1C). As a result, the values of information estimated from a finite amount of samples are not centered around the underlying ground-truth information value (Figures 1B and 1D). This leads to both a systematic error (limited-sampling bias) and a statistical error (limited-sampling variance) in information estimates. While variance can be reduced by averaging (e.g., across experimental subjects or groups of neural units), the limited-sampling bias (shortened to bias hereafter) cannot. Although the bias of Shannon information has been studied extensively for both discrete^24^^,^^25^^,^^27^ and Gaussian estimators,^80^^,^^81^ the bias of individual PID components has remained largely unexplored. Here, we study the bias of the discrete and Gaussian PIDs with numerical simulations, supported by analytical considerations (see Note S6). We simulated (for details see methods) either spike count or Gaussian processes with non-zero ground-truth values for each PID component (because this is the most general case, and it mimics the fact that all PID components had non-zero values in all real neural datasets we investigated). We varied the overall joint information level of these processes to investigate how the bias depended on it (see Figures S4, S5, S9, S10, and S11 for tests on simulated scenarios with some PID components having a null ground-truth value). We focused on the two-source BROJA PID (discrete as in Bertschinger et al.^33^ and Gaussian as in Venkatesh et al.^30^), but we confirmed our results with the two-source *I*_*min*_ and *I*_*MMI*_ (Figures S6 and S7). To simplify presentation, we focused on the bias of redundancy and synergy, as these components are of the highest interest for studying neural interactions. However, the bias properties of the sum of the unique information terms can be inferred by difference between the bias of the joint information and that of synergy and redundancy and will be recapitulated in Figure S2.

**Figure 2:**
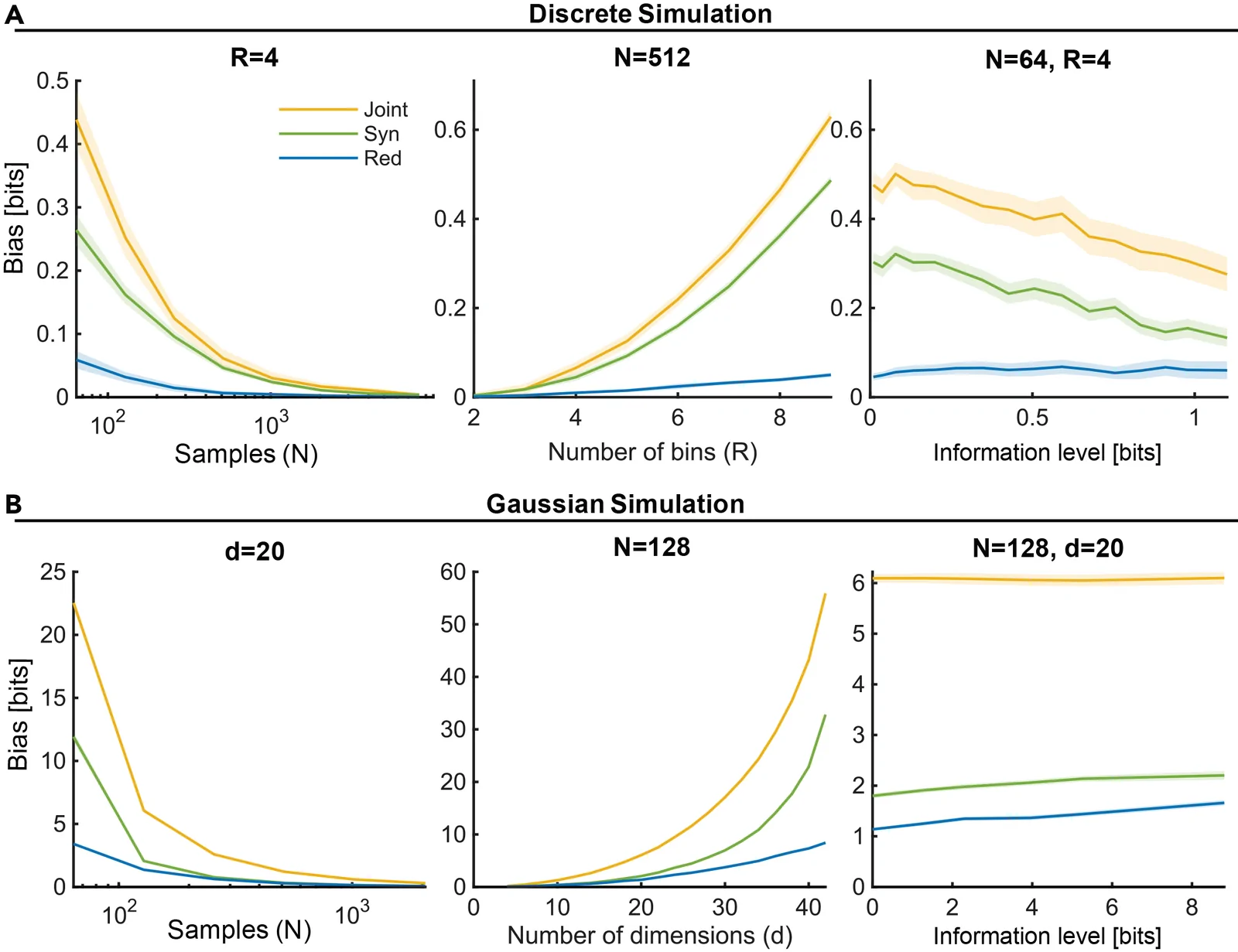
Bias of the plugin PID across number of samples, bins or dimensions, and information level The bias of the plugin (i.e., not bias-corrected) PID terms—joint information (*Joint*), synergy (*Syn*), and redundancy (*Red*)—is computed as averaged empirical estimates from *N* samples minus ground-truth values (see methods). (A) Discrete BROJA PID simulations. Left: we vary (on a log *x* axis) the number of samples *N* used to compute the PID (number of bins *S* = *R* = 4) in the low information value regime. Middle: we vary the number of bins *S* = *R* (number of samples *N* = 512) in the low information value regime. Right: we vary the joint information level by changing the value of the simulation parameter *α* regulating the stimulus-response gain (*N* = 64, *S* = *R* = 4). (B) Gaussian gPID simulations. Left: we vary (on a log *x* axis) the number of samples *N* used to compute the PID components (number of dimensions *d* = 20) in the low information value regime. Middle: we vary the number of dimensions *d* of sources and targets (*N* = 128) in the low information value regime. Right: we vary the joint information level by changing the value of parameter *α* scaling the diagonal of the covariance matrix (*N* = 128, *d* = 20). We plot mean ± 3 SEM over 100 simulations. Discrete and Gaussian simulations were performed under the bit-of-all scenario (see methods and Table S2). For more details, see Table S1.

We first studied the discrete two-source PID. We simulated (Figure 2) spike counts of two individual neurons (sources), each binned into equipopulated bins, emitted in response to different stimuli (target). In this simulation, and in all others presented in this paper, one sample per stimulus presentation (trial) was collected, and thus the number of samples *N* to compute probabilities equaled the number of simulated trials. We studied how the plugin estimate of the joint information and of each PID component depends on the number of available samples (in all simulation figures, we averaged over *n* = 100 simulation repetitions). We term the plugin estimate^27^^,^^64^ as the one that computes information directly plugging the empirical probabilities into the information and PID equations (Equations 1, 2, 3, 4, and 5). The limited-sampling bias in the plugin information estimates is the difference between the average across simulations of the information obtained with a given number of samples and the ground-truth value computed from the true probabilities. As previously reported,^27^^,^^60^ the plugin estimate of the joint Shannon information has an upward bias, which decreases with the number of samples *N* (Figures 1B and 2A [left]), increases with the number of discrete bins *R, S* of each source and target variable (Figure 2A, middle, where we keep *R* = *S* for simplicity), and decreases with the information level (Figure 2A, right). These properties are explained by analytical results (obtained in the large-*N* limit; Note S6) showing that the bias decreases with *N* as powers of 1/*N* and that it is proportional to the number of discrete bins *R*, *S*. The decrease of the bias with the information level can also be explained from these analytical calculations,^24^ because the bias increases with the number of discrete responses that are possible (i.e., with non-zero probability). Low information levels correspond to less concentrated stimulus-specific distributions and, thus, have a larger number of possible responses and are more biased.

We then studied the bias of the plugin estimates of redundancy and synergy computed with the discrete PID. We found several highly consistent results (Figure 2A). First, as for the Shannon joint information, synergy and redundancy have an upward bias decreasing with *N* (Figure 2A, left). Second, for the tens of samples usually available in neuroscience experiments, the bias could be as large as, or even larger than, the target quantities (Figures 2A and S5). This implies that the bias must be corrected for in real-data analyses. Third, the bias of the synergy is larger both proportionally and in absolute terms for lower information levels (Figure 2A, right). Fourth, and unlike what was assumed in a previous study,^30^ the bias is highly uneven across PID components. When varying, at fixed *N*, the number of discrete bins *R*, *S* of each source and target variable (keeping *R* = *S* for simplicity), the bias of the synergy increases rapidly and supralinearly with *R* (Figure 2A, middle), whereas the bias of the redundancy grows much more slowly and is much smaller. The bias of the sum of the Unique information terms (which is the difference between the bias of the joint information and the biases of synergy and redundancy) is plotted in Figure S2A. It increases with 1/*N* and *R* and is in most cases intermediate between that of synergy and redundancy in terms of size and rate of growth with 1/*N* and *R*.

We next study the bias of the Gaussian PID (Figure 2B). The Gaussian joint information has an upward bias that decreases with the number of samples *N* (Figure 2B, left) and increases with the dimensionality *d* of the sources and target (Figure 2B, middle). As predicted by an exact analytical bias expression valid for *N* > 3*d*,^80^^,^^81^ the joint information bias depends only on *N* and *d* but is independent of the information level (Figure 2B, right). Synergy and redundancy are also biased upward. Because the Gaussian joint information bias does not depend on the information level, the bias of synergy and redundancy does not increase for lower information levels as in the discrete case (Figure 2B, right). Instead, the synergy and redundancy bias very slightly increases with the information level. Importantly, the bias of the Gaussian PID is also highly uneven between synergy and redundancy. When varying *d* at fixed *N* (Figure 2B, middle), the bias of both synergy and redundancy increases with *d*, but it increases faster for synergy than for redundancy. The bias of the sum of the Unique information terms is plotted in Figure S2B. It increases with 1/*N* and *d* and is in most cases intermediate between that of synergy and redundancy in terms of size and rate of growth with 1/*N* and *d*.

**Figure 3:**
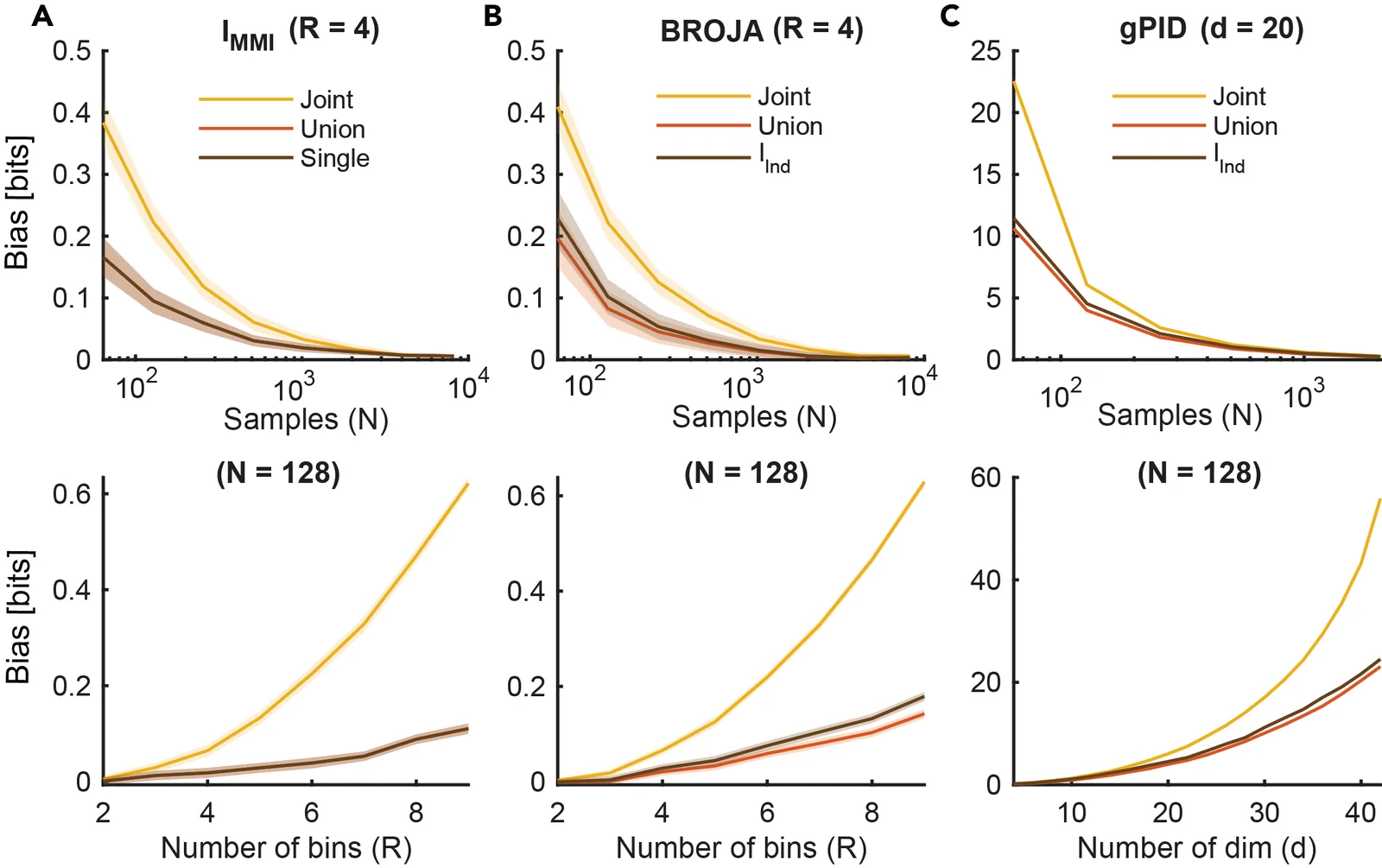
Simulations comparing the bias of union information to that of other information-theoretic quantities with an analytically tractable bias (A) Discrete simulations (using MMI PID) showing the bias (averaged empirical estimates from *N* samples minus ground-truth values) as a function of the number of simulated samples *N* (on a log *x* axis) using *S* = 4 stimuli and *R* = 4 bins per source (top) and as a function of the number of stimuli *S* and response bins *R* fixing *R* = *S* (bottom, with *N* = 128). The bias of the union information matches the one of the single-source information (“single”) because in MMI the union information equals the maximum single-source information. (B) Same as (A) (discrete simulations) but using the BROJA PID. The bias of the union information is compared to that of the conditionally independent joint information *I*_*ind*_, whose bias depends on the fluctuations of the marginal probabilities of one target and one source and is thus less than that of the joint information. (C) Gaussian simulations using the Gaussian gPID computed as a function of the number of simulated samples *N* using *d* = 20 dimensions for target and sources (top) and as a function of the dimension *d* of sources and target (bottom, *N* = 128). The bias of the union information is compared to that of the conditionally independent information *I*_*ind*_, whose bias depends on the fluctuations of the marginal probabilities of one target and one source. Results in each panel are plotted as mean ± 3 SEM over 100 simulations with the considered number of samples. Discrete and Gaussian simulations were performed under the bit-of-all scenario (see methods and Table S2). For more details, see Table S1.

The fact that the bias of the synergy is consistently larger than that of the redundancy and that their difference increases with *R* for the discrete PID and with *d* for the Gaussian PID can be understood from analytical considerations, developed in Note S6 and briefly summarized here. The synergy bias equals the difference between the biases of joint and union information, whereas the redundancy bias equals the difference between the sum of single-source information biases and the union information bias. The bias of synergy and redundancy is thus determined by the bias of these quantities. For the discrete case, exact expressions for the bias are not known. However, approximate expressions are available in the large-*N* limit.^24^ The bias of the joint information scales as (*S* × *R*^2^)/*N* (as it requires the sampling of the joint distribution of *s*, *r*_1_, *r*_2_), while the bias of single-source information scales as (*S* × *R*)/*N* (it requires the sampling of the joint distribution of *s* and of one source). The MMI union bias equals the bias of the maximum single-source information (Equation S12) and thus scales as (*S* × *R*)/*N* and is smaller than the joint information bias (Figure 3A). The union information depends only on marginal distributions of the target and one source also for the BROJA PID. We therefore conjectured, and confirmed numerically (Figure 3B), that the bias of the BROJA union information approximately matches the analytically tractable bias of the conditionally independent information *I*_*ind*_ (computed from the conditional-independent distribution *p*_*ind*_, which belongs to the set Δ_*P*_, in which the union information is computed), which can be shown to scale as (*S* × *R*)/*N*. In sum, for both MMI and BROJA discrete PID the synergy bias scales as (*S* × *R*^2^)/*N*, whereas redundancy bias grows at most as (*S* × *R*)/*N* as *R* increases. In the Gaussian case, an exact expression is available^81^ for the mutual information bias, which grows steeply and supralinearly when increasing the dimension *d* of sources and targets and decreases when increasing *N* (Figure S14). The bias increases much faster with *d* for the joint information (scaling as a supralinear function of 3*d*, as it contains bias of the entropy of the joint distribution of two sources and one target) than for the single-source information (scaling as a supralinear function of 2*d*, as it contains bias of the entropy of the joint distribution of one source and one target). As for the discrete case, the bias of the MMI union information scales as the single-source information. For the gPID^30^ (the Gaussian analog of the discrete BROJA PID), we also conjectured—and confirmed numerically—that the union bias is similar to the analytically tractable bias of the conditionally independent information *I*_*ind*_ (Figure 3C), which, as for the bias of the single-source information, scales as a supralinear function of 2*d*. The Gaussian MMI and the gPID synergy bias are thus dominated by the bias of entropies of 3*d*-dimensional distributions that scale as a supralinear function of 3*d*, whereas the redundancy bias contains only biases of entropies of 2*d*-dimensional distributions that scale as a supralinear function of 2*d*. As a result, in the Gaussian case, the synergy is more biased than the redundancy, and the gap in bias between the two grows supralinearly with *d*.

To recapitulate, for the discrete PID, the bias of the synergy scales with the number of bins as (*S* × *R*^2^)/*N* and is much larger than the bias of redundancy, which scales as (*S* × *R*)/*N*. For the Gaussian PID, the bias of the synergy also grows faster with the source dimension *d* than that of the redundancy. The uneven distribution of bias between synergy and redundancy is practically important because it implies that conclusions taken from limited empirical data without correcting for the bias may offer a distorted view of information coding, artificially skewed toward synergy. For example, considering simulations with ground-truth values of redundancy larger than synergy, not correcting for the bias would lead to incorrectly estimating synergy larger than redundancy for low trial numbers (Figure S5A, third row).

### Corrections for the limited-sampling bias of synergy and redundancy

We next consider how to correct synergy and redundancy for the limited-sampling bias. We first study the discrete PID, for which no previous bias correction was proposed. We introduce two complementary approaches (see methods for full implementation details). The first approach estimates the limited-sampling bias while preserving the information level of the real data. Based on the fact that (Figure 2A) the bias of each PID term decreases smoothly with the number of samples *N*, this approach (termed the quadratic extrapolation [QE] and introduced in Strong et al.^60^ for Shannon information) fits a 1/*N* polynomial to PID terms recomputed on half and a quarter of the available data and uses this fit to estimate and remove the bias. The second approach (“shuffle subtraction”) corrects for the bias by subtracting from the plugin values an estimate of the bias obtained by computing PID terms after randomly shuffling the source-target associations to remove all information about the target from the sources. This approach, which has been employed in the mutual information literature with numerous variations of how to destroy information by data randomization,^27^^,^^79^^,^^82^ assumes implicitly that the bias has similar magnitude across information levels (an assumption that we will investigate below).

**Figure 4:**
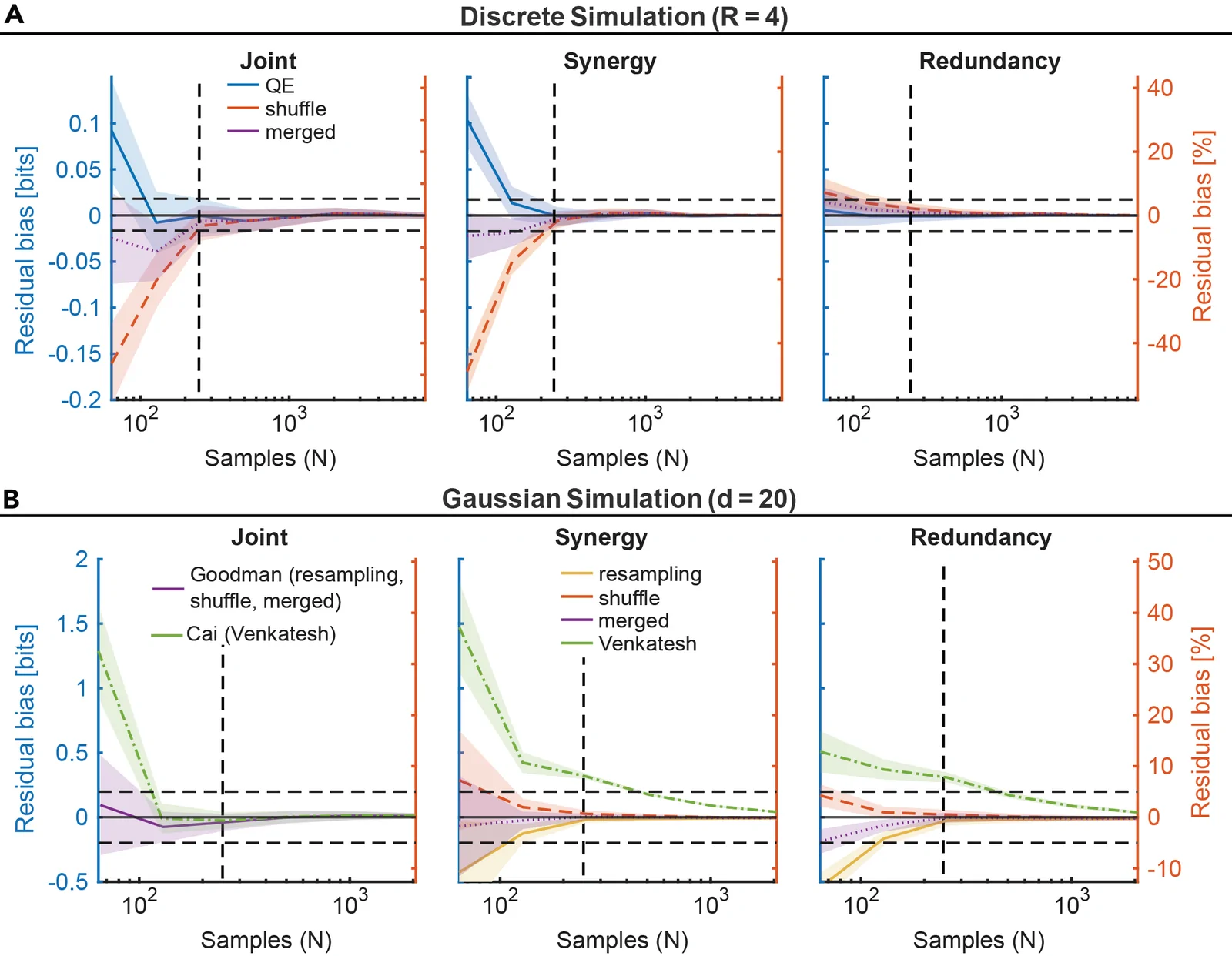
Residual bias after the implementation of different bias-correction methods The residual bias (averaged bias-corrected empirical estimates from *N* samples minus ground-truth values) is plotted (on a log *x* axis) as a function of the number of samples *N* used to compute the joint information (left), the synergy (*Syn*; middle), and the redundancy (*Red*; right). The values of the residual bias are plotted in units of bits on the left *y* axis and as a percentage of the ground-truth joint information on the right *y* axis. (A) Discrete BROJA PID simulations (number of bins *S* = *R* = 4) in the low information value regime. Corrections used for information quantities: QE, shuffle subtraction, and merged. (B) Gaussian gPID simulations (number of dimensions *d* = 20) in the low information value regime. Corrections used for synergy and redundancy: resampling, shuffle subtraction, merged, and Venkatesh. For joint information, we used the Goodman correction^81^ in the resampling, shuffle-subtraction, and merged methods as it is an exact correction, and for the Venkatesh correction we used the Cai et al. correction^80^ to replicate what was done in Venkatesh et al.^30^ We plot mean ± 3 SEM over 100 simulations. Dashed vertical lines mark the boundary of the sufficient-sampling regime. Horizontal dashed lines are ±5% boundary lines. Discrete and Gaussian simulations were performed under the bit-of-all scenario (see methods and Table S2). For more details, see Table S1.

**Figure 5:**
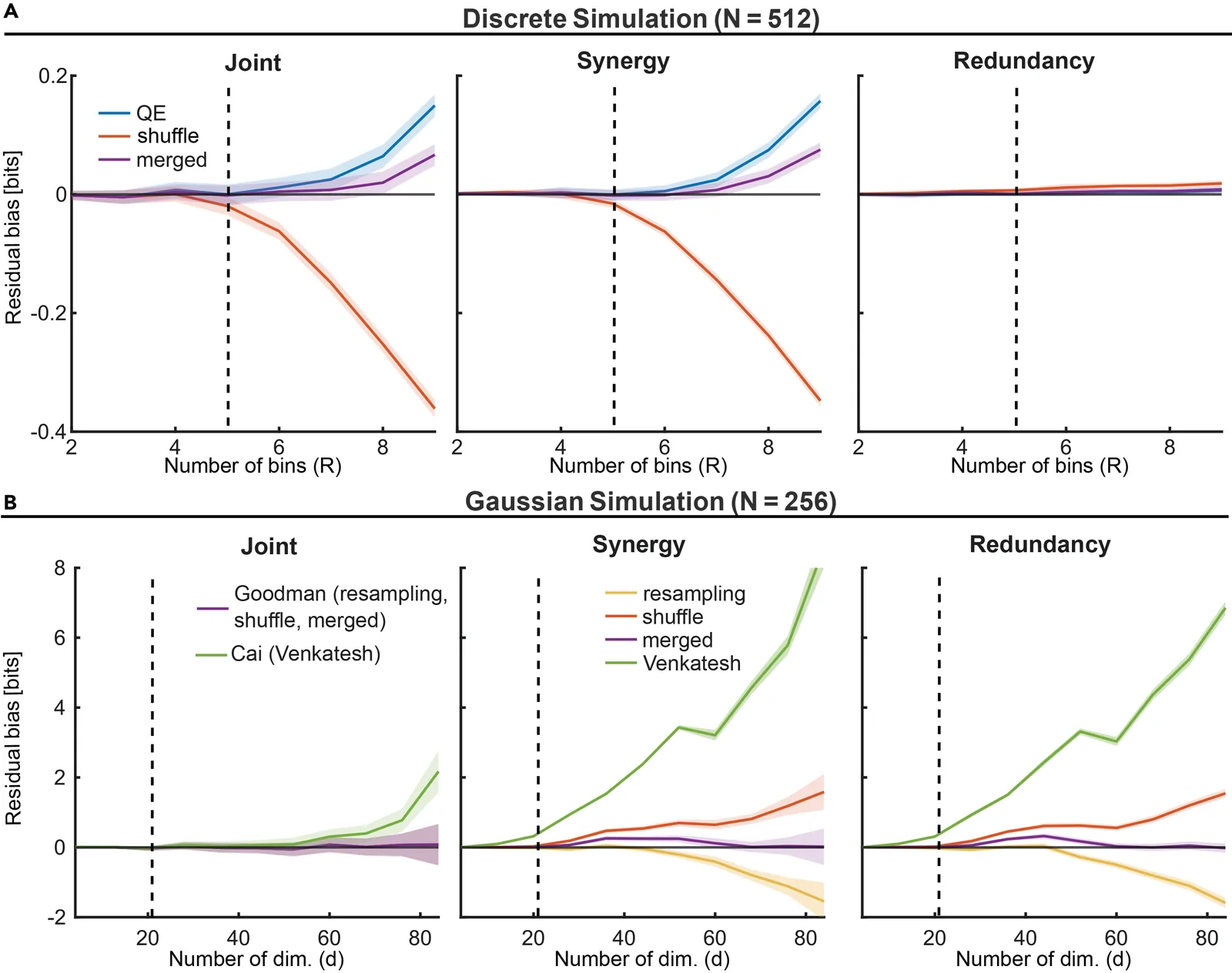
Performance of bias-correction methods as a function of the number of parameters defining the probability distributions The residual bias (averaged bias-corrected empirical estimates from *N* samples minus ground-truth values) of joint information (left), synergy (*Syn*; middle), and redundancy (*Red*; right) after applying different correction techniques is plotted as a function of the number of parameters defining the probability distributions. (A) Discrete BROJA PID simulations. We vary the number of discrete stimuli *S* and of discrete response bins for each neural unit *R*, with *S* = *R* (number of samples *N* = 512). Corrections used for all information quantities: QE, shuffle subtraction, and merged. (B) Gaussian gPID simulations. We vary the number of dimensions *d* of sources and targets (*N* = 256). Corrections used for synergy and redundancy: resampling, shuffle subtraction, merged, and Venkatesh. For joint information, we used the Goodman correction in the resampling, shuffle-subtraction, and merged methods as it is an exact correction, and for the Venkatesh correction we used the Cai et al. correction^80^ to replicate what was done in Venkatesh et al.^30^ We plot mean ± 3 SEM over 100 simulations. Dashed vertical lines mark the boundary of the sufficient-sampling regime. Discrete and Gaussian simulations were performed under the bit-of-all scenario (see methods and Table S2). For more details, see Table S1.

To estimate the effectiveness of the corrections, we plotted the residual bias (defined as the bias still present after applying a bias correction) as a function of the number of samples (Figure 4A), the number of discrete bins (Figure 5A), or the information level (Figure S8A). We find that both corrections reduce considerably the bias of the joint information and of the PID components (especially of the synergy, which is more biased). We consistently find across simulations that both QE and shuffle subtraction worked well for both synergy and redundancy as long as *N* is larger than 4 × *S* × *R* × *R* (dashed vertical line, Figure 4A) or equivalently that $R<\sqrt{\left(N/4S\right)}$ (Figure 5A). We call this sampling condition the “sufficient-sampling regime for discrete PID.” The good performance of these bias corrections within this regime was found across all simulation parameters and across all considered PID types (Figures 4A, 5A, and S3–S7). In the sufficient-sampling regime, the small residual error for synergy and joint information is mostly upward for QE (intuitively because bias terms of higher order in 1/*N*, not included in the QE, are all positive^83^) and downward for the shuffle correction (because lower information levels have larger upward bias). This suggests that computing both QE and shuffle subtraction from real data provides reasonable empirical bounds to the information estimate for synergy. It also led us to implement a third and highly effective bias correction (termed the merged correction) that estimates the bias as a linear combination of the bias obtained with QE and shuffle subtraction, with weight optimized to minimize the absolute value of the residual bias on a simulation training set with many information levels (see methods). The merged correction is highly effective because it evens out the opposite tendencies of the QE and the shuffle subtraction (Figures 4A, 5A, and S8A). It leads to very small residual biases throughout the sufficient-sampling regime for both synergy and redundancy, and it still works well even in conditions of severe undersampling—well outside the sufficient-sampling regime. Comparing Figure 2A (left) with Figure 4A shows that not correcting for the bias would have led to large errors even in the sufficient-sampling regime and that to obtain, without using bias corrections, information measures as accurate as the ones corrected with the merged correction would have needed orders-of-magnitude more samples. For a fixed number of samples, using any of the bias corrections, but especially the merged correction, extends considerably the range of discrete number of bins for which information can be estimated accurately. For example, when using *N* = 512 samples, information estimates remain accurate (small residual bias) up to six discrete bins (Figure 5A), whereas not using a bias correction would have led to small residual errors only when using two discrete bins (Figure 2A). These findings hold also for the Unique information (Figure S2). Finally, when applying the merged correction and the other corrections in the sufficient-sampling regime, accurate estimates with small residual biases are obtained regardless of the information level (Figure S8A), highlighting the robustness of the results.

In sum, for the discrete PID we recommend the use of the merged bias correction, which gives precise results for both synergy and redundancy in the sufficient-sampling regime and often beyond. Using this bias correction leads to much more precise estimates than using no bias corrections and extends the range of applicability of the method to larger numbers of bins and smaller number of samples. To help users, we computed numerically an estimate of the tolerance (estimated magnitude of the absolute value of the residual bias; Figure S15) within the sufficient-sampling regime as a function of the fraction *R*^3^/*N* (the parameter ratio setting the scaling of the bias of the synergy). This estimate of the residual bias helps in both designing the experiments (setting the minimal number of samples needed for a given tolerance) and designing analyses (setting the maximal number of bins that, given the number of samples, would allow being in the sufficient-sampling regime and achieving errors within a chosen tolerance).

We then consider the Gaussian PID. For this, one bias-correction procedure was previously proposed,^30^ which we term the Venkatesh correction. The Venkatesh correction assumes that the union information has the same proportional bias as the joint information (and thus of the synergy, see methods), and it then rescales all PID accordingly (Equations 3, 4, and 5). However, we found that the union information is much less biased upward than the joint information (Figure 3). Thus, this procedure is expected to lead often to a major underestimation of union information, which in turn leads to a severe overestimation of synergy and redundancy (see methods), which persists also for large *N*. In our simulations, the Venkatesh bias correction left a residual error that could be as big as 30%–40% of the true joint information value (Figures 4B, 5B, S9, and S10). We thus introduce two alternative correction procedures that, as in the discrete case, either operate with information levels equal to those of the data or operate by removing all information from the data. The first correction, termed resampling correction, estimates the bias as the difference between the mean information value obtained by repeatedly generating *N* samples from the distribution estimated from the real data and the information value computed from the PID equations using the covariance matrix estimated from the real data. The resampling correction works well for the Gaussian but less so for the discrete PID, because the overall bias of the joint Gaussian information does not depend much on the information level (Figures S9 and S10). We chose it to replace the QE (used in the discrete PID) for the Gaussian PID, as we verified that QE does not work well for the Gaussian information because its bias is non-polynomial.^81^ The second is the shuffle correction, obtained, exactly as for the discrete PID, by estimating the bias on shuffled data and subtracting it. Both corrections reduce by orders of magnitude the bias of the joint information and of synergy and redundancy, leaving a residual bias that was just a small fraction of the uncorrected bias (Figures 4B, 5B, S3, S8B, S9, and S10). As expected intuitively, the shuffle correction works better than resampling for lower but not for higher information levels (Figure S10). Because of the above-described dependence of the bias of the PID components on the information level (Figure 2), the small residual error for synergy and redundancy information was typically downward for the resampling correction and typically upward for the shuffle correction. Because of the opposite trend of their residual errors, we also averaged the resample and shuffle corrections to obtain a merged correction, which further reduces the residual bias.

Across simulations, the merged, resampling, and shuffle corrections consistently leave a negligible residual error for *N* > 4 × 3*d* or *d* < *N*/12 (Figures 4B, 5B, S3, S8B, S9, S10, and S11), a condition that we term the “sufficient-sampling regime for Gaussian PID.” These findings hold also for the Unique information (Figure S2). All our Gaussian bias corrections improve considerably the existing Venkatesh correction.^30^ Also in the Gaussian case, the merged correction is the correction that works better and provides accurate estimates (small residual biases) even for numbers of samples or dimension values well outside the sufficient-sampling regime. In Figure 5, the correction left residual errors of the order of 0.1 bits or smaller against original uncorrected bias values that were of the order of several bits. We thus recommend the use of this merged correction for the Gaussian PID. The merged correction extends the applicability of the PID to much smaller sample sizes and a much higher number of dimensions compared to uncorrected estimates and to the previous available Venkatesh correction (Figure 5B). As for the discrete PID, we provide in Figure S15 a numerical evaluation of the absolute value of the residual error expected as a function of the fraction 3*d*/*N* (which is expected to set the synergy bias from analytical considerations). This allows users to design numbers of samples or trials and set dimensionalities that can be analyzed given a chosen level of tolerance in residual errors.

### Evaluation of bias-correction procedures on real neural data

We evaluated the empirical utility of the PID bias correction using four datasets from previously published studies, which span different species, recording modalities, and cognitive-task designs.

We first consider three datasets of spiking activity recorded from animals performing cognitive tasks (see methods for full experimental details). For all datasets, analysis parameters and trial numbers meet the sufficient-sampling regime. We use discrete PID because it is natural for spiking activity and discrete stimuli and because Gaussian PID gave extremely low information values for two of these datasets (Figure S13 and Note S5).

**Figure 6:**
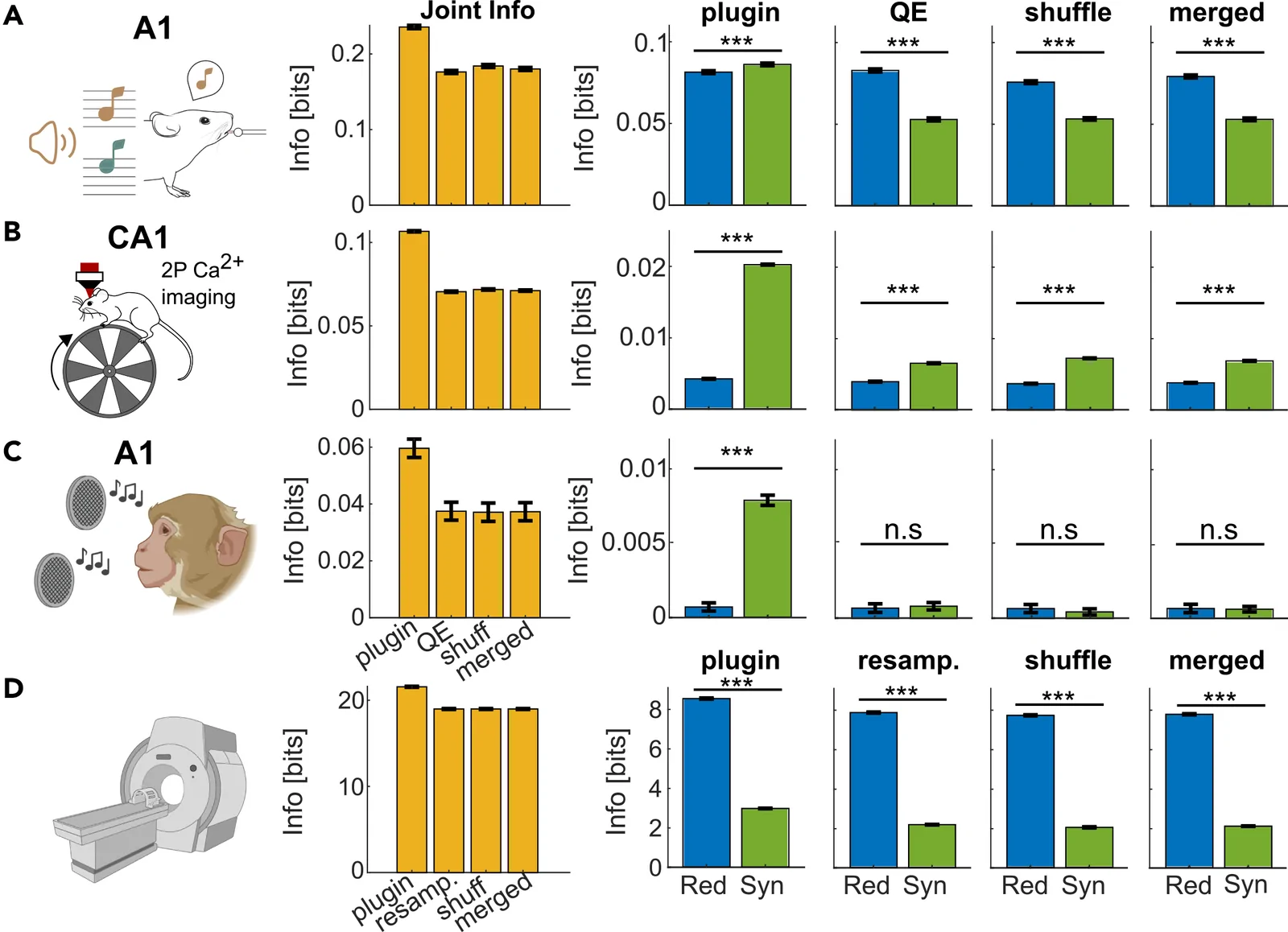
Application of PID bias corrections to real neural data (A–C) Reporting the discrete BROJA PID of the joint information between an external stimulus (*S*, target) and the joint activity of two neurons (sources). In (A) (dataset 1), *S* is the identity of a binary auditory tone, and neuronal responses are spike trains obtained by deconvolving calcium signals recorded from mouse auditory cortex A1 (spike counts binned in *R* = 3 levels). In (B) (dataset 2), *S* is the mouse location (discretized in 12 spatial bins) on a linear track, and neuronal responses are calcium signals recorded from the CA1 hippocampal region (discretized in *R* = 2 bins). In (C) (dataset 3), *S* is the identity of one of 399 natural sounds, and neuronal responses are electrophysiologically recorded spike trains from monkey A1 (spike counts binned in *R* = 2 levels). (D) Reporting the Gaussian gPID of the joint information between the activity of one putative input brain region at a given time (target) and the activity of two other brain regions (sources) at an earlier time (dataset 4). Each panel shows mean ± 3 SEM of information values over all analyzed simultaneously recorded neural pairs (*n* = 6,209, 36,158, and 83 for (A), (B), and (C), respectively) or triples of brain regions (D, *n* = 15,000 triplets of sets of 20 ROIs randomly drawn among 1,000 ROIs). The mean number *N* of available samples per session is 140, 870, and 43,092 for datasets 1–3, respectively. For dataset 4, information values are computed across 256 samples (different time points). Left to right: schematic of each experiment; joint information results with plugin (no correction) or different bias-corrected estimators QE (resample for fMRI), shuffle subtraction, and merged; redundancy and synergy results with plugin or bias-corrected estimators. Comparisons between synergy and redundancy were performed with a two-tailed paired *t* test (∗∗∗*p* < 0.001; n.s., *p* > 0.05). Details are provided in methods. Image credits: (A) mouse sketch adapted from https://doi.org/10.5281/zenodo.3925985, speaker icon from svgrepo.com, and music note icon from svgrepo.com, all modified and licensed under CC BY 4.0; (B) created by the authors; (C) created by the authors in BioRender; and (D) created by the authors in BioRender.

Dataset 1 (Figure 6A) consists of *n* = 6,209 pairs of neurons (mean number of trials per session: 140) simultaneously recorded with two-photon calcium imaging from auditory cortex (A1) during a sound intensity discrimination task.^36^ We compute the information that neurons carry about the intensity of the presented sound (a binary variable, *S* = 2, high vs. low tone intensity). Each neuron’s spiking activity is estimated by deconvolving the calcium fluorescence traces. The single-neuron response variables *r*_1_ and *r*_2_ are computed by summing the deconvolved activity within a 333-ms time window centered around the time of maximal information and discretizing it into *R* = 3 bins^36^ (thus, one sample per trial is collected).

Dataset 2 (Figure 6B) consists of *n* = 36,158 pairs of neurons simultaneously recorded with two-photon calcium imaging from the CA1 region of the hippocampus^63^ while mice navigated a linear track in virtual reality. Because the hippocampus encodes spatial position, we compute the information that neurons carry about the spatial location (linear track discretized in *S* = 12 spatial bins with on average 870 trials per session). The spiking activity for *r*_1_ and *r*_2_ is obtained, following Curreli et al.,^63^ discretizing the fluorescence traces into *R* = 2 bins (low vs. high activity). For this dataset, we had 77–125 samples per spatial location bin.

Dataset 3^11^ (Figure 6C) consists of *n* = 83 pairs of neurons simultaneously recorded with electrodes from the auditory cortex (A1) of a macaque monkey listening to natural sounds. We compute information carried by neural activity *r*_1_, *r*_2_ (defined as spike counts discretized in *R* = 2 bins) concerning which of *S* = 399 different sound stimuli (segments of natural sounds each 50 ms long) were presented (mean number of trials per session: 108 for each of the 399 sound stimuli; one sample of neural activity per trial was collected).

We compute the joint information between the above-defined activity *r*_1_, *r*_2_ of simultaneously recorded pairs of neurons (PID sources) and the above-defined stimulus *s* (PID target). We used the discrete BROJA PID,^33^^,^^84^ as spike responses are better described with discrete distributions, and the stimuli also had discrete values. We focus the analysis and the neuroscientific interpretation on the differences between synergy and redundancy to understand how these two emergent properties shape neural population coding in different brain regions. To test and exemplify the use of the bias-corrected PID algorithms, we compare the plugin PID estimates with those obtained after applying our three bias corrections (Figures 6A–6C).

As shown by the smaller values obtained after correcting for the bias, the plugin (uncorrected) joint information is biased upward by ≈30%–50% (Figures 6A–6C). As predicted by our simulations, redundancy is almost unbiased (shown by the fact that it does not change value after bias corrections; Figures 6A–6C). Despite these datasets having a good number of trials compared to typical experiments, the synergy bias is major, as the difference between bias-corrected and plugin synergy estimates is approximately 60%, 200%, and 800% of the target values across the three datasets (Figures 6A–6C). Using the uncorrected estimator would have thus led to a considerable overestimation of the synergy and to errors in conclusions. In dataset 1 (Figure 6A), using the uncorrected plugin estimator would have led to the erroneous conclusion that synergy was larger than redundancy, whereas all three bias-corrected estimates consistently detected that redundancy was higher than synergy. In datasets 2 and 3 (Figures 6B and 6C), using uncorrected estimates would have led to a very major overestimation of the real importance of synergy, and in dataset 3 it would have led to the wrong conclusions that synergy was higher than redundancy. Importantly and reassuringly, estimates of both redundancy and synergy were highly consistent across methods within each dataset (Figures 6A–6C), suggesting that these estimates on real data are precise and unbiased. Moreover, the fact that the synergy obtained with the conservative shuffle subtraction is positive proves that there is genuine synergy in all datasets that cannot be due to finite sampling artifacts. Further, when using bias corrections (but not when using uncorrected estimates), results are almost identical when running the analysis on half of the datasets (Figure S16), further confirming the robustness of the bias corrections.

To test our methods on non-spiking, continuous-valued measures of neural activity, we next consider dataset 4 (Figure 6D), consisting of fMRI signals collected from *n* = 100 human participants at rest.^85^^,^^86^^,^^87^^,^^88^^,^^89^ To evaluate whether different brain regions interact synergistically or redundantly, we compute the information about the fMRI signal value in a set of *d* regions of interest (ROIs) (PID target) found one imaging timeframe later in the fMRI signal of two *d*-dimensional sets of other (source) fMRI ROIs (Figure 6D). Established analyses of PID of information transfer across brain regions are often performed with Gaussian PID by considering regions as one-dimensional sources/targets (*d* = 1).^49^^,^^51^^,^^90^ As we show in Figure S17, these low-dimensional Gaussian estimates of redundant and synergistic interactions across brain regions have negligible bias. Here, we investigate whether it is possible to extend low-dimensional Gaussian PID analyses of synergistic and redundant interactions to high-dimensional brain regions using the Gaussian gPID with our bias corrections. We focus on information interactions measured across a number of samples (that is, time points) that falls within the range common in the fMRI literature (256 imaging frames^91^^,^^92^) and that can also be realistically collected in clinical settings. When considering large-dimensional sets of ROIs (*d* = 20), the bias of synergy estimation is a substantial fraction (≈35%) of the target value, but it was well corrected (as suggested by the fact that all our bias corrections give consistent results). Redundancy is much less biased (10% of the target value) and also well corrected for (Figure 6D). The Venkatesh correction fails to remove a large part of the synergy and redundancy bias, as predicted by the theoretical and simulation results (Figure S17). Results using the maximum available number of time points (1,189), showing a still significant but much lower bias, are reported in Figure S17. Altogether, this application suggests that our bias corrections are useful to extend the Gaussian estimation of synergistic and redundant interactions to distributed multidimensional brain networks, even with a limited number of samples at different time points, as is common in neuroimaging (especially for clinical applications).^91^^,^^92^

### The bias of three-source PID

While the two-source PID provides valuable insights into pairwise neural interactions, information encoding often involves higher-order interactions among larger ensembles of neural units.^90^^,^^93^^,^^94^^,^^95^^,^^96^ Therefore, developing PID with more than two sources applicable to empirical neural data is of importance because it can capture synergy and redundancy beyond second order.

We thus analyze the bias of the PID with three sources ($\vec{R}=\left(R_{1},R_{2},R_{3}\right)$) and one target (*S*). Although neither the discrete BROJA PID^33^ nor its Gaussian counterpart gPID^30^ have been defined for more than two sources as yet, other PIDs such as *I*_*min*_ and *I*_*MMI*_ have been defined for, and can be readily applied to, more than two sources. Here, we study the bias properties of the MMI with three sources. We focus on two components with straightforward interpretation. The first is three-way redundancy, which quantifies the information that each of the sources *R*_1_, *R*_2_, and *R*_3_ encode about *S* (defined in the MMI PID as the minimum among the single-source information that each source encodes about the target). The second is three-way synergy, which quantifies the information about *S* that is available only from the joint observation of (*R*_1_, *R*_2_, *R*_3_) and not from any individual source or pair of sources (defined in the MMI PID as the joint information that the three sources encode about the target minus the maximum information carried by any pair of sources^97^^,^^98^).

**Figure 7:**
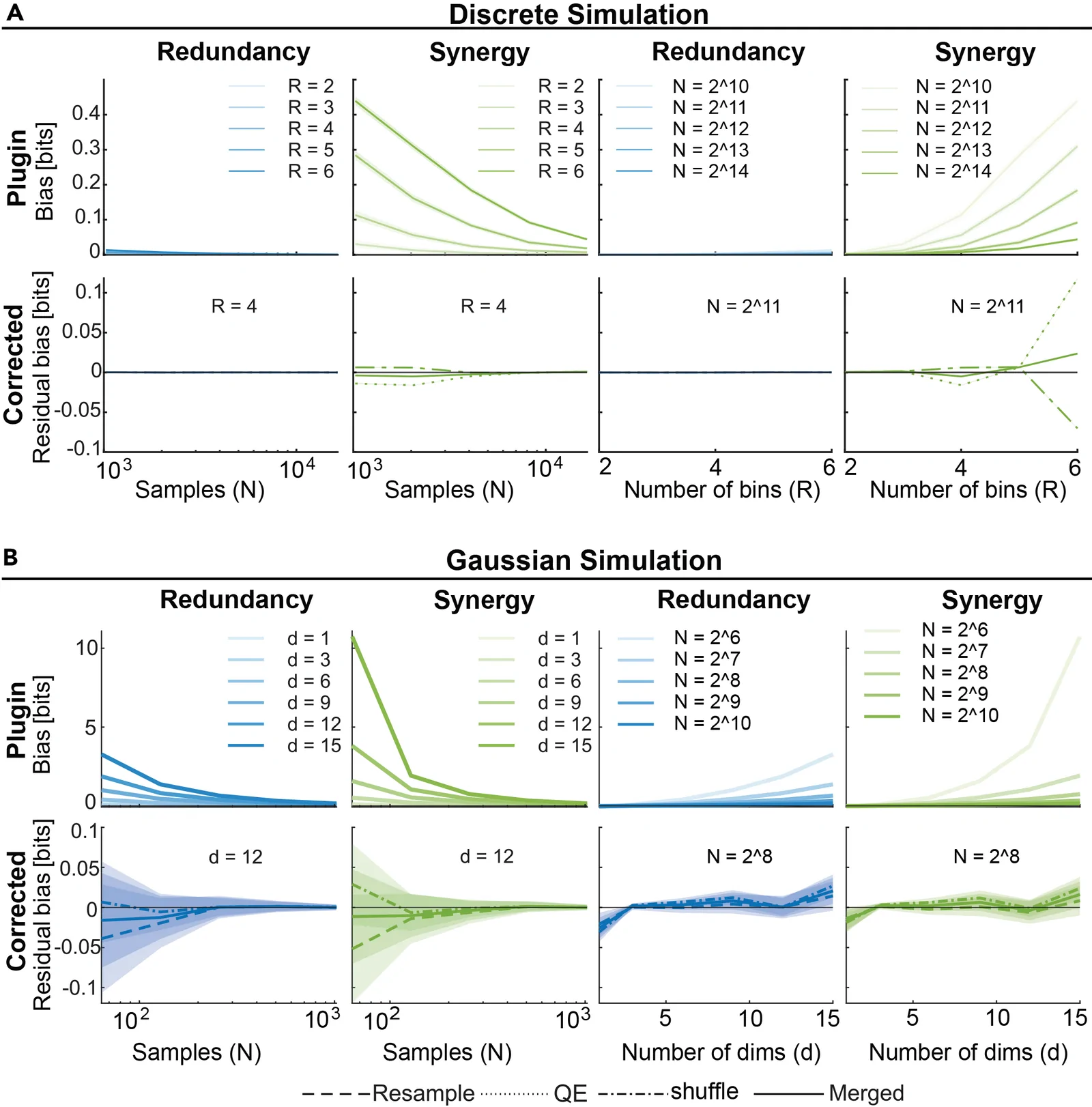
Simulations of the bias of the three-source PID Plots show on the top row the bias (averaged empirical estimates from *N* samples minus ground-truth values) of the plugin (i.e., not bias-corrected) estimate and on the bottom row the residual bias (averaged bias-corrected empirical estimates minus ground truth) of the bias-corrected three-way synergy and three-way redundancy as a function of number of samples or number of parameters defining the probability distributions. (A) Discrete MMI PID simulations. Top: the bias of plugin information as a function of the number of samples *N* (left, on a log *x* axis; each line corresponds to a fixed number of bins *R*) or the number of bins *R* (right; each line corresponds to a fixed *N*) used to compute PID. Bottom: the residual bias of bias-corrected PID for different correction methods (QE, shuffle subtraction, and merged) as a function of the number of samples *N* (left, *R* = 4 on a log *x* axis) or the number of bins *R* (right, *N* = 2,048). In all simulations in this panel, we set *S* = *R*. (B) Gaussian MMI PID simulations. Top: same as top row of (A), but considering the dimension of sources/target *d* instead of *R*. Bottom: same as row of (A), but showing the residual bias for resample, shuffle-subtraction, and merged corrections. *d* = 12 when plotting the residual bias vs. *N* (left, on a log *x* axis); *N* = 256 when plotting the residual bias vs. *d* (right). We plot mean ± 3 SEM over 100 simulations. Details are provided in methods and Table S1.

As we did above for the two-source PID, we studied numerically the bias of both the discrete and Gaussian three-source MMI PID using simulations of discrete and Gaussian processes, respectively, whose ground-truth values of joint information and of three-way synergy and redundancy can be computed from the exact probabilities used to simulate the data. Namely, for the discrete case we simulated a 3-bit parity system with purely ground-truth synergistic information, and for the Gaussian case we simulated a system with a ground-truth covariance matrix having only redundancy (see methods). Again, exactly as for the above two-source simulations, in this case also we computed the bias as the difference between the plugin estimate empirically obtained with a certain number of trials and the corresponding ground-truth value. We find that on simulated data, the bias of the three-way synergy is far larger than the bias of the three-way redundancy, with a larger gap than that found for the synergy and redundancy obtained with the two-source PID (Figure 7) for both discrete and Gaussian PID. This is because (see methods) the bias of the most biased terms of the three-way synergy scales like the bias of the joint information carried by three sources (which scales as (*S* × *R*^3^)/*N* for the discrete PID and as a supralinear function of 4*d* terms for the Gaussian PID), whereas the three-way redundancy has terms with bias scaling as the bias of the single-source information (which scales as (*S* × *R*)/*N* for the discrete PID and as a sum of digamma functions with up to 2*d* terms for the Gaussian PID). We tested the effectiveness on this three-source PID of the various bias corrections that we developed above (Figure 7, lower rows). The bias-correction procedures appear to be highly effective also for the three-source PID.

We evaluated the bias corrections for the three-source PID on an empirical MEG dataset^77^ (dataset 5) collected from human participants performing a decision-making task requiring them to estimate, from several successive samples of a visual stimulus, the generative mean of the angle specifying the position of the visual stimulus. We computed the information encoded by broadband MEG activity recorded across multiple spatial locations (the sources) about the angle of the first sample shown (the target). We thus considered visual stimulus encoding in neural activity. An important property of this dataset is that both the sources (MEG activity) and the target (presented orientation of the visual stimulus) follow approximately a Gaussian distribution (see methods), and thus both the discrete and the Gaussian PID can be meaningfully computed. Between *N* = 519 and *N* = 864 trials were available for each of *n* = 30 human participants, and one sample of neural activity per trial was collected.

**Figure 8:**
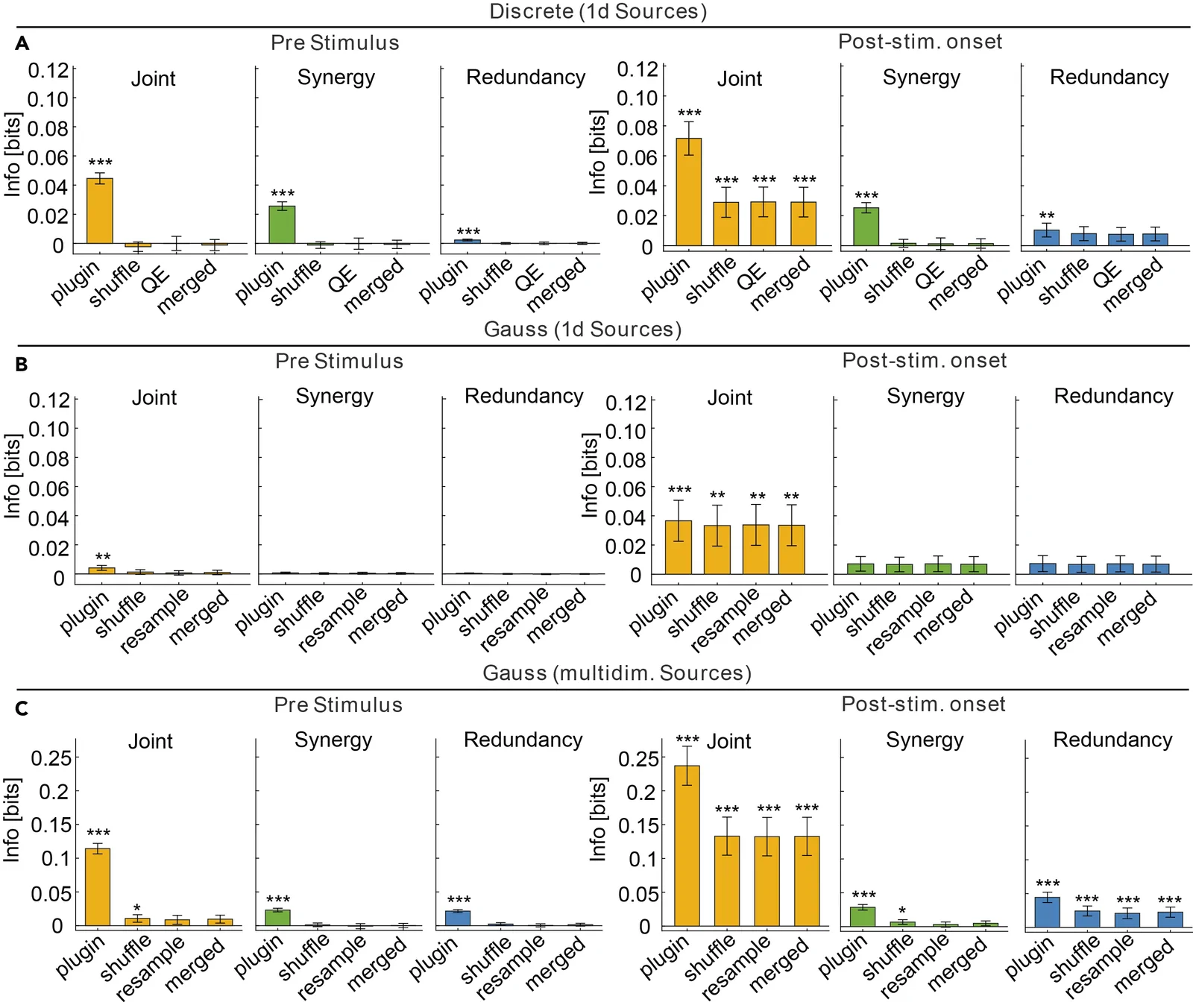
Application of PID bias corrections to an empirical MEG dataset (dataset 5) analyzed with the three-source PID Panels report the joint, redundant, and synergistic information that triplets of sources (MEG activity of one or multiple brain areas) carry about a target (the angle specifying the position of the first visual sample presented at the beginning of each trial). (A) Discrete MMI PID. The three sources are the three visual areas V1, V2, and V3. Bias-correction methods: plugin (no correction), shuffle subtraction, QE, and merged. (B) Gaussian MMI PID with one-dimensional sources. The three sources are the three visual areas V1, V2, and V3. Bias-correction methods: plugin (no correction), shuffle subtraction, resample, and merged. (C) Gaussian MMI PID. The three large-scale, multivariate sources are the joint MEG activity of all areas within the visual cortex, the prefrontal cortex, or the somatosensory and motor cortex, respectively. Bias-correction methods: plugin (no correction), shuffle subtraction, resample, and merged. In all panels, we used MEG activity recorded either (left) during a pre-stimulus time window of length 150 ms preceding the stimulus onset or (right) in a 150-ms window post stimulus onset. Significance against zero was assessed with a one-sample *t* test across participants (∗*p* < 0.05, ∗∗*p* < 0.01, ∗∗∗*p* < 0.001). We plot mean ± 3 SEM over 30 participants. Details are provided in methods.

We first considered the application of the discrete PID to MEG. Because the discrete PID does not scale easily to sources with many dimensions, we used it to address a question about encoding within a single anatomical group. Specifically, we studied the encoding of the visual stimulus orientation across three areas of the visual system (V1, V2, and V3). The activity of each region was a one-dimensional signal discretized into *R* = 3 equipopulated bins. The visual stimulus was also discretized into *S* = 3 bins, similarly to the original study.^77^ Since in this dataset MEG activity was recorded both before and after stimulus presentation, we used pre-stimulus MEG activity (for which the ground-truth value of joint information about the stimulus, and its synergy and redundancy, must all be zero) as a first important test of the bias-correction performance. With the plugin (uncorrected) estimator (Figure 8A, left), both joint information and synergy values were relatively large, indicating a significant bias. Application of our bias corrections successfully removed the spurious pre-stimulus information values, and all corrections gave consistent results. We then considered post-stimulus activity (Figure 8A, top right). The magnitude of the bias removed from post-stimulus estimates was comparable to, but smaller than, that observed in the pre-stimulus activity (compatible with the above theoretical predictions that the bias of synergy and joint information is larger for lower information values in the discrete case). All corrections gave consistent results, and the amount of bias subtracted by the corrections was a large fraction of the value of the resulting quantities after bias subtraction (146% and 1,669% for joint information and synergy, respectively). Conclusions based on plugin estimates would have led to the wrong conclusion that in this system both synergy and redundancy are present, with synergy larger than redundancy, whereas after bias corrections it becomes clear that both synergy and redundancy are small and neither reaches significance. Since in this dataset we could apply both discrete and Gaussian PID, we next applied the Gaussian MMI PID to the same set of one-dimensional sources (Figure 8B). The Gaussian PID in the one-dimensional sources had very small bias, as shown by the fact that the plugin (uncorrected) and bias-subtracted estimators have very similar values. Bias-corrected estimates were very similar between the discrete and Gaussian PID when using bias corrections, indicating that the bias corrections provide reliable and uninflated values for both the discrete and Gaussian PID.

We then considered the highly multivariate problem of how the simultaneous activity of many different brain areas across different anatomical groups jointly encodes information about the visual stimulus. This question cannot be addressed by the discrete PID (as it does not scale to very large multivariate analyses) and, to our knowledge, has not yet been empirically addressed in the literature. Given the small bias of the Gaussian PID on one-dimensional sources shown above and given the promising performance of the bias-correction estimators for the Gaussian three-source PID on the above simulated data, we addressed this multivariate coding problem with the Gaussian PID. Namely, we used the Gaussian MMI^34^ of joint information between the stimulus orientation (target) and the activity of three large-scale, multivariate sources (the joint activity of all 26 areas in the visual cortex, all 47 areas in the prefrontal cortex, and all 25 areas in the somatosensory and motor cortex). From pre-stimulus MEG activity with the plugin (uncorrected) estimator (Figure 8C, bottom left), both joint information and synergy and redundancy values were relatively large (approximately 0.1 and 0.03 bits, respectively), indicating a significant bias and proving that this question could not be addressed without bias-correction techniques even when making Gaussian approximations. Application of our bias corrections successfully removed the spurious pre-stimulus information values, and all corrections gave consistent results. From post-stimulus activity (Figure 8C, bottom right), the bias corrections removed from the plugin estimates biases that were similar in size to those identified in pre-stimulus activity (compatible with the above theoretical predictions). All corrections gave consistent results, and the amount of bias subtracted by the corrections was a large fraction of the value of the corresponding quantities after bias subtraction. After bias corrections, the PID reveals significant redundancy between the activity of the anatomical groups but no or little synergy.

Together, these results confirm that bias corrections are both necessary and highly effective for three-source PID and that their use allows formulating and addressing neuroscientific questions about multisource encoding that would be out of reach without bias corrections.

## Discussion

We investigated thoroughly, with analyses of simulated and real neuroscientific data and with analytical considerations, how limited-sampling bias affects empirical measures of synergy and redundancy across various types of PID. We found that synergy, and to a lesser extent redundancy, suffer from a considerable limited-sampling bias. Furthermore, we observed that the synergy bias (and to a lesser extent the redundancy bias) grows rapidly with the dimensionality of the problem under investigation (related to either the number of sources used or the number of dimensions used to describe each source) and that it is often large enough to be comparable to or larger than the target values of the quantities. Thus, the bias problem cannot be neglected in real-data neuroscience applications. If uncorrected, the bias problem can artificially lead to inaccurate estimates and skew comparisons in favor of synergy. Our work highlights and explains the presence of a major and previously neglected difference in the sampling bias between synergy and redundancy, which can inflate synergy disproportionately. Neural synergy has been widely reported in recent years and has led neuroscientists to rethink how the brain integrates information.^23^ Our results call for a careful evaluation of synergy in neuroscience using suitable bias-correction methods.

Our work is immediately relevant to empirical neuroscience. First, it empowers experimenters with bias-correction methods that greatly improve PID estimates with respect to uncorrected measures or other available methods.^30^ The most effective bias-correction method is what we termed the merged correction, which combines linearly two different bias-correction methods with opposite tendencies for residual errors. We thus recommend the application of the merged correction for practical applications. As confirmed by our extensive real-data analyses, these bias-correction methods are generally applicable to a wide range of experimental situations and types of brain recordings, crucially encompassing both discrete and continuous cases. As shown by our applications to real and simulated data, our bias-correction methods reduce by orders of magnitude the number of samples needed to reach a certain estimation precision or the magnitude of the residual errors in PID estimation from small numbers of samples.

The second direct empirical relevance of our work is that it informs the design of experiments. This is because it provides an understanding of the bias in terms of simple properties of experimental design (number of trials or number of time points that determine the sample size). The rule of thumb is that the information estimation is accurate (with a small tolerance value that can be easily estimated from the data) in the so-called sufficient-sampling regime. For discrete PID, the sufficient-sampling regime requires that the number of samples *N* satisfy *N* > 4 × *S* × *R*^2^, where *R* and *S* are the number of possible discrete values for the sources and target, respectively. For the Gaussian PID, the sufficient-sampling regime requires that the number of samples *N* satisfy *N* > 12*d*, where *d* is the dimension of the sources and the target.

Third, our work informs the choices to be made for the analysis setting. It shows that discrete rather than Gaussian PID should be used for spiking activity and/or for experiments with discrete stimuli set. It then informs (using the same rules of thumb set above) about the range of number of bins or the range of the number of dimensions that can be used given the collected number of samples.

Our simulations and analytical considerations were accompanied by a thorough validation of the performance of our bias-correction algorithms on five different neuroscientific datasets. This analysis confirms the effectiveness of the bias-correction procedures and demonstrates that they are essential for empirical studies. Bias-uncorrected estimates would lead to highly imprecise and possibly misleading conclusions about neural synergy. In most analyzed conditions, the majority (or at least a large part) of the synergy computed without bias corrections (plugin estimator) reflected just the limited-sampling bias and not real neural synergy. Thus, bias corrections should become a routine part of PID analyses of neuroscientific data, as they are now for traditional application of Shannon information theory to neural coding.^27^

Although the work presented here is primarily methodological, in testing our methods on empirical neuroscientific data we provide extensive knowledge about the distribution of synergy and redundancy in the brain. Most PID analyses have been applied to aggregate measures of neural activity such as fMRI. We extend this bulk of work by providing an extensive study of information encoding in real neural spiking activity (the fundamental level of brain information processing) across species and cortical areas. Our systematic analysis of the information carried by tens of thousands of pairs of neurons across different cortical areas revealed widespread presence of both synergy and redundancy across brain areas and species in neuron-to-neuron interactions, with regional variations in the prevalence of synergy vs. redundancy. Moreover, our analyses of real fMRI recordings with Gaussian PID confirm that previous reports of synergy in cross-area brain interactions are genuine and not due to biased estimates,^23^^,^^49^^,^^95^^,^^99^ and they show that bias corrections can improve the accuracy of interaction analyses with data sizes typical of clinical applications. Our work also reported an MEG-based multivariate analysis of sensory information coding across multiple brain regions in different anatomical groups, which reveals the presence of higher-order redundancy in stimulus representations within and across cortical groups.

Importantly, although we focused our applications on neuroscience data, our work could be readily applied more widely, as PID using the discrete or Gaussian estimators studied here are often employed in real-data analyses in non-neuroscience fields, such as the analysis of gene-regulatory networks, the selection of features in machine learning, the analysis of generative neural networks, or the analysis of financial data.^100^^,^^101^^,^^102^^,^^103^

Despite our thorough study of several types of PID and our extensive testing and validation with many simulated scenarios and multiple neural datasets, our work has limitations requiring further theory and simulations, including the following. First, we tested the bias of BROJA, *I*_*min*_, and *I*_*MMI*_ PIDs, which lend confidence to the generality of our results. However, it would be important to test others. For example, it would be important to test how our bias corrections and the prediction that synergy is more biased than redundancy hold across different PIDs including, for example, PIDs for continuous but non-Gaussian distributions.^44^ Because our bias corrections are derived without making assumptions on the underlying distributions, we hypothesize that they would be effective across PIDs including those not designed for Gaussian distributions. Because our analytical considerations predicting a higher bias for synergy than redundancy are based on the assumption that the synergy depends on the full joint distributions and the redundancy depends on the marginal but not the full joint distributions, we expect that these predictions hold for the several PIDs that respect this pairwise marginals property, such as those reported previously.^22^^,^^30^^,^^34^^,^^41^^,^^44^^,^^97^ To facilitate testing the wider applicability of our bias-correction methods, we provide open-source modular software implementation of these algorithms, which easily allows the testing of new PIDs. Second, the bias-correction procedures are heuristic, although we support them with analytical considerations. However, we did not provide theoretical guarantees of the sign of residual errors of different bias-correction procedures. Third, when considering spiking activity at the cellular level (which requires discrete PID), we tested synergy and redundancy between pairs and triplets of neural units, but we have not systematically tested large populations. The direct calculation of information is very precise for small populations, but it does not scale up with population size.^27^ However, our discrete methods could be scaled up by coupling them with dimensionality-reduction techniques that are often used in studies of neural population codes.^13^^,^^63^^,^^104^^,^^105^^,^^106^^,^^107^^,^^108^ We provide an initial analysis of discrete PID of population spiking activity in which each source consists of a population of ten simultaneously recorded neurons encoding stimulus information, and their high-dimensional activity is reduced with a support vector machine and then discretized (Note S7). Encouragingly, we found that our bias corrections provide accurate estimates of population redundancy and synergy even for small trial numbers (Note S7 and Figure S18). It remains to be explored how best to couple PIDs with dimensionality reduction to scale up analyses to large populations. Fourth, some methods proposed for mutual information^28^ and then extended to PID^31^ do not correct for the bias but introduce into the estimator terms designed to compensate for predicted differences in bias across different entropy terms. It would be useful to study in further work whether and how bias-correction and compensatory procedures could be combined to optimize PID estimates from limited samples.

In conclusion, our systematic analysis of the limited-sampling bias of PID analyses reveals that synergy is far more biased than redundancy, that in practical applications the bias may be as large as the target information values, and that this problem can artificially skew comparisons of synergy and redundancy in the brain. We also developed a robust method for correcting for the bias, which significantly reduces the number of samples needed for accurate estimates. This extends the applicability of PID to, for example, clinical rather than laboratory settings. These bias corrections also greatly extend the dimensionality of the problems that can be accurately investigated empirically, allowing, for example, highly multivariate analyses of synergy and redundancy across many brain regions. Because of the aforementioned reasons, our work suggests that correcting for the limited-sampling bias should become standard practice in the analysis of empirical data with PID.

## Methods

### Definitions of Shannon information and PID

We first provide definitions of Shannon information quantities, including entropy and mutual information, and describe relationships to compute redundant and unique information as linear combinations of Shannon quantities and synergy. We then introduce different PID measures that have been proposed and that are relevant for this paper for both the discrete and the Gaussian PID.

#### Shannon information and relationships with PID components

Here, to make the paper more self-consistent we provide a more extensive summary of definitions of Shannon information quantities relevant for this study, and we describe relationships to compute all PID components as linear combinations of Shannon quantities and the synergy *Syn*(*S*:*R*_1_;*R*_2_) (defined in Equation 7 for the BROJA measure).

The foundational quantity in Shannon information theory is entropy, which quantifies the amount of uncertainty associated with a probability distribution *p*(*X*_1_, …,*X*_*M*_), where each *X*_*i*_ is a possibly multivariate stochastic variable of dimension *d*_*i*_ (e.g., the activity of one or several neural units). $D=\sum_{i=1}^{M}d_{i}$ denotes the total dimensions of the system. The entropy of a discrete distribution *p*(*X*_1_, …,*X*_*M*_) is defined as^38^^,^^39^

(Equation 8) $$H\left(X_{1},\ldots ,X_{M}\right)=-\sum_{x_{1}\in X_{1},\ldots ,x_{M}\in X_{M}}p\left(x_{1},\ldots ,x_{M}\right)\log_{2}\left(p\left(x_{1},\ldots ,x_{M}\right)\right)\text{,}$$

where the sum spans all possible combinations of values (*X*_1_ = *x*_1_, …,*X*_*M*_ = *x*_*M*_) in the *D*-dimensional probability space. The base-2 logarithm in Equation 8 implies that entropy is measured in bits. (Note that the base of the logarithm merely acts as a multiplicative term independent of the sampled probabilities. In case of a base change, both the actual information value and the value of the information bias will be rescaled by the same multiplicative factor expressing the base change).

In the case in which full probability distributions take the form of (or are approximated by) a continuous Gaussian distribution *p*_*G*_ with covariance matrix Σ(*X*_1_, …,*X*_*M*_) of dimensions *D* × *D* (capturing the second-order statistics of the original distribution), the sum in Equation 8 becomes a *D*-dimensional integral over continuous variables, with a closed-form solution^39^:

(Equation 9) $$H\left(X_{1},\ldots ,X_{M}\right)=-\int_{x_{1},\ldots ,x_{M}}p_{G}\left(x_{1},\ldots ,x_{M}\right)\log_{2}\left(p_{G}\left(x_{1},\ldots ,x_{M}\right)\right)=\frac{1}{2}\log_{2}\left({\left(2\pi e\right)}^{D}\;\det\left(\Sigma \left(X_{1},\ldots ,X_{M}\right)\right)\right)\text{,}$$

where each variable *X*_*i*_ is integrated over its *d*_*i*_-dimensional support. All the information theoretical quantities defined in the paper as sums and differences of entropy terms can be equivalently expressed in the discrete case by substituting entropy terms with the definition provided in Equation 8 or in the Gaussian case by using the definition in Equation 9.

In this paper, we consider probability distributions of up to *M* = 4 variables, where *S* denotes the target variable of the PID (it could be, e.g., a feature of a sensory stimulus or the activity of a neural unit), and *R*_*i*_ (*i* = 1,2,3) are the sources of the PID (e.g., activity of a neural unit). In this section, we focus on PID with two source variables, which is the case predominantly considered throughout the paper. (We cover definitions of PID with three source variables in a later dedicated section.) Mutual information carried by a source *R*_*i*_ about the target *S* quantifies the average reduction in the entropy of the target *S* upon a single-trial observation of the value of the source *R*_*i*_ and can be expressed as sums and differences of entropy terms:

(Equation 10) $$\begin{array}{l} I\left(S;R_{i}\right)=-H\left(S,R_{i}\right)+H\left(R_{i}\right)+H\left(S\right) \end{array}\text{.}$$

Importantly, *I*(*S*;*R*_*i*_) quantifies the full statistical relationship between *S* and *R*_*i*_ at the single-trial level, capturing the effect of both linear and non-linear interactions. Equation 1 in the main text follows directly from Equation 10 by substituting the entropy terms with their definitions as in Equation 8. Similarly, the mutual information about the target *S* carried by the joint observation of the two sources (*R*_1_, *R*_2_) is defined as

(Equation 11) $$I\left(S;R_{1},R_{2}\right)=-H\left(S,R_{1},R_{2}\right)+H\left(R_{1},R_{2}\right)+H\left(S\right)\text{.}$$

As described in the main text, two-sources PID breaks down the joint mutual information that two sources *R*_1_,*R*_2_ carry about the target variable *S* into the sum of four non-negative components satisfying the constraints in Equations 3, 4, and 5. Once a definition of any of the four PID components is provided, the other three can be obtained as linear combinations between Shannon information quantities and the defined PID component. To facilitate implementation by users, in the following we report the equations to compute the redundancy *Red*(*S*:*R*_1_;*R*_2_) and the unique information components *Unq*(*S*:*R*_1_\*R*_2_) and *Unq*(*S*:*R*_2_\*R*_1_) as the linear combination of Shannon information quantities and the synergy *Syn*(*S*:*R*_1_;*R*_2_) (for which we provided a definition in Equation 7).

By subtracting Equation 4 from Equation 3, we obtain

(Equation 12) $$I\left(S;R_{1},R_{2}\right)-I\left(S;R_{1}\right)=I\left(S;R_{2}|R_{1}\right)=Unq\left(S:R_{2}\setminus R_{1}\right)+Syn\left(S:R_{1};R_{2}\right)\text{,}$$

where *I*(*S*:*R*_2_|*R*_1_) is the conditional mutual information about *S* provided by *R*_2_ given *R*_1_, defined as the joint information carried by *R*_1_ and *R*_2_ minus the information carried individually by *R*_1_.^39^ Analogously, by subtracting Equation 5 from Equation 3, we obtain

(Equation 13) $$I\left(S;R_{1},R_{2}\right)-I\left(S;R_{2}\right)=I\left(S;R_{1}|R_{2}\right)=Unq\left(S:R_{1}\setminus R_{2}\right)+Syn\left(S:R_{1};R_{2}\right)\text{.}$$

Finally, by subtracting Equations 4 and 5 from Equation 3, we obtain

(Equation 14) $$I\left(S;R_{1},R_{2}\right)-I\left(S;R_{1}\right)-I\left(S;R_{2}\right)=Syn\left(S:R_{1};R_{2}\right)-Red\left(S:R_{1};R_{2}\right)\text{,}$$

where the quantity on the left-hand side of Equation 14 is known as the co-information *CoI*(*S*;*R*_1_;*R*_2_) of *S*, *R*_1_, and *R*_2_:

(Equation 15) $$CoI\left(S;R_{1};R_{2}\right)=I\left(S;R_{1},R_{2}\right)-I\left(S;R_{1}\right)-I\left(S;R_{2}\right)\text{.}$$

Linear relationships between the four PID components and Shannon information quantities are depicted in Figure S1. While Equations 12, 13, and 14 do not impose any independent constraint on the four PID components additionally to the ones in Equations 3, 4, and 5, they allow to express *Red*(*S*:*R*_1_;*R*_2_), *Unq*(*S*:*R*_1_\*R*_2_), and *Unq*(*S*:*R*_2_\*R*_1_) as combinations of Shannon information quantities and of *Syn*(*S*:*R*_1_;*R*_2_), as follows:

(Equation 16) $$\begin{array}{l} Red\left(S:R_{1};R_{2}\right)=Syn\left(S:R_{1};R_{2}\right)-I\left(S;R_{1},R_{2}\right)+I\left(S;R_{1}\right)+I\left(S;R_{2}\right)\\ Unq\left(S:R_{1}\setminus R_{2}\right)=I\left(S;R_{1}|R_{2}\right)-Syn\left(S:R_{1};R_{2}\right)\\ Unq\left(S:R_{2}\setminus R_{1}\right)=I\left(S;R_{2}|R_{1}\right)-Syn\left(S:R_{1};R_{2}\right) \end{array}\text{.}$$

Expressions similar to the ones of Equation 16 can be easily obtained to write any three of the PID components as an explicit function of the fourth one. For example, we could have written *Unq*(*S*:*R*_1_\*R*_2_), *Unq*(*S*:*R*_2_\*R*_1_), and *Syn*(*S*:*R*_1_;*R*_2_) as functions of *Red*(*S*:*R*_1_;*R*_2_).

### BROJA discrete PID

We provide a mathematical definition of the main measure of PID components used in this paper for discrete probability distributions with two source variables, the so-called BROJA measure.^33^ The mathematical definitions of the other two discrete PID measures that we use in this paper, *I*_*min*_ and *I*_*MMI*_, are provided in Note S1.

We first briefly remind ourselves that for the discrete case, the single-source and joint-source information about the target can be written as a function of the probability distribution *p*(*s*,*r*_1_,*r*_2_) and its marginals:

(Equation 17) $$\begin{array}{lll} I\left(S;R_{i}\right) & = & \sum_{S,R_{i}}p\left(s,r_{i}\right)\log_{2}\left(p\left(s,r_{i}\right)\right)-\sum_{R_{i}}p\left(r_{i}\right)\log_{2}\left(p\left(r_{i}\right)\right)\\ & & -\sum_{S}p\left(s\right)\log_{2}(p\left(s\right))\text{,} \end{array}$$

(Equation 18) $$\begin{array}{ccc} I\left(S;R_{1},R_{2}\right) & = & \sum_{S,R_{1},R_{2}}p\left(s,r_{1},r_{2}\right)\log_{2}\left(p\left(s,r_{1},r_{2}\right)\right)-\sum_{R_{1},R_{2}}p\left(r_{1},r_{2}\right)\log_{2}\left(p\left(r_{1},r_{2}\right)\right)\\ & & -\sum_{S}p\left(s\right)\log_{2}\left(p\left(s\right)\right) \end{array}\text{.}$$

The BROJA measure^33^ is defined via an optimization problem, minimizing the joint mutual information *I*(*S*;*R*_1_,*R*_2_) (which depends on the trivariate probability distribution *p*(*s*, *r*_1_, *r*_2_)) in the probability space Δ_*P*_ of distributions *q*(*s*,*r*_1_,*r*_2_) having pairwise marginals between each source and the target equal to the original ones, i.e., *q*(*s*,*r*_1_) = *p*(*s*,*r*_1_) and *q*(*s*,*r*_2_) = *p*(*s*,*r*_2_). The rationale of this approach is that the synergy can be conceptualized as the information about the target in the joint space of the two sources that cannot be possibly recovered by observing one source at a time (thus from the marginal probabilities), thus it can be defined operationally as the difference between the original joint information and the minimal joint information about the target that can be found in the space Δ_*P*_ of distributions that preserve the marginals (which is the union information, Equation 6):

(Equation 19) $$Syn\left(S:R_{1};R_{2}\right)=I\left(S;R_{1},R_{2}\right)-\min_{q\in \Delta_{P}}I_{q}\left(S;R_{1},R_{2}\right)\text{.}$$

Redundancy and unique information components can then be computed from the synergy and the Shannon information values using the linear relationships provided above (see Equation 16). Among the advantages of the BROJA PID, we note that this definition ensures that all PID terms satisfy the additivity property (i.e., when both the sources and the target are composed of independent subsystems, the PID components computed for the full system equal the sum of the corresponding components computed separately for each subsystem). However, while *I*_*MMI*_ and *I*_*min*_ have been defined for an arbitrary number of source variables,^22^^,^^97^ the BROJA PID is currently defined only for the case of two source variables.

### Gaussian PID measures

For the Gaussian case, the single-source and joint information about the target can be written in terms of the covariance matrices as follows^39^:

(Equation 20) $$I\left(S;R_{i}\right)=\frac{1}{2}\log_{2}\left(\frac{\det\left(\Sigma \left(S\right)\right)\det\left(\Sigma \left(R_{i}\right)\right)}{\det\left(\Sigma \left(S,R_{i}\right)\right)}\right)\text{,}$$

(Equation 21) $$I\left(S;R_{1},R_{2}\right)=\frac{1}{2}\log_{2}\left(\frac{\det\left(\Sigma \left(S\right)\right)\det\left(\Sigma \left(R_{1},R_{2}\right)\right)}{\det\left(\Sigma \left(S,R_{1},R_{2}\right)\right)}\right)\text{,}$$

where Σ( ) denotes the covariance matrix of the variables listed as arguments (e.g., Σ(*S*, *R*_1_, *R*_2_) is the covariance matrix of the joint multivariate Gaussian probability of observing in the same trial the combination of the target *s* and the two sources *r*_1_,*r*_2_).

In principle, any PID that has redundancy *Red*(*S*:*R*_1_,*R*_2_) and unique information components *Unq*(*S*:*R*_1_\*R*_2_) and *Unq*(*S*:*R*_2_\*R*_1_) depending only on the marginal distributions between the sources and the target (the so-called pairwise marginal property, see Note S2) reduces to *I*_*MMI*_ when computed from jointly Gaussian distributions *p*_*G*_ and the target *S* is one-dimensional.^34^ For the Gaussian MMI, closed-form solutions for PID exist, since the mutual information terms appearing in Equation 3 can be expressed in terms of covariance matrices as in Equation 20. To our knowledge, no general, closed-form expression exists for Gaussian PIDs with a multi-dimensional target, and such systems can yield results that deviate from *I*_*MMI*_.

One PID definition developed explicitly for Gaussian variables is the gPID measure,^30^ which defines the union information via constrained optimization as

(Equation 22) $$Union\left(S:R_{1};R_{2}\right)=\min_{q\in \Delta_{P_{G}}}I_{q}\left(S;R_{1},R_{2}\right)\text{,}$$

where $\Delta_{P_{G}}$ is the set of all jointly Gaussian probability distributions *q*(*s*,*r*_1_,*r*_2_) that have the same pairwise marginals *q*(*s*,*r*_1_) = *p*(*s*,*r*_1_) and *q*(*s*,*r*_2_) = *p*(*s*,*r*_2_) as the original distribution *p*(*s*, *r*_1_, *r*_2_), and *I*_*q*_(*S*;*R*_1_,*R*_2_) is the joint information computed for distribution *q*(*s*;*r*_1_,*r*_2_).

This definition corresponds to the BROJA PID one but restricts the domain of the optimization problem to the space of jointly Gaussian distributions. Given a Gaussian probability distribution with a covariance matrix Σ(*R*_1_,*R*_2_,*S*), the optimization problem in Equation 22 can be expressed in terms of cross- and auto-covariance matrices derived from Σ(*R*_1_, *R*_2_, *S*)^30^:

(Equation 23) $$Union\left(S:R_{1};R_{2}\right)=\frac{{\min_{\Sigma \left(R_{1},R_{2}|S\right)}}^{1}}{2\;\ln\;2}\ln\left[\det\left(I+\Sigma {\left(S\right)}^{-1}\Sigma {\left(R_{1}R_{2},S\right)}^{T}\Sigma \left(R_{1},R_{2}|S\right)\Sigma \left(R_{1}R_{2},S\right)\right)\right]\text{,}$$

where Σ(*R*_1_*R*_2_, *S*) denotes the cross-covariance matrix between the joint neuronal responses (*R*_1_, *R*_2_) and the target *S*, Σ(*R*_1_, *R*_2_|*S*) the conditional cross-covariance between *R*_1_ and *R*_2_ given *S*, and Σ(*S*) the auto-covariance matrix of *S*.

Key properties of PID components for two source variables are reported in Note S2.

### Details of implementation of the bias-correction procedures

#### QE

The QE procedure^60^ assumes we are in the asymptotic sampling regime *N* × *p*(*s*,*r*_1_,*r*_2_) ≫ 1 for all *s*,*r*_1_,*r*_2_, so that the bias of the entropies, information, and PID terms can be accurately approximated as a second-order expansion in 1/*N*. The estimation of the coefficients of this expansion is done by recomputing the information from fractions (*N*/2 and *N*/4) of the data available and then fitting (using a least-squares-error procedure) the plugin (uncorrected) information values obtained with fractions of data to the preceding quadratic function of 1/*N*. From this second-order equation, the value of information estimated for *N* = ∞ is taken as the unbiased information estimate. In our numerical implementation of QE, we allowed for a user-selected number *K* of data partitions into halves and quarters. We performed a separate extrapolation for each partition and then averaged the resulting estimates of the unbiased information value in each extrapolation to obtain our final estimate of the unbiased information. To ensure that the bias-corrected PID terms respect exactly the linear constraints of PID theory (Equations 3, 4, and 5), we computed the bias correction for the synergy, the joint, and the single-source information and then computed the bias-corrected unique and redundant information from the linear constraints (Equation 16). We used *K* = 20 partitions in the simulations in the main text and the supplemental information as well as in the analyses of real data. We applied QE only to the discrete PID because the bias of the Gaussian PID was clearly not polynomial (see Note S6).

#### Shuffle-subtraction bias correction

This procedure was applied in the same way to discrete and Gaussian PID. To estimate the bias, we computed PID after randomly permuting the target values across trials. In this way, all information about the target is lost and all PID terms should be zero but for the finite sampling effect. Thus, the resulting PID terms can be taken as an estimation of their bias, which we subtracted from their plugin estimates. In our implementation, we allowed the user to perform a number *V* of random permutations, and we then took as a measure of the bias (to be subtracted from the plugin estimate to obtain the unbiased estimates) the average of the PID term computed over all random realizations of the permutation. To ensure that the bias-corrected PID terms respect exactly the linear constraints of PID theory (Equations 3, 4, and 5), we computed the bias correction for the synergy, the joint, and the single-source information and computed the bias-corrected unique and redundant information from the linear constraints (Equation 16). We used *V* = 20 in the simulations in the main text and the supplemental information as well as in the analyses of real data.

#### Resampling bias correction

The resampling method for bias correction is grounded on the assumption that information measures from similar distributions suffer from similar biases. From the available dataset with *N* samples, we first compute an estimated probability matrix (for the discrete case) or an estimated covariance matrix (for the Gaussian case). We then generate *N* samples from this estimated distribution, and we compute the bias as the difference between the information obtained from the *N* samples generated by the estimate distribution and the information obtained by inserting the estimated distribution into the information or PID equations. This process is repeated *W* times, so the bias is estimated as the average across many surrogate datasets. Finally, we correct the original information values by subtracting the estimated bias. To ensure that the bias-corrected PID terms respect exactly the linear constraints of PID theory (Equations 3, 4, and 5), we computed the bias correction for the synergy, joint, and single-source information and then computed the bias-corrected unique and redundant information from the linear constraints (Equation 16). We used *W* = 20 resampling iterations in the simulations in the main text and the supplemental information as well as in the analyses of real data. Given that, in the discrete scenario, the resampling bias correction exhibits the same sign of residual bias as QE but consistently poorer performance (Figures S5–S7), we did not include this correction in the discrete analyses presented in the main text.

#### Merged bias correction

Because the shuffle subtraction and the QE (for discrete) or resampling (for Gaussian) tend to have residual errors of opposite sign (see Figures 4, 5, and S3–S10), we introduced a merged bias correction that estimates the bias as the weighted average of the two above-described procedures:

(Equation 24) $$Bias_{\text{merged}}=w_{\text{QE}}\cdot Bias_{\text{QE}}+\left(1-w_{\text{QE}}\right)\cdot Bias_{\text{shuff}}\text{,}$$

where *Bias*_QE_ and *Bias*_shuff_ are the bias estimates from the QE procedure (for the discrete case) or the resampling correction (for Gaussian) and shuffle-subtraction procedures, respectively, *w*_QE_ ∈ [0,1] is the weight assigned to the QE (for discrete PID) or resampling (for Gaussian) correction, and 1 − *w*_QE_ is the weight assigned to the shuffle subtraction. For the discrete two-source PID, *w*_QE_ was set using a heuristically optimized, information-level-dependent weighting. Since shuffle subtraction performs considerably better than QE at low information levels, and the opposite holds at higher information levels, for each simulated data point from all four discrete simulation scenarios shown in Figure S5, we computed

(Equation 25) $$w_{\text{QE}}=\frac{R_{\text{shuff}}}{R_{\text{QE}}+R_{\text{shuff}}}\text{,}$$

where *R*_QE_ and *R*_shuff_ denote the absolute value of the residual biases after QE and shuffle correction, respectively. By construction, *w*_QE_ assigns a higher weight to the correction that has the smaller residual error.

Since Equation 25 depends on the residual biases *R*_QE_ and *R*_shuff_, which require knowledge of the ground-truth information values to be computed and are thus unavailable in practice, we modeled the dependence of *w*_QE_ on the normalized information level (expressed as the joint information value normalized to the value of the entropy of the target variable, thus a number between 0 and 1) by fitting a sigmoid function of the normalized information level to the values of *w*_QE_ and their corresponding information levels pooled across all four simulation scenarios. These fits were performed separately for each combination of *N* and *R*, resulting in a set of sigmoid functions that return the weight *w*_QE_ to be applied given the observed information level and the experimental parameters *N* and *R*. We verified (Figure S12) that this optimized weighted merged procedure improved slightly a simpler version with equal weighting of the shuffle and QE.

For the Gaussian case, we always used an equally weighted combination of the shuffle and resampling bias-correction methods (*w*_QE_ = 0.5), because the residual biases of the shuffle-subtraction and resampling corrections show no differences in their dependence on the information level; thus, heuristically optimizing the weight as function of the information level would not have produced a performance increase.

For the three-source PID analyses (Figures 7 and 8), we used an equal weighting between the shuffle and the QE correction because in the range of simulations we explored, we did not have major shifts in information levels.

For the Gaussian case, since the residual biases of the shuffle-subtraction and resampling corrections show no differences in their dependence on the information level, we used an equally weighted combination of the shuffle and resampling bias-correction methods.

To ensure that the bias-corrected PID terms respect exactly the linear constraints of PID theory (Equations 3, 4, and 5), we computed the bias correction for the synergy, joint, and single-source information and computed the bias-corrected unique and redundant information from the linear constraints (Equation 16). We used *V* = 20 and *K* = 20 in the simulations in the main text and supplemental information as well as in the analyses of real data.

To provide users with an estimate of the magnitude of the expected residual error after applying the merged correction while in the sufficient-sampling regime, we fitted (using the data from simulations in Figures S3A, S4, and S5) the residual bias after applying the merged correction to a second-order polynomial of the parameter ratio that determines the leading-order scaling of the joint information and the synergy bias, namely the ratio between the total number of parameters defining the joint probability distribution (*R*^3^ in the discrete case and 3*d* in the Gaussian case) and the number of samples *N* (see Note S6). The resulting second-order equations (plotted in Figure S15) allow users to easily obtain an estimation of the magnitude of the residual bias of both synergy and redundancy for the discrete and Gaussian PID as a function of *N*, *R*, and *d*. This helps in designing the experiment and analysis that would allow estimation results to comply with a desired tolerance level.

#### Venkatesh et al. procedure for Gaussian PID bias correction

For the Gaussian PID only, we implemented the bias-correction procedures developed by Venkatesh and colleagues.^30^ Applying this procedure to the discrete PID would have been not possible because it assumes an analytical knowledge of the bias, which is only available for the Gaussian case.

The Venkatesh bias-correction procedure^30^ focuses first on the bias of the union information. The Venkatesh procedure corrects for the bias by first computing the bias-corrected joint information (see Equations 17, 106, and 107 of Venkatesh et al.^30^) with the Cai et al.^80^ equations of the entropy bias (Equation S17), then making the assumptions that the union information has the same proportional bias as the joint information, thus rescaling the bias of the union information accordingly (Equation 17 of Venkatesh et al.^30^). Note that, following Venkatesh et al.,^30^ if a Shannon information quantity had a negative value after the bias-correction procedure, it was set to zero. Then, to make sure all PID quantities after bias correction are non-negative and satisfy the PID linear constraints, the Venkatesh procedure applies a double post hoc rectification of the bias-corrected value of the union information as detailed in Equations 106 and 107 of Venkatesh et al.^30^ The resulting double-rectified union is the value taken by Venkatesh et al. as the bias-corrected union information.

From this, the Venkatesh procedure computes the four bias-corrected PID terms using the bias-corrected union information and the bias-corrected joint and individual information using the four linear PID constraints of Equations 3, 4, 5, and 7.

Our results show that, by assuming that the bias of the union information is proportional to the bias of the joint information, this approach can severely misattribute bias across PID components. In the Gaussian case, analytical considerations (see Note S6) indicate that the union information bias depends only on the dimensionality of the covariance matrices *d* and on the number of trials *N* and not on the magnitude of the union information itself (as implicitly assumed in Equation 17 of Venkatesh et al.^30^). Furthermore, the ratio between the biases of union and joint information is not fixed: it varies across sampling regimes, with joint information exhibiting disproportionately larger bias at low trial counts *N* or higher dimensionality of sources and target *d* (see Note S6). Because the Venkatesh procedure relies on these incorrect proportional-bias assumptions, it misassigns the bias among PID components, especially when all PID terms are non-zero and the true union information does not match any of the Shannon information quantities (so that the rectification steps in Equations 106 and 107 of Venkatesh et al.^30^ do not correct the error). Simulations corroborate these analytical predictions (Figures 4, 5, S3, and S8–S11): the Venkatesh correction typically underestimates the bias of synergy and redundancy.

### Details of the binning procedures used to compute the discrete PID

For all simulated data, we estimated the PID components based on the following binning approach. First, in each simulation, we generated a large number (*n* = 2,048) of trials per stimulus for each set of model parameters. We then discretized the probability distribution of spike counts for each neuron in the chosen number of bins. We computed the bin edges that made the partition of trials as equally populated as possible, following the procedure of Lorenz et al.^64^ These bin edges were then used for all simulations computing PID as a function of the number of trials per stimulus. We chose to fix the bin edges for each simulation to ensure that differences across trial numbers in the estimated values of the PID components were due only to trial numerosity and not to other reasons. This was important to study the properties of the information estimates as a function of the number of available trials. For real data, the binning procedure is specified in the supplemental information section describing their information analysis. The number of bins used to discretize single neural unit activity in each figure is reported in Table S1.

### Details of simulations used to test the bias properties

In this section, we report details of methods to generate the simulated tests presented in the paper. Parameters used for each figure are reported in Tables S1 and S2. The parameters used in individual figure panels were chosen with the criteria that these parameters were representative of wider trends, were within the range in our experience found in real-data analyses of neuroscience experiments, and illustrate well the point of interest. In all the simulations, we generated random values of a target variable (conceptualized as a sensory stimulus) and of two sources (conceptualized as the activity of different neural units). In all simulations, we collected one joint sample of target and sources values per trial; thus, the number of samples *N* used to compute the probabilities from simulated data equals the number of simulated trials.

#### Details of discrete simulations

To test the bias of the discrete PID algorithms, we simulated the activity of two neural units (without loss of generality, the spiking activity of two neurons) responding to a set of discrete stimuli.

In Figure 1 (zero-information case), we considered a simple scenario in which, on each simulated trial, the stimulus *S* and the spike count of each individual neuron *R*_1_ and *R*_2_ were drawn independently from a uniform probability distribution with four possible outcomes. Thus, the ground-truth value of joint information about the stimulus carried by the activity of two neurons (and, thus, synergy and redundancy) is zero.

To characterize bias scaling and the performance of bias-correction procedures in scenarios with non-zero joint information, we simulated processes with statistics reasonably close to neural firing while allowing us to control the strength of correlations between neurons as a function of the stimulus. This enabled systematic manipulation of the presence and magnitude of synergy across different stimulated scenarios.

We implemented a simple simulation based on Poisson responses (which are a reasonable approximation to neural firing^71^) that are either independent or shared across neurons. Specifically, the spike count *r*_1_ and *r*_2_ of each of the two simulated neurons for each of the stimulus values were the sum of two Poisson processes: one Poisson process that was independently drawn for each neuron capturing neuron-specific (“private”) variability of responses, and a second Poisson process that was shared between the two neurons, giving rise to noise correlations.

In all discrete PID simulations, apart from those in Figures 2A, 3A–B, 5A, and S3, we used a set of *S* = 4 simulated stimuli. This set of four simulated stimuli was made of two binary stimulus features *s*_1_ and *s*_2_, which varied across trials independently from each other. (This made it a 2-bit stimulus set, and the two independent binary stimulus features are coded as ⌊*s*/2⌋ and *s mod* 2 when spanning the stimulus id from 0 to 3 in the equations below). The equations for the mean rates (that is, mean spike counts) of each Poisson process follow for each simulation the following form:

(Equation 26) $$\begin{array}{l} rate_{shared}=\gamma \text{,}\\ rate_{individual-1}\left(s\right)=B+\alpha \beta s_{1}+\alpha \left(1-\beta \right)s_{2,}\\ rate_{individual-2}\left(s\right)=B+\alpha \left(1-\beta \right)s_{1}+\alpha \beta s_{2}\text{,}\\ s_{1}=\left\lfloor \frac{s}{2}\right\rfloor ,s_{2}=\left(s\;\mathrm{mod}\;2\right),s\in \left\{0,1,2,3\right\}\text{.} \end{array}$$

In the above mean rate equation used for our simulations of the spike counts of pairs of neurons in response to the stimulus, we have the following free parameters, which were varied across simulations. (1) A parameter *α* regulates the strength of stimulus tuning of the spike counts of the individual neurons or, in other words, the separation in response strength between least and most effective stimuli (higher values of *α* leading to higher values of individual neuron information and, thus, higher values of joint information); (2) a baseline parameter *B* expressing the overall baseline level of activity common to all stimuli (higher values of *B* in general decrease information of individual neurons and, thus, the joint information because they increase the standard deviation of responses at fixed stimulus and thus make the rate separation *α* between most and least effective stimuli smaller in standard deviation units); (3) a parameter *β*, which increases the amount of redundancy because it regulates the dissimilarity of tuning of the spike count of each individual neurons to the two stimulus features (*β* close to zero or one means independent tuning, i.e., each neuron encodes a different feature; higher values of *β* close to *β* = 0.5 indicate that both neurons encode the features similarly and redundantly, i.e., the two neurons encode the same linear combination of features); (4) a parameter *γ* regulating the strength of the shared process and, thus, the strength of correlated firing (increasing *γ* increases synergy because it makes the two neurons more strongly correlated—because the individual process changes its rate with different stimuli, having a shared process *γ* > 0 also affects the amount of synergistic information about the stimulus that can be gained by only measuring the joint responses of the two neurons because the fraction of shared spikes will change with the stimulus, a stimulus-dependent correlation case that is known to create synergy^20^).

These formulas allow us to construct analytically the ground-truth probability distribution for each scenario and information level. By using these probability distributions in the PID computation, we can use the resulting values to compute the limited-sample bias that we obtain when using PID on the probability distribution estimated from *N* samples.

To test the generality of our methods, we developed, by tuning the simulation parameters, several scenarios of interest (see Table S2 for full details) with different values of the individual PID component. A first scenario (“bit-of-all”) had all PID components with non-zero values (as it happened in all the four neuroscientific datasets we analyzed with two-source PID). A second scenario (“uncorrelated”) had negligible synergy and negligible redundancy (because the two neurons were uncorrelated, and each neuron encoded a different independently varying stimulus feature). A third scenario (“high redundancy”) had both neurons encoding the stimuli very similarly and thus had high redundancy but negligible synergy. A fourth scenario (“high synergy”), with strong and information-rich correlations between the neurons, had high synergy and negligible redundancy. Information about the scenario used for each discrete PID simulation is indicated in the respective figure captions.

For the simulations of different numbers of bins (Figures 2A, 3A–B, 5A, and S3), we needed to change the number of stimuli *S* together with the number of bins *R.* The coding of the variables indicating the two stimulus features *s*_1_ and *s*_2_ were modified as follows to make it easier to simulate a stimulus set with numerosity ranging from 2 to 9:

(Equation 27) $$\begin{array}{l} s_{1}=\left\lfloor \frac{s}{3}\right\rfloor ,s_{2}=\left(s\;\mathrm{mod}\;3\right),s\in \left\{0,..,S-1\right\} \end{array}\text{.}$$

Table S2 provides a summary of the parameters for all discrete simulation scenarios used in each figure.

#### Details of Gaussian simulations

To test the bias of the Gaussian PID algorithms, we simulated jointly Gaussian systems describing the activity of two neural units (without loss of generality, aggregate measures of neural activity recorded from two brain regions) responding to a Gaussian stimulus.

For the results shown in Figure 1C–D, we simulated jointly Gaussian variables *S*, *R*_1_, and *R*_2_, each of dimension *d* = 5, using a covariance matrix equal to the identity matrix of size 3*d*.

To simulate the different cases with non-zero components, we simulated jointly Gaussian variables *S*, *R*_1_, and *R*_2_, each of dimension *d*, using the “bit-of-all,” “both-unique,” “high-synergy,” and “zero-synergy” scenarios defined and used to test the gPID method in Venkatesh et al.^30^ (see section C.1, p.22 of their supplemental information). The both-unique case is designed to have no redundancy or synergy (Figure S11). The high-synergy case includes a significant amount of synergy (Figure S11). The zero-synergy case has no synergy component (Figure S11). The bit-of-all case combines features of the high-synergy and zero-synergy cases, ensuring that all PID components are non-zero. In all cases, the desired examples are obtained by setting appropriately the structure of the covariance matrix Σ(*S*,*R*_1_,*R*_2_) to achieve the desired effects, as explained in detail in Venkatesh et al.^30^ For all simulations, we used exactly the parameters reported in Venkatesh et al.^30^ for the corresponding case (results shown in Figure S11). Information about the scenario used for each Gaussian PID simulation is indicated in the respective figure captions. For the bit-of-all simulation, to study the sampling bias across different information levels, we scaled the diagonal of the covariance matrix by a factor *α*, thereby increasing the variance of each variable’s dimensions, effectively adding noise and reducing the mutual information between target and sources. *α* = 1 corresponds to choosing parameter values exactly equal to those reported in Venkatesh et al.^30^ *α* > 1 reduces the total mutual information. The *α* values used for each simulated scenario are reported in Table S2. The results of gPID applied to bit-of-all with various levels of *α* are reported in Figures S9 and S10.

In each simulated scenario, we generate *N* samples of multivariate Gaussian data to estimate the sample covariance matrix $\tilde{\Sigma }\left(S,R_{1},R_{2}\right)$. We then apply to the simulated data the gPID estimator^30^ which is a function of only the covariance matrix computed from the data (Equation 23). The bias of the PID components is computed as the difference between the average of the value of the component across all realizations of the simulations with *N* minus the ground-truth value of the component. The latter is computed numerically, giving as input to the gPID equations the true covariance matrix used to generate the data.

For simulations in Figure 3C, we computed the Gaussian PID of the conditionally independent joint information, *I*_*ind*_(*S*;*R*_1_,*R*_2_), built from *p*_*ind*_(*s*,*r*_1_,*r*_2_) (see Note S6 for further details). The distribution *p*_*ind*_(*s*, *r*_1_, *r*_2_) has a covariance matrix with the following form:

(Equation 28) $$\Sigma_{ind}\left(S,R_{1},R_{2}\right)=\left[\begin{array}{lll} \Sigma \left(S\right) & \Sigma \left(S,R_{1}\right) & \Sigma \left(S,R_{2}\right)\\ \Sigma \left(R_{1},S\right) & \Sigma \left(R_{1}\right) & \Sigma \left(R_{1},S\right)\Sigma {\left(S\right)}^{-1}\Sigma {\left(R_{2},S\right)}^{⊤}\\ \Sigma \left(R_{2},S\right) & \Sigma \left(R_{2},S\right)\Sigma {\left(S\right)}^{-1}\Sigma {\left(R_{1},S\right)}^{⊤} & \Sigma \left(R_{2}\right) \end{array}\right]\text{,}$$

where in deriving the above we used the following identities for jointly Gaussian variables:

(Equation 29) $$\begin{array}{l} \Sigma \left(R_{1},R_{2}\right)=\Sigma \left(R_{1},S\right)\Sigma {\left(S\right)}^{-1}\Sigma {\left(R_{2},S\right)}^{⊤}+\Sigma \left(R_{1},R_{2}|S\right)\\ \Sigma_{ind}\left(R_{1},R_{2}|S\right)=0 \end{array}\text{,}$$

which are symmetric under permutation of *R*_1_ and *R*_2_.

Further results of simulations and bias corrections are provided in Note S4.

### Details of two-source PID analysis of real neural data

#### Mouse auditory cortex data recorded during a sound discrimination task

We reanalyzed a previously published dataset^36^ in which the activity of several tens to a few hundreds of neurons was recorded simultaneously using *in vivo* two-photon calcium imaging from A1 L2/3 neurons in head-fixed mice during a pure-tone discrimination task (Figure 6A). After a pre-stimulus interval of 1 s, head-fixed mice were exposed to either a low-frequency (7 or 9.9 kHz) or high-frequency (14 or 19.8 kHz) tone for 1 s. Mice were trained to report their perception of the tone through their behavioral choice by licking a waterspout in the post-stimulus interval (0.5–3 s from stimulus onset) after hearing a low-frequency tone (target tones) and holding still after hearing high-frequency tones (non-target tones). Two-photon calcium imaging was used to record calcium fluorescence signals from individual A1 L2/3 neurons with an imaging frame rate of 30 Hz, from which estimated spike rates were obtained with deconvolution.^36^ We analyzed neurons recorded from *n* = 12 fields of view (FOVs) from *n* = 12 animals. For consistency with the original publication,^36^ we only considered individual neurons that carried significant intersection information,^66^ that is, information about the stimuli used to inform the behavioral discrimination, according to the statistical tests described in Francis et al.^36^ This led to selecting for PID analysis *n* = 375 individual neurons (out of the 2,792 recorded neurons), leading to *n* = 6,209 pairs of simultaneously recorded neurons used for the PID analysis.

For the PID analysis, we identified for each neuron the imaging time frame within the trial of maximal intersection information^66^ as described in Francis et al.^36^ We then considered for each neuron a time window of *n* = 10 imaging frames (333 ms) around the peak intersection information timeframe (we call this the peak time window for the neuron). The activity of each neuron within the peak time window was discretized into *R* = 3 bins corresponding to 0, 1, or >1 spikes. The stimulus set used for this analysis was binary (*S* = 2), dividing the presented sound tones into the low-frequency (*s* = 0) and high-frequency (*s* = 1) categories. For consistency with the original publication,^36^ which analyzed joint stimulus information carried by pairs of neurons without breaking it up with the PID, we estimated the total stimulus information that was jointly carried by pairs of neurons following a time-lagged approach. Namely, we computed the time-lagged stimulus information carried jointly by the activity of each pair of neurons using Equation 2 and using as responses *r*_1_ or *r*_2_, the discretized responses of each neuron measured as detailed above in their respective peak time windows. The analysis of the full dataset is presented in Figure 6A, while a comparison between the half-data and full-data analyses is shown in Figure S16A.

#### Mouse hippocampus data recorded during virtual spatial navigation

We reanalyzed a previously published dataset^63^ in which the activity of several tens to a few hundreds of neurons was recorded simultaneously using *in vivo* two-photon calcium imaging from CA1 neurons in head-fixed mice during virtual-reality navigation of a linear track (Figure 6B). Calcium fluorescence signals were obtained using the jRCaMP1a calcium indicator and an imaging frame rate of 3 frames per second. Full details of the experimental procedures and details of the ethical approval are reported in full in the original publication.^63^

We analyzed neurons recorded from *n* = 11 FOVs from *n* = 7 animals. Because hippocampal neurons encode position in space, we computed the mutual information between the spatial position *S* in which the animal was located in the linear track and the joint activity of two simultaneously recorded neurons *r*_1_ and *r*_2_. For consistency with the previous study reporting the original data,^63^ the spatial position *s* along the linear track was computed by binning the space along the track into *S* = 12 equipopulated bins. For consistency with the previous study,^63^ to quantify the neural activity *r*_*i*_ of each neuron, we took the calcium traces and binned them into *R* = 2 bins (only raw calcium traces and not deconvolved signals were available from Curreli et al.^63^). We computed information between neural activity and spatial position across all available time frames. For the PID analysis, we used all the *n* = 870 individual neurons present in the dataset, leading to *n* = 36,158 pairs of simultaneously recorded neurons used for the PID analysis. The analysis of the full dataset is presented in Figure 6B, while a comparison between the half-data and full-data analyses is shown in Figure S16B.

#### Monkey auditory cortex data recorded during passive listening to natural sounds

We reanalyzed a previously published dataset,^11^ which consisted of simultaneous recordings (*n* = 4 different recording sessions) with multiple electrodes from the auditory cortex (A1) of a monkey (*Macaca mulatta*) passively listening to natural sounds with four different levels of natural noise. Each natural sound snippet was 20 s long and was repeated for 80–120 total trials across sessions (mean 108 trials). We computed the joint information (and its PID) carried by neural activity *R*_1_,*R*_2_ (defined as spike counts discretized in *R* = 2 levels) concerning which of *S* = 399 different sound segments (each 50 ms long) were presented. The analysis of the full dataset is presented in Figure 6C, while a comparison between the half-data and full-data analyses is shown in Figure S16C.

#### Human resting-state fMRI data

The dataset of functional and structural neuroimaging data used in this work came from the Human Connectome Project (HCP, https://www.humanconnectome.org/study/hcp-young-adult/document/q3-data-release), Release Q3.^85^^,^^86^ Per HCP protocol, all subjects gave written informed consent to the HCP consortium. These data contained fMRI and diffusion-weighted imaging (DWI) acquisitions from 100 unrelated subjects of the HCP 900 data release.^85^^,^^86^ All HCP scanning protocols were approved by the local Institutional Review Board at Washington University in St. Louis. Detailed information about the acquisition and imaging is provided in the dedicated HCP publications. In brief, anatomical (T1-weighted) images were acquired in axial orientation, with FOV = 224 × 224 mm, voxel size 0.7 mm^3^ (isotropic), TR 2,400 ms, TE 2.14 ms, and flip angle 8°. fMRI data (1,200 volumes) were acquired with EPI sequence, 2 mm isotropic voxel size, TR 720 ms, TE 33.1 ms, flip angle 52°, and 72 slices.

We used the minimally preprocessed fMRI data from the HCP, which includes bias field correction, functional realignment, motion correction, and spatial normalization to Montreal Neurological Institute (MNI-152) standard space with 2-mm isotropic resampling resolution. We also removed the first 10 volumes to allow magnetization to reach steady state. Additional denoising steps were performed using the CONN toolbox (http://www.nitrc.org/projects/conn), version 17f.^87^ To reduce noise due to cardiac and motion artifacts, we applied the anatomical CompCor method of denoising the functional data. The anatomical CompCor method (also implemented within the CONN toolbox) involves regressing out of the functional data of each individual the first five principal components corresponding to white matter signal and the first five components corresponding to cerebrospinal fluid signal, as well as six subject-specific realignment parameters (three translations and three rotations) and their first-order temporal derivatives, in addition to nuisance regressors identified by the artifact detection software ART.^87^^,^^88^ The subject-specific denoised BOLD signal time series were linearly detrended and band-pass filtered between 0.008 and 0.09 Hz to eliminate both low-frequency drift effects and high-frequency noise. Human brains were parcellated into 1,000 ROIs covering the entire cortex. The 1,000 cortical ROIs were obtained from the scale-1000 version of the recent local-global functional parcellation of Schaefer et al.^89^ The time courses of denoised BOLD signals were averaged between all voxels belonging to a given atlas-derived ROI using the CONN toolbox. The analysis of the full dataset is presented in Figure 6D.

### Synergy and redundancy bias in three-source PID

#### Definition of three-way redundancy and three-way synergy with the MMI PID

We study the bias of the MMI PID of the joint information with three source variables *R*_1_, *R*_2_, *R*_3_ and one target variable *S* for both the discrete and the Gaussian cases.^22^^,^^34^^,^^98^ For three source variables, PID breaks down the joint Shannon information *I*(*S*;*R*_1_,*R*_2_,*R*_3_) into 18 components corresponding to a combination of redundant, unique, and synergistic information.^22^ Here, we focus on two terms that have a straightforward interpretation and have been used for the analysis of real and artificial neural networks.^90^^,^^98^ The first is the three-way redundancy (*Red*_3_), which quantifies the information that each of the sources *R*_1_, *R*_2_, and *R*_3_ encode about *S*. The second is the three-way synergy (*Syn*_3_), which quantifies the information about *S* that is available only from the joint observation of (*R*_1_, *R*_2_, *R*_3_) and not from any individual source or pair of sources. Using MMI, the three-way redundancy is defined as^97^^,^^98^

(Equation 30) $$Red_{3}=\min\left[I\left(S;R_{1}\right),I\left(S;R_{2}\right),I\left(S;R_{3}\right)\right]\text{,}$$

while three-way synergy has the following expression^90^^,^^98^:

(Equation 31) $$Syn_{3}=I\left(S;R_{1},R_{2},R_{3}\right)-\max\left[I\left(S;R_{1},R_{2}\right),I\left(S;R_{1},R_{3}\right),I\left(S;R_{2},R_{3}\right)\right]\text{.}$$

Thus, the bias of three-way synergy *Syn*_3_ and three-way redundancy *Red*_3_ with the MMI PID can be estimated from the bias of Shannon information quantities. For analytical considerations on the bias of the three-way synergy and three-way redundancy, see Note S8.

### Details of the simulations used to test the three-source PID

To evaluate numerically the bias and test bias-correction procedures for the three-source MMI PID (Figure 7), we performed numerical simulations.

To test the discrete PID, we simulated a discrete process that was fully synergistic. Source variables *X*_1_, *X*_2_, and *X*_3_ were sampled from a uniform distribution over *R* possible values, and the target variable was defined as the sum of all source variables, taken modulo *R*:

(Equation 32) $$Y=\left(X_{1}+X_{2}+X_{3}\right)\;\mathrm{mod}\;R\text{.}$$

This is an extension of the 3-bit parity system^109^^,^^110^ using modulo *R* instead of 2 to have a similar structure in the number of bins as in the two-source simulations. In this setting, the value of each individual source, or the combination of any pair of sources, does not provide any information about the target variable, while the combination of the three sources fully determines *Y*. Therefore, by construction, only the three-way synergy *Syn*_3_ PID component is non-zero.

For the Gaussian simulations, we sampled from a multivariate normal distribution such that each source *X*_*i*_ and the target *Y* share a common latent signal *S* plus independent noise ($X_{i}=S+\varepsilon_{i}^{x}$, *Y* = *S* + *ε*^*y*^), with ε∼N(0,1), yielding a block-diagonal covariance matrix where diagonal elements represent total variance ($\sigma_{S}^{2}+\sigma_{\varepsilon }^{2}$) and off-diagonal elements represent the shared signal variance ($\sigma_{S}^{2}$). This system has a ground-truth non-zero redundancy component only.

### Three-source PID analysis of real MEG data

We analyzed a previously published dataset^77^ in which MEG signals were recorded from healthy human participants (*n* = 30) performing a visual evidence accumulation task. In each trial, participants observed a sequence of 12 visual samples (checkered disks), whose orientation from the midline was drawn from a Gaussian distribution with a given generative mean value. Participants performed a decision-making task consisting of estimating the generative mean of these samples. Here, we focused on the encoding by MEG activity of the orientation of this sample, which is thus focused on the sensory encoding rather than on the decision-making aspects of the experiment. MEG data were acquired with a 275-channel CTF system at a sampling rate of 1,200 Hz. The preprocessed MEG data were source-reconstructed onto the 180 cortical areas of the Glasser atlas^111^ using individual head models generated with the aid of T1-weighted structural MRI scans acquired for each participant. To reduce the dimensionality within each area, we focused on the first principal component of MEG activity computed across all vertices in each area.

The Gaussian PID assumes that the target-source distributions are approximately Gaussian. The target variable (visual sample orientation) was drawn from a Gaussian distribution by experimental design.^77^ To verify that this assumption also held for the sources conditioned on the target, we divided the target range into eight equal-width bins spanning the minimum to maximum angle presented across all trials. This gave a bin width of 12° ± 0.25° (mean ± SD across participants). The number of trials available to compute this angle-specific distribution was 90 ± 60 (mean ± SD across participants). We estimated the angle-specific empirical distribution of MEG responses within each bin using kernel density estimation (KDE), then assessed normality with Shapiro-Wilk tests across all 240 angle-specific distributions obtained across all subjects from V1. Only 6.25% of distributions were significantly different from a Gaussian under a false discovery rate (FDR) of 5% (Benjamini-Hochberg FDR correction, *q* < 0.05), meaning deviations from Gaussianity were only slightly more frequent than expected by chance.

For the PID analysis, we quantified the information provided by three neural sources about the orientation of the first visual sample. The three-source PID analysis was applied to both the continuous multivariate data and the discretized data. For the discrete analysis, the three sources were defined as the first principal component of activity from cortical areas V1, V2, and V3. The time series of each source and the stimulus orientation were then discretized into *R* = 3 equipopulated bins. For the multivariate Gaussian analysis, we defined three large-scale sources by pooling the first principal component of activity across multiple cortical areas: source 1 comprised all 26 areas within the visual cortex, source 2 included all 47 areas from the prefrontal cortex, and source 3 consisted of all 25 areas from the somatosensory and motor cortices. For both analyses, we computed PID components using a naive plugin estimator and compared the results against appropriate bias-correction schemes to quantify the effects of finite sampling bias (see Figure 8). We analyzed either pre-stimulus data (MEG responses collected in the 150 ms prior to the onset of stimulus presentation) or post-stimulus data (MEG data recorded in the 150 ms following the onset of stimulus presentation).

## Resource availability

### Lead contact

Requests for further information and resources should be directed to and will be fulfilled by the lead contact, Stefano Panzeri (s.panzeri@uke.de).

### Materials availability

This study did not generate new materials.

### Data and code availability

Code for simulating and analyzing data is made available at our repository at https://doi.org/10.5281/zenodo.20085438.^112^ The code uses the MINT toolbox.^64^ The real neural data used here in Figures 6 and S16 were published previously. Dataset 1 was released with the original publication.^36^ Dataset 2 was first published in Curreli et al.^63^ and is available for download from Lorenz et al.^64^ Dataset 3 was published in Kayser et al.^11^ and is made available in our repository at https://doi.org/10.5281/zenodo.20085438.^112^ Dataset 4 is publicly available from the original publication.^86^ Dataset 5 is publicly available from the original publication.^77^

## Acknowledgments

We are grateful to Hame Park and Tobias H. Donner for discussions and support on the analysis of their MEG data analyzed in the present study. This work was supported by the 10.13039/100014370Simons Foundation Autism Research Initiative grant 982347 (to S.P.), the German Federal Ministry of Education and Research BMBF 01GQ2404 (to S.P.), the European Union’s European Research Council NEUROPATTERNS grant 647725 (to T.F.) and cICMs ERC-2022-AdG-101097402 (to A.K.E.), the European Union’s Horizon Research and Innovation Programme grants 101206609 (Marie Sklodowska-Curie Global Fellowship AstroError to MC) and 101152984 (EU Marie Sklodowska-Curie Action to S.B.M.), a Wellcome Early Career Award 226924/Z/23/Z (to A.I.L.), and St. John’s College Cambridge (A.I.L.). The Funders had no role in study design, data collection and analysis, decision to publish, or preparation of the manuscript. Views and opinions expressed in this article are those of the authors only and do not necessarily reflect those of the Funders. None of the Funders or granting authorities can be held responsible for them.

## Author contributions

S.P. conceived and supervised the project; G.M.L., N.M.E., L.K., M.C., D.O., and S.P. performed research (simulations, data analysis, algorithms development, and analytical calculations); S.C., C.K., and T.F. provided experimental data; S.B.M. and S.C. curated data; A.K.E., A.I.L., and S.P. provided computational infrastructure; S.P., A.K.E., T.F., A.I.L., M.C., and S.B.M. provided funding; and G.M.L., N.M.E., L.K., M.C., A.I.L., and S.P. wrote the paper. All authors edited and commented on the paper and contributed to interpretation of results.

## Declaration of interests

The authors declare no competing interests.

## Supplemental information

Document S1. Figures S1–S18, Tables S1–S2, and Notes S1–S8 and Equations S1–S25

Document S2. Article plus supplemental information
