# Supplementary material for: Sampling bias corrections for discrete and Gaussian partial information decompositions
Source: Patterns (N Y). 2026 Jul 23;7(9):101619. doi: 10.1016/j.patter.2026.101619 (PMC13576656; doi:10.1016/j.patter.2026.101619)
Supplement: Supplementary file 1 [file mmc1.pdf]

Patterns, Volume *7*

Supplemental information

Sampling bias corrections for discrete and Gaussian partial information decompositions

Gabriel Matías Lorenz, Nicola Marie Engel, Loren Koçillari, Marco Celotto, Davide Orsenigo, Sebastiano Curreli, Simone Blanco Malerba, Andreas K. Engel, Christoph Kayser, Tommaso Fellin, Andrea I. Luppi, and Stefano Panzeri

Supplemental Information

Content

Supplemental Figures

Figure S1. Schematic of the Partial Information Decomposition with two sources.
Figure S2. Bias properties and bias corrections for the sum of unique information terms.
Figure S3. Bias of the plugin PID values and residual bias of the bias-corrected PID values as a function of the number of parameters defining the probability distributions.
Figure S4. Dependence of the bias of the discrete BROJA PID on the number of samples N used to compute it.
Figure S5. Bias correction performance for the discrete BROJA PID.
Figure S6. Bias correction performance for the discrete I_{min}.
Figure S7. Bias correction performance for the discrete I_{MMI}.
Figure S8. Residual bias across information levels.
Figure S9. Bias and performance of bias corrections for the Gaussian gPID as a function of the number of dimensions d.
Figure S10. Bias and performance of bias corrections for the Gaussian gPID as a function of the number of samples N.
Figure S11. Bias corrections performance for gPID.
Figure S12. Comparison of the residual bias of the discrete merged correction using optimized and equal weighting.
Figure S13. Using a Gaussian PID on discrete neural data.
Figure S14. Comparison of analytical expressions of the bias for the Gaussian entropy, and evaluations of the residual mutual information bias.
Figure S15. Tolerance analysis performed on the merged-corrected residual synergy and redundancy bias and on the plugin synergy and redundancy bias.
Figure S16. Comparison of PID bias corrections on each of the three full real neural datasets and on the same datasets each recomputed with half the trials.
Figure S17. Further analyses of the human resting-state fMRI dataset (dataset 4).
Figure S18. Evaluation of the bias and the performance of bias correction methods when computing discrete BROJA PID of two 10-dimensional sources with an SVM dimensionality reduction.

Supplemental Tables

Table S1. Summary of support sizes and simulation parameters used across all figures, including main text and supplementary figures.
Table S2. Overview of simulation parameters for simulated scenarios of discrete and Gaussian simulations.

Supplementary Notes

Supplementary Note 1. Other discrete PID measures.
Supplementary Note 2. Key properties of PID components for two source variables.
Supplementary Note 3. Illustration of the origin of the bias.
Supplementary Note 4. Further results of simulations and bias corrections.
Supplementary Note 5. Application of Gaussian PID to simulated spike count data.
Supplementary Note 6. Analytical considerations on the bias of discrete and Gaussian PIDs.

Supplementary Note 7. Study of how to scale up calculations and bias corrections of discrete PID using dimensionality reduction.
Supplementary Note 8. Analytical derivation of Red_3 and Syn_3 bias.

Figure S1

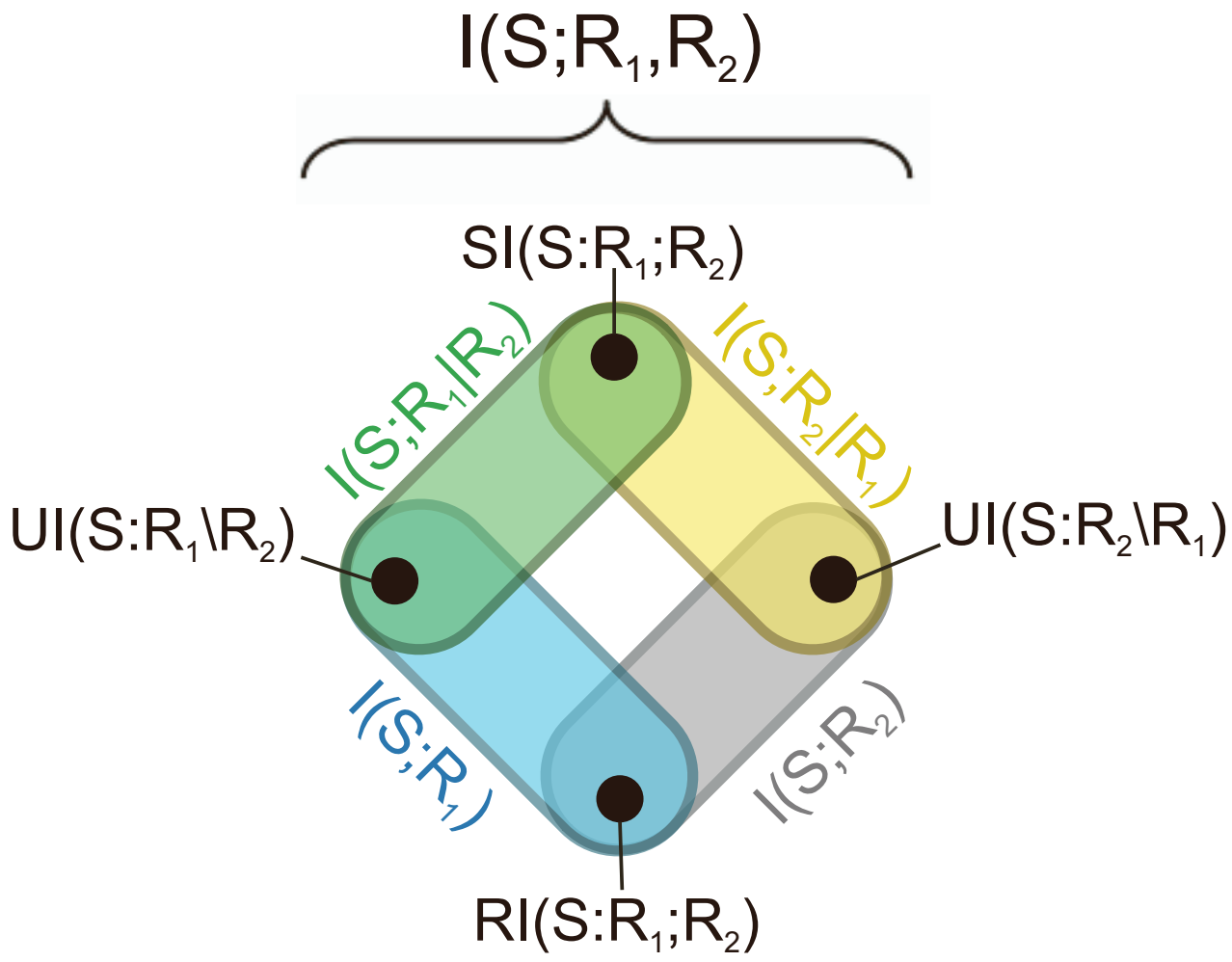
I(S;R1,R2)
SI(S:R1;R2)
I(S;R1|R2)
I(S;R2|R1)
UI(S:R1\R2)
UI(S:R2\R1)
I(S;R1)
I(S;R2)
RI(S:R1;R2)

Figure S2

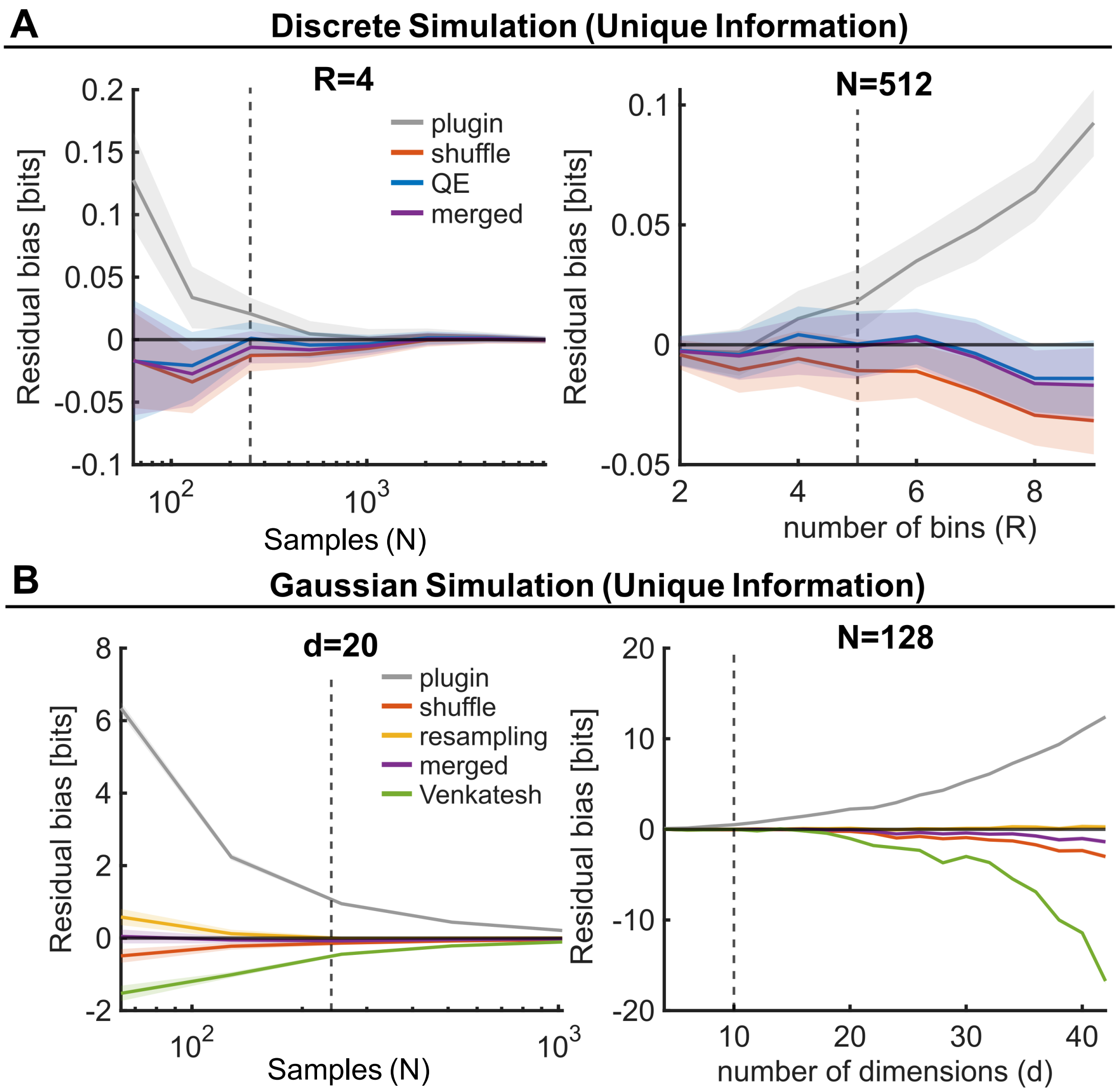
A
Discrete Simulation (Unique Information)
R=4
plugin
shuffle
QE
merged
Residual bias [bits]
Samples (N)
N=512
Residual bias [bits]
number of bins (R)
B
Gaussian Simulation (Unique Information)
d=20
plugin
shuffle
resampling
merged
Venkatesh
Residual bias [bits]
Samples (N)
N=128
Residual bias [bits]
number of dimensions (d)

Figure S3

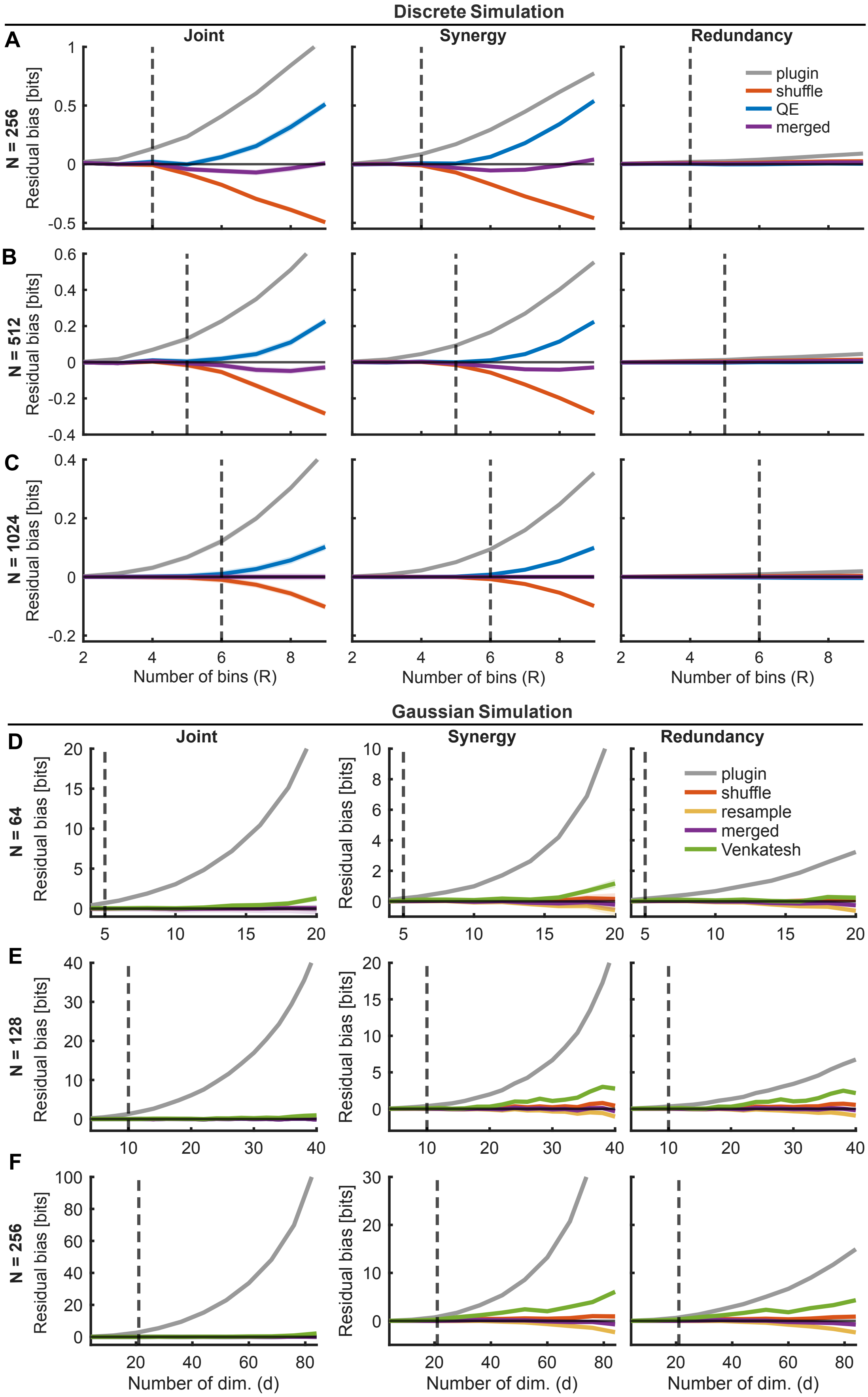

Discrete Simulation
A
B
C
Joint
Synergy
Redundancy
N = 256
N = 512
N = 1024
Residual bias [bits]
plugin
shuffle
QE
merged
Number of bins (R)
Gaussian Simulation
D
E
F
N = 64
N = 128
N = 256
plugin
shuffle
resample
merged
Venkatesh
Number of dim. (d)

Figure S4

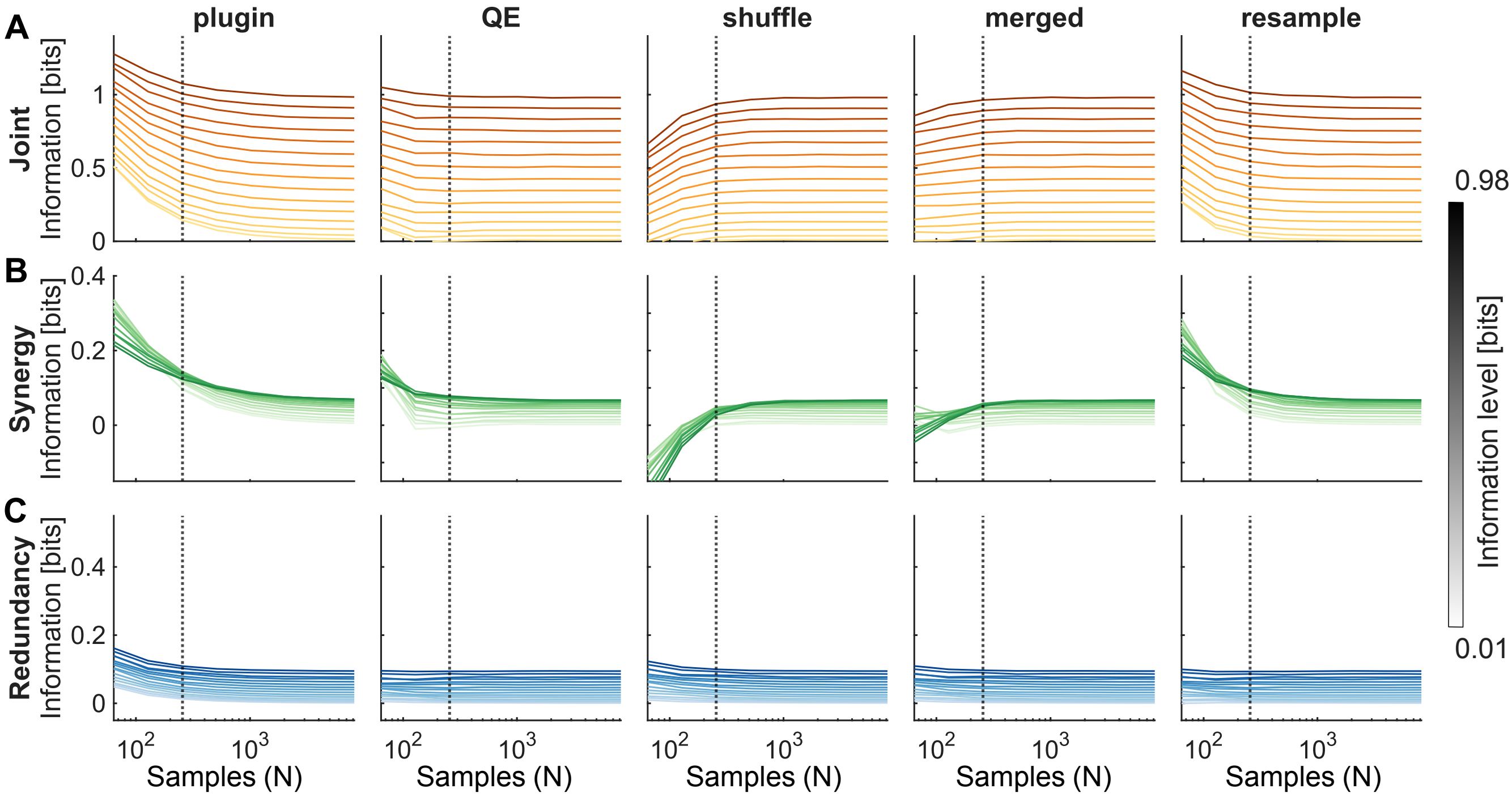

A
B
C
plugin
QE
shuffle
merged
resample
Joint
Synergy
Redundancy
Information [bits]
Samples (N)
Information level [bits]
0.98
0.01

Figure S5

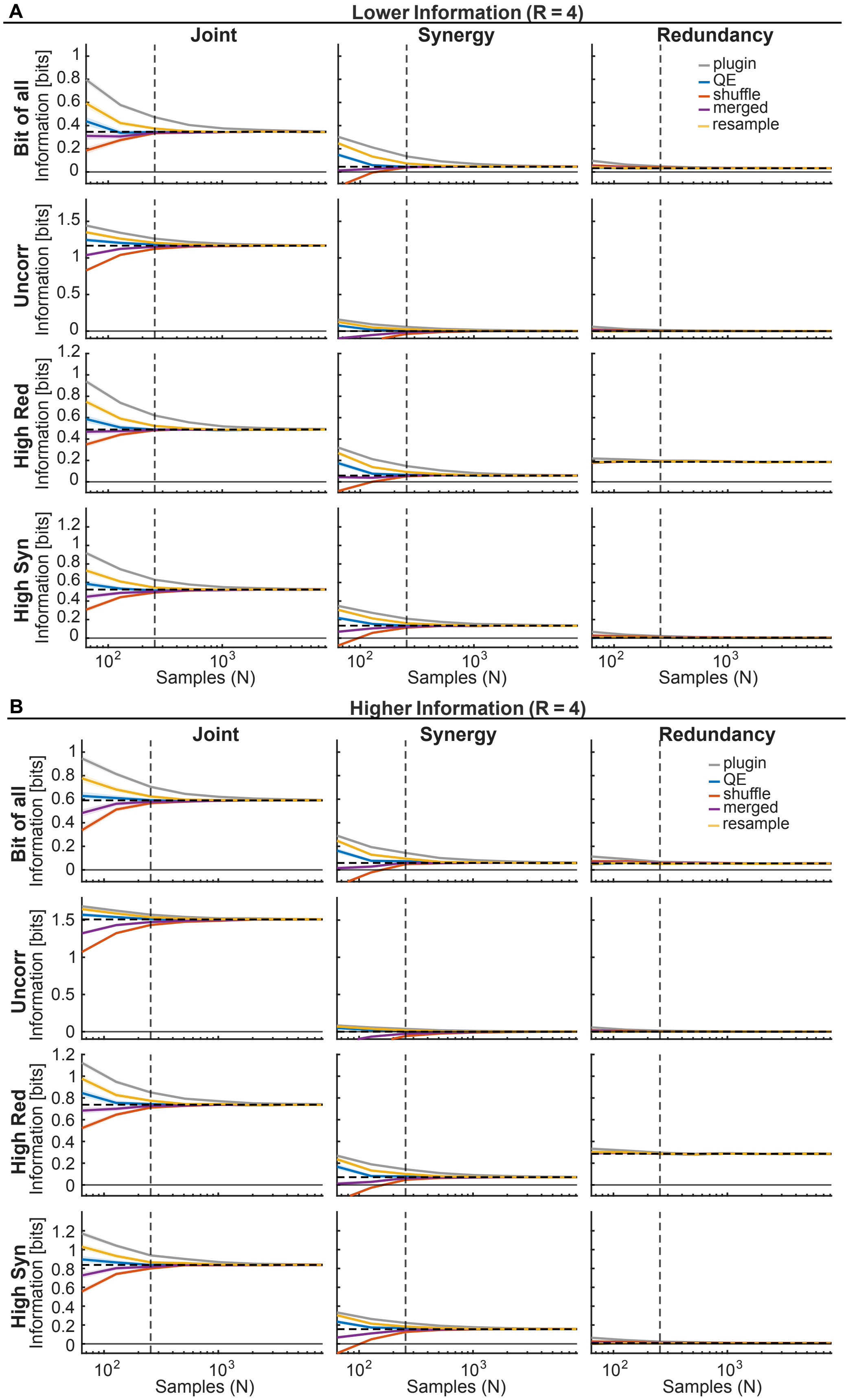

A
Lower Information (R = 4)
Joint
Synergy
Redundancy
plugin
QE
shuffle
merged
resample
Bit of all
Uncorr
High Red
High Syn
Information [bits]
Samples (N)
B
Higher Information (R = 4)

Figure S6

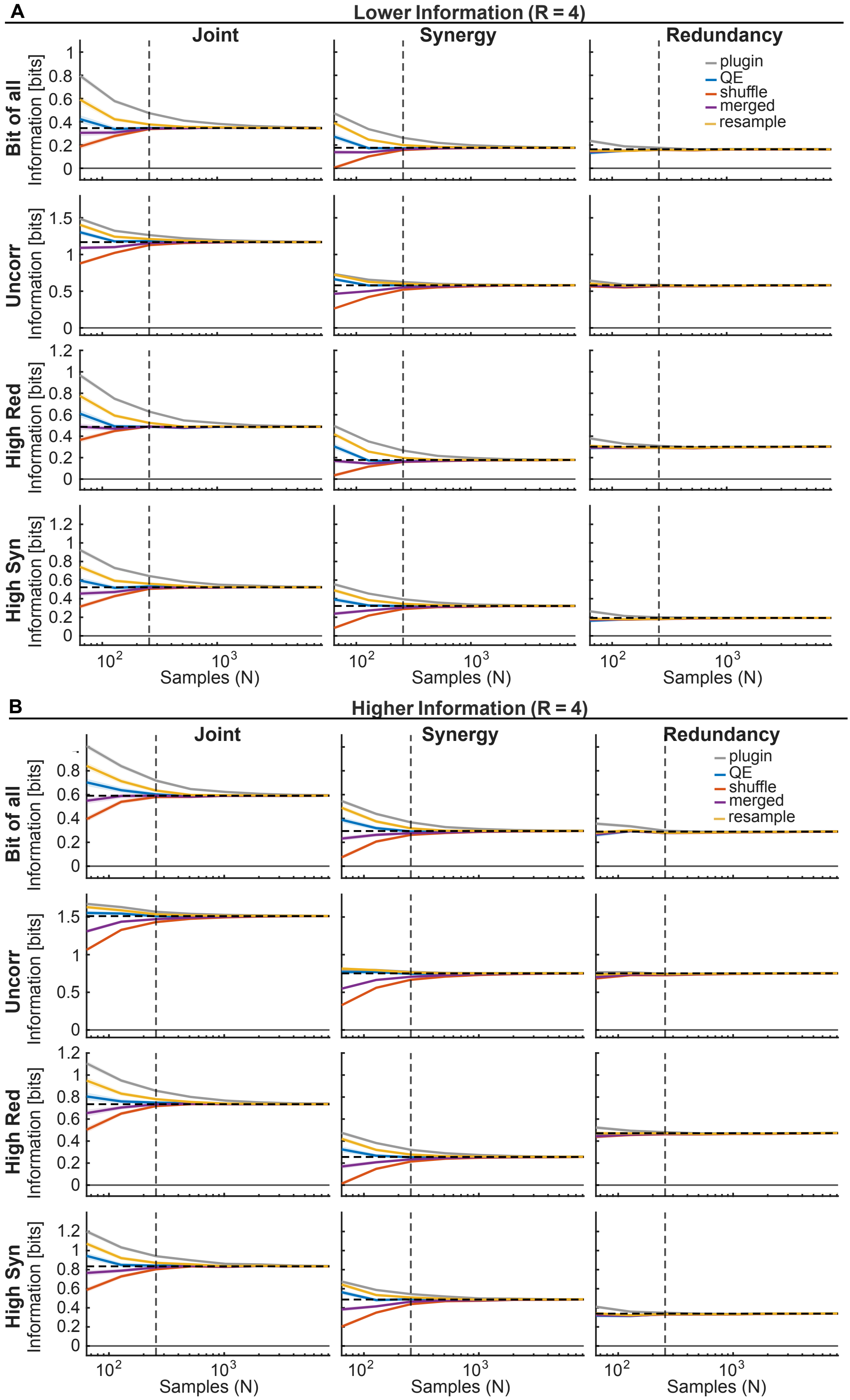
A
Lower Information (R = 4)
Joint
Synergy
Redundancy
plugin
QE
shuffle
merged
resample
Bit of all
Uncorr
High Red
High Syn
Information [bits]
Samples (N)
B
Higher Information (R = 4)

Figure S7

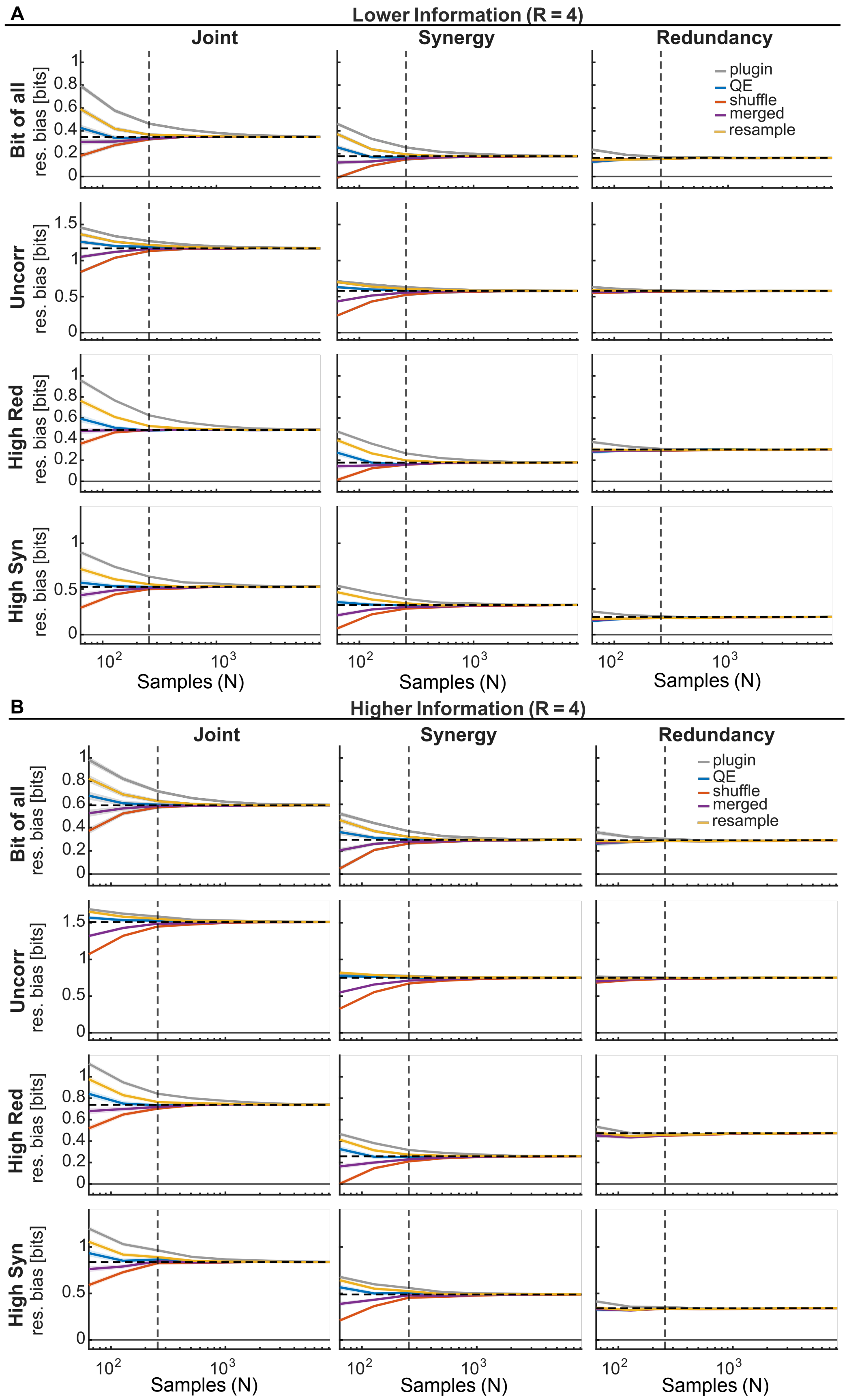

A
Lower Information (R = 4)
Joint
Synergy
Redundancy
plugin
QE
shuffle
merged
resample
Bit of all
Uncorr
High Red
High Syn
res. bias [bits]
Samples (N)
B
Higher Information (R = 4)

Figure S8

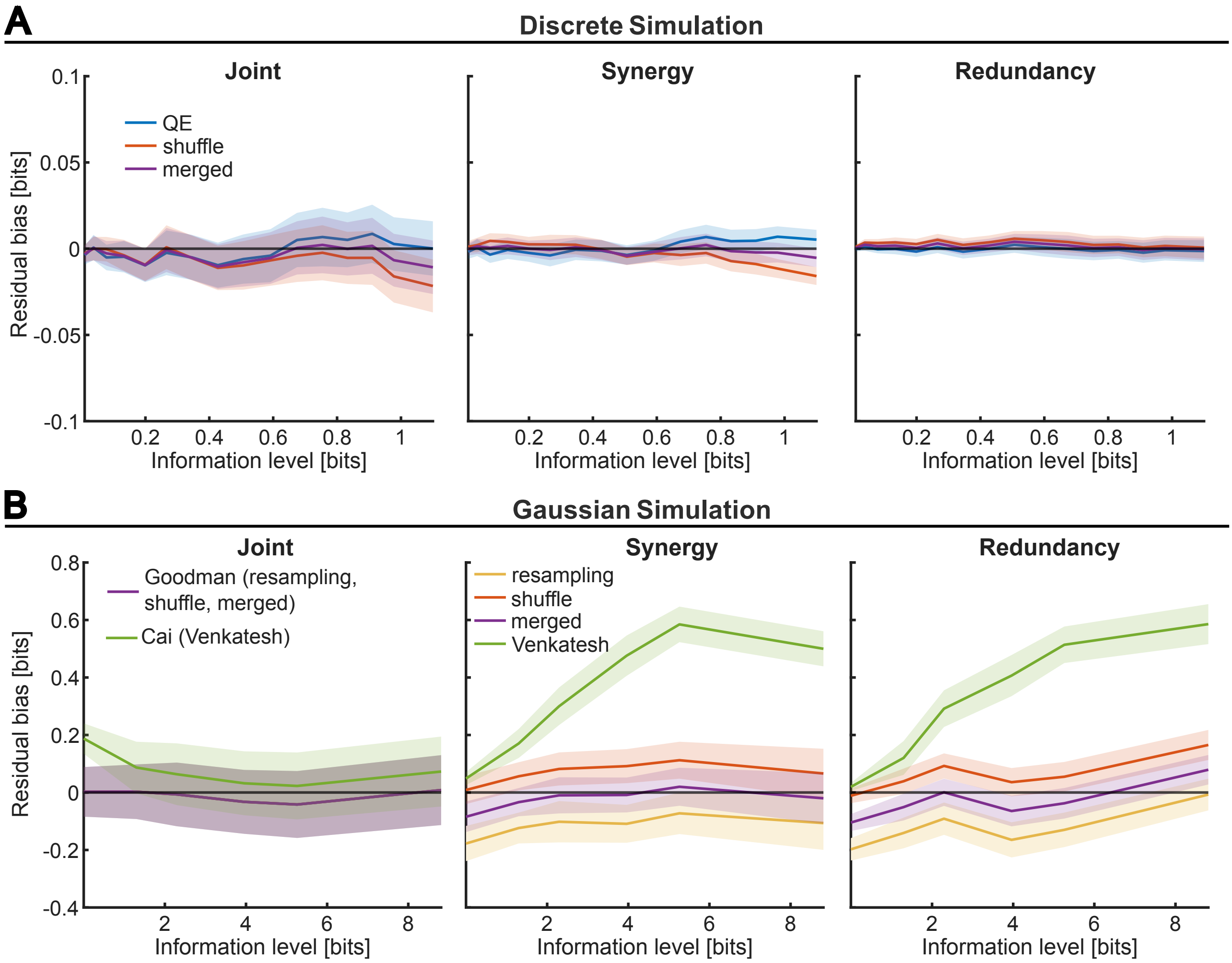
A
Discrete Simulation
Joint
Synergy
Redundancy
QE
shuffle
merged
Residual bias [bits]
0.1
0.05
0
-0.05
-0.1
0.2
0.4
0.6
0.8
1
Information level [bits]
B
Gaussian Simulation
Joint
Synergy
Redundancy
Goodman (resampling, shuffle, merged)
Cai (Venkatesh)
resampling
shuffle
merged
Venkatesh
Residual bias [bits]
0.8
0.6
0.4
0.2
0
-0.2
-0.4
2
4
6
8
Information level [bits]

Figure S9

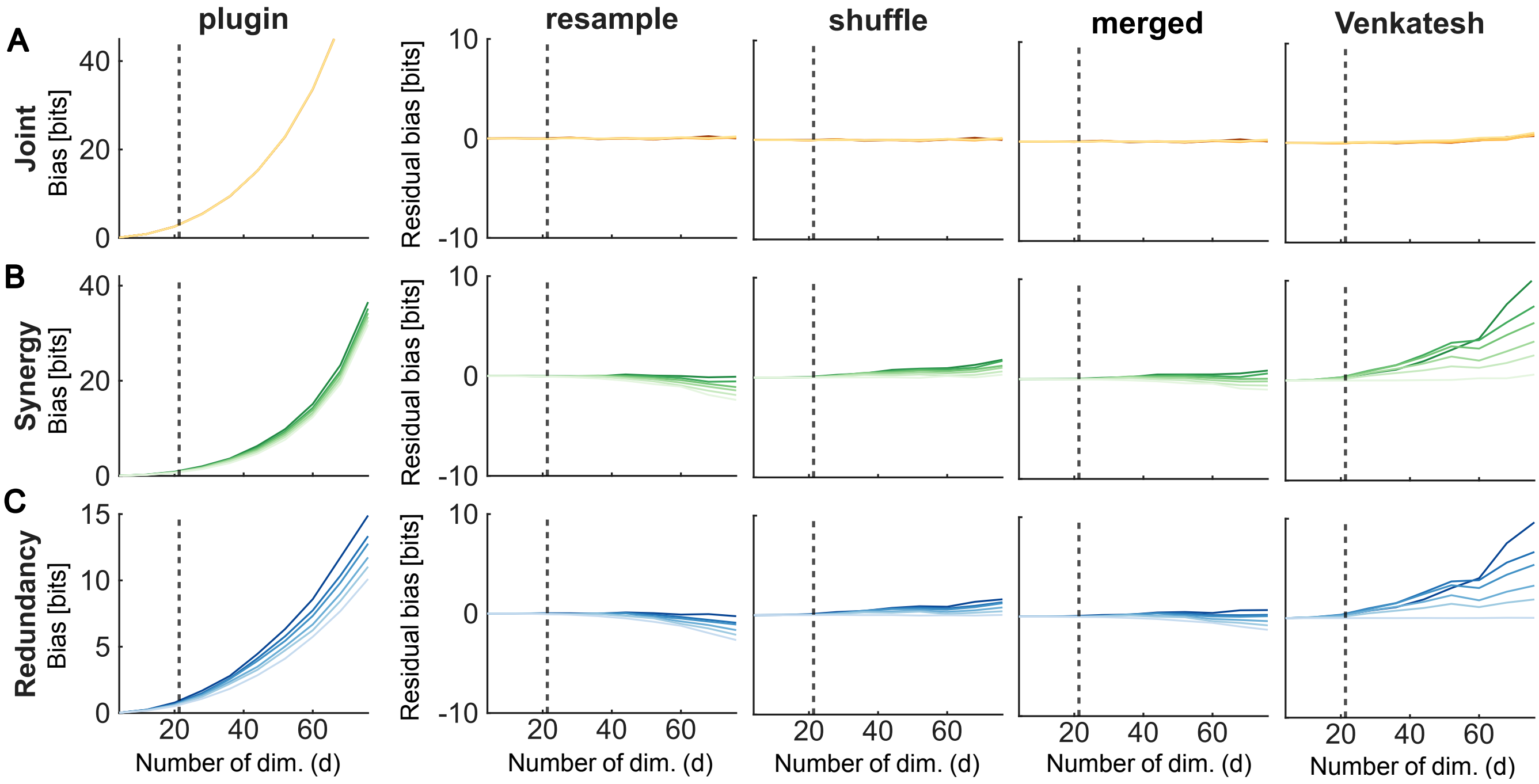

plugin
resample
shuffle
merged
Venkatesh
A
B
C
Joint
Synergy
Redundancy
Bias [bits]
Residual bias [bits]
Number of dim. (d)

Figure S10

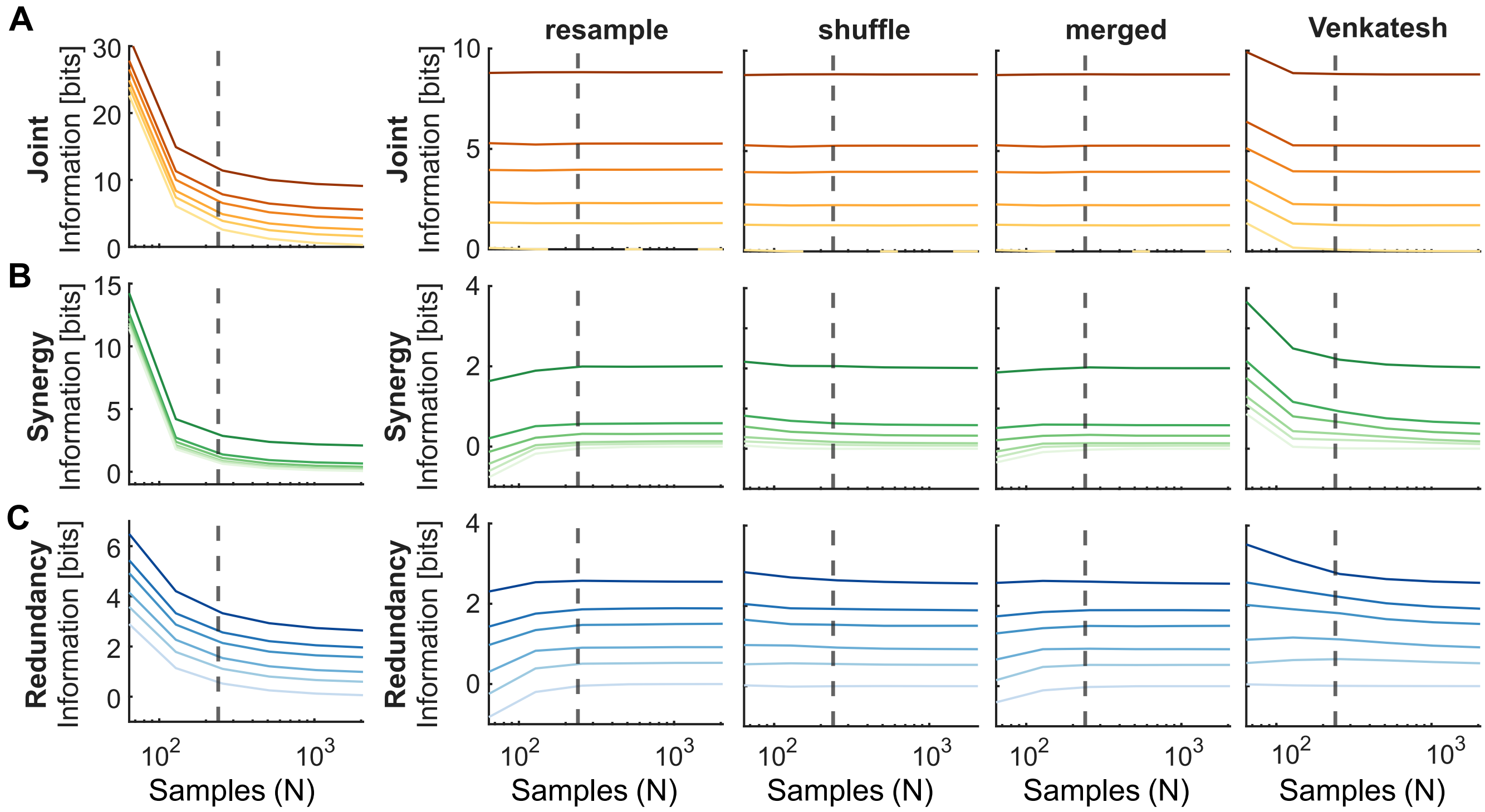

A
B
C
resample
shuffle
merged
Venkatesh
Joint
Information [bits]
Synergy
Information [bits]
Redundancy
Information [bits]
30
20
10
0
10
5
0
15
10
5
0
4
2
0
6
4
2
0
4
2
0
10^2
10^3
Samples (N)

Figure S11

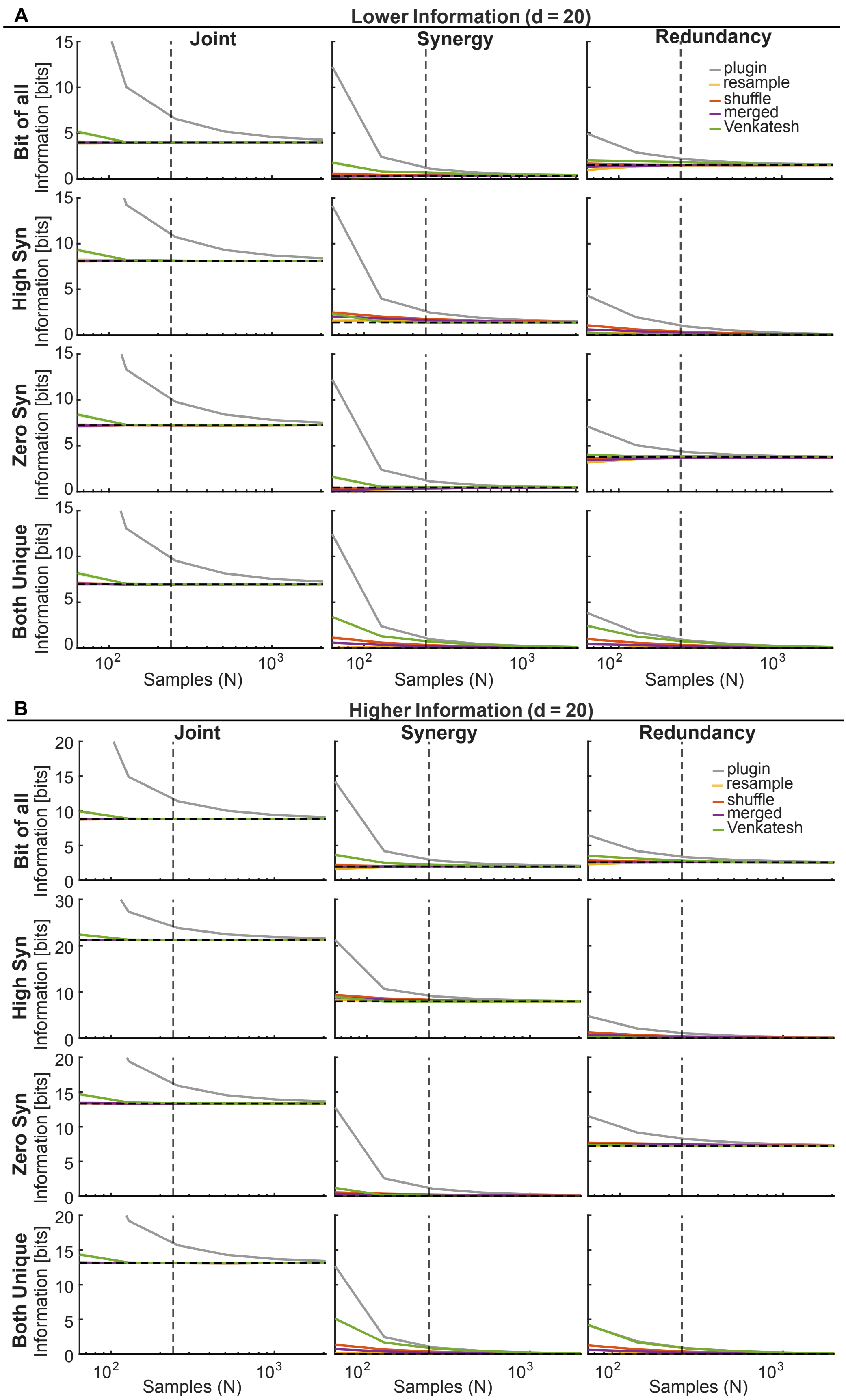
A
Lower Information (d = 20)
Joint
Synergy
Redundancy
plugin
resample
shuffle
merged
Venkatesh
Bit of all
High Syn
Zero Syn
Both Unique
Information [bits]
Samples (N)
B
Higher Information (d = 20)

Figure S12

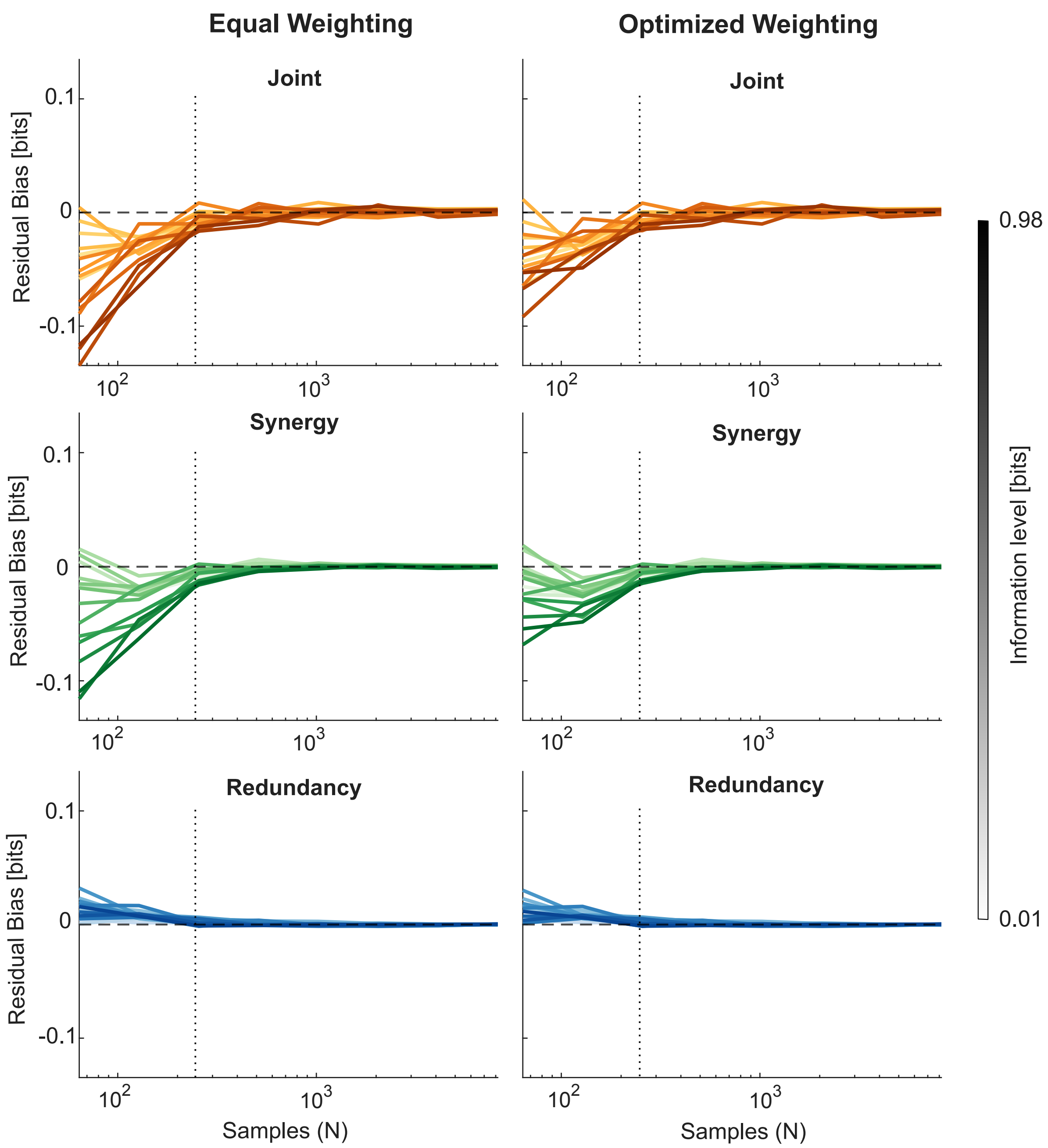

Equal Weighting
Optimized Weighting
Joint
Synergy
Redundancy
Residual Bias [bits]
0.1
0
-0.1
10^2
10^3
Samples (N)
Information level [bits]
0.98
0.01

Figure S13

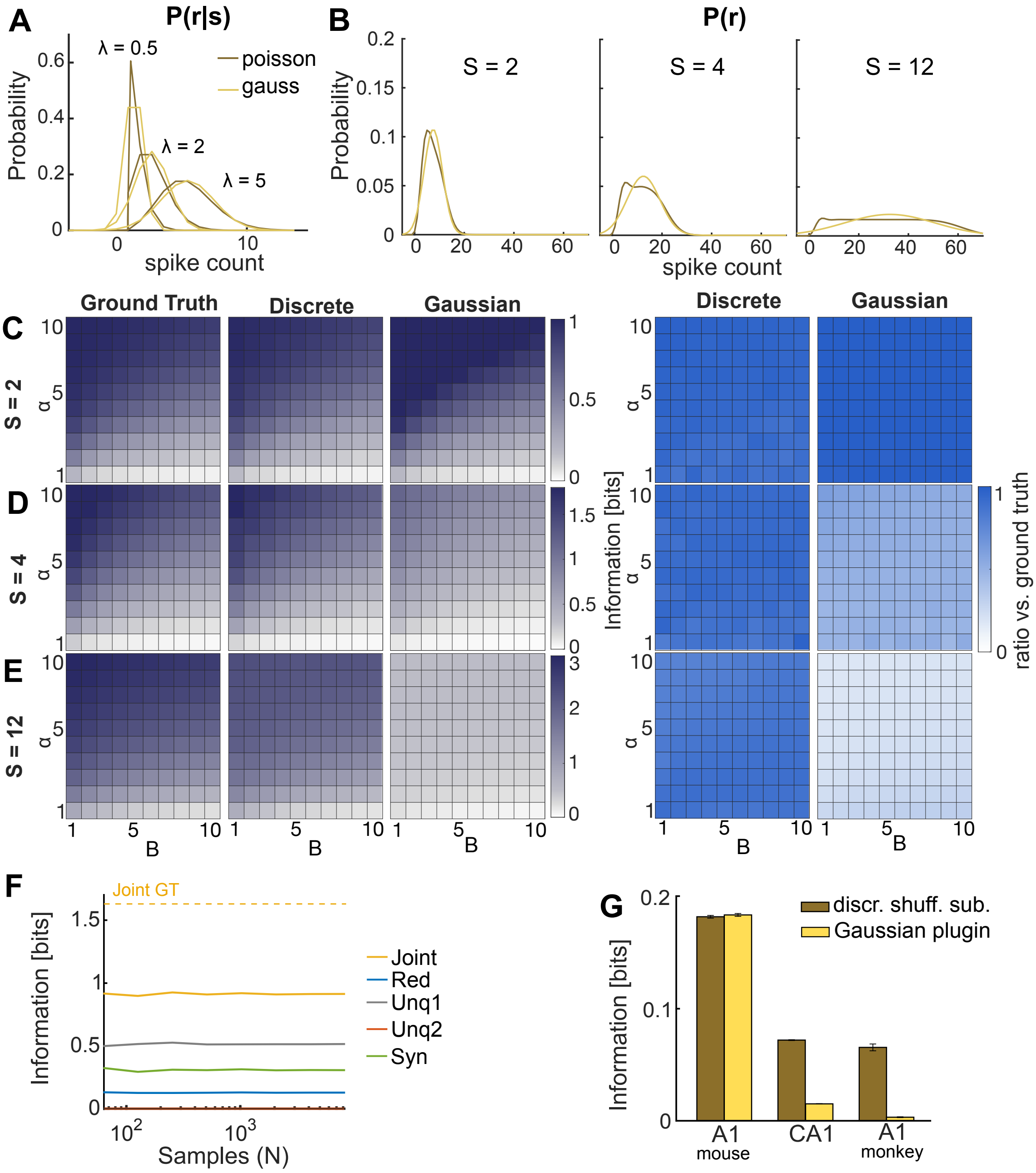

A
P(r|s)
Probability
λ = 0.5
λ = 2
λ = 5
poisson
gauss
spike count
B
P(r)
S = 2
S = 4
S = 12
spike count
C
D
E
Ground Truth
Discrete
Gaussian
S = 2
S = 4
S = 12
α
B
Information [bits]
ratio vs. ground truth
F
Joint GT
Joint
Red
Unq1
Unq2
Syn
Information [bits]
Samples (N)
G
discr. shuff. sub.
Gaussian plugin
Information [bits]
A1
mouse
CA1
A1
monkey

Figure S14

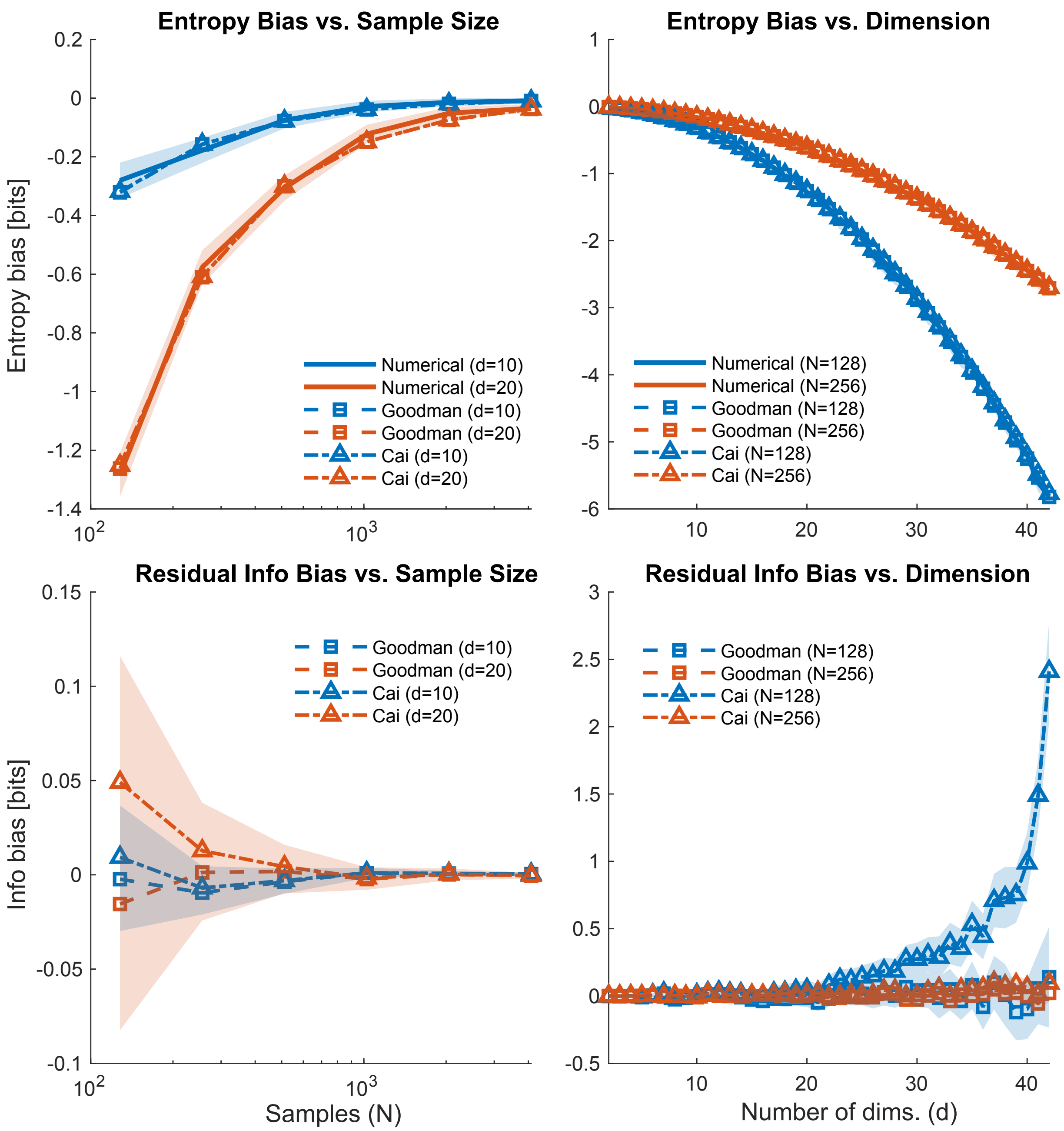

Entropy Bias vs. Sample Size
Entropy bias [bits]
Numerical (d=10)
Numerical (d=20)
Goodman (d=10)
Goodman (d=20)
Cai (d=10)
Cai (d=20)
Entropy Bias vs. Dimension
Numerical (N=128)
Numerical (N=256)
Goodman (N=128)
Goodman (N=256)
Cai (N=128)
Cai (N=256)
Residual Info Bias vs. Sample Size
Info bias [bits]
Goodman (d=10)
Goodman (d=20)
Cai (d=10)
Cai (d=20)
Samples (N)
Residual Info Bias vs. Dimension
Goodman (N=128)
Goodman (N=256)
Cai (N=128)
Cai (N=256)
Number of dims. (d)

Figure S15

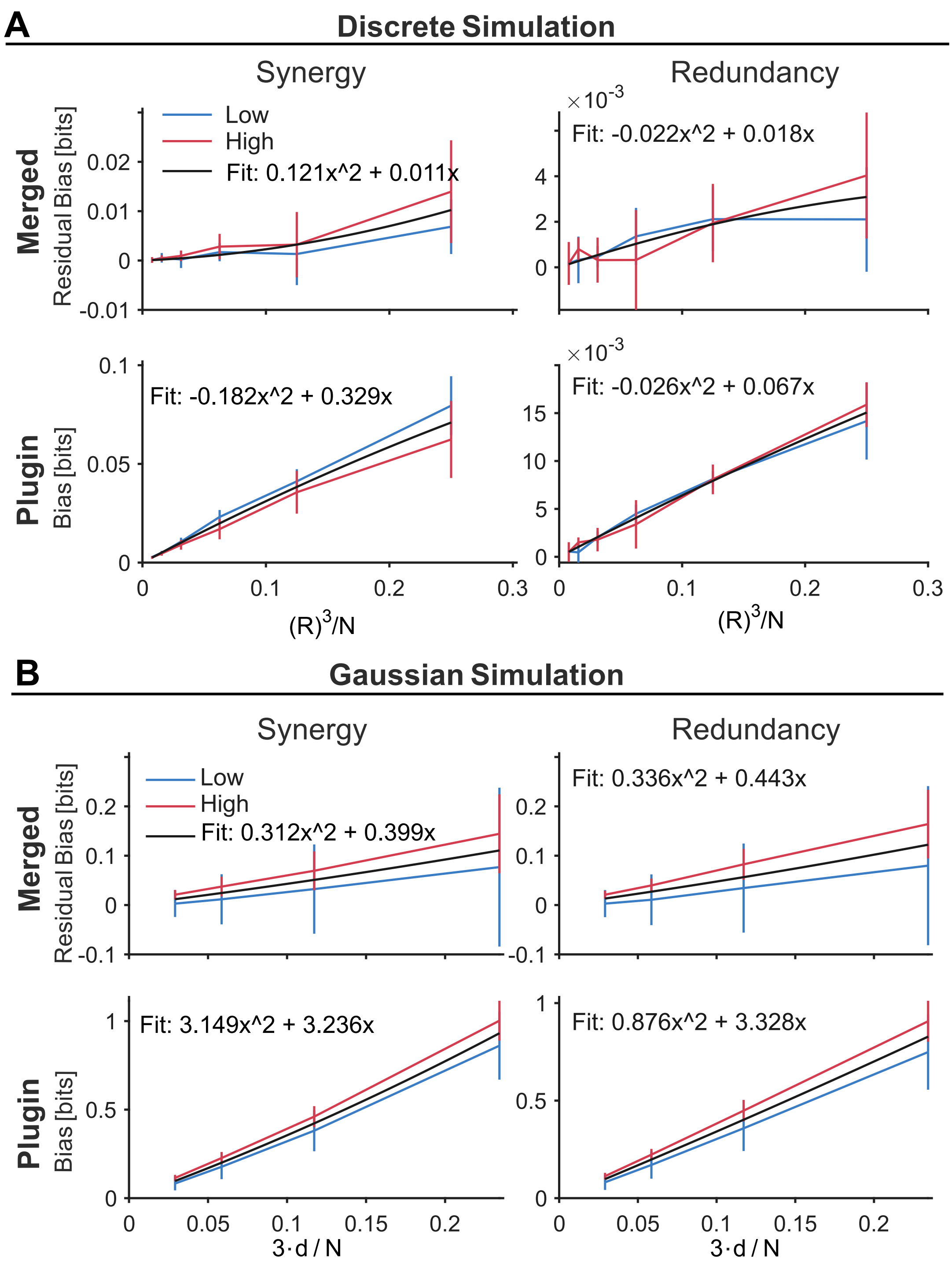
A
Discrete Simulation
Synergy
Redundancy
Merged
Residual Bias [bits]
Low
High
Fit: 0.121x^2 + 0.011x
Fit: -0.022x^2 + 0.018x
Plugin
Bias [bits]
Fit: -0.182x^2 + 0.329x
Fit: -0.026x^2 + 0.067x
(R)^3/N
B
Gaussian Simulation
Synergy
Redundancy
Merged
Residual Bias [bits]
Low
High
Fit: 0.312x^2 + 0.399x
Fit: 0.336x^2 + 0.443x
Plugin
Bias [bits]
Fit: 3.149x^2 + 3.236x
Fit: 0.876x^2 + 3.328x
3·d / N

Figure S16

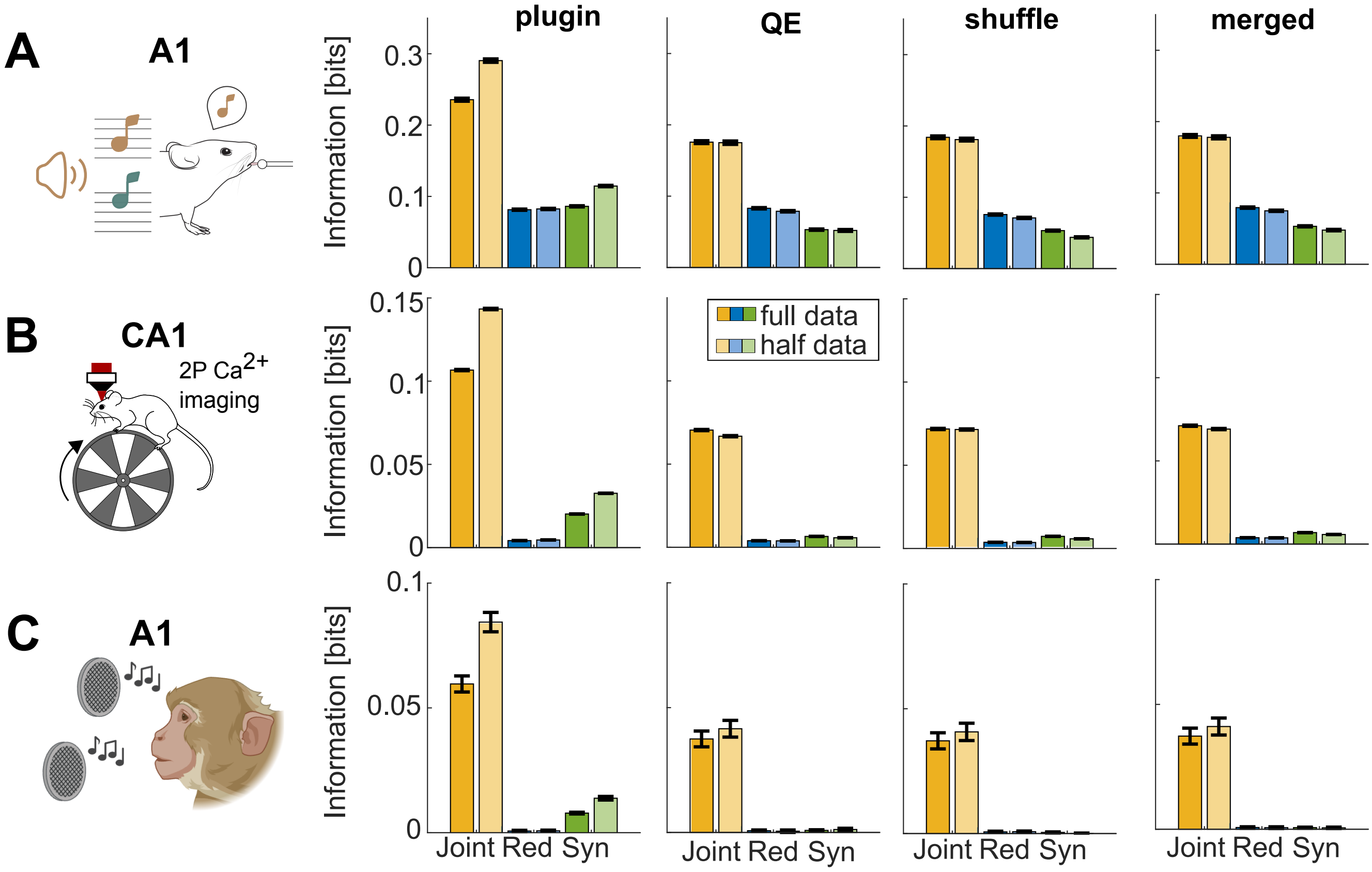

A
A1
B
CA1
2P Ca2+
imaging
C
A1
plugin
QE
shuffle
merged
Information [bits]
0.3
0.2
0.1
0
0.15
0.1
0.05
0
0.1
0.05
0
full data
half data
Joint Red Syn
Joint Red Syn
Joint Red Syn
Joint Red Syn

Figure S17

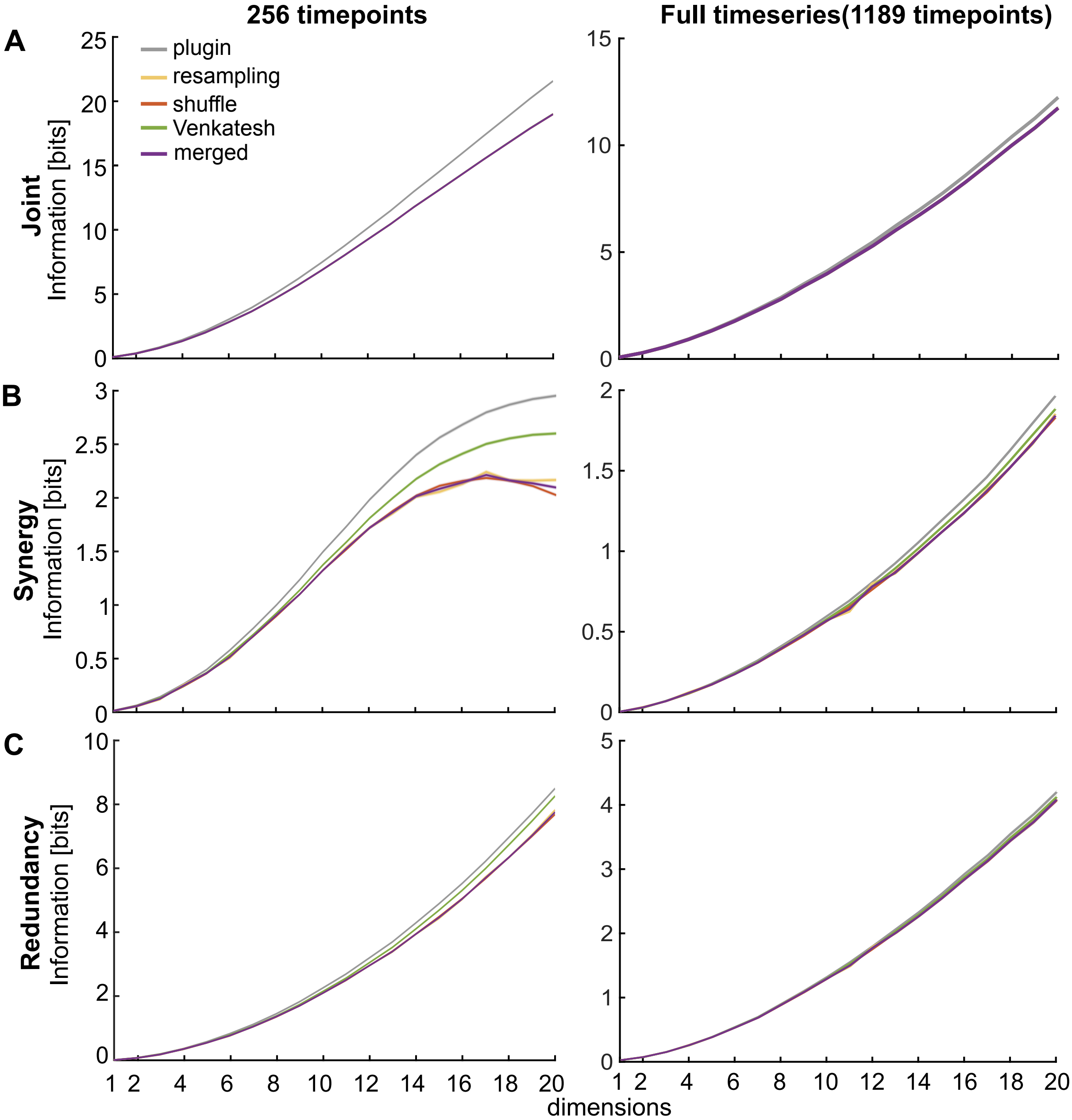
256 timepoints
Full timeseries(1189 timepoints)
A
B
C
plugin
resampling
shuffle
Venkatesh
merged
Joint
Synergy
Redundancy
Information [bits]
dimensions

Figure S18

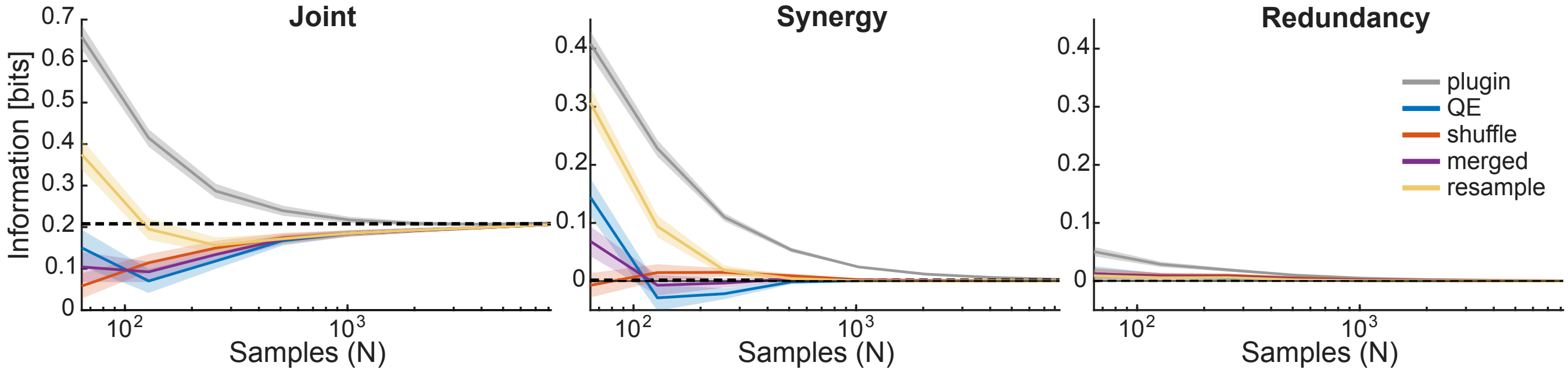

Joint
Synergy
Redundancy
Information [bits]
Samples (N)
plugin
QE
shuffle
merged
resample

Supplemental Figure Legends

Figure S1. Schematic of the Partial Information Decomposition with two sources. PID decomposes the joint information that the sources R_1 and R_2 carry about the target S into four components: the redundancy $Red(S : R_1; R_2)$, the two unique information terms $Unq(S : R_1 \setminus R_2)$ and $Unq(S : R_2 \setminus R_1)$, and the synergy $Syn(S : R_1; R_2)$. The colored outlines represent the four linear equations, Eq. (4–5) and (12–13) that relate the four PID components to Shannon information quantities.

Figure S2. Bias properties and bias corrections for the sum of unique information terms Plots show the residual bias (averaged bias-corrected empirical estimates from N samples minus ground-truth values) of the sum of the two unique information terms $Unq(S : R_1 \setminus R_2) + Unq(S : R_2 \setminus R_1)$ as a function of the number of samples N (left panels, on a log x-axis) and as a function of the number of parameters defining the probability distributions (right panels). A: Discrete BROJA PID simulations ($R = 4$, left; $N = 512$, right). Bias correction methods: plugin (no correction), QE, shuffle-subtraction, and merged. B: Gaussian gPID simulations ($d = 20$, left; $N = 128$, right). Bias correction methods: plugin (no correction), shuffle-subtraction, resampling, merged, and Venkatesh. Results in each panel are plotted as mean $\pm$ 3 SEM over $n = 100$ simulations. Dashed vertical lines: boundary of the sufficient sampling regime. Discrete and Gaussian simulations were performed under the bit-of-all scenario (see Methods and Table S2). For more details see Table S1.

Figure S3. Bias of the plugin PID values and residual bias of the bias-corrected PID values as a function of the number of parameters defining the probability distributions. Biases of plugin estimates and residual biases of bias-corrected estimates are computed as the difference between the averaged plugin (no correction) or bias-corrected information empirically measured from N samples and the corresponding ground-truth values. A–C: results for discrete BROJA PID. Bias correction methods: plugin (no correction), shuffle-subtraction, QE, and merged. D–F: results for Gaussian gPID. Bias correction methods: plugin (no correction), shuffle-subtraction, resample, merged, and Venkatesh. Left to right: joint information, synergy, and redundancy. Each row corresponds to a different number of simulated samples (indicated on the far left). Results in each panel show mean $\pm$ 3 SEM over $n = 100$ simulations. Dashed vertical lines: boundary of the sufficient sampling regime. In both cases, simulations were performed under the bit-of-all scenario (see Methods and Table S2). For more details see Table S1.

Figure S4. Dependence of the bias of the discrete BROJA PID on the number of samples N used to compute it. A: Joint information. B: Synergy. C: Redundancy. Each column corresponds to a different bias correction: plugin (no correction), QE, shuffle-subtraction, merged, and resample. Individual lines correspond to different values of the level of joint information, controlled by the simulation parameter α regulating the stimulus-response gain, with information level indicated by line color (lighter to darker colors indicate lower to higher information; see Methods). The x-axis is plotted on a logarithmic scale. We used $R = 4$ discretization bins for each neuron and $S = 4$ different stimulus values. Results in each panel are plotted as mean $\pm$ 3 SEM over $n = 100$ simulations. Dashed vertical lines: boundary of the sufficient sampling regime. Simulations were performed under the bit-of-all scenario (see Methods and Table S2). For more details see Table S1.

Figure S5. Bias correction performance for the discrete BROJA PID. Values of discrete joint information (left), synergy (middle), and redundancy (right) in four different simulated scenarios ('bit-of-all', 'uncorrelated', 'high redundancy', and 'high synergy'; see Methods and Table S2). Plots show the information values as a function of the number of samples N used to compute

them. The x-axis is plotted on a logarithmic scale. Bias correction methods: plugin (no correction), shuffle-subtraction, QE, merged, and resample. A: Results for the case of lower joint information ($\alpha = 7$). B: Results for the case of higher joint information ($\alpha = 10$). We used $R = 4$ discretization bins for each simulated neuron and $S = 4$ stimuli. Results in each panel are plotted as mean $\pm$ 3 SEM over $n = 100$ simulations. Dashed horizontal lines: ground truth information values computed from the true distributions. Dashed vertical lines: boundary of the sufficient sampling regime. For details see Methods and Tables S1 and S2.

Figure S6. Bias correction performance for the discrete I_{min}. Values of discrete joint information (left), synergy (middle), and redundancy (right) in four different simulated scenarios ('bit-of-all', 'uncorrelated', 'high redundancy', and 'high synergy'; see Methods and Table S2). Plots show the information values as a function of the number of samples N used to compute them. The x-axis is plotted on a logarithmic scale. Bias correction methods: plugin (no correction), shuffle-subtraction, QE, merged, and resample. A: Results for the case of lower joint information ($\alpha = 7$). B: Results for the case of higher joint information ($\alpha = 10$). We used $R = 4$ discretization bins for each simulated neuron and $S = 4$ stimuli. Results in each panel are plotted as mean $\pm$ 3 SEM over $n = 100$ simulations. Dashed horizontal lines: ground truth information values computed from the true distributions. Dashed vertical lines: boundary of the sufficient sampling regime. For details see Methods and Tables S1 and S2.

Figure S7. Bias correction performance for the discrete I_{MMI}. Values of discrete joint information (left), synergy (middle), and redundancy (right) in four different simulated scenarios ('bit-of-all', 'uncorrelated', 'high redundancy', and 'high synergy'; see Methods and Table S2). Plots show the information values as a function of the number of simulated trials (i.e. samples) N used to compute them. The x-axis is plotted on a logarithmic scale. Bias correction methods: plugin (no correction), shuffle-subtraction, QE, merged, and resample. A: Results for the case of lower joint information ($\alpha = 7$). B: Results for the case of higher joint information ($\alpha = 10$). We used $R = 4$ discretization bins for each simulated neuron and $S = 4$ stimuli. Results in each panel are plotted as mean $\pm$ 3 SEM over $n = 100$ simulations. Dashed horizontal lines: ground truth information values computed from the true distributions. Dashed vertical lines: boundary of the sufficient sampling regime. For details see Methods and Tables S1 and S2.

Figure S8. Residual bias across information levels. Plots show the residual bias of joint information (left), synergy (middle), and redundancy (right) as a function of the joint information level. Residual biases are computed as the difference between the averaged bias-corrected information empirically measured from N samples and the corresponding ground-truth values. A: Discrete BROJA PID simulations. We vary the level of joint information (plotted on the x-axis) by changing the value of the simulation parameter α regulating the stimulus-response gain (number of samples $N = 512$ and number of bins $R = S = 4$). Bias corrections used for information quantities: QE, shuffle-subtraction, and merged. B: Gaussian gPID simulations. We vary the level of joint information (plotted on the x-axis) by changing the simulation parameter α scaling the diagonal of the covariance matrix ($N = 256$ and number of sources and target dimensions $d = 20$). Bias corrections used for synergy and redundancy: resampling, shuffle-subtraction, merged, and Venkatesh. For joint information we used the Goodman correction in the resampling, shuffle-subtraction, and merged methods as it is an exact correction, and for the Venkatesh correction we used the Cai et al. correction to replicate what was done in Venkatesh et al.[S1]. We plot mean $\pm$ 3 SEM over $n = 100$ simulations. For both discrete and Gaussian PID, simulations were performed under the bit-of-all scenario (see Methods and Table S2). For more details see Table S1.

Figure S9. Bias and performance of bias corrections for the Gaussian gPID as a function

of the number of dimensions d. Biases of plugin (no correction) estimates and residual biases of bias-corrected estimates are computed as difference between the averaged plugin or bias-corrected information empirically measured from N samples and the corresponding ground-truth values. A: Joint information. B: Synergy. C: Redundancy. The far left column plots the bias of the plugin values. Each of the other columns plots the residual bias after application of the bias correction method listed in the panel title. Individual lines correspond to different values of the level of joint information, controlled by the simulation parameter α scaling the diagonal of the covariance matrix, with information level indicated by line color (lighter to darker colors indicate lower to higher information; see Methods; number of samples $N = 256$). Results in each panel are plotted as mean over $n = 100$ simulations. Dashed vertical lines: boundary of the sufficient sampling regime. Simulations were performed under the bit-of-all scenario (see Methods). For more details see Table S1.

Figure S10. Bias and performance of bias corrections for the Gaussian gPID as a function of the number of samples N used to compute them. The x-axis is plotted on a logarithmic scale. A: Joint information. B: Synergy. C: Redundancy. The far left column plots the plugin (no correction) information values. Each of the other columns plots the information values after application of the bias correction method listed in the panel title. Individual lines correspond to different values of the joint information, controlled by the simulation parameter α scaling the diagonal of the covariance matrix, with information level indicated by line color (lighter to darker colors indicate lower to higher information; see Methods). We set the dimensionality of the target and of the sources to $d = 20$. Results in each panel are plotted as mean over $n = 100$ simulations. Dashed vertical lines: boundary of the sufficient sampling regime. Simulations were performed under the bit-of-all scenario (see Methods). For more details see Table S1.

Figure S11. Bias corrections performance for gPID. Values of Gaussian joint information (left), synergy (middle), and redundancy (right) in four different simulated scenarios ('bit-of-all', 'high synergy', 'zero synergy', and 'both unique'; see Methods). Plots show the information values as a function of the number of samples N used to compute them. The x-axis is plotted on a logarithmic scale. Bias correction methods: plugin (no correction), resample, shuffle-subtraction, merged, and Venkatesh. A: Results for the case of lower joint information ($\alpha = 1.5$). B: Results for the case of higher joint information ($\alpha = 1$). We set the number of dimensions of the target and the sources to $d = 20$. Results in each panel are plotted as mean $\pm$ 3 SEM over $n = 100$ simulations. Dashed horizontal lines: ground truth information values computed from the true distributions. Dashed vertical lines: boundary of the sufficient sampling regime. For details see Methods and Table S1.

Figure S12. Comparison of the residual bias of the discrete merged correction using optimized and equal weighting. The residual bias (difference between the averaged values estimated from N samples and the corresponding ground-truth values) of discrete joint information (top row), synergy (middle row), and redundancy (bottom row) as a function of the number of samples N used to compute them. The x-axis is plotted on a logarithmic scale. Columns compare the merged estimator obtained using equal weighting of QE and shuffle-subtracted estimates (left) with the optimized weighted merged estimator (right), where weights were optimized depending on the joint information level. Individual lines correspond to different joint information levels, controlled by the simulation parameter α regulating the stimulus-response gain, with information level indicated by line color (lighter to darker colors indicate lower to higher information; see Methods). Simulations were performed using the BROJA PID under the bit-of-all discrete scenario (see Table S2) with $R = 4$ discretization bins per neuron and $S = 4$ stimulus values. Results in each panel are plotted as mean $\pm$ 3 SEM over $n = 100$ simulations. Dashed vertical lines: boundary of the sufficient sampling regime.

Figure S13. Using a Gaussian PID on discrete neural data. A: Example of stimulus-conditional probability distributions $p(r|s)$ of spike counts of one Poisson neuron whose spike counts follow a Poisson distribution with a given mean λ in response to different stimuli. The brown line denotes the Poisson distribution of the spike counts and the yellow line denotes a Gaussian with the same mean. B: Same as in panel A, but plotting the marginal (stimulus-unconditional) distribution $p(r)$ of the spike counts obtained assuming S uniformly distributed discrete stimuli. C: Discrete and Gaussian information estimates vs ground truth values of uncorrelated Poisson neuron pairs responding to $S = 2$ discrete stimuli (Eq. (26), $\beta = \gamma = 0$). Parameters α (information level) and B (background firing) were varied. GT indicates ground-truth values computed from the exact Poisson probabilities. Discretized and Gaussian (Eq. (21)) estimates are computed numerically from 10000 simulated samples per stimulus. Left: information values for each parameter combination. Right: for each parameter combination, ratios between estimated (discrete or Gaussian) and GT value. D and E: same as C, but neurons respond to $S = 4$ and $S = 12$ discrete stimuli, respectively. F: Bias corrected PID terms computed with the gPID from a discrete simulation ($\alpha = 10$, $B = 5$, $\beta = \gamma = 0$, Eq. (26)). G: Estimated joint information from the three real neural spiking datasets shown in Fig. 6A–C using the discrete shuffle-subtraction bias correction and the Gaussian plugin (no correction) estimator (mean $\pm$ 3 SEM across all neuron pairs). For more details see Table S1.

Figure S14. Comparison of analytical expressions of the bias for the Gaussian entropy, and evaluations of the residual mutual information bias. A: Bias of the entropy of a Gaussian multivariate distribution as a function of the number of samples N (x-axis on a logarithmic scale). Results computed with dimension $d = 10$ and $d = 20$ are plotted with blue and red colors, respectively. B: Bias of the entropy of a Gaussian multivariate distribution as a function of the dimension d computed with $N = 128$ (blue) and $N = 256$ (red) samples, respectively. The Gaussian process used in simulations for panels A–B had a diagonal covariance matrix, with zero mean and unit variance in each of the d dimensions. C–D: Difference between the numerically measured bias and the analytical bias computed with analytical expressions either from Cai et al. or from Goodman, plotted as a function of the number of samples N (C; with x-axis on a logarithmic scale) or the dimension d (D). The Gaussian process used in simulations for panels C–D was made of one Gaussian source and one Gaussian target of the specified dimension d with a diagonal covariance matrix and thus zero mutual information between the target and the source. Results in each panel are plotted as mean $\pm$ 3 SEM over $n = 100$ simulations. Solid line: numerical evaluation from simulated data. Squares: Goodman analytical formula. Triangles: Cai et al. analytical formula. For more details see Table S1.

Figure S15. Tolerance analysis performed on the merged-corrected residual synergy and redundancy bias and on the plugin synergy and redundancy bias. We fitted the absolute value of the residual merged bias or the plugin (no correction) bias values obtained from all data points within the sufficient sampling regime from simulations in Figs. S3A, S4, and S5 to quadratic functions of a quantity that captures the scaling of the leading term of the synergy bias according to theoretical considerations. This quantity (plotted on the x-axis of each panel) is R^3/N for the discrete BROJA PID (panel A) and $3d/N$ for the Gaussian gPID (panel B). In each panel we plot the averages $\pm$ SEM over all simulation cases ('bit-of-all', 'uncorrelated', 'high redundancy', 'high synergy') from Figs. S3A, S4, and S5 with low (blue) and high (red) joint information, respectively.

Figure S16. Comparison of PID bias corrections on each of the three full real neural datasets from Fig. 6A–C and on the same datasets each recomputed with half the trials. Each panel shows mean $\pm$ 3 SEM across all available simultaneously recorded neural pairs. A: mouse auditory cortex (dataset 1), $n = 6209$ pairs. B: mouse hippocampus (dataset 2),

$n = 36158$ pairs. C: monkey auditory cortex (dataset 3), $n = 83$ pairs. Left to right: schematic of each experiment; PID results with plugin (no correction), QE, shuffle-subtraction, and merged bias correction methods, respectively. Darker colors indicate quantities computed from the full datasets (all available trials); lighter colors indicate quantities recomputed with half the trials. *Image credits:* Panel A: mouse sketch adapted from doi.org/10.5281/zenodo.3925985, speaker icon from svgrepo.com, and music note icon from svgrepo.com; all modified and licensed under CC BY 4.0. Panel B: created by the authors. Panel C: Created in BioRender.

Figure S17. Further analyses of the human resting-state fMRI dataset (dataset 4). A: the joint information between the activity of a target set of ROIs and the activity of two source sets of ROIs shifted by one imaging time frame into the past. B: same as in panel A, for synergy. C: same as in panel A, for redundancy. We plot results as a function of dimension d (the number of ROIs within each group) of each multivariate source and target. We report Gaussian gPID estimates not bias-corrected (plugin) or bias-corrected with: resampling, shuffle-subtraction, merged, and Venkatesh. Left: results using 256 time-points (imaging volumes), corresponding to a typical size of an fMRI clinical resting-state dataset. Right: results using the entire dataset with all 1189 time-points available. Results in each panel are plotted as mean $\pm$ 3 SEM over $n = 100$ subjects.

Figure S18. Evaluation of the bias and the performance of bias correction methods when computing discrete BROJA PID of two 10-dimensional sources with an SVM dimensionality reduction. Each source is a different population of 10 uncorrelated Poisson neurons responding to $S = 4$ uniformly distributed stimuli ($\alpha = 1$, $\beta = \gamma = 0$; Eq. (26)). The 10-dimensional responses of each source are compressed to $R = 4$ discrete responses by using a multi-class linear SVM (2-fold cross-validation) to predict the value of the stimulus feature in each trial from the activity of each population. We plot joint information (left), synergy (middle), and redundancy (right) as a function of the number N of simulated trials. The x-axis is plotted on a logarithmic scale. Results in each panel are plotted as mean $\pm$ 3 SEM over $n = 100$ simulations. For more details see Table S1.

Figure	Support size	parameter
1A,B	R=4	N=100,1000
1C,D	d=5	N=20,50
2A	R=4 (left and right), R=2-9 (middle)	N=64-8192 (left),<br>N=512 (middle), N=64 (right)
2B	d=20 (left and right), R=1-42 (middle)	N=64-2048 (left),<br>N=128 (middle and right), $\alpha = 1$
3 top	R=4 (A and B)	N = 64-8192 (A and B),<br>N= 64-2048 (C)
3 bottom	R=2-9 (A and B), d=1-42 (C)	N=128
4A	R=4	N=64-8192
4B	d=20	N=64-2048
5A	R=2-9	N=512
5B	d=1-84	N=128
6,S16, S17	R = 3, S = 2 (A1 mouse),<br>R = 2, S = 12 (CA1),<br>R = 2, S = 399 (A1 monkey),<br>d = 20 (fMRI)	N = 140 (A1 mouse),<br>N = 870 (CA1),<br>N = 108 (A1 monkey),<br>N = 256 (fMRI)
7A	R=2-6	N=2ˆ10-2ˆ14
7B	d=1-15	N=2ˆ6-2ˆ10
8 top	R=3	N=[519-864]
8 bottom	$d_{sources} = [26, 47, 25]$, $d_{target} = 1$	N=[519-864]
S2A	R=4 (left), R=2-9 (right)	N=64-8192 (left),<br>N=512 (right)
S2B	d=20 (left), d=4-42 (right)	N=64-2048 (left),<br>N=128 (right), $\alpha = 1$
S3A, B, C	R = 2–9	N = 256 trials (Fig. S3A),<br>N = 512 trials (Fig. S3B),<br>N = 1024 trials (Fig. S3C)
S3D, E, F	d = 4-84	$\alpha = 1.5$, N = 64 trials (Fig. S3D),<br>N = 128 trials (Fig. S3E),<br>N = 256 trials (Fig. S3F)
S4,S5,S6,S7,S12, S15	R=4	N = 64-8192
S8A	R=4	N=512
S8B	d=20	N=256
S9	d=1-84	N=256
S10, S11	d=20	N=64-2048
S13	R = 4, S = 2 (Fig. S13C),<br>R = 4, S = 4 (Fig. S13D,F),<br>R = 4, S = 12 (Fig. S13E)	N = 20000 (Fig. S13C),<br>N = 40000 (Fig. S13D,F),<br>N = 120000 (Fig. S13E)<br>α = 1-10,<br>B = 1-10, β = 0, γ = 0
S14A,C	d=10,20	N=64-2048
S14B,D	d=1-42	N=128, 256
S18	population size = 10	N = 64–8192,<br>α = 1,<br>B = 10, β = 0, γ = 0

Table S1: **Summary of support sizes and simulation parameters used across all figures, including main text and supplementary figures**. Support size refers to the number of bins (discrete case) or dimensions (Gaussian case). Parameters include the number of trials and, for simulated data, the simulation parameters.

Scenario	Parameters
bit-of-all (discrete)	$B = 5$; $\beta = 0.25$; $\gamma = 17.5$; $\alpha = 0$ (zero info), $\alpha = 7$ (lower info), 10 (higher info)
Uncorrelated (discrete)	$B = 5$; $\beta = 0$; $\gamma = 0$; $\alpha = 0$ (zero info), $\alpha = 7$ (lower info), 10 (higher info)
High redundancy (discrete)	$B = 5$; $\beta = 0.4$; $\gamma = 2$; $\alpha = 0$ (zero info), $\alpha = 7$ (lower info), 10 (higher info)
High synergy (discrete)	$B = 5$; $\beta = 0.1$; $\gamma = 20$; $\alpha = 0$ (zero info), $\alpha = 7$ (lower info), 10 (higher info)
Gaussian Simulations	$\alpha = 10000$ (zero info), $\alpha = 1.5$ (lower info), 1 (higher info)

Table S2: **Overview of simulation parameters for simulated scenarios of discrete and Gaussian simulations**. The values reported in the table are used for Figures 2A, 2B, 3A, 3B, 4A, 5A, S3A-C, S5, S6, S7. For analyses of bias across information levels, we vary α from 1 to 15 in discrete simulations (see Figs. 2A, S4, S8, S12) and use values $\alpha =$1, 1.1, 1.2, 1.5, 2 and 10000 in Gaussian simulations (see Figs. 2B, S8 and S9). Gaussian simulation scenarios were implemented exactly as described in Sec. C.1 (p. 22) of[S1].

Supplementary Notes

Supplementary Note 1: Other discrete PID measures

$\boldsymbol{I_{min}}$. In the work introducing PID[S2], Williams and Beer proposed a measure of redundant information about S, called I_{min}, that for two source variables R_1 and R_2 is defined as follows:

$$Red(S : R_1; R_2) = \sum_{s \in S} p(s) \min_{R_i \in \{R_1, R_2\}} I(S = s; R_i) \tag{S1}$$

where $I(S = s; R_i)$ is the *specific information* that source R_i carries about a specific value of the target variable $s \in S$, and is defined as:

$$I(S = s; R_i) = \sum_{r_i \in R_i} p(r_i|s) \Big[\log \frac{p(s|r_i)}{p(s)} \Big] \tag{S2}$$

Intuitively, the redundant information computed as in Eq. (S1) quantifies redundancy as the similarity between R_1 and R_2 in discriminating individual values of the target S.

$\boldsymbol{I_{MMI}}$. Minimum mutual information I_{MMI} was introduced in[S3]. This measure is important since both I_{min} and BROJA measures reduce to I_{MMI} for Gaussian systems with an univariate target. This is possibly the simplest PID measure, defining the redundant information that R_1 and R_2 carry about S as follows:

$$Red(S : R_1; R_2) = \min\{I(S; R_1), I(S; R_2)\} \tag{S3}$$

I_{MMI} quantifies redundant information by directly comparing the amount of information encoded individually by each source, and results in at least one component of unique information always being null. (note that in this paper we also compute I_{MMI} with three source variables, and that the definition of MMI redundancy with three sources is provided in Methods).

Supplementary Note 2: Key properties of PID components for two source variables

In this subsection, we review useful key properties of PID measures. In their paper introducing PID for the first time, Williams and Beer[S2] proposed a set of properties that a measure of redundancy $Red(S : R_1, R_2)$ should satisfy. In the following years, other authors introduced new properties which they thought to be important for measures of PID components[S4–6].

Three particularly important properties of PID measures with two source variables are: non-negativity, symmetry and additivity[S1].

- Non-negativity: each PID component should be non-negative, i.e. $Red(S : R_1; R_2) \geq 0$, $Unq(S : R_1 \setminus R_2) \geq 0$, $Unq(S : R_2 \setminus R_1) \geq 0$ and $Syn(S : R_1; R_2) \geq 0$. This property is important since it allows interpreting PID components as fractions of the joint mutual information about the target S encoded by the source variables (i.e., the average reduction of the entropy of S obtained when measuring the two sources simultaneously). All three discrete BROJA, I_{min} and I_{MMI} measures satisfy non-negativity[S2,3,7].

- Symmetry: redundancy and synergy are symmetric under permutation of source variables, i.e.
$Red(S : R_1; R_2) = Red(S : R_2; R_1)$ and $Syn(S : R_1; R_2) = Syn(S : R_2; R_1)$. This property is important since also Shannon information quantities which can be decomposed in terms of redundancy and synergy (including the joint information and the co-information) are symmetric under reordering of R_1 and R_2. All three discrete BROJA, I_{min} and I_{MMI} measures satisfy symmetry[S2,3,7].

- Additivity: given two independent subsystems of random variables (S_1, X_1, Y_1) and (S_2, X_2, Y_2), then

$$\begin{aligned} Red((S_1, S_2) : (X_1, X_2); (Y_1, Y_2)) &= Red(S_1 : X_1; Y_1) + Red(S_2 : X_2; Y_2) \\ Syn((S_1, S_2) : (X_1, X_2); (Y_1, Y_2)) &= Syn(S_1 : X_1; Y_1) + Syn(S_2 : X_2; Y_2) \\ Unq((S_1, S_2) : (X_1, X_2) \setminus (Y_1, Y_2)) &= Unq(S_1 : X_1 \setminus Y_1) + Unq(S_2 : X_2 \setminus Y_2) \\ Unq((S_1, S_2) : (Y_1, Y_2) \setminus (X_1, X_2)) &= Unq(S_1 : Y_1 \setminus X_1) + Unq(S_2 : Y_2 \setminus X_2) \end{aligned} \tag{S4}$$

  This property is important since it guarantees that PID components of two independent subsystems can be computed independently and then summed together to obtain the PID components for the full system. Discrete BROJA satisfies additivity, while I_{min} and I_{MMI} do not[S5].

An additional property satisfied by all three discrete measures is the *pairwise marginals* property[S6], i.e. $Red(S : R_1, R_2)$, $Unq(S : R_1 \setminus R_2)$ and $Unq(S : R_2 \setminus R_1)$ only depend on pairwise marginal distributions between the sources and the target $p(s, r_1)$ and $p(s, r_2)$. This implies that all dependencies between the target S and the sources (R_1, R_2) that go beyond the dependency between the individual sources and the target are quantified as synergy. Even though the pairwise marginals is a fundamental property of the three PID measures, it has been questioned whether PID measures should always satisfy it[S6,8]. For I_{MMI} and I_{min}, which have been defined for more than two source variables, the non-negativity, symmetry and pairwise marginal properties hold for any number of sources.

Venkatesh and colleagues empirically showed, on simulated jointly-Gaussian data, that gPID appears to satisfy the same properties proved for the discrete BROJA decomposition measure[S1]. A formal proof of these properties for gPID is still lacking but would follow immediately by establishing the conjecture that, for every jointly Gaussian distribution $p(s, r_1, r_2)$ the distribution q^* that minimizes the BROJA objective in Eq. (19) is itself Gaussian[S1].

Supplementary Note 3: Illustration of the origin of the bias

Before detailing the procedures for correcting for the limited-sampling bias, we illustrate intuitively the origin of the limited-sampling bias in information calculations.

We first considered the bias for the discrete information calculation. We simulated two neural units (sources for the PID analysis, two spiking neurons in this example) that are completely uninformative about the stimulus (target for the PID analysis) (see Fig. 1A). In this simulation, both the joint and single-neuron information should be zero, but the values we obtain will be different from zero because of limited-sampling of the probabilities. Specifically, we simulated two neurons R_1 and R_2, each responding on each trial with a uniform marginal distribution of spike counts ranging from 1 to 4, regardless of which of $S = 2$ stimuli was presented. The neurons are also uncorrelated, so their joint distribution is uniform across all possible 16 joint responses, and equal between the two stimuli. Examples of marginal and joint stimulus-conditional response probability histograms sampled from a finite number of trials ($N = 100$ trials, that is 50 per stimulus in the top

row and $N = 1000$ trials (that is 500 per stimulus) in the bottom row respectively) are shown in Fig. 1A (responses to stimuli 1 and 2, respectively). Despite all the true response distributions being uniform across response bins and stimuli, a single instantiation of the probabilities from simulated data will have differences across stimuli that are not real but just due to random fluctuations. With smaller numbers of trials (Fig. 1A, top), we can appreciate that the random fluctuations that generate spurious information are larger for the joint probability (the few trials are spread over a larger set of possible responses $R = 16$) than for the marginal probabilities (the few trials are spread over a smaller set of responses $R = 4$). As a result of these spurious differences between stimulus-specific response distributions, the values of information computed from individual simulations with the limited number of trials (Fig. 1B, top) would not be distributed around the true value of zero bits, but around a spurious non-zero value (the bias). Because the redundancy is computed from marginal probabilities and the joint information and the synergy rely instead on the joint probabilities, the bias is smaller for redundancy than for synergy. With larger number of trials (Fig. 1A, bottom), the random fluctuations in the empirical probabilities get smaller (the single instantiation looks more uniform across stimuli, as it should be by ground-truth), and thus the bias is reduced (Fig. 1B, bottom).

We next considered the limited-sampling bias obtained when computing information with the Gaussian method (which assumes that the joint distribution of stimulus and target is a multivariate Gaussian). In this case, the joint information between target and the two sources is just a function of the covariance matrix $\Sigma(S, R_1, R_2)$, see Eq. (21). We can thus understand the limited-sampling bias of the Gaussian information as arising from limited-sampling fluctuations of the covariance matrix. We simulated two source Gaussian signals and one for the target, all of them with $d = 5$ dimensions (see Fig. 1C,D). These simulated data are independently distributed, both across different variables and across different dimensions within the same variable, with zero mean and unit variance. The true covariance matrix should be diagonal with no off-diagonal covariance and thus the information between the target and the single source and the joint information between the target and the two sources should be zero. Examples of empirical full covariance matrices of the stimulus and the neural response sampled from a finite number of trials (20 and 50 trials, respectively in Fig. 1C) show that, however, individual realizations of the covariance matrix sampled from the data show random off-diagonal structure which are treated by the information equation as relationship between the variables and thus give non-zero information values. Similarly to random fluctuations of joint probability values for the discrete case, the off-diagonal entries are larger in absolute value for smaller trial numbers (and would be smaller for marginal covariances, not shown, as they average random fluctuations along the values of the marginalized variable). Thus, each information calculation with a fixed number of trials N would give a positive information value, and thus the average of the information calculation over many realizations with N trials would be larger than zero (rather than equal to zero as the ground truth value should be). Hence the information calculation suffers from an upward limited-sampling bias, which becomes larger for smaller numbers of trials N. Again, because the redundancy and single neuron information depends only on the marginal covariances and the joint information depend on the full joint covariance, the bias of the redundancy is smaller than that of synergy and joint information (Fig. 1D).

Supplementary Note 4: Further results of simulations and bias corrections

Here we present and collect all additional analyses of simulated data reporting the bias properties of the PID including results of the PID bias correction methods on simulated data. In Fig. S3 we show further details of how the bias of the discrete PID varies with R.

In Figs. S4 and S8 we show further details of how the bias of the discrete PID varies for

different information levels.

In Fig. S5 we show further results for simulated scenarios with some discrete PID components having a null ground-truth value.

In Figs. S6, S7 we show how the bias of two different discrete PIDs (I_{min} and I_{MMI}) scales with the number of trials and we confirm the effectiveness of our bias correction procedures with these PIDs.

In Figs. S9 and S10 we show details of how the Gaussian bias varies with d and N.

In Fig. S11 we show further results for simulated scenarios with some Gaussian PID components having a null ground-truth value.

Supplementary Note 5: Application of Gaussian PID to simulated spike count data

In this section, we test with both real data and simulations whether a Gaussian PID can be used for the analysis of spike counts in response to discrete uniformly distributed stimuli. We conclude that using the Gaussian PID on such data is not recommended, as it can give a large underestimation of the information values as well as non-credible synergy and redundancy attribution. In contrast, the discrete PID gives more robust estimates of the information values and credible attribution of synergy and redundancy. We thus recommend the use of the discrete PID for these types of data.

Gaussian PIDs[S1,3] are convenient because they allow analyses of high multivariate responses with relatively low trial numbers. However, they rely on the assumption that sources and targets are distributed according to multivariate Gaussian probabilities. Here we investigated whether these are generally applicable to typical experiments recording the spiking activity of neurons in response to discrete classes of stimuli with uniform distribution of the experimentally presented stimuli, which corresponds to a major class of experimental data. We will investigate this first by simulations under these conditions and then by analyzing real experiments with spiking activity.

It is well documented that the number of spikes emitted by a neuron in response to different stimuli follows approximately a Poisson distribution[S9]. Moreover, the time windows in which neurons process and transmit information are usually short, often of only few tens of milliseconds[S10]. Given that the firing rate of cortical neurons ranges from one or a few spikes per second (when considering the less effective stimuli) to several tens of spikes per second (when considering the most effective stimuli), firing rate distributions relevant for neural information processing can be conceptualized as near-Poisson mean spike counts not exceeding a few spikes. We will thus examine applications of Gaussian PID to simulated neural firing under those conditions.

To explain the effect of using Gaussian approximations on neural data, it is easier to express mutual information as the difference between response entropy $H(R_1, R_2)$ (function of $p(r_1, r_2)$) and noise entropy $H(R_1, R_2|S)$ (function of $p(r_1, r_2|s)$):

$$I(S; R_1, R_2) \quad = -H(R_1, R_2|S) + H(R_1, R_2) \tag{S5}$$

Errors in estimating information with the Gaussian PID will arise from errors in approximating the response probability with Gaussians. Because the Gaussian distribution is the one with the highest entropy across all distributions with a given variance[S11], replacing a true underlying non-Gaussian distribution with a Gaussian with the same mean and variance will overestimate the corresponding entropy. When the Poisson process has a relatively low mean spike count, the probabilities of neural responses conditional to the stimuli $p(r_1, r_2|s)$ are not well approximated by Gaussian distributions (Fig. S13A). This may result in an overestimation of $H(R_1, R_2|S)$ when using a Gaussian. When pooling together responses to individual stimuli using a uniform probability of stimulus presentation to form a marginal probability of responses independent of the stimuli $p(r_1, r_2)$, this probability will differ from a Gaussian, especially for larger values of the

number of uniformly distributed stimuli S (Fig. S13B). This will lead to a large overestimation of $H(R_1, R_2)$. All these considerations are illustrated in Fig. S13A,B with a cartoon of the stimulus-conditional and stimulus unconditional probability distributions of an individual neuron, and similar considerations apply to joint distributions.

To test quantitatively the errors introduced by approximating the discrete distributions with Gaussians on the mutual information estimation (which combines the effects of estimation errors on response and noise entropy), we ran a parameter sweep of a simulation with two uncorrelated Poisson neurons, as described in the 'uncorrelated' scenario of the discrete simulations (see Methods and Table S2). Stimuli were presented with a uniform distribution, as it is often the case with experimental design when using discrete classes of stimuli. We varied the number of stimuli S from 2 to 12 to investigate the effect of this experimental design parameter.

We computed the ground-truth information values from exact Poisson probabilities of spike counts that we used to generate the data. We then compared the information values obtained with the two approaches that can be applied to real data in which the probabilities are unknown. The first is the discrete approach, the one at the basis of the discrete PID. We discretized the responses into $R = 4$ equipopulated bins (as it could be done reliably also in experiments with more limited numbers of trials). In the second approach, we used the single-neuron mutual information with the Gaussian formula (Eq. (20)), which fits a multidimensional Gaussian to the joint distribution of target and sources.

In Fig. S13C-E we plot the values of the ground-truth information as well as those obtained with the discrete and the Gaussian information approach. We simulated neural responses across different values of simulation parameters α regulating the difference in firing rates across stimuli (the higher α, the larger the information) and B regulating the background stimulus unrelated firing (the larger B, the smaller the information). We varied α and B independently from 1 to 10, both in steps of 1. We computed information using a large number (10000) of simulated trials per stimulus to avoid bias issues. For $S = 2$ equiprobable stimuli the Gaussian procedure gave results reasonably close to the ground truth value, but for $S = 4$ and $S = 12$ it severely underestimated the ground truth information. The Gaussian information values were only $\approx 50\%$ and $\approx 20\%$ of the ground-truth values for $S = 4$ and $S = 12$ stimuli, respectively. In contrast, the discrete PID computed with $R = 4$ response bins provided a good approximation to the ground truth information across a wide range of parameters and values of S (the discretized information values were $\approx 90 - 100\%$ of the ground-truth value, with the small information loss due to the data processing inequality as some different spike counts are grouped together when discretizing into $R = 4$ bins).

In addition to checking whether the joint information values are accurate, we also studied whether the results of the PID decomposition were credible. In our simulations, with each neuron uncorrelated with the other and each neuron encoding a different and independent binary stimulus, synergy and redundancy must be zero. We verified that the discretized PID BROJA indeed gave zero synergy and redundancy in these cases (see Fig. S5 case 'Uncorr'). However, when we applied the Gaussian PID to these uncorrelated Poisson simulated data we found high synergy despite the absence of correlations (results are plotted for the $S = 4$ case in Fig. S13F, but we verified that similar results held for $S = 2$ and $S = 12$, not shown). Additionally we found that the unique information of neuron 1 was considerably larger than that of neuron 2, although both neurons were simulated with identical information levels and properties. These not-credible PID decomposition values were not due to numerical errors due to limited sampling as the results were stable across trials, and are thus a consequence of fitting a probability model (the Multivariate Gaussian) to data that are not well described by this parametric distribution. Moreover, we verified that whenever we used uncorrelated processes generated with a true multivariate Gaussian distribution we had zero redundancy and synergy with the gPID (see also[S1] and Fig. S11 case 'both unique').

Finally, to test the Gaussian PID on real spiking data, we tested it on the three spiking datasets. We computed the joint information between one target (stimulus) and two sources (activity of two neurons). As predicted by simulations, we find an excellent approximation to the discrete values for dataset 1 in which $S = 2$, but very poor performance and major underestimations for the datasets with larger S. For dataset 2 (which had $S = 12$), we had a severe underestimation (the Gaussian information was 20% of the discrete one) and for dataset 3 (which had $S = 399$) we had an even poorer underestimation of the discrete information (the Gaussian information was 5% of the discrete one).

Supplementary Note 6: Analytical considerations on the bias of discrete and Gaussian PIDs

Here we describe analytical calculations that account for the bias properties of Shannon information and for the fact that for both discrete and Gaussian PID the synergy suffers from an upward bias larger than that of the redundancy.

Analytical considerations for the discrete PID using an asymptotic expansion

We consider first the discrete PID.

Suppose that we compute the entropy in a certain discrete space X (in the applications of this paper, this space will either be the joint space of pairwise sources and target, or the marginal space of a subset of these quantities) having at our disposal only a finite number N of trials. The entropy of this probability distribution is defined (in bits) as:

$$H(X) = -\sum_{x} p(x) \log_2(p(x)) \tag{S6}$$

The true values of the probabilities are not known a priori in experimental situations. The probabilities could be perfectly computed from the data if we had infinite amounts of experimental trials, but here we consider the setting in which we have only a finite number of trials N. If we have an empirical estimate of the probabilities as the empirical occurrences $p_N(x)$ in the N available trials, we compute the value of the entropy H_N obtained with a finite number of trials N:

$$H_N(X) = -\sum_{x} p_N(x) \log_2(p_N(x)) \tag{S7}$$

From this, we can compute the finite sampling bias of the entropy, defined as the difference between the average value $\langle H_N \rangle$ of H_N over all instantiations with N trials and the true value H obtained from the ground-truth distributions.

Analytical approximations to the bias have been derived[S12–14] in the large N limit, defined formally as the case when $N \cdot p(x) \gg 1$. In our applications, this would translate to $N \cdot p(r_1, r_2, s) \gg 1$ for each target value s and joint source value r_1, r_2. If this is satisfied for the joint distribution of the three variables then it is also satisfied for the marginal distributions, e.g. $N \cdot p(r_1, r_2) \gg 1$ for each joint response r_1, r_2, and $N \cdot p(r_i, s) \gg 1$ (i = 1,2). While it is unrealistic that this large N limit case will be formally satisfied in practical experiments, this type of derivation has proven very useful to provide approximate expressions of the bias of Shannon information (which are quantitatively precise if N is large enough but are still qualitatively useful for intermediate values of N), to understand its properties and to design strategies to correct for it[S12,13,15]. This previous work has reported an expansion of the bias of the entropy in powers of $1/N$

$$\langle H_N \rangle - H = \frac{C_1}{N} + \frac{C_2}{N^2} + \cdots \tag{S8}$$

with the leading term $1/N$ in the bias being

$$\frac{C_1}{N} = -\frac{(R_x - 1)}{2N\ln(2)} \tag{S9}$$

where R_x denotes the number of relevant events (called responses here with applications to neural activity in mind) for the considered distribution, defined as the number of responses x with probability $p(x) > 0$[S13,16]. Thus, although the bias is not the same for all probability distributions, in the asymptotic sampling regime, its leading term depends on a remarkably simple detail of the response distribution: the number of trials and the number of relevant responses. This makes it possible to derive simple rules of thumb for estimating the bias magnitude and compare the relative bias in different situations. As we will discuss now, this equation can predict effectively how the bias differs across PID terms depending on the number of discrete bins of sources and targets and on the overall information level.

The leading bias terms of the joint information can be computed by writing information as sums and differences between three entropies (Eq. (11)). Using the above leading term for the bias of each entropy term in (Eq. (11)) we obtain the following expression for the bias of the joint information:

$$\langle I_N(S; R_1, R_2)\rangle - I(S; R_1, R_2) = \frac{1}{2N\ln(2)}\left[(\tilde{R}_{s,r_1,r_2} - 1) - (\tilde{R}_{r_1,r_2} - 1) - (S-1)\right] \tag{S10}$$

where in the above $\langle I_N(S; R_1, R_2)\rangle$ denotes the average value of the joint information estimated with a finite number of trials N, taken over all instantiations with N trials. $\tilde{R}_{s,r_1,r_2}$ is the number of bins with non-zero joint probability $p(s, r_1, r_2)$, $\tilde{R}_{r_1,r_2}$ is the number of bins with non-zero marginal probability $p(r_1, r_2)$ and S is the number of values of the target variable (as in most experimental designs, the target variable is the experimentally-presented stimulus, for obvious reasons we assume with this notation that each presented stimulus has a non-zero probability of being presented). Because the absolute value of the entropy bias increases with the number of possible responses, the biggest contribution to the bias of the joint information $I(S; R_1, R_2)$ (Eq. (11)) comes from the bias of the joint entropy of target and two sources $H(S, R_1, R_2)$, whose bias originates from finite sampling fluctuations of the joint probability $p(s, r_1, r_2)$ and thus has a proportionality coefficient of $\tilde{R}_{s,r_1,r_2}$. If all or most bins have non-zero probability, then $\tilde{R}_{s,r_1,r_2} \approx S \times R \times R$.

The leading term of the bias of the single cell information (Eq. (10)) comes instead from $H(S, R_i)$, which depends on the marginal distribution $p(r_i, s)$ of target and one source. The finite sampling fluctuations of the marginal probabilities (Fig. 1A) are smaller than the ones of the joint probabilities and thus its bias has a proportionality coefficient of $\tilde{R}_{s,r_i}$ which scales as $\approx S \times R$ if all or most bins have non-zero probability. Thus, the bias of the single cells is approximately R times smaller than that of the joint information and its leading term in $1/N$ is as follows:

$$\langle I_N(S; R_i)\rangle - I(S; R_i) = \frac{1}{2N\ln(2)}\left[(\tilde{R}_{s,r_i} - 1) - (\tilde{R}_{r_i} - 1) - (S-1)\right] \tag{S11}$$

where $\langle\cdot\rangle$ denotes the average over all instantiations of $I_N(S; R_i)$ computed with N trials.

Importantly, the analytical expression can also explain why the bias of the zero-information shuffled data (and in general of the bias with lower information levels) is larger than the bias of the original distribution and give an idea of the situations in which they are expected to be tight[S13]. Take for example the joint information, for which the bias is mostly dictated by the number of relevant response bins $\tilde{R}_{s,r_1,r_2}$ of the joint distribution. Informative cases will have stimulus-specific distributions that are restricted to a smaller number of relevant bins than the total number of bins (because informative cases have less variability at fixed stimulus so more restricted stimulus-specific distributions) and also the bins that are relevant for a specific stimulus

would not all be relevant for other stimulus-specific distributions (because informative cases will also show diversity of response distributions to different stimuli). When we randomly shuffle the trials combining trials from different stimuli, the shuffling operation will mix up responses and thus create larger stimulus-specific distributions (larger numbers of stimulus-specific relevant bins) which will then increase the bias. This prediction is confirmed by all our simulations, which show larger finite-sampling bias for lower information levels in discrete Shannon information estimates (Figs. 1B, S4, S5). This result is important because it implies that bias corrections based on subtracting shuffled estimators lead to a residual downward bias, which is useful to produce lower-bounded or conservative estimates. It also implies that the shuffled-subtracted bias corrected estimates will be precise and not too conservative when the information level in the original data is low (because in this case the number of relevant bins of the shuffled and original data will be similar) and it will be too conservative when the information levels in the data are high (because in this case the number of relevant bins of the shuffled distribution will be much larger than the corresponding one for the original distribution). This leads to the design of tighter downward biased estimators of information that may be helpful to draw conservative yet sufficiently accurate conclusions.

Having understood the bias of Shannon information measures relevant for PID, we now use this knowledge to evaluate the bias of the PID terms. We focused first on the bias of the synergy as defined in I_{MMI}. We remind that the synergy is defined as a difference between the joint and the Union information (Eq.7), with the I_{MMI} Union information defined as follows:

$$Union = \max(I(S;R_1), I(S;R_2)) \tag{S12}$$

Thus, the bias of the Union information equals the bias (Eq. (S11)) of the single source information $(I(S;R_i))$ with the highest plugin information value of the two single-source information. The bias of the synergy is the difference between the bias of the joint information, which scales as $(S \times R \times R)/N$, and the one of the single-source information which scales as $(S \times R)/N$ (thus R times smaller than the bias of the joint information). Thus the bias of the synergy will be positive and scaling as $(S \times R \times R)/N$. This prediction is confirmed in all numerical simulations with I_{MMI}(Fig. S7).

We then repeated this approach but focusing on the bias of the synergy as defined in BROJA. The BROJA Union information is defined as the result of an optimization problem, minimizing the joint information $I_q(S;R_1,R_2)$ over the set Δ_P of all joint probability distributions $q(s,r_1,r_2)$ that have the same pairwise marginals $q(s,r_1) = p(s,r_1)$ and $q(s,r_2) = p(s,r_2)$ as the original distribution $p(s,r_1,r_2)$ (Eq. (6)). When applied to limited data N, the minimization within the Δ_P space will tend to select a minimum information within the space set by the marginals $p_N(r_1,s)$ and $p_N(r_2,s)$ computed with N trials. It will thus select probability distributions with a low finite sampling information value. Because this bias is by and large positive, we expect that the minimization procedure will select probabilities with lower bias. We therefore conjecture that the distribution q^* minimizing the mutual information over the Δ_P space has a bias similar to the one obtained from the probability distribution $p_{ind}(r_1,r_2,s)$ defined directly from the marginals such that the neural units would have the same single unit probabilities but no interactions at fixed stimulus (stimulus-conditional independence, or lack of noise correlation as it is termed in the neural literature[S17,18])

$$p_{ind}(r_1,r_2,s) = p(r_1|s)p(r_2|s)p(s) \tag{S13}$$

We call the I_q computed on $q = p_{ind}$ the conditionally-independent information $I_{ind}(S;R_1,R_2)$, defined as the joint mutual information with stimulus-conditional probabilities $p_{ind}(r_1,r_2|s)$ and stimulus unconditional probabilities $p_{ind}(r_1,r_2) = \sum_s p_{ind}(r_1,r_2,s)$. For clarity, the conditionally-independent information is the information carried by the population about the target if the single neural unit responses were the same as the original data but there were no noise correlations

(no target-conditional interactions between the neural units), and it has been used as a reference distribution for understanding whether noise correlations increase or decrease information (see[S19,20]). $I_{ind}(S;R_1,R_2)$ can be expressed in terms of entropies as:

$$I_{ind}(S;R_1,R_2) = -H(S,R_1) - H(S,R_2) + H_{ind}(R_1,R_2) + 2H(S) \tag{S14}$$

Importantly for our PID bias understanding, in I_{ind}, compared to the joint information, we replace the entropy of the two sources and the target $H(S,R_1,R_2)$, whose bias scales as $(R^2 \times S)/N$, with the term $H(S,R_1) + H(S,R_2)$, whose bias scales as $(R \times S)/N$ which is R times smaller. Thus, the bias of the joint information is larger than and grows faster with R than the bias of $I_{ind}(S;R_1,R_2)$. Because the minimal set of finite sampling variations in the Δ_P space is set by the finite sampling fluctuations of the marginals, we assume that the minimization in the Union calculation will select distributions roughly with the same bias as $I_{ind}(S;R_1,R_2)$. To test this, we performed simulations of pairs of neural units. We found (see Fig. 3) across all conditions and trial numbers that the bias of the Union information is very similar to the bias of $I_{ind}(S;R_1,R_2)$. It is actually slightly lower, which is compatible with the understanding that the minimum selects a similar bias and of course smaller statistical fluctuations. The synergy is the difference between the joint and the Union information. Similarly to the cases of the MMI described above, the bias of the joint information is the largest of the two and scales as $(S \times R \times R)/N$, whereas the bias of the Union information is smaller and scales as $(S \times R)/N$. Thus, again, the bias of the synergy will be positive and scale as $(S \times R \times R)/N$ when R increases or N decreases. Because the biggest bias term of the synergy comes from the joint information, which decreases with the information level, the bias of the synergy should decrease with the information level. The above predictions are confirmed in all numerical simulations (Figs. 1B, 2A, S3A-C, S4, S5).

The redundancy is the difference between the sum of single source information and the Union information. The bias of the redundancy is thus a difference of two terms that scale as $(S \times R)/N$. These terms may partly cancel out and none of the two scales with R as fast as the bias of the joint information, which is R times larger. Therefore redundancy is expected to be less biased than synergy, with the gap between the two biases expected to increase steeply as R increases. Once again, these predictions are confirmed in all numerical simulations with both BROJA and I_{MMI} (Figs. 1B, 2A, S3A-C, S4, S5, S7).

While we derived these bias properties for the BROJA and I_{MMI} PID, we expect that the same conclusions would hold for I_{min} and we verified with simulations that these predictions indeed hold with I_{min} (Fig. S6). In fact also for I_{min} the Union information depends on the two marginal probabilities $p(r_1,s)$ and $p(r_2,s)$ because they satisfy the so-called pairwise marginals property. We expect that all the considerations we made about the relative size of the bias of synergy and redundancy hold for any definition of PID satisfying the pairwise marginals property, because in this case our key reasoning (that Union information has bias determined by the finite size fluctuations of the marginals) would hold true.

In sum, the above explains why synergy has a large positive bias, larger than the one of redundancy, and it explains why the bias of synergy gets proportionally bigger for large numbers of possible discretized single cell responses.

Analytical considerations for the Gaussian PID

We consider a multivariate Gaussian variable X with dimensionality d_X and covariance matrix $\Sigma(X)$. The Shannon entropy of this multivariate Gaussian can be expressed as a function of the covariance matrix and of the dimensionality, as follows:

$$H(X) = \frac{1}{2\ln(2)}\ln(\det(2\pi e\Sigma(X))) = \frac{1}{2\ln(2)}(d_X \cdot (1+\ln(2\pi)) + \ln(\det(\Sigma(X)))) \tag{S15}$$

As for the discrete case, we consider the setting in which the Gaussian PID and thus the covariance matrix $\Sigma(X)$ are to be estimated by N available trials. The sampled covariance matrix $\Sigma_N(X)$ estimated from the data will have statistical fluctuations from one random realization with N trials to another (Fig. 1C). It will be on average over all instantiations with N trials, equal to the true one. Since the log-determinant is a nonlinear concave function, the average across realizations of the log-determinant of the sampled covariance matrix will be lower or equal to the log-determinant of the average sampled covariance matrix. As a result, the entropy will have a downward bias. This bias has been computed analytically by Goodman[S21] and it takes the form:

$$\mathrm{Bias}\big[H(X)\big] = \frac{1}{2\ln(2)}\left(\langle \ln\det(\Sigma_N)\rangle - \ln\det(\Sigma)\right) = \frac{1}{2\ln(2)}\left(d_X\,\ln\Big(\frac{2}{N-1}\Big) + \sum_{k=1}^{d_X}\psi\Big(\frac{N-k}{2}\Big)\right) \tag{S16}$$

where $\psi(x)$ is the digamma function and $\langle \ln\det(\Sigma_N)\rangle$ indicates the average value of the log-determinant of the sampled covariance matrix taken over all random instantiations with N trials. Another study[S22] provided a more compact, closed-form approximation for this bias in terms of logarithms, namely:

$$\mathrm{Bias}\big[H(X)\big] = \frac{1}{2\ln(2)}\sum_{k=1}^{d_X}\ln\Big(1-\frac{k}{N}\Big) \tag{S17}$$

which is obtained by approximating the digamma function as a logarithm. These two analytical expressions of the Gaussian entropy show that the bias depends only on the sample size N and the dimensionality d_X of the Gaussian variable, not on the structure of the covariance matrix $\Sigma(X)$. In our numerical bias corrections we used the analytical expression of Eq. (S16) bias equation, because it is an exact formula that works slightly better than the approximate one provided by Cai and colleagues (see Fig. S14).

Importantly, the entropy bias is a negative function which decreases in absolute value when increasing N and increases in absolute value when increasing d_X. This is known from the properties of the digamma functions and can be easily appreciated by plots of their values (Fig. S14). Note that the absolute value of the bias increases steeply and supralinearly with d_X at fixed N. Thus the bias of the entropy of a variable with e.g. $2d_X$ dimensions would be more than twice larger in absolute value than the bias of a variable with d_X dimensions (Fig. S14). For example the bias of $H(S, R_1, R_2)$ would be more than three times larger than that of $H(R_1)$, which has three times fewer dimensions.

To understand the bias of the Gaussian information quantities and of the PID components, for simplicity, we assume that both Gaussian sources and the Gaussian target have all the same dimensionality d. We now recall that the joint information between the two sources R_1 and R_2 about the target S can be written as a function of three entropy terms (Eq. (11)). Using Eq. (S16) this leads to an expression of the bias of $I(S; R_1, R_2)$ only in terms of d and N:

$$\text{bias}[I(S;R_1,R_2)] = \frac{1}{2\ln(2)}\left(-\sum_{k=1}^{3d}\psi\left(\frac{N-k}{2}\right) + \sum_{k=1}^{2d}\psi\left(\frac{N-k}{2}\right) + \sum_{k=1}^{d}\psi\left(\frac{N-k}{2}\right)\right) \tag{S18}$$

Plots of the bias of $I(S; R_1, R_2)$ show that its bias is positive because, as for the discrete case, the largest contribution to the bias among the three entropies (Eq. (11)) comes from $-H(S, R_1, R_2)$ whose bias originates from finite sampling fluctuations of the joint probability $p(s, r_1, r_2)$ with covariance matrix $\Sigma(S, R_1, R_2)$ (Eq. (9)). The bias of $I(S; R_1, R_2)$ increases steeply with d and decreases with N (Fig. 2B).

Instead, the single cell information (Eq. (10)) has the biggest bias term coming from $H(S, R_i)$, which depends on the marginal distribution $p(r_i, s)$ of target and one source and thus from the

covariance matrix $\Sigma(S, R_i)$. Using Eq. (S16), the single cell information has the following bias:

$$\text{bias}[I(S; R_i)] = \frac{1}{2\ln(2)}\left(-\sum_{k=1}^{2d}\psi\left(\frac{N-k}{2}\right) + 2\sum_{k=1}^{d}\psi\left(\frac{N-k}{2}\right)\right) \tag{S19}$$

which has the largest contribution from a term with sums of digamma functions up to $2d$ and thus is smaller than the bias of $I(S; R_1, R_2)$ which has the highest contribution from sums of digamma functions up to $3d$. The intuitive reason why the bias is smaller is that finite sampling fluctuations of the marginal probabilities and of the marginal covariance matrices are smaller than the ones of the joint probabilities.

We then considered the bias of the Gaussian PID terms. We first focused on the synergy bias (which is the difference between the joint and the Union information bias) defined in I_{MMI}. The bias of the joint information was discussed above and is dominated by the sum of digamma functions up to $3d$. Because the Union information of I_{MMI} is defined as the maximum between the two single source information (Eq. (S12)), its bias equals the bias of the single source information $(I(S; R_i))$ with the highest information value. In the Gaussian case, both have the same bias, and thus the bias of the Union MMI information equals the one of single source information defined in Eq. (S19). Because the former has a larger positive bias dominated by the sum of digamma functions with terms up to $3d$ and the latter has a smaller positive bias whose larger term is a sum of digamma functions with terms up to $2d$, the synergy has a positive bias that increases steeply with d and decreases with N.

We then repeat this approach but focusing on the bias of the synergy with gPID[S1] (the Gaussian equivalent of BROJA). As for the discrete BROJA case, for the same reasoning we can approximate the bias of the gPID Union information with the one of the conditionally-independent joint information, $I_{ind}(S; R_1, R_2)$, built from $p_{ind}(s, r_1, r_2)$. The fact that the gPID Union information bias can be well approximated by the I_{ind} bias is numerically verified with high accuracy in the simulations (Fig. 3C). Because of the constraints used to construct the independent covariance matrix $\Sigma_{ind}(S, R_1, R_2)$ (see Methods), this covariance matrix has fewer parameters than the full covariance matrix used for the joint information, which suggests that the information $I_{ind}(S; R_1, R_2)$ is easier to sample with finite data. The information $I_{ind}(S; R_1, R_2)$ is obtained by replacing in Eq. (21) the full covariance matrix with the independent one $\Sigma_{ind}(S, R_1, R_2)$. For the sake of understanding its bias, it is useful to decompose $I_{ind}(S; R_1, R_2)$ into a sum of entropies as follows:

$$\begin{aligned} I_{ind} &= -H_{ind}(S, R_1, R_2) + H_{ind}(R_1, R_2) + H(S) = \\ &= -H(S, R_1) - H(S, R_2) + H_{ind}(R_1, R_2) + 2H(S) \end{aligned} \tag{S20}$$

The largest term of the bias of $I_{ind}(S; R_1, R_2)$ is due to $H_{ind}(S, R_1, R_2) = H(S, R_1) + H(S, R_2) - H(S)$ which has a smaller bias than the corresponding entropy $H(S, R_1, R_2)$ entering the synergy and joint information, because the bias of $H_{ind}(S, R_1, R_2)$ has contributions from terms up to dimensionality $2d$ whereas the bias of $H(S, R_1, R_2)$ is larger because it has contributions up to terms of dimensionality $3d$:

$$\text{bias}[H(S, R_1, R_2)] = \frac{1}{2\ln(2)}\left(3d\ln\left(\frac{2}{N-1}\right) + \sum_{k=1}^{3d}\psi\left(\frac{N-k}{2}\right)\right) \tag{S21}$$

$$\text{bias}[H_{\text{ind}}(S, R_1, R_2)] = \frac{1}{2\ln(2)}\left(3d\ln\left(\frac{2}{N-1}\right) + 2\sum_{k=1}^{2d}\psi\left(\frac{N-k}{2}\right) - \sum_{k=1}^{d}\psi\left(\frac{N-k}{2}\right)\right) \tag{S22}$$

Because the bias of the synergy is a difference of the larger bias of the joint information and the smaller of the Union information, and because the difference between the two increases

monotonically with d, the bias of the synergy is positive and grows steeply with d. Again, this is numerically well verified in all simulated cases (Figs. 1C,D, 2B, S3D-F, S9, S10, S11).

The redundancy is the difference between the sum of single neuron information and the Union information. For both MMI and gPID, its bias is a difference of terms that are as biased as the single neuron information, which is expressed by a sum of digamma functions with up to $2d$ terms (rather than $3d$ terms as for the dominant term of the synergy bias). Redundancy is thus less biased than the synergy for large d or small N.

Supplementary Note 7: Study of how to scale up calculations and bias corrections of discrete PID using dimensionality reduction

In this section we report a preliminary investigation of how discrete PID calculations may be scaled up to become feasible and unbiased even with higher dimensionality of the sources (for neurons, for example with large neuronal populations).

We simulate two independent populations of 10 uncorrelated Poisson neurons, responding to four different stimuli with uniform distribution (Eq. (26) with parameters $\alpha = 1$, $B = 10$, $\beta = 0$, $\gamma = 0$). These stimuli represent two binary features, with each pool selectively encoding one of the two features. The two neural pools serve as independent 10-dimensional sources in the Partial Information Decomposition (PID) analysis. Because the two populations are uncorrelated and each population represents only one of the two independent binary stimulus features s_1 and s_2 (see Methods), the PID of the information about the stimulus (the target) and the activity of the two sources (adding up to a total population of 20 neurons) must have zero synergy or redundancy across neurons.

The discrete PID with two 10-dimensional sources cannot be performed directly with reasonably sized datasets. Assuming we discretize each neuron in 4 bins each, we would have that the number of possible discrete values of each source would be $R = 4^{10}$, which would require $N > 4^{10} \times 4^{10} \times 4 \times 4$ trials for the sufficient sampling regime, a number that far exceeds what is feasible experimentally. We thus reduced the dimensionality of each source to four possible discrete responses, by training a one-vs-one multiclass linear SVM (implemented using the MINT toolbox[S23], regularization parameter $C = 1$) to predict the value of the stimulus in a trial from the activity of each source made of a population of 10 neurons. (For this SVM analysis, we used a 2-fold cross-validation, ensuring that the trials were identically split across sources). Reducing the dimensionality of different populations of neurons with SVM to study the effect on information coding of the interactions between neurons is well established in neuroscience[S23–25], although to our knowledge the coupling of SVM to PID in the way described here is a novelty of our approach. These reduced neural representation data of each population are then fed as the value of the source in each trial to the PID routine. Encouragingly, we found (Fig. S18) that when applying our three discrete PID bias correction methods (QE, shuffle subtraction and merged) with an SVM dimensionality reduction, the PID estimates were well bias corrected. The estimation converged to the expected ground truth values of zero synergy and zero-redundancy, even for small trial numbers (results were very accurately corrected for the bias even down to $N = 256$ trials). Without bias corrections, far more trials would have been needed to conclude correctly that synergy and redundancy are zero.

While more work is needed to understand how to optimally scale up discrete PID to large populations, these results encourage us to think that the bias corrections methods developed here will be useful and crucial to devise scalable multidimensional versions of discrete PIDs.

Supplementary Note 8: Analytical derivation of Red_3 and Syn_3 bias

In this supplementary note we derive analytically the leading bias term of the three-way redundancy Red_3 and the three-way synergy Syn_3, for both the discrete and the Gaussian case.

Discrete PID

In this subsection we derive analytically the leading bias term of the three-way redundancy Red_3 and the three-way synergy Syn_3 in the discrete case.

As shown in Supplementary Note 6, in the asymptotic regime, the leading term of the bias of discrete Shannon information quantities scales as the number of parameters defining the highest-dimensional probability distribution entering the definition of the corresponding information quantity.

The bias of Red_3 scales as the bias of the minimum single-source Shannon information quantity (Eq. (30)). Thus, following the arguments of Supplementary Note 6, the bias of Red_3 scales as $(S \times R)/N$.

The bias of Syn_3 is the difference between the bias of the three-source joint information and the bias of the maximum two-source joint information (Eq. (31)). The three-source joint information can be expressed as a function of entropy terms as

$$I(S; R_1, R_2, R_3) = -H(S, R_1, R_2, R_3) + H(R_1, R_2, R_3) + H(S) \tag{S23}$$

Using the leading bias term for entropy as in Eq. (S9), we obtain the following expression for the three-source Shannon information bias:

$$\langle I_N(S; R_1, R_2, R_3)\rangle - I(S; R_1, R_2, R_3) = \frac{1}{2N\ln(2)}\left[\sum_s (\tilde{R}_{s,r_1,r_2,r_3} - 1) - (\tilde{R}_{r_1,r_2,r_3} - 1) - (S-1)\right] \tag{S24}$$

where $\langle I_N(S; R_1, R_2, R_3)\rangle$ denotes the average value of the three-source information estimated with a finite number of trials N, taken over all instantiations with N trials. $\tilde{R}_{s,r_1,r_2,r_3}$ is the number of bins with non-zero joint probability $p(s, r_1, r_2, r_3)$, and $\tilde{R}_{r_1,r_2,r_3}$ is the number of bins with non-zero marginal probability $p(r_1, r_2, r_3)$. If all or most bins have non-zero probability then $\tilde{R}_{s,r_1,r_2,r_3} \approx S \times R^3$, while $\tilde{R}_{r_1,r_2,r_3} \approx R^3$. Thus, in the asymptotic regime the leading term of the $I(S; R_1, R_2, R_3)$ bias scales as $(S \times R^3)/N$. Instead, as discussed in Supplementary Note 6 the leading term of the largest two-source Shannon information scales as $(S \times R^2)/N$. Thus, the bias of Syn_3 is dominated by the bias of the three-source joint information and scales as $(S \times R^3)/N$ in the asymptotic regime.

In summary, in the discrete case, the bias of Syn_3 scales much more quickly than the one of Red_3 (with the difference between them even greater than the one between the synergy and the redundancy of the two-source PID). This prediction is confirmed by all our simulations and experimental results (Fig. 7 and 8).

Gaussian PID

In this subsection we derive analytically the leading bias term of Red_3 and Syn_3 in the MMI Gaussian case (since gPID is only defined for two source variables[S1]).

As shown in Supplementary Note 6, the bias of Gaussian Shannon information quantities is determined by a sum of digamma-function terms whose upper limit is set by the dimensionality of the highest-dimensional entropy term (i.e. the largest covariance matrix) entering the definition of

the corresponding information quantity. Consequently, the upward bias of information estimates increases supralinearly with the dimensionality d of the sources and targets, decreases with the number of trials N, and grows more rapidly for information quantities involving higher-dimensional entropy terms.

The bias of Red_3 scales as the bias of the minimum single-source Shannon information (Eq. (30)). Thus, following the arguments of Supplementary Note 6, for the Gaussian PID the bias of Red_3 is dominated by the sum of digamma functions up to $2d$ dimensions (Eq. (S19)).

The bias of Syn_3 is the difference between the bias of the three-source joint information and the bias of the maximum two-source joint information (Eq. (31)). In Supplementary Note 6 (Eq. (S18)) we already showed that the bias of $I(S; R_i, R_j)$ is dominated by the sum of digamma functions up to $3d$ dimensions. Replacing the terms of Eq. (S23) using the Goodman formula (Eq. (S16)) we find the following analytical expression for the bias of $I(S; R_1, R_2, R_3)$:

$$\text{bias}[I(S; R_1, R_2, R_3)] = \frac{1}{2\ln(2)}\left(-\sum_{k=1}^{4d}\psi\left(\frac{N-k}{2}\right)+\sum_{k=1}^{3d}\psi\left(\frac{N-k}{2}\right)+\sum_{k=1}^{d}\psi\left(\frac{N-k}{2}\right)\right) \quad \text{(S25)}$$

Thus, the bias of $I(S; R_1, R_2, R_3)$ is dominated by the sum of digamma functions up to $4d$ dimensions, and it determines the leading term of the bias of Syn_3.

In summary, the Gaussian bias of the three-way synergy Syn_3 is dominated by terms of dimension $4d$ and is much larger than the bias of the three-way redundancy Red_3 (dominated by terms of dimension $2d$). Moreover, the gap between Syn_3 and Red_3 bias grows supralinearly both with increasing the number of dimensions d and with decreasing number of trials N. These predictions are confirmed by all our simulations and experimental results (Fig. 7 and 8).

References

S1. Venkatesh, P., Bennett, C., Gale, S., Ramirez, T., Heller, G., Durand, S., Olsen, S., and Mihalas, S. (2023). Gaussian partial information decomposition: Bias correction and application to high-dimensional data. Advances in Neural Information Processing Systems *36*, 74602–74635. URL: proceedings.neurips.cc/paper_files/paper/2023/file/ec0bff8bf4b11e36f874790046dfdb65-Paper-Conference.pdf.

S2. Williams, P. L., and Beer, R. D. (2010). Nonnegative decomposition of multivariate information. Preprint at arXiv. doi:doi.org/10.48550/arXiv.1004.2515.

S3. Barrett, A. B. (2015). Exploration of synergistic and redundant information sharing in static and dynamical Gaussian systems. Phys. Rev. E *91*, 052802. doi:10.1103/PhysRevE.91.052802.

S4. Harder, M., Salge, C., and Polani, D. (2013). Bivariate measure of redundant information. Phys. Rev. E *87*, 012130. doi:10.1103/PhysRevE.87.012130.

S5. Rauh, J., Banerjee, P. K., Olbrich, E., Montúfar, G., and Jost, J. (2023). Continuity and additivity properties of information decompositions. Int. J. Approx. Reason. *161*, 108979. doi:10.1016/j.ijar.2023.108979.

S6. Kolchinsky, A. (2022). A novel approach to the partial information decomposition. Entropy *24*. doi:10.3390/e24030403.

S7. Bertschinger, N., Rauh, J., Olbrich, E., Jost, J., and Ay, N. (2014). Quantifying unique information. Entropy *16*, 2161–2183. doi:10.3390/e16042161.

S8. Gomes, A. F. C., and Figueiredo, M. A. T. (2024). A measure of synergy based on union information. Entropy *26*, 271. doi:10.3390/e26030271.

S9. Tolhurst, D. J., Movshon, J. A., and Dean, A. F. (1983). The statistical reliability of signals in single neurons in cat and monkey visual cortex. Vision Res. *23*, 775–785. doi:10.1016/0042-6989(83)90200-6.

S10. Panzeri, S., Brunel, N., Logothetis, N. K., and Kayser, C. (2010). Sensory neural codes using multiplexed temporal scales. Trends Neurosci. *33*, 111–120. doi:10.1016/j.tins.2009.12.001.

S11. Cover, T. M., and Thomas, J. A. Elements of Information Theory. Wiley & Sons (2006). doi:10.1002/0471200611.

S12. Treves, A., and Panzeri, S. (1995). The Upward Bias in Measures of Information Derived from Limited Data Samples. Neural Comput. *7*, 399–407. doi:10.1162/neco.1995.7.2.399.

S13. Panzeri, S., and Treves, A. (1996). Analytical estimates of limited sampling biases in different information measures. Network *7*, 87–107. doi:10.1088/0954-898X/7/1/006.

S14. Paninski, L. (2003). Estimation of entropy and mutual information. Neural Comput. *15*, 1191–1253. doi:10.1162/089976603321780272.

S15. Strong, S. P., Koberle, R., De Ruyter Van Steveninck, R., and Bialek, W. (1998). Entropy and information in neural spike trains. Phys. Rev. Lett. *80*, 197. doi:10.1103/PhysRevLett.80.197.

S16. Panzeri, S., Senatore, R., Montemurro, M. A., and Petersen, R. S. (2007). Correcting for the sampling bias problem in spike train information measures. J. Neurophysiol. *98*, 1064–1072. doi:10.1152/jn.00559.2007.

S17. Averbeck, B. B., Latham, P. E., and Pouget, A. (2006). Neural correlations, population coding and computation. Nat. Rev. Neurosci. *7*, 358–366. doi:10.1038/nrn1888.

S18. Panzeri, S., Moroni, M., Safaai, H., and Harvey, C. D. (2022). The structures and functions of correlations in neural population codes. Nat. Rev. Neurosci. *23*, 551–567. doi:10.1038/s41583-022-00606-4.

S19. Schneidman, E., Bialek, W., and Berry, M. J. (2003). Synergy, redundancy, and independence in population codes. J. Neurosci. *23*, 11539–11553. doi:10.1523/JNEUROSCI.23-37-11539.2003.

S20. Pola, G., Thiele, A., Hoffmann, K., and Panzeri, S. (2003). An exact method to quantify the information transmitted by different mechanisms of correlational coding. Network *14*, 35–60. doi:10.1088/0954-898X/14/1/303.

S21. Goodman, N. (1963). The distribution of the determinant of a complex wishart distributed matrix. Ann. Math. Stat. *34*, 178–180. doi:10.1214/aoms/1177704251.

S22. Cai, T. T., Liang, T., and Zhou, H. H. (2015). Law of log determinant of sample covariance matrix and optimal estimation of differential entropy for high-dimensional Gaussian distributions. J. Multivar. Anal. *137*, 161–172. doi:10.1016/j.jmva.2015.02.003.

S23. Lorenz, G. M., Engel, N. M., Celotto, M., Koçillari, L., Curreli, S., Fellin, T., and Panzeri, S. (2025). MINT: a toolbox for the analysis of multivariate neural information coding and transmission. PLOS Comput. Biol. *21*, e1012934. doi:10.1371/journal.pcbi.1012934.

S24. Iurilli, G., and Datta, S. R. (2017). Population coding in an innately relevant olfactory area. Neuron *93*, 1180–1197. doi:10.1016/j.neuron.2017.02.010.

S25. Valente, M., Pica, G., Bondanelli, G., Moroni, M., Runyan, C. A., Morcos, A. S., Harvey, C. D., and Panzeri, S. (2021). Correlations enhance the behavioral readout of neural population activity in association cortex. Nat. Neurosci. *24*, 975–986. doi:10.1038/s41593-021-00845-1.
