# Supplementary material for: Sampling bias corrections for discrete and Gaussian partial information decompositions
Source: Patterns (N Y). 2026 Jul 23;7(9):101619. doi: 10.1016/j.patter.2026.101619 (PMC13576656; doi:10.1016/j.patter.2026.101619)
Supplement: Supplementary file 2 [file mmc2.pdf]

Article

Patterns

Sampling bias corrections for discrete and Gaussian partial information decompositions

Graphical abstract

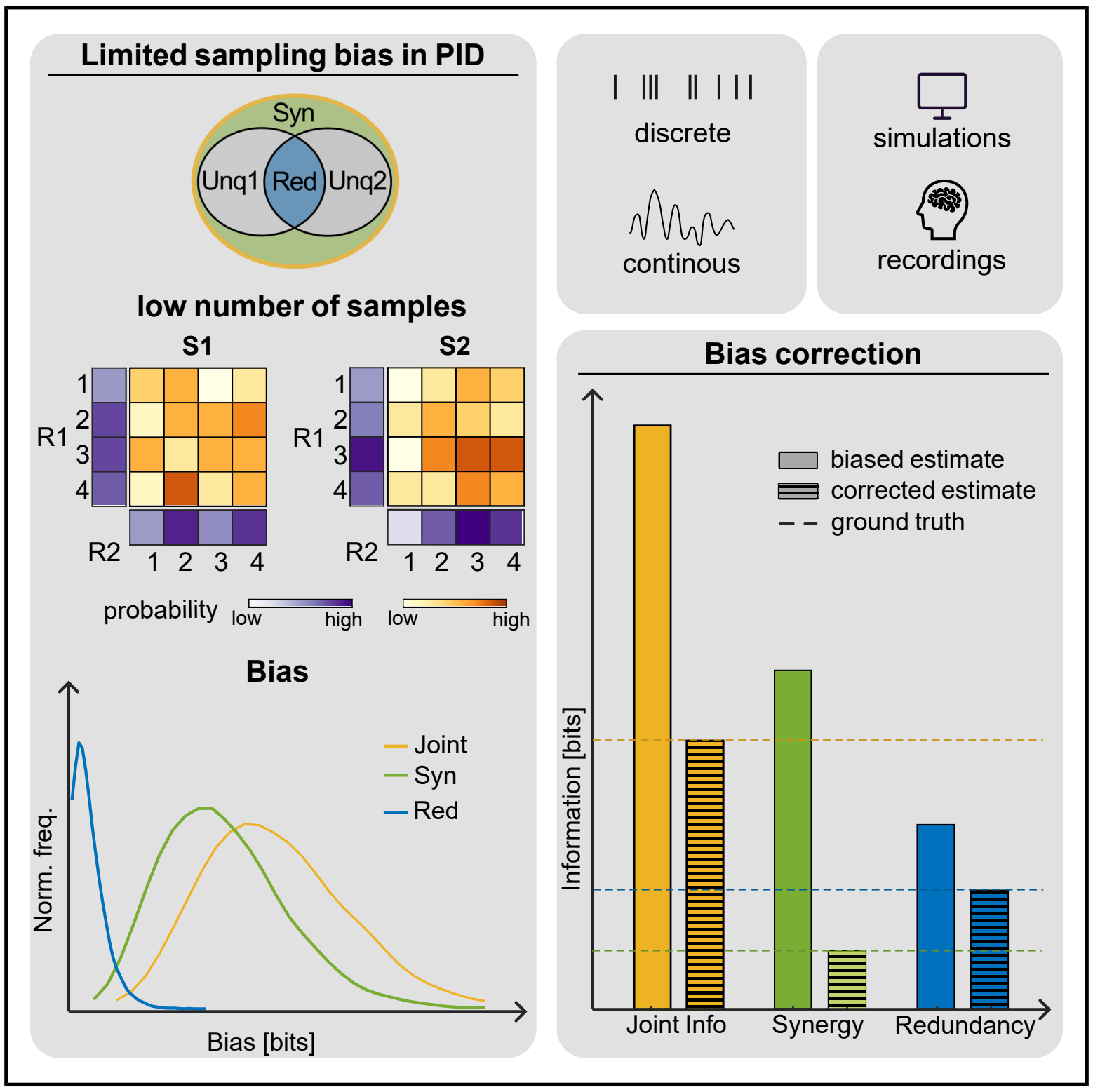

Highlights

- Partial information decompositions suffer from major limited-sampling bias
- The bias is much larger for information synergy than for redundancy
- New bias-correction methods are developed and validated
- These methods work effectively on simulated and real brain data

Authors

Gabriel Matías Lorenz,
Nicola Marie Engel, Loren Koçillari, ...,
Tommaso Fellin, Andrea I. Luppi,
Stefano Panzeri

Correspondence

s.panzeri@uke.de

In brief

Lorenz, Engel et al. characterize the limited-sampling bias in partial information decomposition calculations. The bias is substantial when computed on empirical brain datasets, but the bias-correction methods developed here are demonstrated to work highly effectively to produce unbiased estimates of information synergy and redundancy.

Lorenz et al., 2026, Patterns 7, 101619
September 11, 2026 © 2026 The Authors. Published by Elsevier Inc.
https://doi.org/10.1016/j.patter.2026.101619

CellPress

Patterns

CellPress
OPEN ACCESS

Article

Sampling bias corrections for discrete and Gaussian partial information decompositions

Gabriel Matías Lorenz,[1,10] Nicola Marie Engel,[1,10] Loren Koçillari,[1,2,10] Marco Celotto,[1,3,10] Davide Orsenigo,[4] Sebastiano Curreli,[5] Simone Blanco Malerba,[1] Andreas K. Engel,[2] Christoph Kayser,[6] Tommaso Fellin,[5] Andrea I. Luppi,[7,8,9] and Stefano Panzeri[1,11,*]

[1]Institute for Neural Information Processing, Center for Molecular Neurobiology, University Medical Center Hamburg-Eppendorf (UKE), 20251 Hamburg, Germany
[2]Department of Neurophysiology and Pathophysiology, University Medical Center Hamburg-Eppendorf (UKE), 20246 Hamburg, Germany
[3]Picower Institute for Learning and Memory, Massachusetts Institute of Technology, Cambridge, MA 02139, USA
[4]Department of Psychology, University of Torino, 10124 Turin, Italy
[5]Optical Approaches to Brain Function Laboratory, Istituto Italiano di Tecnologia, 16163 Genova, Italy
[6]Department of Cognitive Neuroscience, Bielefeld University, 33501 Bielefeld, Germany
[7]Department of Psychiatry, University of Oxford, Oxford OX3 7JX, UK
[8]St. John's College, University of Cambridge, Cambridge CB2 1TP, UK
[9]Division of Information Engineering, University of Cambridge, Cambridge CB2 1PZ, UK
[10]These authors contributed equally
[11]Lead contact
*Correspondence: s.panzeri@uke.de
https://doi.org/10.1016/j.patter.2026.101619

THE BIGGER PICTURE Cognitive functions emerge because neurons interact and communicate. Recent work has used the formal principles of information theory to uncover how the interactions between neurons or brain areas contribute to information relevant for cognition.

This work has uncovered two types of interactions. Redundant interactions share the same information among different neurons, and this redundancy can make brain function more reliable, because the same information is available in more than one place. Synergistic interactions occur when a group of neurons carries a message that cannot be found in any member of the group alone. This synergy may allow the brain to represent complex aspects of the world and to use combinations of signals in flexible ways.

A central challenge is that these forms of cooperation must be inferred from experimental recordings. These datasets are limited, because animals and people can perform only so many trials, clinical datasets are especially difficult to collect, and recordings from many brain areas create many quantities that must be estimated at once.

We show how limited data affect estimates of redundancy and synergy, and we provide corrections that make these estimates more reliable. We also give guidance on how much data are needed for different types of analysis, helping researchers to plan experiments as well as analyze completed datasets. By reducing misleading effects of small sample sizes, these tools make it possible to compare redundancy and synergy more fairly across experiments, species, recording methods, and brain systems. They also open the door to studying these forms of neural cooperation in settings where data are precious, such as patient studies, disease models, and recordings from large-scale brain networks.

SUMMARY

Partial information decomposition (PID) has emerged as a principled way to decompose the information carried by neural activity into components identifying whether interactions among neurons or brain areas generate synergistic or redundant information. Here, we demonstrate that empirical measures of synergy and redundancy based on either Gaussian or discrete probability estimators suffer from a substantial limited-sampling estimation bias. This bias is much larger for synergy than for redundancy. The gap between them increases with the number of parameters specifying the probability distributions. We develop procedures that effectively correct for the bias and provide rules of thumb for the sample sizes required to obtain unbiased estimates. We show that, when used on empirical brain datasets, they successfully remove large

This is an open access article under the CC BY license (http://creativecommons.org/licenses/by/4.0/).

synergy biases across species, recording modalities, and experimental designs. Our bias corrections extend the range of neuroscience questions and experimental designs addressable with PID and allow accurate comparisons between synergy and redundancy.

INTRODUCTION

The brain is a complex system whose behavior emerges from the interactions between neurons or brain areas.[1] Because of this, the study of how interactions between neurons or brain areas shape information processing has fascinated neuroscientists for decades.[2–6] Information theory has been a prominent tool in this research as it is uniquely positioned for investigating neural information processing.[7–9] Shannon information can capture all ways (linear and non-linear) in which systems carry information and is thus equally applicable across species and recording modalities,[10–14] real data, and neural network models.[15–18]

While earlier work has concentrated on understanding whether correlations between the activity of different neurons or brain areas increase or decrease information,[3,4,19–21] recent advances in information theory based on partial information decomposition (PID)[22] have enabled neuroscientists to formulate questions about the unique information carried by each individual neuron or brain area, the synergistic information generated by neural interactions (that is, new information that emerges specifically from and is found only in the interactions between neurons or areas), or redundant information present when different neurons or areas carry the same information.[23] These PID tools have been developed and used both in forms suitable for discrete neural responses (usually applied for the spiking activity of neurons) and in Gaussian-distributed neural responses (usually applied to aggregate measures of neural activity).

Earlier applications of information theory to neuroscience have recognized that Shannon information measures from neural activity suffer from a prominent limited-sampling bias.[24–26] This bias not only affects the accuracy of the estimate but can also skew comparisons between the information carried by simpler (less biased) and more complex (more biased) neural representations defined by a larger number of parameters. For example, the information about a stimulus carried jointly by two neurons is more biased than the information carried by each neuron individually. This would artificially inflate emergent effects if this bias is not corrected.[27] The information-theoretic neuroscience literature has long recognized the importance of correcting for the limited-sampling bias and has provided effective tools for correcting the Shannon information sampling bias.[24–28] However, to date the problem of whether and how synergy or redundancy components of PID are biased has not been studied systematically. While the problem of null models for information-theoretic quantities is gaining recognition,[29] to our knowledge only two studies have investigated PID sampling bias, specifically for a Gaussian PID[30] and for a PID with a mix of discrete and Gaussian variables.[31] Here, we study systematically the sampling bias of both discrete and Gaussian estimators of PID with two or three sources. We find that synergy is far more biased than redundancy, especially in the discrete case. Using extensive numerical simulations accompanied by analytical considerations, we study and provide an understanding of the origin and properties of the bias. Based on this understanding, we provide effective methods that reduce this problem to provide accurate measures. Finally, we test our bias corrections on empirical neuroscience datasets, from the brain of mice, monkeys, and humans, of spikes recorded with either two-photon calcium imaging or electrophysiology and of functional MRI (fMRI) or magnetoencephalography (MEG) signals.

RESULTS

Background: PID and its neuroscience use

For completeness, we first summarize the PID concepts we use. We focus this summary on the discrete PID, but the extension to Gaussian PID is straightforward (full details of discrete and Gaussian PIDs are given in methods). PID decomposes the information jointly carried by a set of source variables $\vec{R}$ (here, the activity of a set of simultaneously recorded neurons or brain areas, henceforth simply neural units) about a target S (here, a sensory stimulus or the activity of another simultaneously recorded neural unit) into components (non-negative for the PIDs we consider here) that capture information about the target that is either redundantly encoded across sources, uniquely encoded by each individual source, or synergistically encoded by the combination of sources, such that it is not available from any of them alone.[22] We focus this introduction on the case of two source variables (that is, $\vec{R} = (R_1, R_2)$), which has received the most attention and which has the most established theoretical foundations.[30,32–37] Unless otherwise stated, all our results refer to the two-source PID, while the three-source PID is considered separately in later sections. We consider the Shannon information $I(S;R_1,R_2)$ about the target variable S carried jointly by the activity of two neural units R_1 and R_2 and the Shannon information $I(S;R_i)$ that each of the two neural units (i = 1,2) carries about S. $I(S;R_1,R_2)$ and $I(S;R_i)$ are computed from the probability distributions $p(s, r_1, r_2)$ and $p(s,r_i)$ (i = 1, 2),[38,39] as follows:

$$I(S;R_i) = \sum_{s \in S, r_i \in R_i} p(s,r_i) \log \frac{p(s,r_i)}{p(s)p(r_i)}, \quad \text{(Equation 1)}$$

$$I(S;R_1,R_2) = \sum_{s \in S, r_1 \in R_1, r_2 \in R_2} p(s,r_1,r_2) \log \frac{p(s,r_1,r_2)}{p(s)p(r_1,r_2)}. \quad \text{(Equation 2)}$$

PID decomposes $I(S;R_1,R_2)$ into the sum of four components, subject to three constraints involving the two single-source mutual information $I(S;R_1)$ and $I(S;R_2)$:

$$I(S;R_1,R_2) = Red(S:R_1;R_2) + Unq(S:R_1 \backslash R_2) + Unq(S:R_2 \backslash R_1) + Syn(S:R_1;R_2), \quad \text{(Equation 3)}$$

$$I(S;R_1) = Red(S:R_1;R_2) + Unq(S:R_1 \backslash R_2), \quad \text{(Equation 4)}$$

$$I(S;R_2) = Red(S:R_1;R_2) + Unq(S:R_2 \backslash R_1), \quad \text{(Equation 5)}$$

where $Red(S:R_1;R_2)$ is the redundant (or shared) information that both R_1 and R_2 encode about S; $Unq(S:R_1\backslash R_2)$ and $Unq(S:R_2\backslash R_1)$ are the unique information about S provided by one neural unit but not by the other; and $Syn(S:R_1;R_2)$ is the synergistic information about S encoded jointly by R_1 and R_2. Equations 3, 4, and 5 imply that all four components contribute to the joint information, whereas only unique and redundant components contribute to single-neural unit information. Because the four PID components satisfy three linear constraints (Equations 3, 4, and 5), determining the value of one of the four components and the Shannon information values is sufficient to compute the other three PID terms (see equations of all components as a function of synergy in Equation 16).

Several definitions of PID have been proposed, which differ in their theoretical properties, including in whether redundancy and unique information do or not depend only on source-target pairwise marginals and in whether individual terms are or are not required to be non-negative.[40–45] We consider in our study some of those that satisfy the two properties of non-negativity of each component and symmetry of redundancy and synergy under permutation of R_1,R_2, as the resulting components are clearly interpretable as fractions of the joint mutual information $I(S;R_1,R_2)$ and are invariant to arbitrary reordering of the sources. In this section, we summarize the PID that we will use the most in our simulations unless otherwise stated, that is, the BROJA (Bertschinger-Rauh-Olbrich-Jost-Ay) PID (defined in Bertschinger et al.[33] for discrete PID and in Venkatesh et al.,[30] termed gPID, for Gaussian PID). It satisfies many desirable properties including additivity of all PID components for independent systems of sources and targets (proven for discrete[33,46] and empirically validated for Gaussian PID[30]) and has been widely applied to neural data.[13,36,37,47,48] The BROJA PID defines the union information, that is, the target information in the joint source space that cannot be possibly attributed to synergistic interactions or, equivalently, the total target information that can be extracted from a single source, as

$$Union(S:R_1;R_2) = \min_{q \in \Delta_P} I_q(S;R_1,R_2), \quad \text{(Equation 6)}$$

where Δ_P is the set of all joint probability distributions $q(s,r_1,r_2)$ that have the same pairwise marginals $q(s,r_1) = p(s,r_1)$ and $q(s,r_2) = p(s,r_2)$ as the original distribution $p(s,r_1,r_2)$, and $I_q(S;R_1,R_2)$ is the joint information computed for distribution $q(s,r_1,r_2)$. The synergy is then defined as the difference between the joint and the union information:

$$Syn(S:R_1;R_2) = I(S;R_1,R_2) - Union(S:R_1;R_2). \quad \text{(Equation 7)}$$

Other PID definitions that satisfy non-negativity and symmetry but do not satisfy additivity were also successfully applied to neuroscience and were used for both discrete and Gaussian calculations.[35,49–52] These include the original PID termed I_{min}[22] (quantifying redundancy as the similarity between R_1 and R_2 in discriminating individual values of S), and the PID termed minimum mutual information I_{MMI}[34] (quantifying redundancy as the minimum between the mutual information individually carried by R_1 and R_2, thus capturing the amount but not the content of information carried by each neural unit[41]).

An approach extensively used in neuroscience for both Shannon information[3,10,13,48,53–64] and PID[13,35,36,47–50,64–69] is the discrete approach, in which all the variables are discrete or are discretized. The discrete PID can be computed by simply estimating the probabilities as the empirical occurrences across experimental samples (maximum-likelihood estimators) and plugging them into the PID equations (the so-called plugin approach[64]). The discrete approach is suitable for many neuroscience applications because traditional quantifications of neural spiking activity naturally lead to discrete variables. When focusing on spike count codes, the neural response is described by the number of spikes emitted by the neuron in the time window of interest.[53,54,56–58,70,71] When investigating whether the timing of spikes encodes additional information above and beyond that present in spike counts, the most established approach[10,59,60,72,73] is to discretize the neural response time window of interest into a number of small time bins and then turn the spike train into a binary word. In addition, in most experiments, often only a discrete number of different stimulus conditions is presented, usually with uniform probability, and thus the target S is often a discrete variable. In such conditions, as we show in Figure S13 and Note S5, the Gaussian PID may give inaccurate information values and PID components that are not credible (e.g., finding synergy or redundancy that clearly should not be present in the distributions). Because it is simple and does not require assumptions on the probability distributions, discretization of neural responses for computing information has also been used to compute information from continuous-valued non-spiking aggregate measures of neural activity such as local field potentials (LFPs), electroencephalography (EEG), or fMRI.[69,74–77]

Another approach extensively used in neuroscience is the Gaussian PID, which assumes Gaussian multivariate distributions for sources and target.[30,34,78] The Gaussian PID can be computed from the covariance matrix, as this matrix fully defines the Gaussian probability distributions. The Gaussian PID is thus empirically computed from real data by estimating covariance matrices from the data and inserting them into the PID equations (see methods). Gaussian PIDs are well suited to analyze the activity of continuous-valued non-spiking aggregate measures of neural activity such as LFP, EEG, or fMRI.[49,51]

Bias of synergy and redundancy for discrete and Gaussian estimators

Calculations of information and PID require the accurate estimation of the target-source joint probabilities. With an infinite amount of data, the true probabilities (or the parameters defining them) could be measured exactly. However, any real neuroscience experiment will only yield a finite number of samples N that can be used to estimate the probabilities. For example, in some neuroscience experiments a sensory stimulus (the target) will be presented in a given trial, and the activity of different neural units (sources) will be recorded. If the neural activity is quantified as a single number per trial (for example, the spike count),[56,70] the number of samples N to compute the

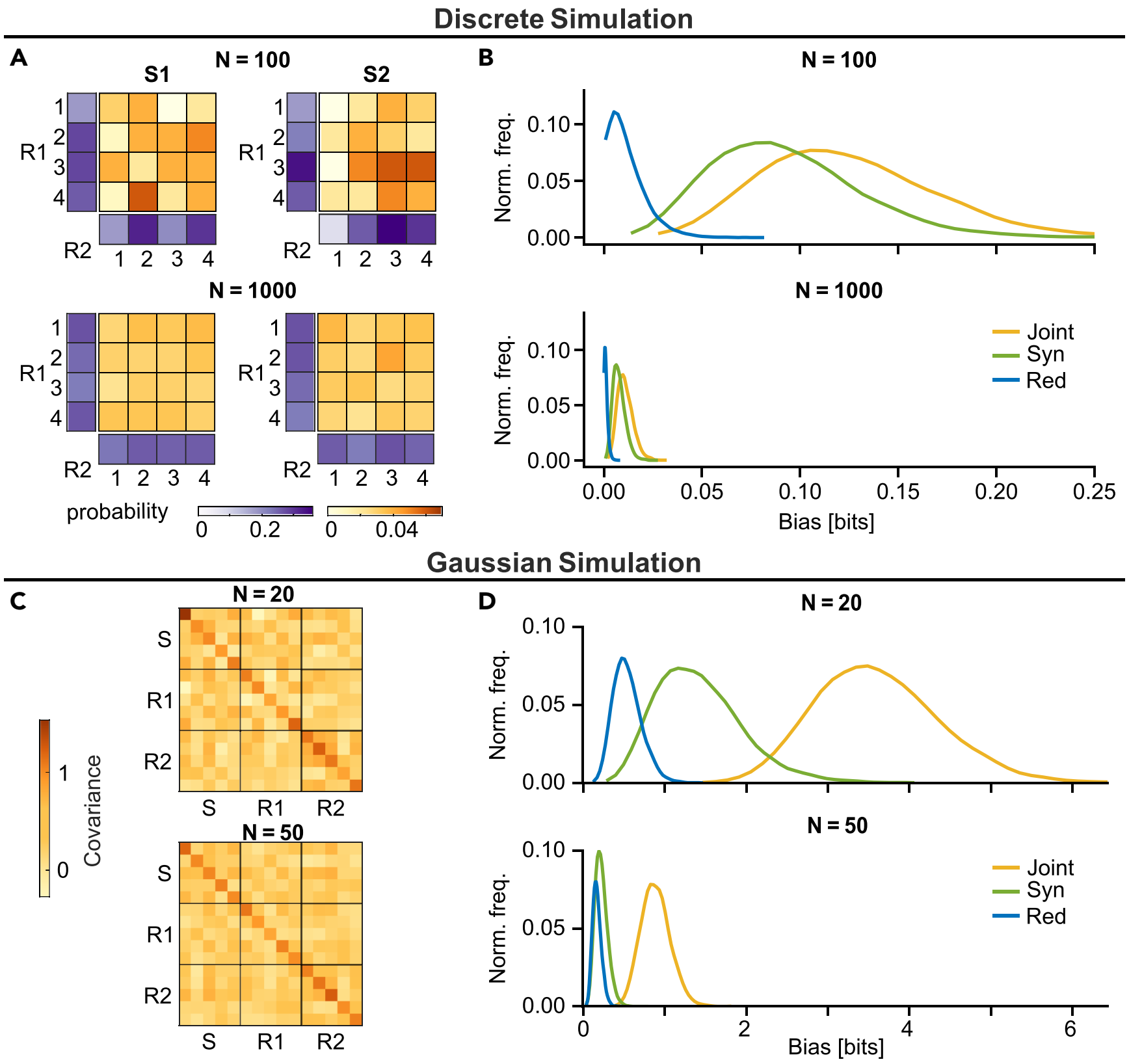

Figure 1. Illustration of the origin of the limited-sampling bias

(A) Illustration of the bias for the discrete BROJA PID. Two uninformative neural units (in this example, two spiking neurons) respond with spike counts uniformly distributed from 1 to 4 (i.e., $R = 4$ bins) independently of which of $S = 2$ stimuli (target) is presented. Empirical stimulus-specific joint response probabilities are estimated from $N = 100$ (top) and $N = 1{,}000$ (bottom) samples and plotted with an orange color scale. Marginal distributions are shown at the margins (blue color scale). Deviations from uniformity are evident and are stronger for joint than for marginal distributions and for lower N.

(B) Distribution (over 5,000 simulations) of the plugin (i.e., not bias-corrected) joint information, synergy (*Syn*), and redundancy (*Red*) values obtained with 100 (top) and 1,000 (bottom) samples respectively.

(C) Illustration of bias for the Gaussian gPID. Empirical covariance matrices are estimated from $N = 20$ (top) and $N = 50$ (bottom) samples and plotted with an orange color scale. Presented stimuli (S) and responses of the two uninformative neural units (R_1 and R_2) are five-dimensional independent and identically distributed Gaussian variables with zero mean and unit variance. Deviations from diagonal covariance matrices are evident and stronger for lower N.

(D) Distribution (over 5,000 simulations) of the plugin (i.e., not bias-corrected) joint information, synergy (*Syn*), and redundancy (*Red*) values obtained with 20 (top) and 50 (bottom) samples, respectively.

For details, see Note S3.

probabilities will equal the number of trials. If several samples of neural activity can be taken in each stimulus presentation trial,[63,79] the number of samples will be equal to the number of trials times the number of samples per trial. In other cases, such as when computing information between the activity of some neural population (the target) and the activity of another neural population (the sources) in resting-state conditions, a trial structure may be absent, and the number of samples equals the number of time points at which activity of the different populations can be sampled during the experiment.[35,49]

When real-data probabilities are estimated from the finite number of available samples N, both the probabilities to be estimated for the discrete PID and the covariance matrices needed for the Gaussian PID have finite sampling fluctuations around their true values, which make the distributions of sources typically more dependent on the target than they really are (Figures 1A and 1C). As a result, the values of information estimated from a finite amount of samples are not centered around the underlying ground-truth information value (Figures 1B and 1D). This leads to both a systematic error (limited-sampling bias) and a statistical error (limited-sampling variance) in information estimates. While variance can be reduced by averaging (e.g., across experimental subjects or groups of neural units), the limited-sampling bias (shortened to bias hereafter) cannot. Although the bias of Shannon information has been studied extensively for both discrete[24,25,27] and Gaussian estimators,[80,81] the bias of individual PID components has remained largely unexplored. Here, we study the bias of the discrete and Gaussian PIDs with numerical simulations, supported by analytical considerations (see Note S6). We simulated (for details see methods) either spike count or Gaussian processes with non-zero ground-truth values for each PID component (because this is the most general case, and it mimics the fact that all PID components had non-zero values in all real neural datasets we investigated). We varied the overall joint information level of these processes to investigate how the bias depended on it (see Figures S4, S5, S9, S10, and S11 for tests on simulated scenarios with some PID components having a null ground-truth value). We focused on the two-source BROJA PID (discrete as in Bertschinger et al.[33] and Gaussian as in Venkatesh et al.[30]), but we confirmed our results with the two-source I_{min} and I_{MMI} (Figures S6 and S7). To simplify presentation, we focused on the bias of redundancy and synergy, as these components are of the highest interest for studying neural interactions. However, the bias properties of the sum of the unique information terms can be inferred by difference between the bias of the joint information and that of synergy and redundancy and will be recapitulated in Figure S2.

We first studied the discrete two-source PID. We simulated (Figure 2) spike counts of two individual neurons (sources), each binned into equipopulated bins, emitted in response to different stimuli (target). In this simulation, and in all others

CellPress
OPEN ACCESS

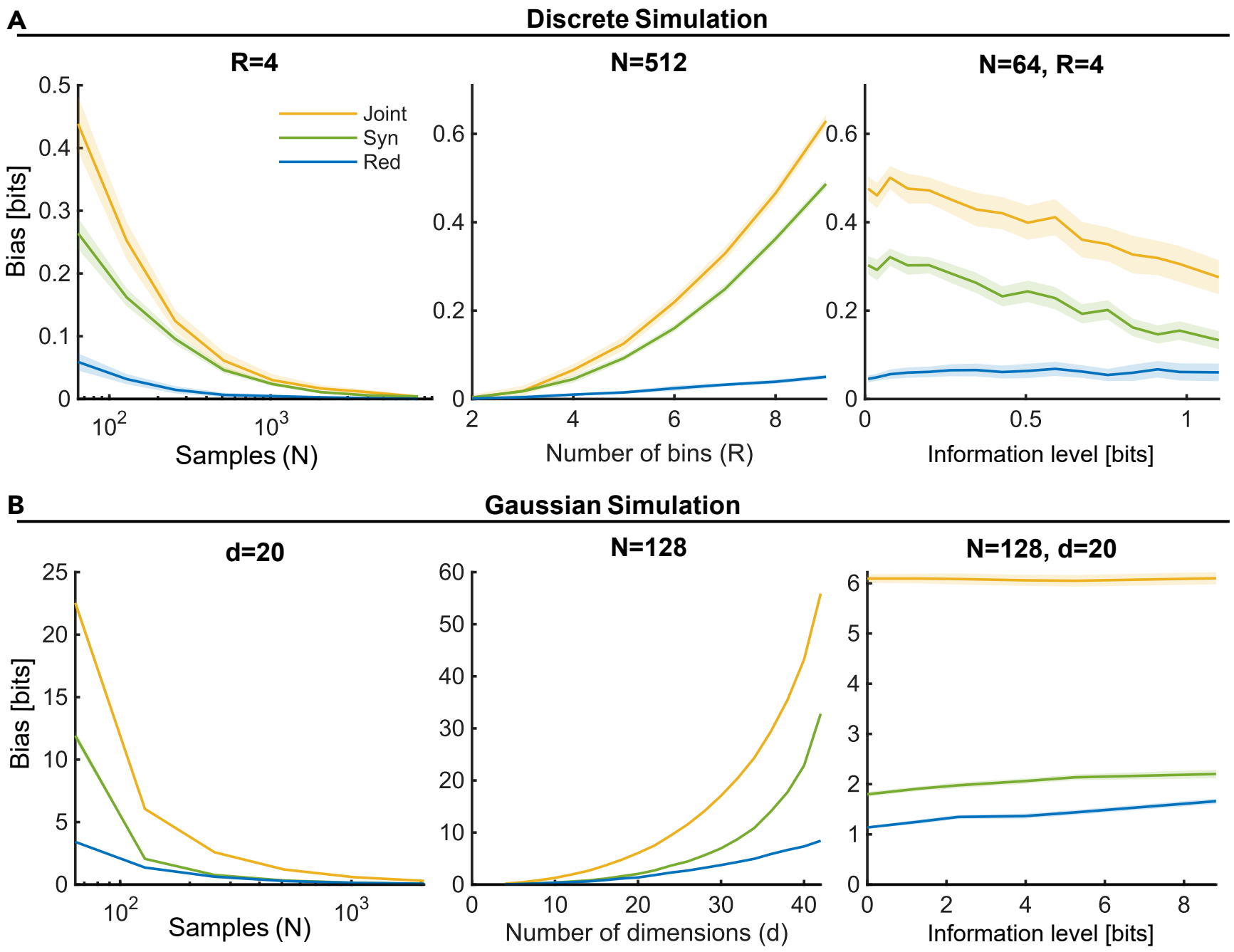

Figure 2. Bias of the plugin PID across number of samples, bins or dimensions, and information level

The bias of the plugin (i.e., not bias-corrected) PID terms—joint information (*Joint*), synergy (*Syn*), and redundancy (*Red*)—is computed as averaged empirical estimates from N samples minus ground-truth values (see methods).

(A) Discrete BROJA PID simulations. Left: we vary (on a log x axis) the number of samples N used to compute the PID (number of bins $S = R = 4$) in the low information value regime. Middle: we vary the number of bins $S = R$ (number of samples $N = 512$) in the low information value regime. Right: we vary the joint information level by changing the value of the simulation parameter α regulating the stimulus-response gain ($N = 64$, $S = R = 4$).

(B) Gaussian gPID simulations. Left: we vary (on a log x axis) the number of samples N used to compute the PID components (number of dimensions $d = 20$) in the low information value regime. Middle: we vary the number of dimensions d of sources and targets ($N = 128$) in the low information value regime. Right: we vary the joint information level by changing the value of parameter α scaling the diagonal of the covariance matrix ($N = 128$, $d = 20$).

We plot mean ± 3 SEM over 100 simulations. Discrete and Gaussian simulations were performed under the bit-of-all scenario (see methods and Table S2). For more details, see Table S1.

presented in this paper, one sample per stimulus presentation (trial) was collected, and thus the number of samples N to compute probabilities equaled the number of simulated trials. We studied how the plugin estimate of the joint information and of each PID component depends on the number of available samples (in all simulation figures, we averaged over $n = 100$ simulation repetitions). We term the plugin estimate[27,64] as the one that computes information directly plugging the empirical probabilities into the information and PID equations (Equations 1, 2, 3, 4, and 5). The limited-sampling bias in the plugin information estimates is the difference between the average across simulations of the information obtained with a given number of samples and the ground-truth value computed from the true probabilities. As previously reported,[27,60] the plugin estimate of the joint Shannon information has an upward bias, which decreases with the number of samples N (Figures 1B and 2A [left]), increases with the number of discrete bins R, S of each source and target variable (Figure 2A, middle, where we keep $R = S$ for simplicity), and decreases with the information level (Figure 2A, right). These properties are explained by analytical results (obtained in the large-N limit; Note S6) showing that the bias decreases with N as powers of $1/N$ and that it is proportional to the number of discrete bins R, S. The decrease of the bias with the information level can also be explained from these analytical calculations,[24] because the bias increases with the number of discrete responses that are possible (i.e., with non-zero probability). Low information levels correspond to less concentrated stimulus-specific distributions and, thus, have a larger number of possible responses and are more biased.

We then studied the bias of the plugin estimates of redundancy and synergy computed with the discrete PID. We found several highly consistent results (Figure 2A). First, as for the Shannon joint information, synergy and redundancy have an upward bias decreasing with N (Figure 2A, left). Second, for the tens of samples usually available in neuroscience experiments, the bias could be as large as, or even larger than, the target quantities (Figures 2A and S5). This implies that the bias must be corrected for in real-data analyses. Third, the bias of the synergy is larger both proportionally and in absolute terms for lower information levels (Figure 2A, right). Fourth, and unlike what was assumed in a previous study,[30] the bias is highly uneven across PID components. When varying, at fixed N, the number of discrete bins R, S of each source and target variable (keeping $R = S$ for simplicity), the bias of the synergy increases rapidly and supralinearly with R (Figure 2A, middle), whereas the bias of the redundancy grows much more slowly and is much smaller. The bias of the sum of the Unique information terms (which is the difference between the bias of the joint information and the biases of synergy and redundancy) is plotted in Figure S2A. It increases with $1/N$ and R and is in most cases intermediate between that of synergy and redundancy in terms of size and rate of growth with $1/N$ and R.

We next study the bias of the Gaussian PID (Figure 2B). The Gaussian joint information has an upward bias that decreases with the number of samples N (Figure 2B, left) and increases with the dimensionality d of the sources and target (Figure 2B, middle). As predicted by an exact analytical bias expression valid for $N > 3d$,[80,81] the joint information bias depends only on N and d but is independent of the information level (Figure 2B, right). Synergy and redundancy are also biased upward. Because the Gaussian joint information bias does not depend on the information level, the bias of synergy and redundancy does not increase for lower information levels as in the discrete case (Figure 2B, right). Instead, the synergy and redundancy bias very slightly increases with the information level. Importantly, the bias of the Gaussian PID is also highly uneven

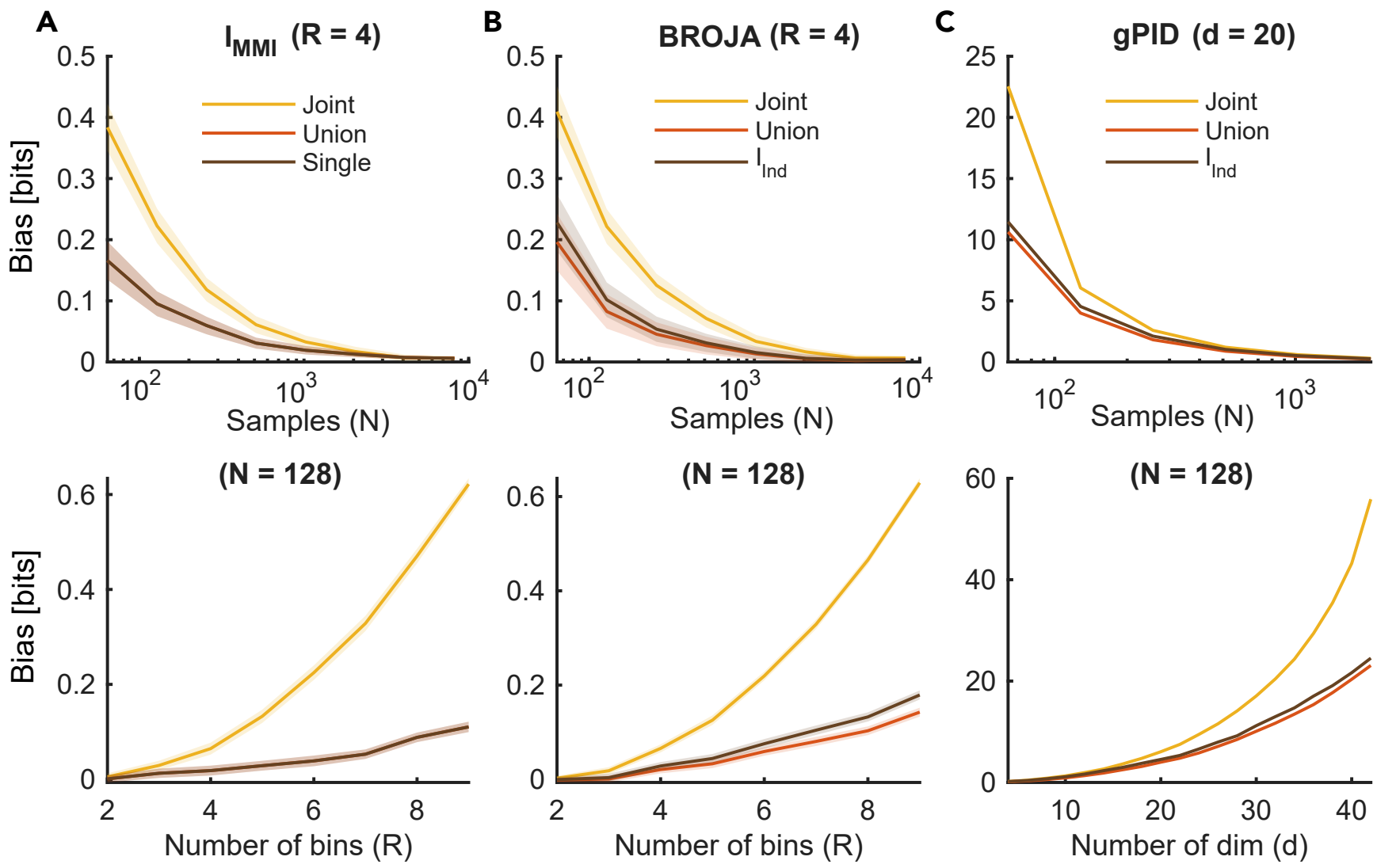

Figure 3. Simulations comparing the bias of union information to that of other information-theoretic quantities with an analytically tractable bias

(A) Discrete simulations (using MMI PID) showing the bias (averaged empirical estimates from N samples minus ground-truth values) as a function of the number of simulated samples N (on a log x axis) using $S = 4$ stimuli and $R = 4$ bins per source (top) and as a function of the number of stimuli S and response bins R fixing $R = S$ (bottom, with $N = 128$). The bias of the union information matches the one of the single-source information ("single") because in MMI the union information equals the maximum single-source information.

(B) Same as (A) (discrete simulations) but using the BROJA PID. The bias of the union information is compared to that of the conditionally independent joint information I_{ind}, whose bias depends on the fluctuations of the marginal probabilities of one target and one source and is thus less than that of the joint information.

(C) Gaussian simulations using the Gaussian gPID computed as a function of the number of simulated samples N using $d = 20$ dimensions for target and sources (top) and as a function of the dimension d of sources and target (bottom, $N = 128$). The bias of the union information is compared to that of the conditionally independent information I_{ind}, whose bias depends on the fluctuations of the marginal probabilities of one target and one source.

Results in each panel are plotted as mean ± 3 SEM over 100 simulations with the considered number of samples. Discrete and Gaussian simulations were performed under the bit-of-all scenario (see methods and Table S2). For more details, see Table S1.

between synergy and redundancy. When varying d at fixed N (Figure 2B, middle), the bias of both synergy and redundancy increases with d, but it increases faster for synergy than for redundancy. The bias of the sum of the Unique information terms is plotted in Figure S2B. It increases with $1/N$ and d and is in most cases intermediate between that of synergy and redundancy in terms of size and rate of growth with $1/N$ and d.

The fact that the bias of the synergy is consistently larger than that of the redundancy and that their difference increases with R for the discrete PID and with d for the Gaussian PID can be understood from analytical considerations, developed in Note S6 and briefly summarized here. The synergy bias equals the difference between the biases of joint and union information, whereas the redundancy bias equals the difference between the sum of single-source information biases and the union information bias. The bias of synergy and redundancy is thus determined by the bias of these quantities. For the discrete case, exact expressions for the bias are not known. However, approximate expressions are available in the large-N limit.[24] The bias of the joint information scales as $(S \times R^2)/N$ (as it requires the sampling of the joint distribution of s, r_1, r_2), while the bias of single-source information scales as $(S \times R)/N$ (it requires the sampling of the joint distribution of s and of one source). The MMI union bias equals the bias of the maximum single-source information (Equation S12) and thus scales as $(S \times R)/N$ and is smaller than the joint information bias (Figure 3A). The union information depends only on marginal distributions of the target and one source also for the BROJA PID. We therefore conjectured, and confirmed numerically (Figure 3B), that the bias of the BROJA union information approximately matches the analytically tractable bias of the conditionally independent information I_{ind} (computed from the conditional-independent distribution p_{ind}, which belongs to the set Δ_P, in which the union information is computed), which can be shown to scale as $(S \times R)/N$. In sum, for both MMI and BROJA discrete PID the synergy bias scales as $(S \times R^2)/N$, whereas redundancy bias grows at most as $(S \times R)/N$ as R increases. In the Gaussian case, an exact expression is available[81] for the mutual information bias, which grows steeply and supralinearly when increasing the dimension d of sources and targets and decreases when increasing N (Figure S14). The bias increases much faster with d for the joint information (scaling as a supralinear function of $3d$, as it contains bias of the entropy of the joint distribution of two sources and one target) than for the single-source information (scaling as a supralinear function of $2d$, as it contains bias of the entropy of the joint distribution of one source and one target). As for the discrete case, the bias of the MMI union information scales as the single-source information. For the gPID[30] (the Gaussian analog of the discrete BROJA PID), we also conjectured—and confirmed numerically—that the union bias is similar to the analytically tractable bias of the conditionally independent information I_{ind} (Figure 3C), which, as for the bias of the single-source information, scales as a supralinear function of $2d$. The Gaussian MMI and the gPID synergy bias are thus dominated by the bias of entropies of $3d$-dimensional distributions that scale as a supralinear function of $3d$, whereas the redundancy bias contains only biases of entropies of $2d$-dimensional distributions that scale as a supralinear function of $2d$. As a result, in the Gaussian case, the synergy is more biased than the redundancy, and the gap in bias between the two grows supralinearly with d.

To recapitulate, for the discrete PID, the bias of the synergy scales with the number of bins as $(S \times R^2)/N$ and is much larger than the bias of redundancy, which scales as $(S \times R)/N$. For the Gaussian PID, the bias of the synergy also grows faster with the source dimension d than that of the redundancy. The uneven distribution of bias between synergy and redundancy is practically important because it implies that conclusions taken from limited empirical data without correcting for the bias may offer a

CellPress
OPEN ACCESS

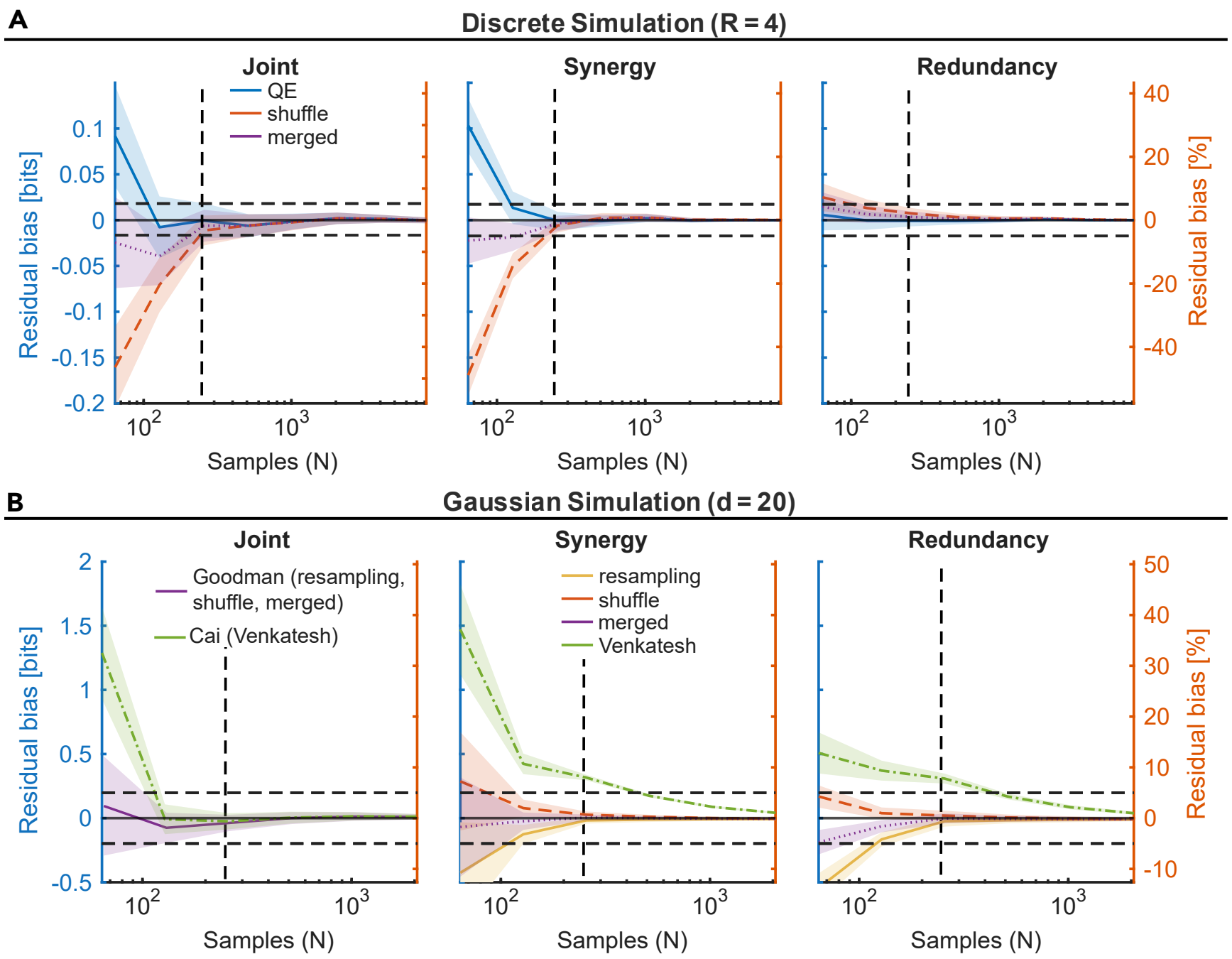

Figure 4. Residual bias after the implementation of different bias-correction methods

The residual bias (averaged bias-corrected empirical estimates from N samples minus ground-truth values) is plotted (on a log x axis) as a function of the number of samples N used to compute the joint information (left), the synergy (*Syn*; middle), and the redundancy (*Red*; right). The values of the residual bias are plotted in units of bits on the left y axis and as a percentage of the ground-truth joint information on the right y axis.

(A) Discrete BROJA PID simulations (number of bins $S = R = 4$) in the low information value regime. Corrections used for information quantities: QE, shuffle subtraction, and merged.

(B) Gaussian gPID simulations (number of dimensions d = 20) in the low information value regime. Corrections used for synergy and redundancy: resampling, shuffle subtraction, merged, and Venkatesh. For joint information, we used the Goodman correction[81] in the resampling, shuffle-subtraction, and merged methods as it is an exact correction, and for the Venkatesh correction we used the Cai et al. correction[80] to replicate what was done in Venkatesh et al.[30]

We plot mean ± 3 SEM over 100 simulations. Dashed vertical lines mark the boundary of the sufficient-sampling regime. Horizontal dashed lines are ±5% boundary lines. Discrete and Gaussian simulations were performed under the bit-of-all scenario (see methods and Table S2). For more details, see Table S1.

distorted view of information coding, artificially skewed toward synergy. For example, considering simulations with ground-truth values of redundancy larger than synergy, not correcting for the bias would lead to incorrectly estimating synergy larger than redundancy for low trial numbers (Figure S5A, third row).

Corrections for the limited-sampling bias of synergy and redundancy

We next consider how to correct synergy and redundancy for the limited-sampling bias. We first study the discrete PID, for which no previous bias correction was proposed. We introduce two complementary approaches (see methods for full implementation details). The first approach estimates the limited-sampling bias while preserving the information level of the real data. Based on the fact that (Figure 2A) the bias of each PID term decreases smoothly with the number of samples N, this approach (termed the quadratic extrapolation [QE] and introduced in Strong et al.[60] for Shannon information) fits a $1/N$ polynomial to PID terms recomputed on half and a quarter of the available data and uses this fit to estimate and remove the bias. The second approach ("shuffle subtraction") corrects for the bias by subtracting from the plugin values an estimate of the bias obtained by computing PID terms after randomly shuffling the source-target associations to remove all information about the target from the sources. This approach, which has been employed in the mutual information literature with numerous variations of how to destroy information by data randomization,[27,79,82] assumes implicitly that the bias has similar magnitude across information levels (an assumption that we will investigate below).

To estimate the effectiveness of the corrections, we plotted the residual bias (defined as the bias still present after applying a bias correction) as a function of the number of samples (Figure 4A), the number of discrete bins (Figure 5A), or the information level (Figure S8A). We find that both corrections reduce considerably the bias of the joint information and of the PID components (especially of the synergy, which is more biased). We consistently find across simulations that both QE and shuffle subtraction worked well for both synergy and redundancy as long as N is larger than $4 \times S \times R \times R$ (dashed vertical line, Figure 4A) or equivalently that $R < \sqrt{(N/4S)}$ (Figure 5A). We call this sampling condition the "sufficient-sampling regime for discrete PID." The good performance of these bias corrections within this regime was found across all simulation parameters and across all considered PID types (Figures 4A, 5A, and S3–S7). In the sufficient-sampling regime, the small residual error for synergy and joint information is mostly upward for QE (intuitively because bias terms of higher order in $1/N$, not included in the QE, are all positive[83]) and downward for the shuffle correction (because lower information levels have larger upward bias). This suggests that computing both QE and shuffle subtraction from real data provides reasonable empirical bounds to the information estimate for synergy. It also led us to implement a third and highly effective bias correction (termed the merged correction) that estimates the bias as a linear combination of the bias obtained with QE and shuffle subtraction, with weight optimized to minimize the absolute value of the residual bias on a simulation training set with many information levels (see methods). The merged correction is highly effective because it evens out the opposite tendencies of the QE and the shuffle subtraction (Figures 4A, 5A, and S8A). It leads to very small residual biases throughout the sufficient-sampling regime for both synergy and redundancy, and it still works well even in conditions of severe undersampling—well outside the sufficient-sampling regime. Comparing Figure 2A (left) with Figure 4A shows that not correcting for the bias would have led to large errors even in the sufficient-sampling regime and that to obtain, without using bias corrections, information measures as accurate as the ones corrected

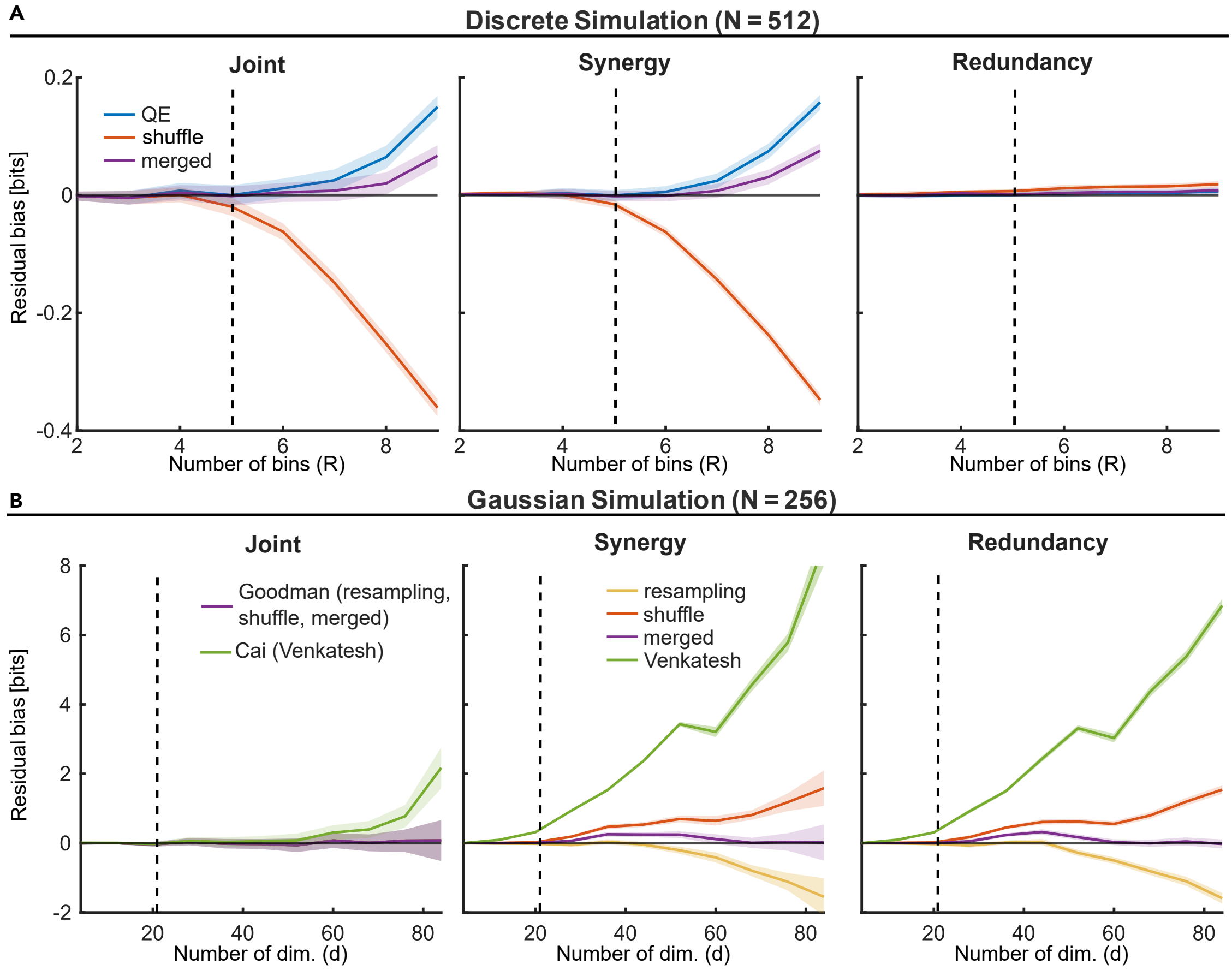

Figure 5. Performance of bias-correction methods as a function of the number of parameters defining the probability distributions

The residual bias (averaged bias-corrected empirical estimates from N samples minus ground-truth values) of joint information (left), synergy (*Syn*; middle), and redundancy (*Red*; right) after applying different correction techniques is plotted as a function of the number of parameters defining the probability distributions.

(A) Discrete BROJA PID simulations. We vary the number of discrete stimuli S and of discrete response bins for each neural unit R, with $S = R$ (number of samples $N = 512$). Corrections used for all information quantities: QE, shuffle subtraction, and merged.

(B) Gaussian gPID simulations. We vary the number of dimensions d of sources and targets ($N = 256$). Corrections used for synergy and redundancy: resampling, shuffle subtraction, merged, and Venkatesh. For joint information, we used the Goodman correction in the resampling, shuffle-subtraction, and merged methods as it is an exact correction, and for the Venkatesh correction we used the Cai et al. correction[80] to replicate what was done in Venkatesh et al.[30]

We plot mean ± 3 SEM over 100 simulations. Dashed vertical lines mark the boundary of the sufficient-sampling regime. Discrete and Gaussian simulations were performed under the bit-of-all scenario (see methods and Table S2). For more details, see Table S1.

with the merged correction would have needed orders-of-magnitude more samples. For a fixed number of samples, using any of the bias corrections, but especially the merged correction, extends considerably the range of discrete number of bins for which information can be estimated accurately. For example, when using $N = 512$ samples, information estimates remain accurate (small residual bias) up to six discrete bins (Figure 5A), whereas not using a bias correction would have led to small residual errors only when using two discrete bins (Figure 2A). These findings hold also for the Unique information (Figure S2). Finally, when applying the merged correction and the other corrections in the sufficient-sampling regime, accurate estimates with small residual biases are obtained regardless of the information level (Figure S8A), highlighting the robustness of the results.

In sum, for the discrete PID we recommend the use of the merged bias correction, which gives precise results for both synergy and redundancy in the sufficient-sampling regime and often beyond. Using this bias correction leads to much more precise estimates than using no bias corrections and extends the range of applicability of the method to larger numbers of bins and smaller number of samples. To help users, we computed numerically an estimate of the tolerance (estimated magnitude of the absolute value of the residual bias; Figure S15) within the sufficient-sampling regime as a function of the fraction R^3/N (the parameter ratio setting the scaling of the bias of the synergy). This estimate of the residual bias helps in both designing the experiments (setting the minimal number of samples needed for a given tolerance) and designing analyses (setting the maximal number of bins that, given the number of samples, would allow being in the sufficient-sampling regime and achieving errors within a chosen tolerance).

We then consider the Gaussian PID. For this, one bias-correction procedure was previously proposed,[30] which we term the Venkatesh correction. The Venkatesh correction assumes that

the union information has the same proportional bias as the joint information (and thus of the synergy, see methods), and it then rescales all PID accordingly (Equations 3, 4, and 5). However, we found that the union information is much less biased upward than the joint information (Figure 3). Thus, this procedure is expected to lead often to a major underestimation of union information, which in turn leads to a severe overestimation of synergy and redundancy (see methods), which persists also for large N. In our simulations, the Venkatesh bias correction left a residual error that could be as big as 30%–40% of the true joint information value (Figures 4B, 5B, S9, and S10). We thus introduce two alternative correction procedures that, as in the discrete case, either operate with information levels equal to those of the data or operate by removing all information from the data. The first correction, termed resampling correction, estimates the bias as the difference between the mean information value obtained by repeatedly generating N samples from the distribution estimated from the real data and the information value computed from the PID equations using the covariance matrix estimated from the real data. The resampling correction works well for the Gaussian but less so for the discrete PID, because the overall bias of the joint Gaussian information does not depend much on the information level (Figures S9 and S10). We chose it to replace the QE (used in the discrete PID) for the Gaussian PID, as we verified that QE does not work well for the Gaussian information because its bias is non-polynomial.[81] The second is the shuffle correction, obtained, exactly as for the discrete PID, by estimating the bias on shuffled data and subtracting it. Both corrections reduce by orders of magnitude the bias of the joint information and of synergy and redundancy, leaving a residual bias that was just a small fraction of the uncorrected bias (Figures 4B, 5B, S3, S8B, S9, and S10). As expected intuitively, the shuffle correction works better than resampling for lower but not for higher information levels (Figure S10). Because of the above-described dependence of the bias of the PID components on the information level (Figure 2), the small residual error for synergy and redundancy information was typically downward for the resampling correction and typically upward for the shuffle correction. Because of the opposite trend of their residual errors, we also averaged the resample and shuffle corrections to obtain a merged correction, which further reduces the residual bias.

Across simulations, the merged, resampling, and shuffle corrections consistently leave a negligible residual error for $N > 4 \times 3d$ or $d < N/12$ (Figures 4B, 5B, S3, S8B, S9, S10, and S11), a condition that we term the "sufficient-sampling regime for Gaussian PID." These findings hold also for the Unique information (Figure S2). All our Gaussian bias corrections improve considerably the existing Venkatesh correction.[30] Also in the Gaussian case, the merged correction is the correction that works better and provides accurate estimates (small residual biases) even for numbers of samples or dimension values well outside the sufficient-sampling regime. In Figure 5, the correction left residual errors of the order of 0.1 bits or smaller against original uncorrected bias values that were of the order of several bits. We thus recommend the use of this merged correction for the Gaussian PID. The merged correction extends the applicability of the PID to much smaller sample sizes and a much higher number of dimensions compared to uncorrected estimates and to the previous available Venkatesh correction (Figure 5B). As for the discrete PID, we provide in Figure S15 a numerical evaluation of the absolute value of the residual error expected as a function of the fraction $3d/N$ (which is expected to set the synergy bias from analytical considerations). This allows users to design numbers of samples or trials and set dimensionalities that can be analyzed given a chosen level of tolerance in residual errors.

Evaluation of bias-correction procedures on real neural data

We evaluated the empirical utility of the PID bias correction using four datasets from previously published studies, which span different species, recording modalities, and cognitive-task designs.

We first consider three datasets of spiking activity recorded from animals performing cognitive tasks (see methods for full experimental details). For all datasets, analysis parameters and trial numbers meet the sufficient-sampling regime. We use discrete PID because it is natural for spiking activity and discrete stimuli and because Gaussian PID gave extremely low information values for two of these datasets (Figure S13 and Note S5).

Dataset 1 (Figure 6A) consists of n = 6,209 pairs of neurons (mean number of trials per session: 140) simultaneously recorded with two-photon calcium imaging from auditory cortex (A1) during a sound intensity discrimination task.[36] We compute the information that neurons carry about the intensity of the presented sound (a binary variable, S = 2, high vs. low tone intensity). Each neuron's spiking activity is estimated by deconvolving the calcium fluorescence traces. The single-neuron response variables r_1 and r_2 are computed by summing the deconvolved activity within a 333-ms time window centered around the time of maximal information and discretizing it into R = 3 bins[36] (thus, one sample per trial is collected).

Dataset 2 (Figure 6B) consists of n = 36,158 pairs of neurons simultaneously recorded with two-photon calcium imaging from the CA1 region of the hippocampus[63] while mice navigated a linear track in virtual reality. Because the hippocampus encodes spatial position, we compute the information that neurons carry about the spatial location (linear track discretized in S = 12 spatial bins with on average 870 trials per session). The spiking activity for r_1 and r_2 is obtained, following Curreli et al.,[63] discretizing the fluorescence traces into R = 2 bins (low vs. high activity). For this dataset, we had 77–125 samples per spatial location bin.

Dataset 3[11] (Figure 6C) consists of n = 83 pairs of neurons simultaneously recorded with electrodes from the auditory cortex (A1) of a macaque monkey listening to natural sounds. We compute information carried by neural activity r_1, r_2 (defined as spike counts discretized in R = 2 bins) concerning which of S = 399 different sound stimuli (segments of natural sounds each 50 ms long) were presented (mean number of trials per session: 108 for each of the 399 sound stimuli; one sample of neural activity per trial was collected).

We compute the joint information between the above-defined activity r_1, r_2 of simultaneously recorded pairs of neurons (PID sources) and the above-defined stimulus s (PID target). We used the discrete BROJA PID,[33,84] as spike responses are better described with discrete distributions, and the stimuli also had discrete values. We focus the analysis and the neuroscientific interpretation on the differences between synergy and redundancy to understand how these two emergent properties shape

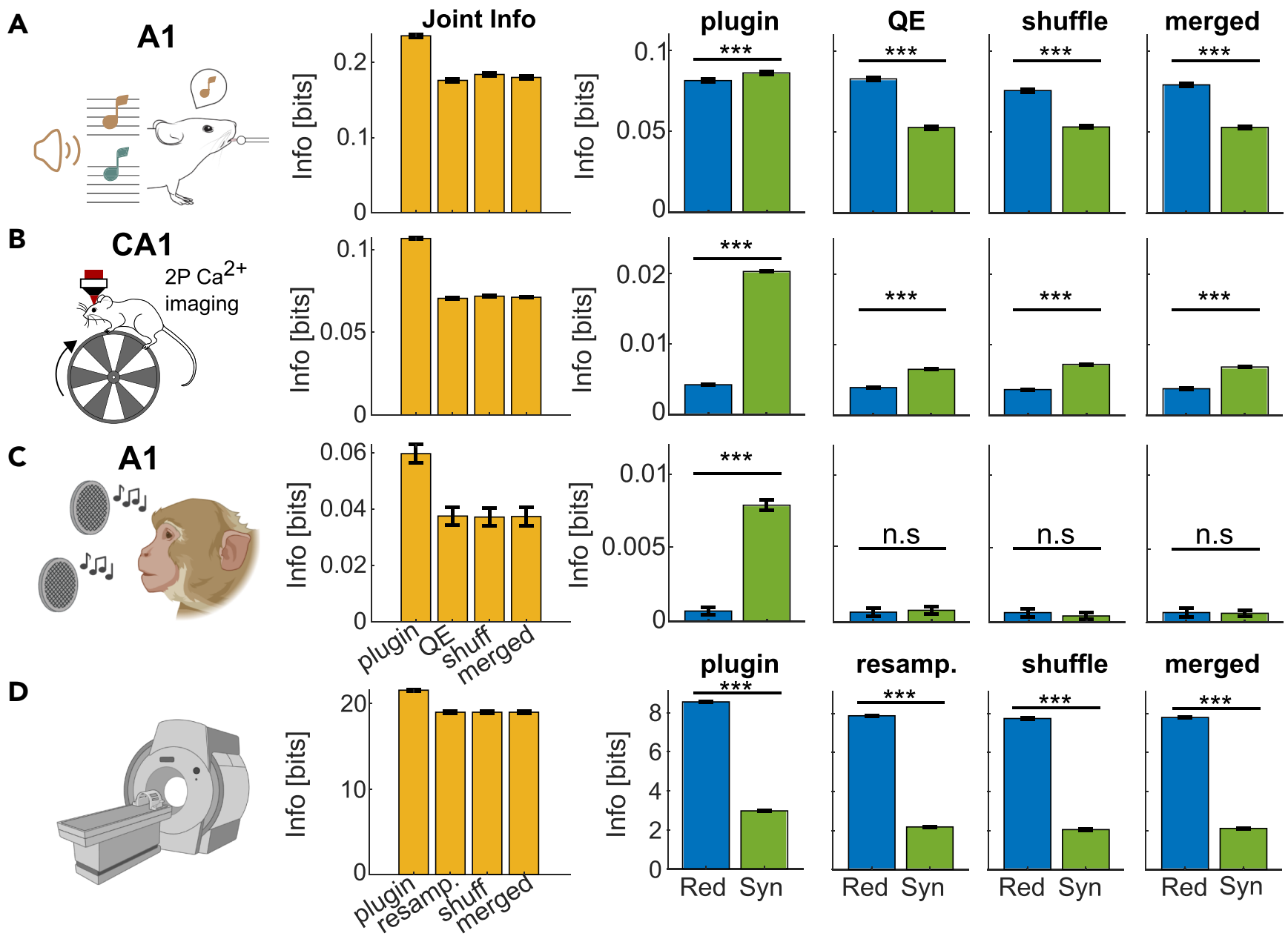

Figure 6. Application of PID bias corrections to real neural data

(A–C) Reporting the discrete BROJA PID of the joint information between an external stimulus (S, target) and the joint activity of two neurons (sources). In (A) (dataset 1), S is the identity of a binary auditory tone, and neuronal responses are spike trains obtained by deconvolving calcium signals recorded from mouse auditory cortex A1 (spike counts binned in $R = 3$ levels). In (B) (dataset 2), S is the mouse location (discretized in 12 spatial bins) on a linear track, and neuronal responses are calcium signals recorded from the CA1 hippocampal region (discretized in $R = 2$ bins). In (C) (dataset 3), S is the identity of one of 399 natural sounds, and neuronal responses are electrophysiologically recorded spike trains from monkey A1 (spike counts binned in $R = 2$ levels).

(D) Reporting the Gaussian gPID of the joint information between the activity of one putative input brain region at a given time (target) and the activity of two other brain regions (sources) at an earlier time (dataset 4).

Each panel shows mean ± 3 SEM of information values over all analyzed simultaneously recorded neural pairs (n = 6,209, 36,158, and 83 for (A), (B), and (C), respectively) or triples of brain regions (D, n = 15,000 triplets of sets of 20 ROIs randomly drawn among 1,000 ROIs). The mean number N of available samples per session is 140, 870, and 43,092 for datasets 1–3, respectively. For dataset 4, information values are computed across 256 samples (different time points). Left to right: schematic of each experiment; joint information results with plugin (no correction) or different bias-corrected estimators QE (resample for fMRI), shuffle subtraction, and merged; redundancy and synergy results with plugin or bias-corrected estimators. Comparisons between synergy and redundancy were performed with a two-tailed paired t test (***$p < 0.001$; n.s., $p > 0.05$). Details are provided in methods. Image credits: (A) mouse sketch adapted from https://doi.org/10.5281/zenodo.3925985, speaker icon from svgrepo.com, and music note icon from svgrepo.com, all modified and licensed under CC BY 4.0; (B) created by the authors; (C) created by the authors in BioRender; and (D) created by the authors in BioRender.

neural population coding in different brain regions. To test and exemplify the use of the bias-corrected PID algorithms, we compare the plugin PID estimates with those obtained after applying our three bias corrections (Figures 6A–6C).

As shown by the smaller values obtained after correcting for the bias, the plugin (uncorrected) joint information is biased upward by ≈30%–50% (Figures 6A–6C). As predicted by our simulations, redundancy is almost unbiased (shown by the fact that it does not change value after bias corrections; Figures 6A–6C). Despite these datasets having a good number of trials compared to typical experiments, the synergy bias is major, as the difference between bias-corrected and plugin synergy estimates is approximately 60%, 200%, and 800% of the target values across the three datasets (Figures 6A–6C). Using the uncorrected estimator would have thus led to a considerable overestimation of the synergy and to errors in conclusions. In dataset 1 (Figure 6A), using the uncorrected plugin estimator would have led to the erroneous conclusion that synergy was larger than redundancy, whereas all three bias-corrected estimates consistently detected that redundancy was higher than synergy. In datasets 2 and 3 (Figures 6B and 6C), using uncorrected estimates would have led to a very major overestimation of the real importance of synergy, and in dataset 3 it would have led to the wrong conclusions that synergy was higher than redundancy. Importantly and reassuringly, estimates of both redundancy and synergy were highly consistent across methods within each dataset (Figures 6A–6C), suggesting that these estimates on real data are precise and unbiased. Moreover, the fact that the synergy obtained with the conservative shuffle subtraction is positive proves that there is genuine synergy in all datasets that cannot be due to finite sampling artifacts. Further, when using bias corrections (but not when using uncorrected estimates), results are almost identical when running the analysis on half of the datasets (Figure S16), further confirming the robustness of the bias corrections.

To test our methods on non-spiking, continuous-valued measures of neural activity, we next consider dataset 4 (Figure 6D), consisting of fMRI signals collected from n = 100 human participants at rest.[85–89] To evaluate whether different brain regions interact synergistically or redundantly, we compute the information about the fMRI signal value in a set of d regions of interest (ROIs) (PID target) found one imaging timeframe later in the fMRI signal of two d-dimensional sets of other (source) fMRI ROIs (Figure 6D). Established analyses of PID of information transfer across brain regions are often performed with Gaussian PID by considering regions as one-dimensional sources/targets (d = 1).[49,51,90] As we show in Figure S17, these low-dimensional Gaussian estimates of redundant and synergistic interactions across brain regions have negligible bias. Here, we investigate whether it is possible to extend low-dimensional Gaussian PID analyses of synergistic and redundant interactions to high-dimensional brain regions using the Gaussian gPID with our bias corrections. We focus on information interactions measured across a number of samples (that is, time points) that falls within the range common in the fMRI literature (256 imaging frames[91,92]) and that can also be realistically collected in clinical settings. When considering large-dimensional sets of ROIs (d = 20), the bias of synergy estimation is a substantial fraction (≈35%) of the target value, but it was well corrected (as

suggested by the fact that all our bias corrections give consistent results). Redundancy is much less biased (10% of the target value) and also well corrected for (Figure 6D). The Venkatesh correction fails to remove a large part of the synergy and redundancy bias, as predicted by the theoretical and simulation results (Figure S17). Results using the maximum available number of time points (1,189), showing a still significant but much lower bias, are reported in Figure S17. Altogether, this application suggests that our bias corrections are useful to extend the Gaussian estimation of synergistic and redundant interactions to distributed multidimensional brain networks, even with a limited number of samples at different time points, as is common in neuroimaging (especially for clinical applications).[91,92]

The bias of three-source PID

While the two-source PID provides valuable insights into pairwise neural interactions, information encoding often involves higher-order interactions among larger ensembles of neural units.[90,93–96] Therefore, developing PID with more than two sources applicable to empirical neural data is of importance because it can capture synergy and redundancy beyond second order.

We thus analyze the bias of the PID with three sources ($\vec{R} = (R_1, R_2, R_3)$) and one target ($S$). Although neither the discrete BROJA PID[33] nor its Gaussian counterpart gPID[30] have been defined for more than two sources as yet, other PIDs such as I_{min} and I_{MMI} have been defined for, and can be readily applied to, more than two sources. Here, we study the bias properties of the MMI with three sources. We focus on two components with straightforward interpretation. The first is three-way redundancy, which quantifies the information that each of the sources R_1, R_2, and R_3 encode about S (defined in the MMI PID as the minimum among the single-source information that each source encodes about the target). The second is three-way synergy, which quantifies the information about S that is available only from the joint observation of (R_1, R_2, R_3) and not from any individual source or pair of sources (defined in the MMI PID as the joint information that the three sources encode about the target minus the maximum information carried by any pair of sources[97,98]).

As we did above for the two-source PID, we studied numerically the bias of both the discrete and Gaussian three-source MMI PID using simulations of discrete and Gaussian processes, respectively, whose ground-truth values of joint information and of three-way synergy and redundancy can be computed from the exact probabilities used to simulate the data. Namely, for the discrete case we simulated a 3-bit parity system with purely ground-truth synergistic information, and for the Gaussian case we simulated a system with a ground-truth covariance matrix having only redundancy (see methods). Again, exactly as for the above two-source simulations, in this case also we computed the bias as the difference between the plugin estimate empirically obtained with a certain number of trials and the corresponding ground-truth value. We find that on simulated data, the bias of the three-way synergy is far larger than the bias of the three-way redundancy, with a larger gap than that found for the synergy and redundancy obtained with the two-source PID (Figure 7) for both discrete and Gaussian PID. This is because (see methods) the bias of the most biased terms of the three-way synergy scales like the bias of the joint information carried by three sources (which scales as $(S \times R^3)/N$ for the discrete PID and as a supralinear function of $4d$ terms for the Gaussian PID), whereas the three-way redundancy has terms with bias scaling as the bias of the single-source information (which scales as $(S \times R)/N$ for the discrete PID and as a sum of digamma functions with up to $2d$ terms for the Gaussian PID). We tested the effectiveness on this three-source PID of the various bias corrections that we developed above (Figure 7, lower rows). The bias-correction procedures appear to be highly effective also for the three-source PID.

We evaluated the bias corrections for the three-source PID on an empirical MEG dataset[77] (dataset 5) collected from human participants performing a decision-making task requiring them to estimate, from several successive samples of a visual stimulus, the generative mean of the angle specifying the position of the visual stimulus. We computed the information encoded by broadband MEG activity recorded across multiple spatial locations (the sources) about the angle of the first sample shown (the target). We thus considered visual stimulus encoding in neural activity. An important property of this dataset is that both the sources (MEG activity) and the target (presented orientation of the visual stimulus) follow approximately a Gaussian distribution (see methods), and thus both the discrete and the Gaussian PID can be meaningfully computed. Between $N = 519$ and $N = 864$ trials were available for each of $n = 30$ human participants, and one sample of neural activity per trial was collected.

We first considered the application of the discrete PID to MEG. Because the discrete PID does not scale easily to sources with many dimensions, we used it to address a question about encoding within a single anatomical group. Specifically, we studied the encoding of the visual stimulus orientation across three areas of the visual system (V1, V2, and V3). The activity of each region was a one-dimensional signal discretized into $R = 3$ equipopulated bins. The visual stimulus was also discretized into $S = 3$ bins, similarly to the original study.[77] Since in this dataset MEG activity was recorded both before and after stimulus presentation, we used pre-stimulus MEG activity (for which the ground-truth value of joint information about the stimulus, and its synergy and redundancy, must all be zero) as a first important test of the bias-correction performance. With the plugin (uncorrected) estimator (Figure 8A, left), both joint information and synergy values were relatively large, indicating a significant bias. Application of our bias corrections successfully removed the spurious pre-stimulus information values, and all corrections gave consistent results. We then considered post-stimulus activity (Figure 8A, top right). The magnitude of the bias removed from post-stimulus estimates was comparable to, but smaller than, that observed in the pre-stimulus activity (compatible with the above theoretical predictions that the bias of synergy and joint information is larger for lower information values in the discrete case). All corrections gave consistent results, and the amount of bias subtracted by the corrections was a large fraction of the value of the resulting quantities after bias subtraction (146% and 1,669% for joint information and synergy, respectively). Conclusions based on plugin estimates would have led to the wrong conclusion that in this system both synergy and redundancy are present, with synergy larger than redundancy, whereas after bias corrections it becomes clear that both synergy and redundancy are small and neither reaches significance. Since in this dataset we could apply both discrete and Gaussian PID, we next applied the

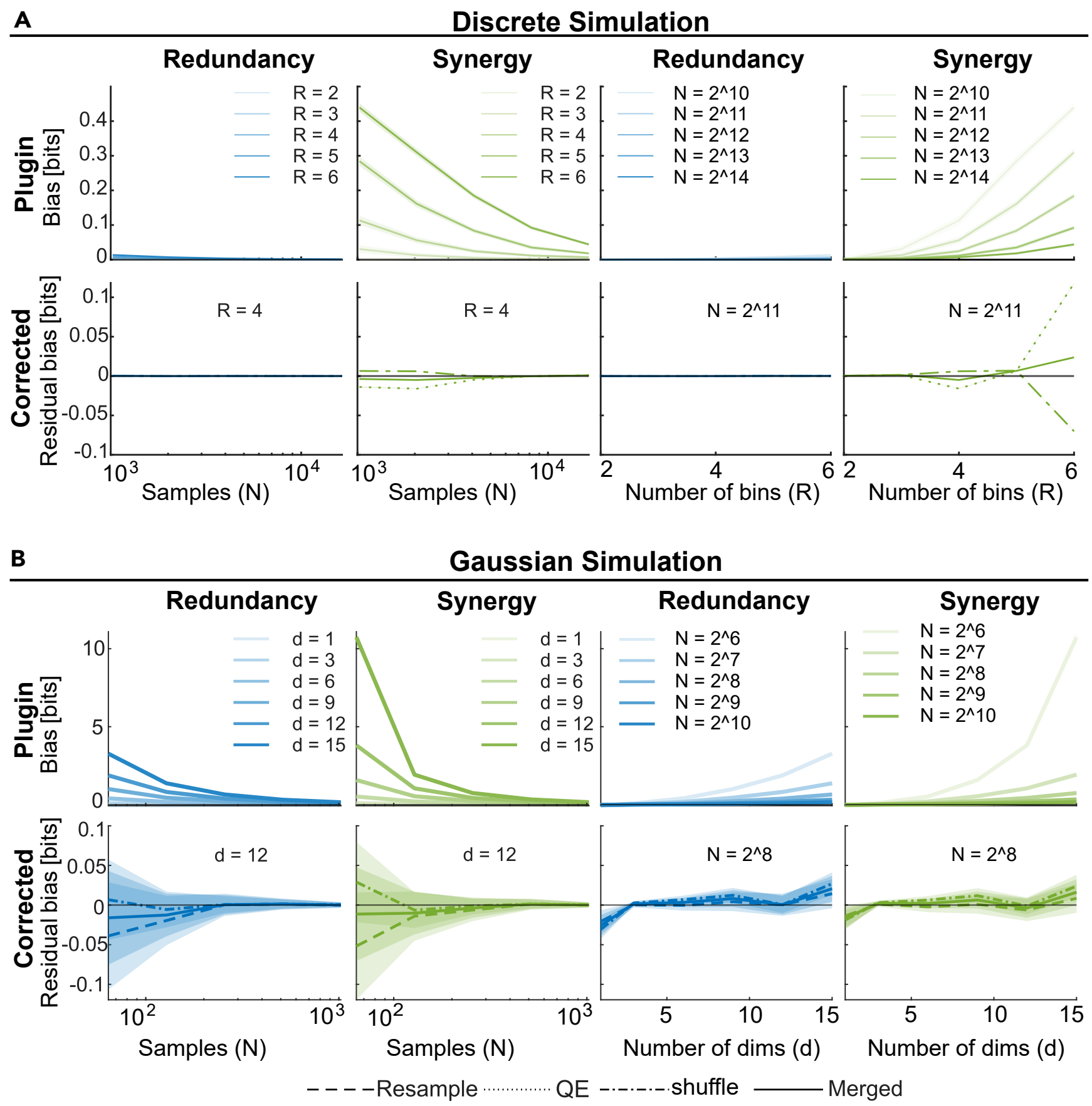

Figure 7. Simulations of the bias of the three-source PID

Plots show on the top row the bias (averaged empirical estimates from N samples minus ground-truth values) of the plugin (i.e., not bias-corrected) estimate and on the bottom row the residual bias (averaged bias-corrected empirical estimates minus ground truth) of the bias-corrected three-way synergy and three-way redundancy as a function of number of samples or number of parameters defining the probability distributions.

(A) Discrete MMI PID simulations. Top: the bias of plugin information as a function of the number of samples N (left, on a log x axis; each line corresponds to a fixed number of bins R) or the number of bins R (right; each line corresponds to a fixed N) used to compute PID. Bottom: the residual bias of bias-corrected PID for different correction methods (QE, shuffle subtraction, and merged) as a function of the number of samples N (left, $R = 4$ on a log x axis) or the number of bins R (right, $N =$ 2,048). In all simulations in this panel, we set $S = R$.

(B) Gaussian MMI PID simulations. Top: same as top row of (A), but considering the dimension of sources/target d instead of R. Bottom: same as row of (A), but showing the residual bias for resample, shuffle-subtraction, and merged corrections. $d = 12$ when plotting the residual bias vs. N (left, on a log x axis); $N = 256$ when plotting the residual bias vs. d (right).

We plot mean ± 3 SEM over 100 simulations. Details are provided in methods and Table S1.

Gaussian MMI PID to the same set of one-dimensional sources (Figure 8B). The Gaussian PID in the one-dimensional sources had very small bias, as shown by the fact that the plugin (uncorrected) and bias-subtracted estimators have very similar values. Bias-corrected estimates were very similar between the discrete and Gaussian PID when using bias corrections, indicating that the bias corrections provide reliable and uninflated values for both the discrete and Gaussian PID.

We then considered the highly multivariate problem of how the simultaneous activity of many different brain areas across different anatomical groups jointly encodes information about the visual stimulus. This question cannot be addressed by the discrete PID (as it does not scale to very large multivariate analyses) and, to our knowledge, has not yet been empirically addressed in the literature. Given the small bias of the Gaussian PID on one-dimensional sources shown above and given the promising performance of the bias-correction estimators for the Gaussian three-source PID on the above simulated data, we addressed this multivariate coding problem with the Gaussian PID. Namely, we used the Gaussian MMI[34] of joint information between the stimulus orientation (target) and the activity of three large-scale, multivariate sources (the joint activity of all 26 areas in the visual cortex, all 47 areas in the prefrontal cortex, and all 25 areas in the somatosensory and motor cortex). From pre-stimulus MEG activity with the plugin (uncorrected) estimator (Figure 8C, bottom left), both joint information and synergy and redundancy values were relatively large (approximately 0.1 and 0.03 bits, respectively), indicating a significant bias and proving that this question could not be addressed without bias-correction techniques even when making Gaussian approximations. Application of our bias corrections successfully removed the spurious pre-stimulus information values, and all corrections gave consistent results. From post-stimulus activity (Figure 8C, bottom right), the bias corrections removed from the plugin estimates biases that were similar in size to those identified in pre-stimulus activity (compatible with the above theoretical predictions). All corrections gave consistent results, and the amount of bias subtracted by the corrections was a large fraction of the value of the corresponding quantities after bias subtraction. After bias corrections, the PID reveals significant redundancy between the activity of the anatomical groups but no or little synergy.

Together, these results confirm that bias corrections are both necessary and highly effective for three-source PID and that their use allows formulating and addressing neuroscientific questions about multisource encoding that would be out of reach without bias corrections.

DISCUSSION

We investigated thoroughly, with analyses of simulated and real neuroscientific data and with analytical considerations, how

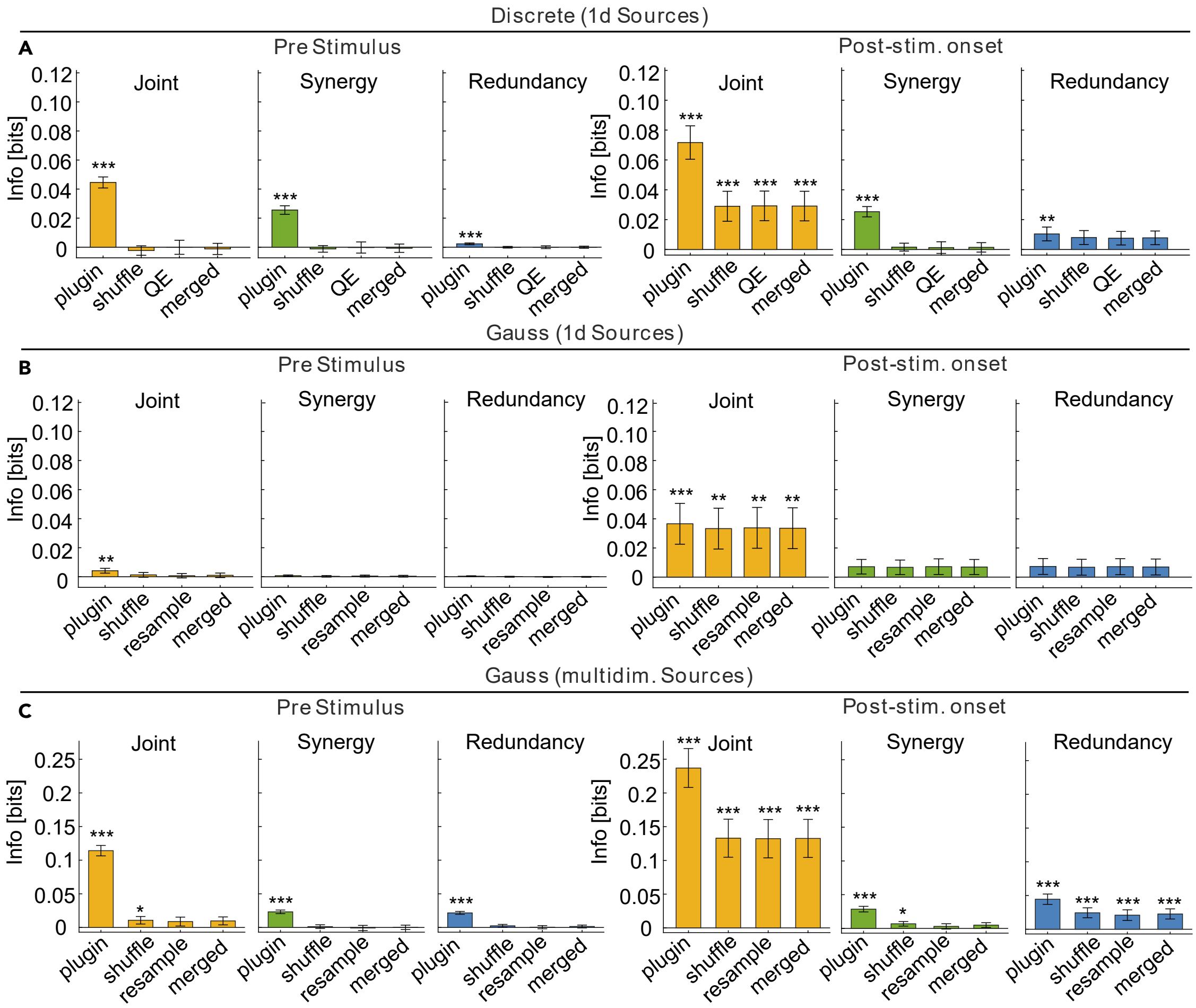

Figure 8. Application of PID bias corrections to an empirical MEG dataset (dataset 5) analyzed with the three-source PID

Panels report the joint, redundant, and synergistic information that triplets of sources (MEG activity of one or multiple brain areas) carry about a target (the angle specifying the position of the first visual sample presented at the beginning of each trial).

(A) Discrete MMI PID. The three sources are the three visual areas V1, V2, and V3. Bias-correction methods: plugin (no correction), shuffle subtraction, QE, and merged.

(B) Gaussian MMI PID with one-dimensional sources. The three sources are the three visual areas V1, V2, and V3. Bias-correction methods: plugin (no correction), shuffle subtraction, resample, and merged.

(C) Gaussian MMI PID. The three large-scale, multivariate sources are the joint MEG activity of all areas within the visual cortex, the prefrontal cortex, or the somatosensory and motor cortex, respectively. Bias-correction methods: plugin (no correction), shuffle subtraction, resample, and merged.

In all panels, we used MEG activity recorded either (left) during a pre-stimulus time window of length 150 ms preceding the stimulus onset or (right) in a 150-ms window post stimulus onset. Significance against zero was assessed with a one-sample t test across participants (*$p < 0.05$, **$p < 0.01$, ***$p < 0.001$). We plot mean ± 3 SEM over 30 participants. Details are provided in methods.

limited-sampling bias affects empirical measures of synergy and redundancy across various types of PID. We found that synergy, and to a lesser extent redundancy, suffer from a considerable limited-sampling bias. Furthermore, we observed that the synergy bias (and to a lesser extent the redundancy bias) grows rapidly with the dimensionality of the problem under investigation (related to either the number of sources used or the number of dimensions used to describe each source) and that it is often large enough to be comparable to or larger than the target values of the quantities. Thus, the bias problem cannot be neglected in real-data neuroscience applications. If uncorrected, the bias problem can artificially lead to inaccurate estimates and skew comparisons in favor of synergy. Our work highlights and explains the presence of a major and previously neglected difference in the sampling bias between synergy and redundancy, which can inflate synergy disproportionately. Neural synergy has been widely reported in recent years and has led neuroscientists to rethink how the brain integrates information.[23] Our results call for a careful evaluation of synergy in neuroscience using suitable bias-correction methods.

Our work is immediately relevant to empirical neuroscience. First, it empowers experimenters with bias-correction methods that greatly improve PID estimates with respect to uncorrected measures or other available methods.[30] The most effective bias-correction method is what we termed the merged correction, which combines linearly two different bias-correction

methods with opposite tendencies for residual errors. We thus recommend the application of the merged correction for practical applications. As confirmed by our extensive real-data analyses, these bias-correction methods are generally applicable to a wide range of experimental situations and types of brain recordings, crucially encompassing both discrete and continuous cases. As shown by our applications to real and simulated data, our bias-correction methods reduce by orders of magnitude the number of samples needed to reach a certain estimation precision or the magnitude of the residual errors in PID estimation from small numbers of samples.

The second direct empirical relevance of our work is that it informs the design of experiments. This is because it provides an understanding of the bias in terms of simple properties of experimental design (number of trials or number of time points that determine the sample size). The rule of thumb is that the information estimation is accurate (with a small tolerance value that can be easily estimated from the data) in the so-called sufficient-sampling regime. For discrete PID, the sufficient-sampling regime requires that the number of samples N satisfy $N > 4 \times S \times R^2$, where R and S are the number of possible discrete values for the sources and target, respectively. For the Gaussian PID, the sufficient-sampling regime requires that the number of samples N satisfy $N > 12d$, where d is the dimension of the sources and the target.

Third, our work informs the choices to be made for the analysis setting. It shows that discrete rather than Gaussian PID should be used for spiking activity and/or for experiments with discrete stimuli set. It then informs (using the same rules of thumb set above) about the range of number of bins or the range of the number of dimensions that can be used given the collected number of samples.

Our simulations and analytical considerations were accompanied by a thorough validation of the performance of our bias-correction algorithms on five different neuroscientific datasets. This analysis confirms the effectiveness of the bias-correction procedures and demonstrates that they are essential for empirical studies. Bias-uncorrected estimates would lead to highly imprecise and possibly misleading conclusions about neural synergy. In most analyzed conditions, the majority (or at least a large part) of the synergy computed without bias corrections (plugin estimator) reflected just the limited-sampling bias and not real neural synergy. Thus, bias corrections should become a routine part of PID analyses of neuroscientific data, as they are now for traditional application of Shannon information theory to neural coding.[27]

Although the work presented here is primarily methodological, in testing our methods on empirical neuroscientific data we provide extensive knowledge about the distribution of synergy and redundancy in the brain. Most PID analyses have been applied to aggregate measures of neural activity such as fMRI. We extend this bulk of work by providing an extensive study of information encoding in real neural spiking activity (the fundamental level of brain information processing) across species and cortical areas. Our systematic analysis of the information carried by tens of thousands of pairs of neurons across different cortical areas revealed widespread presence of both synergy and redundancy across brain areas and species in neuron-to-neuron interactions, with regional variations in the prevalence of synergy vs. redundancy. Moreover, our analyses of real fMRI recordings with Gaussian PID confirm that previous reports of synergy in cross-area brain interactions are genuine and not due to biased estimates,[23,49,95,99] and they show that bias corrections can improve the accuracy of interaction analyses with data sizes typical of clinical applications. Our work also reported an MEG-based multivariate analysis of sensory information coding across multiple brain regions in different anatomical groups, which reveals the presence of higher-order redundancy in stimulus representations within and across cortical groups.

Importantly, although we focused our applications on neuroscience data, our work could be readily applied more widely, as PID using the discrete or Gaussian estimators studied here are often employed in real-data analyses in non-neuroscience fields, such as the analysis of gene-regulatory networks, the selection of features in machine learning, the analysis of generative neural networks, or the analysis of financial data.[100–103]

Despite our thorough study of several types of PID and our extensive testing and validation with many simulated scenarios and multiple neural datasets, our work has limitations requiring further theory and simulations, including the following. First, we tested the bias of BROJA, I_{min}, and I_{MMI} PIDs, which lend confidence to the generality of our results. However, it would be important to test others. For example, it would be important to test how our bias corrections and the prediction that synergy is more biased than redundancy hold across different PIDs including, for example, PIDs for continuous but non-Gaussian distributions.[44] Because our bias corrections are derived without making assumptions on the underlying distributions, we hypothesize that they would be effective across PIDs including those not designed for Gaussian distributions. Because our analytical considerations predicting a higher bias for synergy than redundancy are based on the assumption that the synergy depends on the full joint distributions and the redundancy depends on the marginal but not the full joint distributions, we expect that these predictions hold for the several PIDs that respect this pairwise marginals property, such as those reported previously.[22,30,34,41,44,97] To facilitate testing the wider applicability of our bias-correction methods, we provide open-source modular software implementation of these algorithms, which easily allows the testing of new PIDs. Second, the bias-correction procedures are heuristic, although we support them with analytical considerations. However, we did not provide theoretical guarantees of the sign of residual errors of different bias-correction procedures. Third, when considering spiking activity at the cellular level (which requires discrete PID), we tested synergy and redundancy between pairs and triplets of neural units, but we have not systematically tested large populations. The direct calculation of information is very precise for small populations, but it does not scale up with population size.[27] However, our discrete methods could be scaled up by coupling them with dimensionality-reduction techniques that are often used in studies of neural population codes.[13,63,104–108] We provide an initial analysis of discrete PID of population spiking activity in which each source consists of a population of ten simultaneously recorded neurons encoding stimulus information, and their high-dimensional activity is reduced with a support vector machine and then discretized (Note S7). Encouragingly, we found that our bias corrections provide accurate estimates of population

redundancy and synergy even for small trial numbers (Note S7 and Figure S18). It remains to be explored how best to couple PIDs with dimensionality reduction to scale up analyses to large populations. Fourth, some methods proposed for mutual information[28] and then extended to PID[31] do not correct for the bias but introduce into the estimator terms designed to compensate for predicted differences in bias across different entropy terms. It would be useful to study in further work whether and how bias-correction and compensatory procedures could be combined to optimize PID estimates from limited samples.

In conclusion, our systematic analysis of the limited-sampling bias of PID analyses reveals that synergy is far more biased than redundancy, that in practical applications the bias may be as large as the target information values, and that this problem can artificially skew comparisons of synergy and redundancy in the brain. We also developed a robust method for correcting for the bias, which significantly reduces the number of samples needed for accurate estimates. This extends the applicability of PID to, for example, clinical rather than laboratory settings. These bias corrections also greatly extend the dimensionality of the problems that can be accurately investigated empirically, allowing, for example, highly multivariate analyses of synergy and redundancy across many brain regions. Because of the aforementioned reasons, our work suggests that correcting for the limited-sampling bias should become standard practice in the analysis of empirical data with PID.

METHODS

Definitions of Shannon information and PID

We first provide definitions of Shannon information quantities, including entropy and mutual information, and describe relationships to compute redundant and unique information as linear combinations of Shannon quantities and synergy. We then introduce different PID measures that have been proposed and that are relevant for this paper for both the discrete and the Gaussian PID.

Shannon information and relationships with PID components

Here, to make the paper more self-consistent we provide a more extensive summary of definitions of Shannon information quantities relevant for this study, and we describe relationships to compute all PID components as linear combinations of Shannon quantities and the synergy $Syn(S{:}R_1;R_2)$ (defined in Equation 7 for the BROJA measure).

The foundational quantity in Shannon information theory is entropy, which quantifies the amount of uncertainty associated with a probability distribution $p(X_1, \ldots, X_M)$, where each X_i is a possibly multivariate stochastic variable of dimension d_i (e.g., the activity of one or several neural units). $D = \sum_{i=1}^{M} d_i$ denotes the total dimensions of the system. The entropy of a discrete distribution $p(X_1, \ldots, X_M)$ is defined as[38,39]

$$H(X_1, \ldots, X_M) = -\sum_{x_1 \in X_1, \ldots, x_M \in X_M} p(x_1, \ldots, x_M) \log_2(p(x_1, \ldots, x_M)), \quad \text{(Equation 8)}$$

where the sum spans all possible combinations of values ($X_1 = x_1, \ldots, X_M = x_M$) in the D-dimensional probability space. The base-2 logarithm in Equation 8 implies that entropy is measured in bits. (Note that the base of the logarithm merely acts as a multiplicative term independent of the sampled probabilities. In case of a base change, both the actual information value and the value of the information bias will be rescaled by the same multiplicative factor expressing the base change).

In the case in which full probability distributions take the form of (or are approximated by) a continuous Gaussian distribution p_G with covariance matrix $\Sigma(X_1, \ldots, X_M)$ of dimensions $D \times D$ (capturing the second-order statistics of the original distribution), the sum in Equation 8 becomes a D-dimensional integral over continuous variables, with a closed-form solution[39]:

$$\begin{aligned} H(X_1, \ldots, X_M) &= -\int_{x_1, \ldots, x_M} p_G(x_1, \ldots, x_M) \log_2(p_G(x_1, \ldots, x_M)) \\ &= \frac{1}{2} \log_2\left((2\pi e)^D \det(\Sigma(X_1, \ldots, X_M))\right), \end{aligned} \quad \text{(Equation 9)}$$

where each variable X_i is integrated over its d_i-dimensional support. All the information theoretical quantities defined in the paper as sums and differences of entropy terms can be equivalently expressed in the discrete case by substituting entropy terms with the definition provided in Equation 8 or in the Gaussian case by using the definition in Equation 9.

In this paper, we consider probability distributions of up to $M = 4$ variables, where S denotes the target variable of the PID (it could be, e.g., a feature of a sensory stimulus or the activity of a neural unit), and R_i ($i = 1,2,3$) are the sources of the PID (e.g., activity of a neural unit). In this section, we focus on PID with two source variables, which is the case predominantly considered throughout the paper. (We cover definitions of PID with three source variables in a later dedicated section.) Mutual information carried by a source R_i about the target S quantifies the average reduction in the entropy of the target S upon a single-trial observation of the value of the source R_i and can be expressed as sums and differences of entropy terms:

$$I(S;R_i) = -H(S,R_i) + H(R_i) + H(S). \quad \text{(Equation 10)}$$

Importantly, $I(S;R_i)$ quantifies the full statistical relationship between S and R_i at the single-trial level, capturing the effect of both linear and non-linear interactions. Equation 1 in the main text follows directly from Equation 10 by substituting the entropy terms with their definitions as in Equation 8. Similarly, the mutual information about the target S carried by the joint observation of the two sources (R_1, R_2) is defined as

$$I(S;R_1,R_2) = -H(S,R_1,R_2) + H(R_1,R_2) + H(S). \quad \text{(Equation 11)}$$

As described in the main text, two-sources PID breaks down the joint mutual information that two sources R_1,R_2 carry about the target variable S into the sum of four non-negative components satisfying the constraints in Equations 3, 4, and 5. Once a definition of any of the four PID components is provided, the other three can be obtained as linear combinations between Shannon information quantities and the defined PID component. To facilitate implementation by users, in the following we report the equations to compute

the redundancy $Red(S{:}R_1;R_2)$ and the unique information components $Unq(S{:}R_1\backslash R_2)$ and $Unq(S{:}R_2\backslash R_1)$ as the linear combination of Shannon information quantities and the synergy $Syn(S{:}R_1;R_2)$ (for which we provided a definition in Equation 7).

By subtracting Equation 4 from Equation 3, we obtain

$$I(S;R_1,R_2) - I(S;R_1) = I(S;R_2|R_1) = Unq(S:R_2\backslash R_1) + Syn(S:R_1;R_2), \quad \text{(Equation 12)}$$

where $I(S{:}R_2|R_1)$ is the conditional mutual information about S provided by R_2 given R_1, defined as the joint information carried by R_1 and R_2 minus the information carried individually by R_1.[39] Analogously, by subtracting Equation 5 from Equation 3, we obtain

$$I(S;R_1,R_2) - I(S;R_2) = I(S;R_1|R_2) = Unq(S:R_1\backslash R_2) + Syn(S:R_1;R_2). \quad \text{(Equation 13)}$$

Finally, by subtracting Equations 4 and 5 from Equation 3, we obtain

$$I(S;R_1,R_2) - I(S;R_1) - I(S;R_2) = Syn(S:R_1;R_2) - Red(S:R_1;R_2), \quad \text{(Equation 14)}$$

where the quantity on the left-hand side of Equation 14 is known as the co-information $CoI(S;R_1;R_2)$ of S, R_1, and R_2:

$$CoI(S;R_1;R_2) = I(S;R_1,R_2) - I(S;R_1) - I(S;R_2). \quad \text{(Equation 15)}$$

Linear relationships between the four PID components and Shannon information quantities are depicted in Figure S1. While Equations 12, 13, and 14 do not impose any independent constraint on the four PID components additionally to the ones in Equations 3, 4, and 5, they allow to express $Red(S{:}R_1;R_2)$, $Unq(S{:}R_1\backslash R_2)$, and $Unq(S{:}R_2\backslash R_1)$ as combinations of Shannon information quantities and of $Syn(S{:}R_1;R_2)$, as follows:

$$\begin{aligned} Red(S:R_1;R_2) &= Syn(S:R_1;R_2) - I(S;R_1,R_2) + I(S;R_1) + I(S;R_2) \\ Unq(S:R_1\backslash R_2) &= I(S;R_1|R_2) - Syn(S:R_1;R_2) \\ Unq(S:R_2\backslash R_1) &= I(S;R_2|R_1) - Syn(S:R_1;R_2) \end{aligned} . \quad \text{(Equation 16)}$$

Expressions similar to the ones of Equation 16 can be easily obtained to write any three of the PID components as an explicit function of the fourth one. For example, we could have written $Unq(S{:}R_1\backslash R_2)$, $Unq(S{:}R_2\backslash R_1)$, and $Syn(S{:}R_1;R_2)$ as functions of $Red(S{:}R_1;R_2)$.

BROJA discrete PID

We provide a mathematical definition of the main measure of PID components used in this paper for discrete probability distributions with two source variables, the so-called BROJA measure.[33] The mathematical definitions of the other two discrete PID measures that we use in this paper, I_{min} and I_{MMI}, are provided in Note S1.

We first briefly remind ourselves that for the discrete case, the single-source and joint-source information about the target can be written as a function of the probability distribution $p(s,r_1,r_2)$ and its marginals:

$$I(S;R_i) = \sum_{S,R_i} p(s,r_i)\log_2(p(s,r_i)) - \sum_{R_i} p(r_i)\log_2(p(r_i)) - \sum_{S} p(s)\log_2(p(s)), \quad \text{(Equation 17)}$$

$$I(S;R_1,R_2) = \sum_{S,R_1,R_2} p(s,r_1,r_2)\log_2(p(s,r_1,r_2)) - \sum_{R_1,R_2} p(r_1,r_2)\log_2(p(r_1,r_2)) - \sum_{S} p(s)\log_2(p(s)). \quad \text{(Equation 18)}$$

The BROJA measure[33] is defined via an optimization problem, minimizing the joint mutual information $I(S;R_1,R_2)$ (which depends on the trivariate probability distribution $p(s, r_1, r_2)$) in the probability space Δ_P of distributions $q(s,r_1,r_2)$ having pairwise marginals between each source and the target equal to the original ones, i.e., $q(s,r_1) = p(s,r_1)$ and $q(s,r_2) = p(s,r_2)$. The rationale of this approach is that the synergy can be conceptualized as the information about the target in the joint space of the two sources that cannot be possibly recovered by observing one source at a time (thus from the marginal probabilities), thus it can be defined operationally as the difference between the original joint information and the minimal joint information about the target that can be found in the space Δ_P of distributions that preserve the marginals (which is the union information, Equation 6):

$$Syn(S:R_1;R_2) = I(S;R_1,R_2) - \min_{q \in \Delta_P} I_q(S;R_1,R_2). \quad \text{(Equation 19)}$$

Redundancy and unique information components can then be computed from the synergy and the Shannon information values using the linear relationships provided above (see Equation 16). Among the advantages of the BROJA PID, we note that this definition ensures that all PID terms satisfy the additivity property (i.e., when both the sources and the target are composed of independent subsystems, the PID components computed for the full system equal the sum of the corresponding components computed separately for each subsystem). However, while I_{MMI} and I_{min} have been defined for an arbitrary number of source variables,[22,97] the BROJA PID is currently defined only for the case of two source variables.

Gaussian PID measures

For the Gaussian case, the single-source and joint information about the target can be written in terms of the covariance matrices as follows[39]:

$$I(S;R_i) = \frac{1}{2}\log_2\left(\frac{\det(\Sigma(S))\det(\Sigma(R_i))}{\det(\Sigma(S,R_i))}\right), \qquad \text{(Equation 20)}$$

$$I(S;R_1,R_2) = \frac{1}{2}\log_2\left(\frac{\det(\Sigma(S))\det(\Sigma(R_1,R_2))}{\det(\Sigma(S,R_1,R_2))}\right), \qquad \text{(Equation 21)}$$

where $\Sigma()$ denotes the covariance matrix of the variables listed as arguments (e.g., $\Sigma(S, R_1, R_2)$ is the covariance matrix of the joint multivariate Gaussian probability of observing in the same trial the combination of the target s and the two sources r_1,r_2).

In principle, any PID that has redundancy $Red(S{:}R_1,R_2)$ and unique information components $Unq(S{:}R_1\backslash R_2)$ and $Unq(S{:}R_2\backslash R_1)$ depending only on the marginal distributions between the sources and the target (the so-called pairwise marginal property, see Note S2) reduces to I_{MMI} when computed from jointly Gaussian distributions p_G and the target S is one-dimensional.[34] For the Gaussian MMI, closed-form solutions for PID exist, since the mutual information terms appearing in Equation 3 can be expressed in terms of covariance matrices as in Equation 20. To our knowledge, no general, closed-form expression exists for Gaussian PIDs with a multi-dimensional target, and such systems can yield results that deviate from I_{MMI}.

One PID definition developed explicitly for Gaussian variables is the gPID measure,[30] which defines the union information via constrained optimization as

$$Union(S:R_1;R_2) = \min_{q\in\Delta_{P_G}} I_q(S;R_1,R_2), \qquad \text{(Equation 22)}$$

where Δ_{P_G} is the set of all jointly Gaussian probability distributions $q(s,r_1,r_2)$ that have the same pairwise marginals $q(s,r_1) = p(s,r_1)$ and $q(s,r_2) = p(s,r_2)$ as the original distribution $p(s, r_1, r_2)$, and $I_q(S;R_1,R_2)$ is the joint information computed for distribution $q(s;r_1,r_2)$.

This definition corresponds to the BROJA PID one but restricts the domain of the optimization problem to the space of jointly Gaussian distributions. Given a Gaussian probability distribution with a covariance matrix $\Sigma(R_1,R_2,S)$, the optimization problem in Equation 22 can be expressed in terms of cross- and auto-covariance matrices derived from $\Sigma(R_1, R_2, S)$[30]:

$$Union(S:R_1;R_2) = \frac{\min_{\Sigma(R_1,R_2|S)} 1}{2\ln 2}\ln\left[\det\left(I + \Sigma(S)^{-1}\Sigma(R_1R_2,S)^T\Sigma(R_1,R_2|S)\Sigma(R_1R_2,S)\right)\right], \qquad \text{(Equation 23)}$$

where $\Sigma(R_1R_2, S)$ denotes the cross-covariance matrix between the joint neuronal responses (R_1, R_2) and the target S, $\Sigma(R_1, R_2|S)$ the conditional cross-covariance between R_1 and R_2 given S, and $\Sigma(S)$ the auto-covariance matrix of S.

Key properties of PID components for two source variables are reported in Note S2.

Details of implementation of the bias-correction procedures

QE

The QE procedure[60] assumes we are in the asymptotic sampling regime $N \times p(s,r_1,r_2) \gg 1$ for all s,r_1,r_2, so that the bias of the entropies, information, and PID terms can be accurately approximated as a second-order expansion in $1/N$. The estimation of the coefficients of this expansion is done by recomputing the information from fractions ($N/2$ and $N/4$) of the data available and then fitting (using a least-squares-error procedure) the plugin (uncorrected) information values obtained with fractions of data to the preceding quadratic function of $1/N$. From this second-order equation, the value of information estimated for $N = \infty$ is taken as the unbiased information estimate. In our numerical implementation of QE, we allowed for a user-selected number K of data partitions into halves and quarters. We performed a separate extrapolation for each partition and then averaged the resulting estimates of the unbiased information value in each extrapolation to obtain our final estimate of the unbiased information. To ensure that the bias-corrected PID terms respect exactly the linear constraints of PID theory (Equations 3, 4, and 5), we computed the bias correction for the synergy, the joint, and the single-source information and then computed the bias-corrected unique and redundant information from the linear constraints (Equation 16). We used $K = 20$ partitions in the simulations in the main text and the supplemental information as well as in the analyses of real data. We applied QE only to the discrete PID because the bias of the Gaussian PID was clearly not polynomial (see Note S6).

Shuffle-subtraction bias correction

This procedure was applied in the same way to discrete and Gaussian PID. To estimate the bias, we computed PID after randomly permuting the target values across trials. In this way, all information about the target is lost and all PID terms should be zero but for the finite sampling effect. Thus, the resulting PID terms can be taken as an estimation of their bias, which we subtracted from their plugin estimates. In our implementation, we allowed the user to perform a number V of random permutations, and we then took as a measure of the bias (to be subtracted from the plugin estimate to obtain the unbiased estimates) the average of the PID term computed over all random realizations of the permutation. To ensure that the bias-corrected PID terms respect exactly the linear constraints of PID theory (Equations 3, 4, and 5), we computed the bias correction for the synergy, the joint, and the single-source information and computed the bias-corrected unique and redundant information from the linear constraints (Equation 16). We used $V = 20$ in the simulations in the main text and the supplemental information as well as in the analyses of real data.

Resampling bias correction

The resampling method for bias correction is grounded on the assumption that information measures from similar distributions suffer from similar biases. From the available dataset with N samples, we first compute an estimated probability matrix (for the discrete case) or an estimated covariance matrix (for the Gaussian case). We then generate N samples from this estimated distribution, and we compute the bias as the difference between the information obtained from the N samples generated by the estimate distribution and the information obtained by inserting the estimated distribution into the information or PID equations. This process is repeated W times, so the bias is estimated as the average across many surrogate datasets. Finally, we correct the original information values by subtracting the

estimated bias. To ensure that the bias-corrected PID terms respect exactly the linear constraints of PID theory (Equations 3, 4, and 5), we computed the bias correction for the synergy, joint, and single-source information and then computed the bias-corrected unique and redundant information from the linear constraints (Equation 16). We used $W = 20$ resampling iterations in the simulations in the main text and the supplemental information as well as in the analyses of real data. Given that, in the discrete scenario, the resampling bias correction exhibits the same sign of residual bias as QE but consistently poorer performance (Figures S5–S7), we did not include this correction in the discrete analyses presented in the main text.

Merged bias correction

Because the shuffle subtraction and the QE (for discrete) or resampling (for Gaussian) tend to have residual errors of opposite sign (see Figures 4, 5, and S3–S10), we introduced a merged bias correction that estimates the bias as the weighted average of the two above-described procedures:

$$Bias_{\text{merged}} = w_{\text{QE}} \cdot Bias_{\text{QE}} + (1 - w_{\text{QE}}) \cdot Bias_{\text{shuff}}, \quad \text{(Equation 24)}$$

where $Bias_{\text{QE}}$ and $Bias_{\text{shuff}}$ are the bias estimates from the QE procedure (for the discrete case) or the resampling correction (for Gaussian) and shuffle-subtraction procedures, respectively, $w_{\text{QE}} \in [0,1]$ is the weight assigned to the QE (for discrete PID) or resampling (for Gaussian) correction, and $1 - w_{\text{QE}}$ is the weight assigned to the shuffle subtraction. For the discrete two-source PID, w_{QE} was set using a heuristically optimized, information-level-dependent weighting. Since shuffle subtraction performs considerably better than QE at low information levels, and the opposite holds at higher information levels, for each simulated data point from all four discrete simulation scenarios shown in Figure S5, we computed

$$w_{\text{QE}} = \frac{R_{\text{shuff}}}{R_{\text{QE}} + R_{\text{shuff}}}, \quad \text{(Equation 25)}$$

where R_{QE} and R_{shuff} denote the absolute value of the residual biases after QE and shuffle correction, respectively. By construction, w_{QE} assigns a higher weight to the correction that has the smaller residual error.

Since Equation 25 depends on the residual biases R_{QE} and R_{shuff}, which require knowledge of the ground-truth information values to be computed and are thus unavailable in practice, we modeled the dependence of w_{QE} on the normalized information level (expressed as the joint information value normalized to the value of the entropy of the target variable, thus a number between 0 and 1) by fitting a sigmoid function of the normalized information level to the values of w_{QE} and their corresponding information levels pooled across all four simulation scenarios. These fits were performed separately for each combination of N and R, resulting in a set of sigmoid functions that return the weight w_{QE} to be applied given the observed information level and the experimental parameters N and R. We verified (Figure S12) that this optimized weighted merged procedure improved slightly a simpler version with equal weighting of the shuffle and QE.

For the Gaussian case, we always used an equally weighted combination of the shuffle and resampling bias-correction methods ($w_{\text{QE}} = 0.5$), because the residual biases of the shuffle-subtraction and resampling corrections show no differences in their dependence on the information level; thus, heuristically optimizing the weight as function of the information level would not have produced a performance increase.

For the three-source PID analyses (Figures 7 and 8), we used an equal weighting between the shuffle and the QE correction because in the range of simulations we explored, we did not have major shifts in information levels.

For the Gaussian case, since the residual biases of the shuffle-subtraction and resampling corrections show no differences in their dependence on the information level, we used an equally weighted combination of the shuffle and resampling bias-correction methods.

To ensure that the bias-corrected PID terms respect exactly the linear constraints of PID theory (Equations 3, 4, and 5), we computed the bias correction for the synergy, joint, and single-source information and computed the bias-corrected unique and redundant information from the linear constraints (Equation 16). We used $V = 20$ and $K = 20$ in the simulations in the main text and supplemental information as well as in the analyses of real data.

To provide users with an estimate of the magnitude of the expected residual error after applying the merged correction while in the sufficient-sampling regime, we fitted (using the data from simulations in Figures S3A, S4, and S5) the residual bias after applying the merged correction to a second-order polynomial of the parameter ratio that determines the leading-order scaling of the joint information and the synergy bias, namely the ratio between the total number of parameters defining the joint probability distribution (R^3 in the discrete case and $3d$ in the Gaussian case) and the number of samples N (see Note S6). The resulting second-order equations (plotted in Figure S15) allow users to easily obtain an estimation of the magnitude of the residual bias of both synergy and redundancy for the discrete and Gaussian PID as a function of N, R, and d. This helps in designing the experiment and analysis that would allow estimation results to comply with a desired tolerance level.

Venkatesh et al. procedure for Gaussian PID bias correction

For the Gaussian PID only, we implemented the bias-correction procedures developed by Venkatesh and colleagues.[30] Applying this procedure to the discrete PID would have been not possible because it assumes an analytical knowledge of the bias, which is only available for the Gaussian case.

The Venkatesh bias-correction procedure[30] focuses first on the bias of the union information. The Venkatesh procedure corrects for the bias by first computing the bias-corrected joint information (see Equations 17, 106, and 107 of Venkatesh et al.[30]) with the Cai et al.[80] equations of the entropy bias (Equation S17), then making the assumptions that the union information has the same proportional bias as the joint information, thus rescaling the bias of the union information accordingly (Equation 17 of Venkatesh et al.[30]). Note that, following Venkatesh et al.,[30] if a Shannon information quantity had a negative value after the bias-correction procedure, it was set to zero. Then, to make sure all PID quantities after bias correction are non-negative and satisfy the PID linear constraints, the Venkatesh procedure applies a double post hoc rectification of the bias-corrected value of the union

information as detailed in Equations 106 and 107 of Venkatesh et al.[30] The resulting double-rectified union is the value taken by Venkatesh et al. as the bias-corrected union information.

From this, the Venkatesh procedure computes the four bias-corrected PID terms using the bias-corrected union information and the bias-corrected joint and individual information using the four linear PID constraints of Equations 3, 4, 5, and 7.

Our results show that, by assuming that the bias of the union information is proportional to the bias of the joint information, this approach can severely misattribute bias across PID components. In the Gaussian case, analytical considerations (see Note S6) indicate that the union information bias depends only on the dimensionality of the covariance matrices d and on the number of trials N and not on the magnitude of the union information itself (as implicitly assumed in Equation 17 of Venkatesh et al.[30]). Furthermore, the ratio between the biases of union and joint information is not fixed: it varies across sampling regimes, with joint information exhibiting disproportionately larger bias at low trial counts N or higher dimensionality of sources and target d (see Note S6). Because the Venkatesh procedure relies on these incorrect proportional-bias assumptions, it misassigns the bias among PID components, especially when all PID terms are non-zero and the true union information does not match any of the Shannon information quantities (so that the rectification steps in Equations 106 and 107 of Venkatesh et al.[30] do not correct the error). Simulations corroborate these analytical predictions (Figures 4, 5, S3, and S8–S11): the Venkatesh correction typically underestimates the bias of synergy and redundancy.

Details of the binning procedures used to compute the discrete PID

For all simulated data, we estimated the PID components based on the following binning approach. First, in each simulation, we generated a large number (n = 2,048) of trials per stimulus for each set of model parameters. We then discretized the probability distribution of spike counts for each neuron in the chosen number of bins. We computed the bin edges that made the partition of trials as equally populated as possible, following the procedure of Lorenz et al.[64] These bin edges were then used for all simulations computing PID as a function of the number of trials per stimulus. We chose to fix the bin edges for each simulation to ensure that differences across trial numbers in the estimated values of the PID components were due only to trial numerosity and not to other reasons. This was important to study the properties of the information estimates as a function of the number of available trials. For real data, the binning procedure is specified in the supplemental information section describing their information analysis. The number of bins used to discretize single neural unit activity in each figure is reported in Table S1.

Details of simulations used to test the bias properties

In this section, we report details of methods to generate the simulated tests presented in the paper. Parameters used for each figure are reported in Tables S1 and S2. The parameters used in individual figure panels were chosen with the criteria that these parameters were representative of wider trends, were within the range in our experience found in real-data analyses of neuroscience experiments, and illustrate well the point of interest. In all the simulations, we generated random values of a target variable (conceptualized as a sensory stimulus) and of two sources (conceptualized as the activity of different neural units). In all simulations, we collected one joint sample of target and sources values per trial; thus, the number of samples N used to compute the probabilities from simulated data equals the number of simulated trials.

Details of discrete simulations

To test the bias of the discrete PID algorithms, we simulated the activity of two neural units (without loss of generality, the spiking activity of two neurons) responding to a set of discrete stimuli.

In Figure 1 (zero-information case), we considered a simple scenario in which, on each simulated trial, the stimulus S and the spike count of each individual neuron R_1 and R_2 were drawn independently from a uniform probability distribution with four possible outcomes. Thus, the ground-truth value of joint information about the stimulus carried by the activity of two neurons (and, thus, synergy and redundancy) is zero.

To characterize bias scaling and the performance of bias-correction procedures in scenarios with non-zero joint information, we simulated processes with statistics reasonably close to neural firing while allowing us to control the strength of correlations between neurons as a function of the stimulus. This enabled systematic manipulation of the presence and magnitude of synergy across different stimulated scenarios.

We implemented a simple simulation based on Poisson responses (which are a reasonable approximation to neural firing[71]) that are either independent or shared across neurons. Specifically, the spike count r_1 and r_2 of each of the two simulated neurons for each of the stimulus values were the sum of two Poisson processes: one Poisson process that was independently drawn for each neuron capturing neuron-specific ("private") variability of responses, and a second Poisson process that was shared between the two neurons, giving rise to noise correlations.

In all discrete PID simulations, apart from those in Figures 2A, 3A–B, 5A, and S3, we used a set of $S = 4$ simulated stimuli. This set of four simulated stimuli was made of two binary stimulus features s_1 and s_2, which varied across trials independently from each other. (This made it a 2-bit stimulus set, and the two independent binary stimulus features are coded as $\lfloor s/2 \rfloor$ and $s \bmod 2$ when spanning the stimulus id from 0 to 3 in the equations below). The equations for the mean rates (that is, mean spike counts) of each Poisson process follow for each simulation the following form:

$$\begin{aligned}
&rate_{shared} = \gamma, \\
&rate_{individual-1}(s) = B + \alpha\beta s_1 + \alpha(1-\beta)s_2, \\
&rate_{individual-2}(s) = B + \alpha(1-\beta)s_1 + \alpha\beta s_2, \\
&s_1 = \left\lfloor \frac{s}{2} \right\rfloor, s_2 = (s \bmod 2), s \in \{0,1,2,3\}.
\end{aligned} \quad \text{(Equation 26)}$$

In the above mean rate equation used for our simulations of the spike counts of pairs of neurons in response to the stimulus, we have the following free parameters, which were

varied across simulations. (1) A parameter α regulates the strength of stimulus tuning of the spike counts of the individual neurons or, in other words, the separation in response strength between least and most effective stimuli (higher values of α leading to higher values of individual neuron information and, thus, higher values of joint information); (2) a baseline parameter B expressing the overall baseline level of activity common to all stimuli (higher values of B in general decrease information of individual neurons and, thus, the joint information because they increase the standard deviation of responses at fixed stimulus and thus make the rate separation α between most and least effective stimuli smaller in standard deviation units); (3) a parameter β, which increases the amount of redundancy because it regulates the dissimilarity of tuning of the spike count of each individual neurons to the two stimulus features (β close to zero or one means independent tuning, i.e., each neuron encodes a different feature; higher values of β close to $\beta = 0.5$ indicate that both neurons encode the features similarly and redundantly, i.e., the two neurons encode the same linear combination of features); (4) a parameter γ regulating the strength of the shared process and, thus, the strength of correlated firing (increasing γ increases synergy because it makes the two neurons more strongly correlated—because the individual process changes its rate with different stimuli, having a shared process $\gamma > 0$ also affects the amount of synergistic information about the stimulus that can be gained by only measuring the joint responses of the two neurons because the fraction of shared spikes will change with the stimulus, a stimulus-dependent correlation case that is known to create synergy[20]).

These formulas allow us to construct analytically the ground-truth probability distribution for each scenario and information level. By using these probability distributions in the PID computation, we can use the resulting values to compute the limited-sample bias that we obtain when using PID on the probability distribution estimated from N samples.

To test the generality of our methods, we developed, by tuning the simulation parameters, several scenarios of interest (see Table S2 for full details) with different values of the individual PID component. A first scenario (‘‘bit-of-all’’) had all PID components with non-zero values (as it happened in all the four neuroscientific datasets we analyzed with two-source PID). A second scenario (‘‘uncorrelated’’) had negligible synergy and negligible redundancy (because the two neurons were uncorrelated, and each neuron encoded a different independently varying stimulus feature). A third scenario (‘‘high redundancy’’) had both neurons encoding the stimuli very similarly and thus had high redundancy but negligible synergy. A fourth scenario (‘‘high synergy’’), with strong and information-rich correlations between the neurons, had high synergy and negligible redundancy. Information about the scenario used for each discrete PID simulation is indicated in the respective figure captions.

For the simulations of different numbers of bins (Figures 2A, 3A–B, 5A, and S3), we needed to change the number of stimuli S together with the number of bins R. The coding of the variables indicating the two stimulus features s_1 and s_2 were modified as follows to make it easier to simulate a stimulus set with numerosity ranging from 2 to 9:

$$s_1 = \left\lfloor \frac{s}{3} \right\rfloor, s_2 = (s \bmod 3), s \in \{0,..,S-1\}. \quad \text{(Equation 27)}$$

Table S2 provides a summary of the parameters for all discrete simulation scenarios used in each figure.

Details of Gaussian simulations

To test the bias of the Gaussian PID algorithms, we simulated jointly Gaussian systems describing the activity of two neural units (without loss of generality, aggregate measures of neural activity recorded from two brain regions) responding to a Gaussian stimulus.

For the results shown in Figure 1C–D, we simulated jointly Gaussian variables S, R_1, and R_2, each of dimension $d = 5$, using a covariance matrix equal to the identity matrix of size $3d$.

To simulate the different cases with non-zero components, we simulated jointly Gaussian variables S, R_1, and R_2, each of dimension d, using the ‘‘bit-of-all,’’ ‘‘both-unique,’’ ‘‘high-synergy,’’ and ‘‘zero-synergy’’ scenarios defined and used to test the gPID method in Venkatesh et al.[30] (see section C.1, p.22 of their supplemental information). The both-unique case is designed to have no redundancy or synergy (Figure S11). The high-synergy case includes a significant amount of synergy (Figure S11). The zero-synergy case has no synergy component (Figure S11). The bit-of-all case combines features of the high-synergy and zero-synergy cases, ensuring that all PID components are non-zero. In all cases, the desired examples are obtained by setting appropriately the structure of the covariance matrix $\Sigma(S,R_1,R_2)$ to achieve the desired effects, as explained in detail in Venkatesh et al.[30] For all simulations, we used exactly the parameters reported in Venkatesh et al.[30] for the corresponding case (results shown in Figure S11). Information about the scenario used for each Gaussian PID simulation is indicated in the respective figure captions. For the bit-of-all simulation, to study the sampling bias across different information levels, we scaled the diagonal of the covariance matrix by a factor α, thereby increasing the variance of each variable’s dimensions, effectively adding noise and reducing the mutual information between target and sources. $\alpha = 1$ corresponds to choosing parameter values exactly equal to those reported in Venkatesh et al.[30] $\alpha > 1$ reduces the total mutual information. The α values used for each simulated scenario are reported in Table S2. The results of gPID applied to bit-of-all with various levels of α are reported in Figures S9 and S10.

In each simulated scenario, we generate N samples of multivariate Gaussian data to estimate the sample covariance matrix $\tilde{\Sigma}(S, R_1, R_2)$. We then apply to the simulated data the gPID estimator[30] which is a function of only the covariance matrix computed from the data (Equation 23). The bias of the PID components is computed as the difference between the average of the value of the component across all realizations of the simulations with N minus the ground-truth value of the component. The latter is computed numerically, giving as input to the gPID equations the true covariance matrix used to generate the data.

For simulations in Figure 3C, we computed the Gaussian PID of the conditionally independent joint information,

$I_{ind}(S;R_1,R_2)$, built from $p_{ind}(s,r_1,r_2)$ (see Note S6 for further details). The distribution $p_{ind}(s, r_1, r_2)$ has a covariance matrix with the following form:

$$\Sigma_{ind}(S,R_1,R_2) = \begin{bmatrix} \Sigma(S) & \Sigma(S,R_1) & \Sigma(S,R_2) \\ \Sigma(R_1,S) & \Sigma(R_1) & \Sigma(R_1,S)\Sigma(S)^{-1}\Sigma(R_2,S)^{\top} \\ \Sigma(R_2,S) & \Sigma(R_2,S)\Sigma(S)^{-1}\Sigma(R_1,S)^{\top} & \Sigma(R_2) \end{bmatrix}, \quad \text{(Equation 28)}$$

where in deriving the above we used the following identities for jointly Gaussian variables:

$$\begin{aligned} \Sigma(R_1,R_2) &= \Sigma(R_1,S)\Sigma(S)^{-1}\Sigma(R_2,S)^{\top} + \Sigma(R_1,R_2|S) \\ \Sigma_{ind}(R_1,R_2|S) &= 0 \end{aligned}, \quad \text{(Equation 29)}$$

which are symmetric under permutation of R_1 and R_2.

Further results of simulations and bias corrections are provided in Note S4.

Details of two-source PID analysis of real neural data

Mouse auditory cortex data recorded during a sound discrimination task

We reanalyzed a previously published dataset[36] in which the activity of several tens to a few hundreds of neurons was recorded simultaneously using *in vivo* two-photon calcium imaging from A1 L2/3 neurons in head-fixed mice during a pure-tone discrimination task (Figure 6A). After a pre-stimulus interval of 1 s, head-fixed mice were exposed to either a low-frequency (7 or 9.9 kHz) or high-frequency (14 or 19.8 kHz) tone for 1 s. Mice were trained to report their perception of the tone through their behavioral choice by licking a waterspout in the post-stimulus interval (0.5–3 s from stimulus onset) after hearing a low-frequency tone (target tones) and holding still after hearing high-frequency tones (non-target tones). Two-photon calcium imaging was used to record calcium fluorescence signals from individual A1 L2/3 neurons with an imaging frame rate of 30 Hz, from which estimated spike rates were obtained with deconvolution.[36] We analyzed neurons recorded from n = 12 fields of view (FOVs) from n = 12 animals. For consistency with the original publication,[36] we only considered individual neurons that carried significant intersection information,[66] that is, information about the stimuli used to inform the behavioral discrimination, according to the statistical tests described in Francis et al.[36] This led to selecting for PID analysis n = 375 individual neurons (out of the 2,792 recorded neurons), leading to n = 6,209 pairs of simultaneously recorded neurons used for the PID analysis.

For the PID analysis, we identified for each neuron the imaging time frame within the trial of maximal intersection information[66] as described in Francis et al.[36] We then considered for each neuron a time window of n = 10 imaging frames (333 ms) around the peak intersection information timeframe (we call this the peak time window for the neuron). The activity of each neuron within the peak time window was discretized into R = 3 bins corresponding to 0, 1, or >1 spikes. The stimulus set used for this analysis was binary (S = 2), dividing the presented sound tones into the low-frequency (s = 0) and high-frequency (s = 1) categories. For consistency with the original publication,[36] which analyzed joint stimulus information carried by pairs of neurons without breaking it up with the PID, we estimated the total stimulus information that was jointly carried by pairs of neurons following a time-lagged approach. Namely, we computed the time-lagged stimulus information carried jointly by the activity of each pair of neurons using Equation 2 and using as responses r_1 or r_2, the discretized responses of each neuron measured as detailed above in their respective peak time windows. The analysis of the full dataset is presented in Figure 6A, while a comparison between the half-data and full-data analyses is shown in Figure S16A.

Mouse hippocampus data recorded during virtual spatial navigation

We reanalyzed a previously published dataset[63] in which the activity of several tens to a few hundreds of neurons was recorded simultaneously using *in vivo* two-photon calcium imaging from CA1 neurons in head-fixed mice during virtual-reality navigation of a linear track (Figure 6B). Calcium fluorescence signals were obtained using the jRCaMP1a calcium indicator and an imaging frame rate of 3 frames per second. Full details of the experimental procedures and details of the ethical approval are reported in full in the original publication.[63]

We analyzed neurons recorded from n = 11 FOVs from n = 7 animals. Because hippocampal neurons encode position in space, we computed the mutual information between the spatial position S in which the animal was located in the linear track and the joint activity of two simultaneously recorded neurons r_1 and r_2. For consistency with the previous study reporting the original data,[63] the spatial position s along the linear track was computed by binning the space along the track into S = 12 equipopulated bins. For consistency with the previous study,[63] to quantify the neural activity r_i of each neuron, we took the calcium traces and binned them into R = 2 bins (only raw calcium traces and not deconvolved signals were available from Curreli et al.[63]). We computed information between neural activity and spatial position across all available time frames. For the PID analysis, we used all the n = 870 individual neurons present in the dataset, leading to n = 36,158 pairs of simultaneously recorded neurons used for the PID analysis. The analysis of the full dataset is presented in Figure 6B, while a comparison between the half-data and full-data analyses is shown in Figure S16B.

Monkey auditory cortex data recorded during passive listening to natural sounds

We reanalyzed a previously published dataset,[11] which consisted of simultaneous recordings (n = 4 different recording sessions) with multiple electrodes from the auditory cortex (A1) of a monkey (*Macaca mulatta*) passively listening to natural sounds with four different levels of natural noise. Each natural sound snippet was 20 s long and was repeated for 80–120 total trials

across sessions (mean 108 trials). We computed the joint information (and its PID) carried by neural activity R_1,R_2 (defined as spike counts discretized in R = 2 levels) concerning which of S = 399 different sound segments (each 50 ms long) were presented. The analysis of the full dataset is presented in Figure 6C, while a comparison between the half-data and full-data analyses is shown in Figure S16C.

Human resting-state fMRI data

The dataset of functional and structural neuroimaging data used in this work came from the Human Connectome Project (HCP, https://www.humanconnectome.org/study/hcp-young-adult/document/q3-data-release), Release Q3.[85,86] Per HCP protocol, all subjects gave written informed consent to the HCP consortium. These data contained fMRI and diffusion-weighted imaging (DWI) acquisitions from 100 unrelated subjects of the HCP 900 data release.[85,86] All HCP scanning protocols were approved by the local Institutional Review Board at Washington University in St. Louis. Detailed information about the acquisition and imaging is provided in the dedicated HCP publications. In brief, anatomical (T1-weighted) images were acquired in axial orientation, with FOV = 224 $\times$ 224 mm, voxel size 0.7 mm^3 (isotropic), TR 2,400 ms, TE 2.14 ms, and flip angle 8°. fMRI data (1,200 volumes) were acquired with EPI sequence, 2 mm isotropic voxel size, TR 720 ms, TE 33.1 ms, flip angle 52°, and 72 slices.

We used the minimally preprocessed fMRI data from the HCP, which includes bias field correction, functional realignment, motion correction, and spatial normalization to Montreal Neurological Institute (MNI-152) standard space with 2-mm isotropic resampling resolution. We also removed the first 10 volumes to allow magnetization to reach steady state. Additional denoising steps were performed using the CONN toolbox (http://www.nitrc.org/projects/conn), version 17f.[87] To reduce noise due to cardiac and motion artifacts, we applied the anatomical CompCor method of denoising the functional data. The anatomical CompCor method (also implemented within the CONN toolbox) involves regressing out of the functional data of each individual the first five principal components corresponding to white matter signal and the first five components corresponding to cerebrospinal fluid signal, as well as six subject-specific realignment parameters (three translations and three rotations) and their first-order temporal derivatives, in addition to nuisance regressors identified by the artifact detection software ART.[87,88] The subject-specific denoised BOLD signal time series were linearly detrended and band-pass filtered between 0.008 and 0.09 Hz to eliminate both low-frequency drift effects and high-frequency noise. Human brains were parcellated into 1,000 ROIs covering the entire cortex. The 1,000 cortical ROIs were obtained from the scale-1000 version of the recent local-global functional parcellation of Schaefer et al.[89] The time courses of denoised BOLD signals were averaged between all voxels belonging to a given atlas-derived ROI using the CONN toolbox. The analysis of the full dataset is presented in Figure 6D.

Synergy and redundancy bias in three-source PID

Definition of three-way redundancy and three-way synergy with the MMI PID

We study the bias of the MMI PID of the joint information with three source variables R_1, R_2, R_3 and one target variable S for both the discrete and the Gaussian cases.[22,34,98] For three source variables, PID breaks down the joint Shannon information $I(S;R_1,R_2,R_3)$ into 18 components corresponding to a combination of redundant, unique, and synergistic information.[22] Here, we focus on two terms that have a straightforward interpretation and have been used for the analysis of real and artificial neural networks.[90,98] The first is the three-way redundancy (Red_3), which quantifies the information that each of the sources R_1, R_2, and R_3 encode about S. The second is the three-way synergy (Syn_3), which quantifies the information about S that is available only from the joint observation of (R_1, R_2, R_3) and not from any individual source or pair of sources. Using MMI, the three-way redundancy is defined as[97,98]

$$Red_3 = \min[I(S;R_1), I(S;R_2), I(S;R_3)], \quad \text{(Equation 30)}$$

while three-way synergy has the following expression[90,98]:

$$Syn_3 = I(S;R_1,R_2,R_3) - \max[I(S;R_1,R_2), I(S;R_1,R_3), I(S;R_2,R_3)]. \quad \text{(Equation 31)}$$

Thus, the bias of three-way synergy Syn_3 and three-way redundancy Red_3 with the MMI PID can be estimated from the bias of Shannon information quantities. For analytical considerations on the bias of the three-way synergy and three-way redundancy, see Note S8.

Details of the simulations used to test the three-source PID

To evaluate numerically the bias and test bias-correction procedures for the three-source MMI PID (Figure 7), we performed numerical simulations.

To test the discrete PID, we simulated a discrete process that was fully synergistic. Source variables X_1, X_2, and X_3 were sampled from a uniform distribution over R possible values, and the target variable was defined as the sum of all source variables, taken modulo R:

$$Y = (X_1 + X_2 + X_3) \bmod R. \quad \text{(Equation 32)}$$

This is an extension of the 3-bit parity system[109,110] using modulo R instead of 2 to have a similar structure in the number of bins as in the two-source simulations. In this setting, the value of each individual source, or the combination of any pair of sources, does not provide any information about the target variable, while the combination of the three sources fully determines Y. Therefore, by construction, only the three-way synergy Syn_3 PID component is non-zero.

For the Gaussian simulations, we sampled from a multivariate normal distribution such that each source X_i and the target Y share a common latent signal S plus independent noise ($X_i = S + \varepsilon_i^x$, $Y = S + \varepsilon^y$), with $\varepsilon \sim \mathcal{N}(0,1)$, yielding a block-diagonal covariance matrix where diagonal elements represent total variance ($\sigma_S^2 + \sigma_\varepsilon^2$) and off-diagonal elements represent the shared signal variance (σ_S^2). This system has a ground-truth non-zero redundancy component only.

Three-source PID analysis of real MEG data

We analyzed a previously published dataset[77] in which MEG signals were recorded from healthy human participants (n =

CellPress
OPEN ACCESS

30) performing a visual evidence accumulation task. In each trial, participants observed a sequence of 12 visual samples (checkered disks), whose orientation from the midline was drawn from a Gaussian distribution with a given generative mean value. Participants performed a decision-making task consisting of estimating the generative mean of these samples. Here, we focused on the encoding by MEG activity of the orientation of this sample, which is thus focused on the sensory encoding rather than on the decision-making aspects of the experiment. MEG data were acquired with a 275-channel CTF system at a sampling rate of 1,200 Hz. The preprocessed MEG data were source-reconstructed onto the 180 cortical areas of the Glasser atlas[111] using individual head models generated with the aid of T1-weighted structural MRI scans acquired for each participant. To reduce the dimensionality within each area, we focused on the first principal component of MEG activity computed across all vertices in each area.

The Gaussian PID assumes that the target-source distributions are approximately Gaussian. The target variable (visual sample orientation) was drawn from a Gaussian distribution by experimental design.[77] To verify that this assumption also held for the sources conditioned on the target, we divided the target range into eight equal-width bins spanning the minimum to maximum angle presented across all trials. This gave a bin width of $12^\circ \pm 0.25^\circ$ (mean ± SD across participants). The number of trials available to compute this angle-specific distribution was 90 ± 60 (mean ± SD across participants). We estimated the angle-specific empirical distribution of MEG responses within each bin using kernel density estimation (KDE), then assessed normality with Shapiro-Wilk tests across all 240 angle-specific distributions obtained across all subjects from V1. Only 6.25% of distributions were significantly different from a Gaussian under a false discovery rate (FDR) of 5% (Benjamini-Hochberg FDR correction, $q < 0.05$), meaning deviations from Gaussianity were only slightly more frequent than expected by chance.

For the PID analysis, we quantified the information provided by three neural sources about the orientation of the first visual sample. The three-source PID analysis was applied to both the continuous multivariate data and the discretized data. For the discrete analysis, the three sources were defined as the first principal component of activity from cortical areas V1, V2, and V3. The time series of each source and the stimulus orientation were then discretized into $R = 3$ equipopulated bins. For the multivariate Gaussian analysis, we defined three large-scale sources by pooling the first principal component of activity across multiple cortical areas: source 1 comprised all 26 areas within the visual cortex, source 2 included all 47 areas from the prefrontal cortex, and source 3 consisted of all 25 areas from the somatosensory and motor cortices. For both analyses, we computed PID components using a naive plugin estimator and compared the results against appropriate bias-correction schemes to quantify the effects of finite sampling bias (see Figure 8). We analyzed either pre-stimulus data (MEG responses collected in the 150 ms prior to the onset of stimulus presentation) or post-stimulus data (MEG data recorded in the 150 ms following the onset of stimulus presentation).

RESOURCE AVAILABILITY

Lead contact

Requests for further information and resources should be directed to and will be fulfilled by the lead contact, Stefano Panzeri (s.panzeri@uke.de).

Materials availability

This study did not generate new materials.

Data and code availability

Code for simulating and analyzing data is made available at our repository at https://doi.org/10.5281/zenodo.20085438.[112] The code uses the MINT toolbox.[64] The real neural data used here in Figures 6 and S16 were published previously. Dataset 1 was released with the original publication.[36] Dataset 2 was first published in Curreli et al.[63] and is available for download from Lorenz et al.[64] Dataset 3 was published in Kayser et al.[11] and is made available in our repository at https://doi.org/10.5281/zenodo.20085438.[112] Dataset 4 is publicly available from the original publication.[86] Dataset 5 is publicly available from the original publication.[77]

ACKNOWLEDGMENTS

We are grateful to Hame Park and Tobias H. Donner for discussions and support on the analysis of their MEG data analyzed in the present study. This work was supported by the Simons Foundation Autism Research Initiative grant 982347 (to S.P.), the German Federal Ministry of Education and Research BMBF 01GQ2404 (to S.P.), the European Union's European Research Council NEUROPATTERNS grant 647725 (to T.F.) and cICMs ERC-2022-AdG-101097402 (to A.K.E.), the European Union's Horizon Research and Innovation Programme grants 101206609 (Marie Sklodowska-Curie Global Fellowship AstroError to MC) and 101152984 (EU Marie Sklodowska-Curie Action to S.B.M.), a Wellcome Early Career Award 226924/Z/23/Z (to A.I.L.), and St. John's College Cambridge (A.I.L.). The Funders had no role in study design, data collection and analysis, decision to publish, or preparation of the manuscript. Views and opinions expressed in this article are those of the authors only and do not necessarily reflect those of the Funders. None of the Funders or granting authorities can be held responsible for them.

AUTHOR CONTRIBUTIONS

S.P. conceived and supervised the project; G.M.L., N.M.E., L.K., M.C., D.O., and S.P. performed research (simulations, data analysis, algorithms development, and analytical calculations); S.C., C.K., and T.F. provided experimental data; S.B.M. and S.C. curated data; A.K.E., A.I.L., and S.P. provided computational infrastructure; S.P., A.K.E., T.F., A.I.L., M.C., and S.B.M. provided funding; and G.M.L., N.M.E., L.K., M.C., A.I.L., and S.P. wrote the paper. All authors edited and commented on the paper and contributed to interpretation of results.

DECLARATION OF INTERESTS

The authors declare no competing interests.

SUPPLEMENTAL INFORMATION

Supplemental information can be found online at https://doi.org/10.1016/j.patter.2026.101619.

Received: January 17, 2026
Revised: April 20, 2026
Accepted: June 26, 2026
Published: July 23, 2026

REFERENCES

1. Barlow, H.B. (1972). Single units and sensation: A neuron doctrine for perceptual psychology? Perception *1*, 371–394. https://doi.org/10.1068/p010371.

2. Abbott, L.F., and Dayan, P. (1999). The effect of correlated variability on the accuracy of a population code. Neural Comput. *11*, 91–101. https://doi.org/10.1162/089976699300016827.

3. Nirenberg, S., Carcieri, S.M., Jacobs, A.L., and Latham, P.E. (2001). Retinal ganglion cells act largely as independent encoders. Nature *411*, 698–701. https://doi.org/10.1038/35079612.

4. Schneidman, E., Bialek, W., and Berry, M.J. (2003). Synergy, redundancy, and independence in population codes. J. Neurosci. *23*, 11539–11553. https://doi.org/10.1523/JNEUROSCI.23-37-11539.2003.

5. Averbeck, B.B., Latham, P.E., and Pouget, A. (2006). Neural correlations, population coding and computation. Nat. Rev. Neurosci. *7*, 358–366. https://doi.org/10.1038/nrn1888.

6. Panzeri, S., Moroni, M., Safaai, H., and Harvey, C.D. (2022). The structures and functions of correlations in neural population codes. Nat. Rev. Neurosci. *23*, 551–567. https://doi.org/10.1038/s41583-022-00606-4.

7. Borst, A., and Theunissen, F.E. (1999). Information theory and neural coding. Nat. Neurosci. *2*, 947–957. https://doi.org/10.1038/14731.

8. Quiroga, R.Q., and Panzeri, S. (2009). Extracting information from neuronal populations: information theory and decoding approaches. Nat. Rev. Neurosci. *10*, 173–185. https://doi.org/10.1038/nrn2578.

9. Fairhall, A., Shea-Brown, E., and Barreiro, A. (2012). Information theoretic approaches to understanding circuit function. Curr. Opin. Neurobiol. *22*, 653–659. https://doi.org/10.1016/j.conb.2012.06.005.

10. de Ruyter van Steveninck, R.R., Lewen, G.D., Strong, S.P., Koberle, R., and Bialek, W. (1997). Reproducibility and variability in neural spike trains. Science *275*, 1805–1808. https://doi.org/10.1126/science.275.5307.1805.

11. Kayser, C., Montemurro, M.A., Logothetis, N.K., and Panzeri, S. (2009). Spike-phase coding boosts and stabilizes information carried by spatial and temporal spike patterns. Neuron *61*, 597–608. https://doi.org/10.1016/j.neuron.2009.01.008.

12. Fuhrmann Alpert, G., Sun, F.T., Handwerker, D., D'Esposito, M., and Knight, R.T. (2007). Spatio-temporal information analysis of event-related bold responses. Neuroimage *34*, 1545–1561. https://doi.org/10.1016/j.neuroimage.2006.10.020.

13. Runyan, C.A., Piasini, E., Panzeri, S., and Harvey, C.D. (2017). Distinct timescales of population coding across cortex. Nature *548*, 92–96. https://doi.org/10.1038/nature23020.

14. Munn, B.R., Müller, E.J., Favre-Bulle, I., Scott, E., Lizier, J.T., Breakspear, M., and Shine, J.M. (2024). Multiscale organization of neuronal activity unifies scale-dependent theories of brain function. Cell *187*, 7303–7313.e15. https://doi.org/10.1016/j.cell.2024.10.004.

15. Atick, J.J., and Redlich, A.N. (1990). Towards a theory of early visual processing. Neural Comput. *2*, 308–320. https://doi.org/10.1162/neco.1990.2.3.308.

16. Hermundstad, A.M., Briguglio, J.J., Conte, M.M., Victor, J.D., Balasubramanian, V., and Tkačik, G. (2014). Variance predicts salience in central sensory processing. eLife *3*, e03722. https://doi.org/10.7554/eLife.03722.

17. Chalk, M., Marre, O., and Tkačik, G. (2018). Toward a unified theory of efficient, predictive, and sparse coding. Proc. Natl. Acad. Sci. USA *115*, 186–191. https://doi.org/10.1073/pnas.1711114115.

18. Gabrié, M., Manoel, A., Luneau, C., Barbier, J., Macris, N., Krzakala, F., and Zdeborová, L. (2018). Entropy and mutual information in models of deep neural networks. Adv. Neural Inf. Process. Syst. *31*, 1826–1836. https://proceedings.neurips.cc/paper_files/paper/2018/file/6d0f846348a856321729a2f36734d1a7-Paper.pdf.

19. Dan, Y., Alonso, J.-M., Usrey, W.M., and Reid, R.C. (1998). Coding of visual information by precisely correlated spikes in the lateral geniculate nucleus. Nat. Neurosci. *1*, 501–507. https://doi.org/10.1038/2217.

20. Pola, G., Thiele, A., Hoffmann, K.P., and Panzeri, S. (2003). An exact method to quantify the information transmitted by different mechanisms of correlational coding. Network *14*, 35–60. https://doi.org/10.1088/0954-898X/14/1/303.

21. Golledge, H.D.R., Panzeri, S., Zheng, F., Pola, G., Scannell, J.W., Giannikopoulos, D.V., Mason, R.J., Tovée, M.J., and Young, M.P. (2003). Correlations, feature-binding and population coding in primary visual cortex. Neuroreport *14*, 1045–1050. https://doi.org/10.1097/01.wnr.0000073681.00308.9c.

22. Williams, P.L., and Beer, R.D. (2010). Nonnegative decomposition of multivariate information. Preprint at arXiv. https://doi.org/10.48550/arXiv.1004.2515.

23. Luppi, A.I., Rosas, F.E., Mediano, P.A.M., Menon, D.K., and Stamatakis, E.A. (2024). Information decomposition and the informational architecture of the brain. Trends Cogn. Sci. *28*, 352–368. https://doi.org/10.1016/j.tics.2023.11.005.

24. Panzeri, S., and Treves, A. (1996). Analytical estimates of limited sampling biases in different information measures. Network *7*, 87–107. https://doi.org/10.1088/0954-898X/7/1/006.

25. Paninski, L. (2003). Estimation of entropy and mutual information. Neural Comput. *15*, 1191–1253. https://doi.org/10.1162/089976603321780272.

26. Nemenman, I., Bialek, W., and de Ruyter van Steveninck, R. (2004). Entropy and information in neural spike trains: Progress on the sampling problem. Phys. Rev. E *69*, 056111. https://doi.org/10.1103/PhysRevE.69.056111.

27. Panzeri, S., Senatore, R., Montemurro, M.A., and Petersen, R.S. (2007). Correcting for the sampling bias problem in spike train information measures. J. Neurophysiol. *98*, 1064–1072. https://doi.org/10.1152/jn.00559.2007.

28. Kraskov, A., Stögbauer, H., and Grassberger, P. (2004). Estimating mutual information. Phys. Rev. E *69*, 066138. https://doi.org/10.1103/PhysRevE.69.066138.

29. Liardi, A., Rosas, F.E., Carhart-Harris, R.L., Blackburne, G., Bor, D., and Mediano, P.A.M. (2025). Null models for comparing information decomposition across complex systems. PLoS Comput. Biol. *21*, e1013629. https://doi.org/10.1371/journal.pcbi.1013629.

30. Venkatesh, P., Bennett, C., Gale, S., Ramirez, T., Heller, G., Durand, S., Olsen, S., and Mihalas, S. (2023). Gaussian partial information decomposition: Bias correction and application to high-dimensional data. Adv. Neural Inf. Process. Syst. *36*, 74602–74635.

31. Barà, C., Antonacci, Y., Iovino, M., Lazic, I., and Faes, L. (2025). Partial information decomposition for discrete target and continuous source random variables. Phys. Rev. E *112*, L012301. https://doi.org/10.1103/PhysRevE.112.L012301.

32. Harder, M., Salge, C., and Polani, D. (2013). Bivariate measure of redundant information. Phys. Rev. E *87*, 012130. https://doi.org/10.1103/PhysRevE.87.012130.

33. Bertschinger, N., Rauh, J., Olbrich, E., Jost, J., and Ay, N. (2014). Quantifying unique information. Entropy *16*, 2161–2183. https://doi.org/10.3390/e16042161.

34. Barrett, A.B. (2015). Exploration of synergistic and redundant information sharing in static and dynamical Gaussian systems. Phys. Rev. E *91*, 052802. https://doi.org/10.1103/PhysRevE.91.052802.

35. Sherrill, S.P., Timme, N.M., Beggs, J.M., and Newman, E.L. (2021). Partial information decomposition reveals that synergistic neural integration is greater downstream of recurrent information flow in organotypic cortical cultures. PLoS Comput. Biol. *17*, e1009196. https://doi.org/10.1371/journal.pcbi.1009196.

36. Francis, N.A., Mukherjee, S., Koçillari, L., Panzeri, S., Babadi, B., and Kanold, P.O. (2022). Sequential transmission of task-relevant information in cortical neuronal networks. Cell Rep. *39*, 110878. https://doi.org/10.1016/j.celrep.2022.110878.

37. Varley, T.F., Sporns, O., Schaffelhofer, S., Scherberger, H., and Dann, B. (2023). Information-processing dynamics in neural networks of macaque cerebral cortex reflect cognitive state and behavior. Proc. Natl. Acad. Sci. USA *120*, e2207677120. https://doi.org/10.1073/pnas.2207677120.

CellPress
OPEN ACCESS

38. Shannon, C.E. (1948). A mathematical theory of communication. Bell Syst. Tech. J. *27*, 379–423. https://doi.org/10.1002/j.1538-7305.1948.tb01338.x.

39. Cover, T.M., and Thomas, J.A. (2006). Elements of Information Theory (Wiley & Sons). https://doi.org/10.1002/0471200611.

40. Lizier, J.T., Bertschinger, N., Jost, J., and Wibral, M. (2018). Information decomposition of target effects from multi-source interactions: Perspectives on previous, current and future work. Entropy *20*, 307. https://doi.org/10.3390/e20040307.

41. Kolchinsky, A. (2022). A novel approach to the partial information decomposition. Entropy *24*, 403. https://doi.org/10.3390/e24030403.

42. Gomes, A.F.C., and Figueiredo, M.A.T. (2024). A measure of synergy based on union information. Entropy *26*, 271. https://doi.org/10.3390/e26030271.

43. Makkeh, A., Gutknecht, A.J., and Wibral, M. (2021). Introducing a differentiable measure of pointwise shared information. Phys. Rev. E *103*, 032149. https://doi.org/10.1103/PhysRevE.103.032149.

44. Pakman, A., Nejatbakhsh, A., Gilboa, D., Makkeh, A., Mazzucato, L., Wibral, M., and Schneidman, E. (2021). Estimating the unique information of continuous variables. Adv. Neural Inf. Process. Syst. *34*, 20295–20307. https://proceedings.neurips.cc/paper/2021/hash/a9a1d5317a33ae8cef33961c34144f84-Abstract.html.

45. Finn, C., and Lizier, J.T. (2018). Probability mass exclusions and the directed components of mutual information. Entropy *20*, 826. https://doi.org/10.3390/e20110826.

46. Rauh, J., Banerjee, P.K., Olbrich, E., Montúfar, G., and Jost, J. (2023). Continuity and additivity properties of information decompositions. Int. J. Approx. Reason. *161*, 108979. https://doi.org/10.1016/j.ijar.2023.108979.

47. Wollstadt, P., Rathbun, D.L., Usrey, W.M., Bastos, A.M., Lindner, M., Priesemann, V., and Wibral, M. (2023). Information-theoretic analyses of neural data to minimize the effect of researchers' assumptions in predictive coding studies. PLoS Comput. Biol. *19*, e1011567. https://doi.org/10.1371/journal.pcbi.1011567.

48. Lemke, S.M., Celotto, M., Maffulli, R., Ganguly, K., and Panzeri, S. (2024). Information flow between motor cortex and striatum reverses during skill learning. Curr. Biol. *34*, 1831–1843.e7. https://doi.org/10.1016/j.cub.2024.03.023.

49. Luppi, A.I., Mediano, P.A.M., Rosas, F.E., Holland, N., Fryer, T.D., O'Brien, J.T., Rowe, J.B., Menon, D.K., Bor, D., and Stamatakis, E.A. (2022). A synergistic core for human brain evolution and cognition. Nat. Neurosci. *25*, 771–782. https://doi.org/10.1038/s41593-022-01070-0.

50. Celotto, M., Bím, J., Tlaie, A., De Feo, V., Toso, A., Lemke, S., Chicharro, D., Nili, H., Bieler, M., Hanganu-Opatz, I., et al. (2023). An information-theoretic quantification of the content of communication between brain regions. Adv. Neural Inf. Process. Syst. *36*, 64213–64265.

51. Greco, A., Moser, J., Preissl, H., and Siegel, M. (2024). Predictive learning shapes the representational geometry of the human brain. Nat. Commun. *15*, 9670. https://doi.org/10.1038/s41467-024-54032-4.

52. Chen, Z., Fang, Y., Yan, W., Wang, J., Li, J., and Hou, F. (2020). Research on the MEG of depression patients based on multivariate TE partial information decomposition. IOP Conf. Ser. Mater. Sci. Eng. *768*, 052053. https://doi.org/10.1088/1757-899X/768/5/052053.

53. Reich, D.S., Mechler, F., and Victor, J.D. (2001). Independent and redundant information in nearby cortical neurons. Science *294*, 2566–2568. https://doi.org/10.1126/science.1065839.

54. Schneidman, E., Berry, M.J., Segev, R., and Bialek, W. (2006). Weak pairwise correlations imply strongly correlated network states in a neural population. Nature *440*, 1007–1012. https://doi.org/10.1038/nature04701.

55. Schneidman, E., Bialek, W., and II, M.J.B. (2002). An information theoretic approach to the functional classification of neurons. Adv. Neural Inf. Process. Syst. *15*, 197–204.

56. Ohiorhenuan, I.E., Mechler, F., Purpura, K.P., Schmid, A.M., Hu, Q., and Victor, J.D. (2010). Sparse coding and high-order correlations in fine-scale cortical networks. Nature *466*, 617–621. https://doi.org/10.1038/nature09178.

57. Emanuel, A.J., Lehnert, B.P., Panzeri, S., Harvey, C.D., and Ginty, D.D. (2021). Cortical responses to touch reflect subcortical integration of LTMR signals. Nature *600*, 680–685. https://doi.org/10.1038/s41586-021-04094-x.

58. Kuan, A.T., Bondanelli, G., Driscoll, L.N., Han, J., Kim, M., Hildebrand, D.G.C., Graham, B.J., Wilson, D.E., Thomas, L.A., Panzeri, S., et al. (2024). Synaptic wiring motifs in posterior parietal cortex support decision-making. Nature *627*, 367–373. https://doi.org/10.1038/s41586-024-07088-7.

59. Stevens, C., and Zador, A. (1995). Information through a spiking neuron. Adv. Neural Inf. Process. Syst. *8*, 75–81.

60. Strong, S.P., Koberle, R., de Ruyter van Steveninck, R.R., and Bialek, W. (1998). Entropy and information in neural spike trains. Phys. Rev. Lett. *80*, 197–200. https://doi.org/10.1103/PhysRevLett.80.197.

61. Granot-Atedgi, E., Tkačik, G., Segev, R., and Schneidman, E. (2013). Stimulus-dependent maximum entropy models of neural population codes. PLoS Comput. Biol. *9*, e1002922. https://doi.org/10.1371/journal.pcbi.1002922.

62. Nigam, S., Pojoga, S., and Dragoi, V. (2019). Synergistic coding of visual information in columnar networks. Neuron *104*, 402–411.e4. https://doi.org/10.1016/j.neuron.2019.07.006.

63. Curreli, S., Bonato, J., Romanzi, S., Panzeri, S., and Fellin, T. (2022). Complementary encoding of spatial information in hippocampal astrocytes. PLoS Biol. *20*, e3001530. https://doi.org/10.1371/journal.pbio.3001530.

64. Lorenz, G.M., Engel, N.M., Celotto, M., Koçillari, L., Curreli, S., Fellin, T., and Panzeri, S. (2025). MINT: a toolbox for the analysis of multivariate neural information coding and transmission. PLoS Comput. Biol. *21*, e1012934. https://doi.org/10.1371/journal.pcbi.1012934.

65. Timme, N.M., Ito, S., Myroshnychenko, M., Nigam, S., Shimono, M., Yeh, F.-C., Hottowy, P., Litke, A.M., and Beggs, J.M. (2016). High-degree neurons feed cortical computations. PLoS Comput. Biol. *12*, e1004858. https://doi.org/10.1371/journal.pcbi.1004858.

66. Pica, G., Piasini, E., Safaai, H., Runyan, C., Harvey, C., Diamond, M.E., Kayser, C., Fellin, T., and Panzeri, S. (2017). Quantifying how much sensory information in a neural code is relevant for behavior. Adv. Neural Inf. Process. Syst. *30*, 3686–3696.

67. Wibral, M., Finn, C., Wollstadt, P., Lizier, J., and Priesemann, V. (2017). Quantifying information modification in developing neural networks via partial information decomposition. Entropy *19*, 494. https://doi.org/10.3390/e19090494.

68. Koçillari, L., Celotto, M., Francis, N.A., Mukherjee, S., Babadi, B., Kanold, P.O., and Panzeri, S. (2023). Behavioural relevance of redundant and synergistic stimulus information between functionally connected neurons in mouse auditory cortex. Brain Inform. *10*, 34. https://doi.org/10.1186/s40708-023-00212-9.

69. Douchamps, V., di Volo, M., Torcini, A., Battaglia, D., and Goutagny, R. (2024). Gamma oscillatory complexity conveys behavioral information in hippocampal networks. Nat. Commun. *15*, 1849. https://doi.org/10.1038/s41467-024-46012-5.

70. Panzeri, S., Petersen, R.S., Schultz, S.R., Lebedev, M., and Diamond, M.E. (2001). The role of spike timing in the coding of stimulus location in rat somatosensory cortex. Neuron *29*, 769–777. https://doi.org/10.1016/S0896-6273(01)00251-3.

71. Tolhurst, D.J., Movshon, J.A., and Dean, A.F. (1983). The statistical reliability of signals in single neurons in cat and monkey visual cortex. Vision Res. *23*, 775–785. https://doi.org/10.1016/0042-6989(83)90200-6.

72. Kayser, C., Logothetis, N.K., and Panzeri, S. (2010). Millisecond encoding precision of auditory cortex neurons. Proc. Natl. Acad. Sci. USA *107*, 16976–16981. https://doi.org/10.1073/pnas.1012656107.

73. Panzeri, S., Brunel, N., Logothetis, N.K., and Kayser, C. (2010). Sensory neural codes using multiplexed temporal scales. Trends Neurosci. *33*, 111–120. https://doi.org/10.1016/j.tins.2009.12.001.

74. Pessoa, L., and Padmala, S. (2007). Decoding near-threshold perception of fear from distributed single-trial brain activation. Cereb. Cortex *17*, 691–701. https://doi.org/10.1093/cercor/bhk020.

75. Belitski, A., Gretton, A., Magri, C., Murayama, Y., Montemurro, M.A., Logothetis, N.K., and Panzeri, S. (2008). Low-Frequency Local Field Potentials and Spikes in Primary Visual Cortex Convey Independent Visual Information. J. Neurosci. *28*, 5696–5709. https://doi.org/10.1523/JNEUROSCI.0009-08.2008.

76. Ostwald, D., Porcaro, C., and Bagshaw, A.P. (2010). An information theoretic approach to eeg–fmri integration of visually evoked responses. Neuroimage *49*, 498–516. https://doi.org/10.1016/j.neuroimage.2009.07.038.

77. Park, H., Arazi, A., Talluri, B.C., Celotto, M., Panzeri, S., Stocker, A.A., and Donner, T.H. (2025). Confirmation bias through selective readout of information encoded in human parietal cortex. Nat. Commun. *16*, 5391. https://doi.org/10.1038/s41467-025-61010-x.

78. Faes, L., Marinazzo, D., and Stramaglia, S. (2017). Multiscale information decomposition: Exact computation for multivariate Gaussian processes. Entropy *19*, 408. https://doi.org/10.3390/e19080408.

79. Besserve, M., Lowe, S.C., Logothetis, N.K., Schölkopf, B., and Panzeri, S. (2015). Shifts of gamma phase across primary visual cortical sites reflect dynamic stimulus-modulated information transfer. PLoS Biol. *13*, e1002257. https://doi.org/10.1371/journal.pbio.1002257.

80. Cai, T.T., Liang, T., and Zhou, H.H. (2015). Law of log determinant of sample covariance matrix and optimal estimation of differential entropy for high-dimensional Gaussian distributions. J. Multivar. Anal. *137*, 161–172. https://doi.org/10.1016/j.jmva.2015.02.003.

81. Goodman, N.R. (1963). The distribution of the determinant of a complex wishart distributed matrix. Ann. Math. Stat. *34*, 178–180. https://doi.org/10.1214/aoms/1177704251.

82. Papana, A., Kyrtsou, C., Kugiumtzis, D., and Diks, C. (2017). Assessment of resampling methods for causality testing: A note on the us inflation behavior. PLoS One *12*, e0180852. https://doi.org/10.1371/journal.pone.0180852.

83. Treves, A., and Panzeri, S. (1995). The Upward Bias in Measures of Information Derived from Limited Data Samples. Neural Comput. *7*, 399–407. https://doi.org/10.1162/neco.1995.7.2.399.

84. Makkeh, A., Theis, D.O., and Vicente, R. (2018). BROJA-2PID: A Robust Estimator for Bivariate Partial Information Decomposition. Entropy *20*, 271. https://doi.org/10.3390/e20040271.

85. Glasser, M.F., Sotiropoulos, S.N., Wilson, J.A., Coalson, T.S., Fischl, B., Andersson, J.L., Xu, J., Jbabdi, S., Webster, M., Polimeni, J.R., et al. (2013). The minimal preprocessing pipelines for the human connectome project. Neuroimage *80*, 105–124. https://doi.org/10.1016/j.neuroimage.2013.04.127.

86. Van Essen, D.C., Smith, S.M., Barch, D.M., Behrens, T.E.J., Yacoub, E., and Ugurbil, K.; WU-Minn HCP Consortium (2013). The wu-minn human connectome project: an overview. Neuroimage *80*, 62–79. https://doi.org/10.1016/j.neuroimage.2013.05.041.

87. Whitfield-Gabrieli, S., and Nieto-Castanon, A. (2012). Conn: a functional connectivity toolbox for correlated and anticorrelated brain networks. Brain Connect. *2*, 125–141. https://doi.org/10.1089/brain.2012.0073.

88. Behzadi, Y., Restom, K., Liau, J., and Liu, T.T. (2007). A component based noise correction method (CompCor) for BOLD and perfusion based fMRI. Neuroimage *37*, 90–101. https://doi.org/10.1016/j.neuroimage.2007.04.042.

89. Schaefer, A., Kong, R., Gordon, E.M., Laumann, T.O., Zuo, X.-N., Holmes, A.J., Eickhoff, S.B., and Yeo, B.T.T. (2018). Local-global parcellation of the human cerebral cortex from intrinsic functional connectivity MRI. Cereb. Cortex *28*, 3095–3114. https://doi.org/10.1093/cercor/bhx179.

90. Combrisson, E., Basanisi, R., Neri, M., Auzias, G., Petri, G., Marinazzo, D., Panzeri, S., and Brovelli, A. (2025). Higher-order and distributed synergistic functional interactions encode information gain in goal-directed learning. Nat. Commun. *16*, 7179. https://doi.org/10.1038/s41467-025-62507-1.

91. Van Dijk, K.R.A., Hedden, T., Venkataraman, A., Evans, K.C., Lazar, S.W., and Buckner, R.L. (2010). Intrinsic functional connectivity as a tool for human connectomics: theory, properties, and optimization. J. Neurophysiol. *103*, 297–321. https://doi.org/10.1152/jn.00783.2009.

92. Birn, R.M., Molloy, E.K., Patriat, R., Parker, T., Meier, T.B., Kirk, G.R., Nair, V.A., Meyerand, M.E., and Prabhakaran, V. (2013). The effect of scan length on the reliability of resting-state fMRI connectivity estimates. Neuroimage *83*, 550–558. https://doi.org/10.1016/j.neuroimage.2013.05.099.

93. Martignon, L., Deco, G., Laskey, K., Diamond, M., Freiwald, W., and Vaadia, E. (2000). Neural coding: higher-order temporal patterns in the neurostatistics of cell assemblies. Neural Comput. *12*, 2621–2653. https://doi.org/10.1162/089976600300014872.

94. Shahidi, N., Andrei, A.R., Hu, M., and Dragoi, V. (2019). High-order coordination of cortical spiking activity modulates perceptual accuracy. Nat. Neurosci. *22*, 1148–1158. https://doi.org/10.1038/s41593-019-0406-3.

95. Varley, T.F., Pope, M., Grazia, M., Joshua, and Sporns, O. (2023). Partial entropy decomposition reveals higher-order information structures in human brain activity. Proc. Natl. Acad. Sci. USA *120*, e2300888120. https://doi.org/10.1073/pnas.2300888120.

96. Yu, S., Yang, H., Nakahara, H., Santos, G.S., Nikolić, D., and Plenz, D. (2011). Higher-order interactions characterized in cortical activity. J. Neurosci. *31*, 17514–17526. https://doi.org/10.1523/JNEUROSCI.3127-11.2011.

97. Bertschinger, N., Rauh, J., Olbrich, E., and Jost, J. (2013). Shared information—new insights and problems in decomposing information in complex systems. In Proceedings of the European Conference on Complex Systems 2012. Springer Proceedings in Complexity, T. Gilbert, M. Kirkilionis, and G. Nicolis, eds. (Springer), pp. 251–269. https://doi.org/10.1007/978-3-319-00395-5.

98. Proca, A.M., Rosas, F.E., Luppi, A.I., Bor, D., Crosby, M., and Mediano, P.A.M. (2024). Synergistic information supports modality integration and flexible learning in neural networks solving multiple tasks. PLoS Comput. Biol. *20*, e1012178. https://doi.org/10.1371/journal.pcbi.1012178.

99. Varley, T.F., Pope, M., Faskowitz, J., and Sporns, O. (2023). Multivariate information theory uncovers synergistic subsystems of the human cerebral cortex. Commun. Biol. *6*, 451. https://doi.org/10.1038/s42003-023-04843-w.

100. Chan, T.E., Stumpf, M.P.H., and Babtie, A.C. (2017). Gene regulatory network inference from single-cell data using multivariate information measures. Cell Syst. *5*, 251–267.e3. https://doi.org/10.1016/j.cels.2017.08.014.

101. Wollstadt, P., Schmitt, S., and Wibral, M. (2023). A rigorous information-theoretic definition of redundancy and relevancy in feature selection based on (partial) information decomposition. J. Mach. Learn. Res. *24*, 1–44.

102. Tax, T., Mediano, P., and Shanahan, M. (2017). The partial information decomposition of generative neural network models. Entropy *19*, 474. https://doi.org/10.3390/e19090474.

103. Scagliarini, T., Faes, L., Marinazzo, D., Stramaglia, S., and Mantegna, R.N. (2020). Synergistic information transfer in the global system of financial markets. Entropy *22*, 1000. https://doi.org/10.3390/e22091000.

104. Valente, M., Pica, G., Bondanelli, G., Moroni, M., Runyan, C.A., Morcos, A.S., Harvey, C.D., and Panzeri, S. (2021). Correlations enhance the behavioral readout of neural population activity in association cortex. Nat. Neurosci. *24*, 975–986. https://doi.org/10.1038/s41593-021-00845-1.

105. Cunningham, J.P., and Yu, B.M. (2014). Dimensionality reduction for large-scale neural recordings. Nat. Neurosci. *17*, 1500–1509. https://doi.org/10.1038/nn.3776.

CellPress
OPEN ACCESS

106. Iurilli, G., and Datta, S.R. (2017). Population coding in an innately relevant olfactory area. Neuron *93*, 1180–1197.e7. https://doi.org/10.1016/j.neuron.2017.02.010.

107. Sharpee, T.O., and Berkowitz, J.A. (2019). Linking neural responses to behavior with information-preserving population vectors. Curr. Opin. Behav. Sci. *29*, 37–44. https://doi.org/10.1016/j.cobeha.2019.03.004.

108. Froudarakis, E., Berens, P., Ecker, A.S., Cotton, R.J., Sinz, F.H., Yatsenko, D., Saggau, P., Bethge, M., and Tolias, A.S. (2014). Population code in mouse v1 facilitates readout of natural scenes through increased sparseness. Nat. Neurosci. *17*, 851–857. https://doi.org/10.1038/nn.3707.

109. James, R., and Crutchfield, J. (2017). Multivariate dependence beyond shannon information. Entropy *19*, 531. https://doi.org/10.3390/e19100531.

110. Perrone, P., and Ay, N. (2016). Hierarchical quantification of synergy in channels. Front. Robot. AI *2*, 35. https://doi.org/10.3389/frobt.2015.00035.

111. Glasser, M.F., Coalson, T.S., Robinson, E.C., Hacker, C.D., Harwell, J., Yacoub, E., Ugurbil, K., Andersson, J., Beckmann, C.F., Jenkinson, M., et al. (2016). A multi-modal parcellation of human cerebral cortex. Nature *536*, 171–178. https://doi.org/10.1038/nature18933.

112. Engel, N. M., Lorenz, G. M., and Celotto, M. (2026). panzerilab/Sampling_bias_corrections_Syn_Red: v1.2 of Sampling bias. Zenodo. doi:https://doi.org/10.5281/zenodo.20085438.

Patterns, Volume *7*

Supplemental information

Sampling bias corrections for discrete and Gaussian partial information decompositions

Gabriel Matías Lorenz, Nicola Marie Engel, Loren Koçillari, Marco Celotto, Davide Orsenigo, Sebastiano Curreli, Simone Blanco Malerba, Andreas K. Engel, Christoph Kayser, Tommaso Fellin, Andrea I. Luppi, and Stefano Panzeri

Supplemental Information

Content

Supplemental Figures

Figure S1. Schematic of the Partial Information Decomposition with two sources.
Figure S2. Bias properties and bias corrections for the sum of unique information terms.
Figure S3. Bias of the plugin PID values and residual bias of the bias-corrected PID values as a function of the number of parameters defining the probability distributions.
Figure S4. Dependence of the bias of the discrete BROJA PID on the number of samples N used to compute it.
Figure S5. Bias correction performance for the discrete BROJA PID.
Figure S6. Bias correction performance for the discrete I_{min}.
Figure S7. Bias correction performance for the discrete I_{MMI}.
Figure S8. Residual bias across information levels.
Figure S9. Bias and performance of bias corrections for the Gaussian gPID as a function of the number of dimensions d.
Figure S10. Bias and performance of bias corrections for the Gaussian gPID as a function of the number of samples N.
Figure S11. Bias corrections performance for gPID.
Figure S12. Comparison of the residual bias of the discrete merged correction using optimized and equal weighting.
Figure S13. Using a Gaussian PID on discrete neural data.
Figure S14. Comparison of analytical expressions of the bias for the Gaussian entropy, and evaluations of the residual mutual information bias.
Figure S15. Tolerance analysis performed on the merged-corrected residual synergy and redundancy bias and on the plugin synergy and redundancy bias.
Figure S16. Comparison of PID bias corrections on each of the three full real neural datasets and on the same datasets each recomputed with half the trials.
Figure S17. Further analyses of the human resting-state fMRI dataset (dataset 4).
Figure S18. Evaluation of the bias and the performance of bias correction methods when computing discrete BROJA PID of two 10-dimensional sources with an SVM dimensionality reduction.

Supplemental Tables

Table S1. Summary of support sizes and simulation parameters used across all figures, including main text and supplementary figures.
Table S2. Overview of simulation parameters for simulated scenarios of discrete and Gaussian simulations.

Supplementary Notes

Supplementary Note 1. Other discrete PID measures.
Supplementary Note 2. Key properties of PID components for two source variables.
Supplementary Note 3. Illustration of the origin of the bias.
Supplementary Note 4. Further results of simulations and bias corrections.
Supplementary Note 5. Application of Gaussian PID to simulated spike count data.
Supplementary Note 6. Analytical considerations on the bias of discrete and Gaussian PIDs.

Supplementary Note 7. Study of how to scale up calculations and bias corrections of discrete PID using dimensionality reduction.
Supplementary Note 8. Analytical derivation of Red_3 and Syn_3 bias.

Figure S1

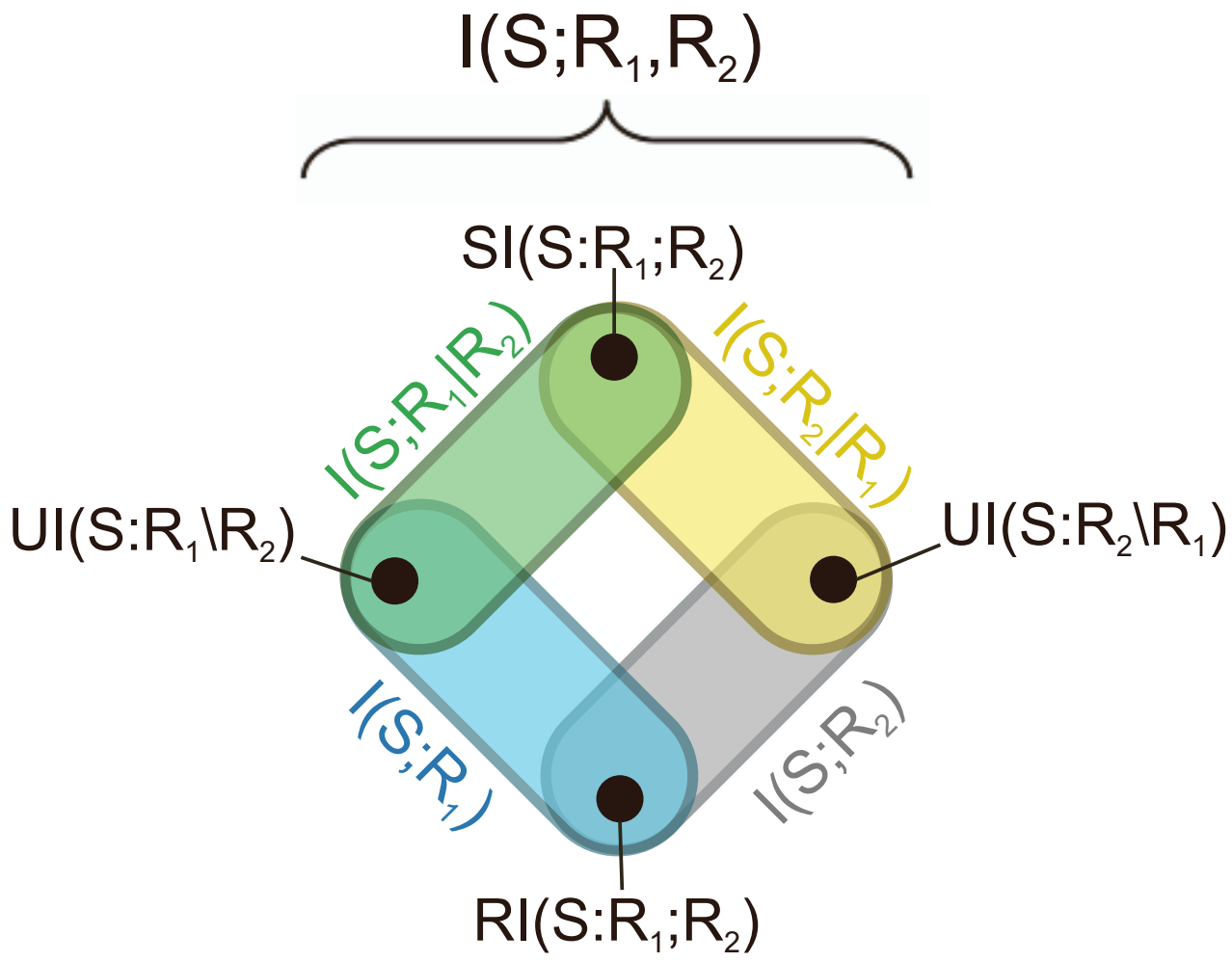

I(S;R1,R2)
SI(S:R1;R2)
I(S;R1|R2)
I(S;R2|R1)
UI(S:R1\R2)
UI(S:R2\R1)
I(S;R1)
I(S;R2)
RI(S:R1;R2)

Figure S2

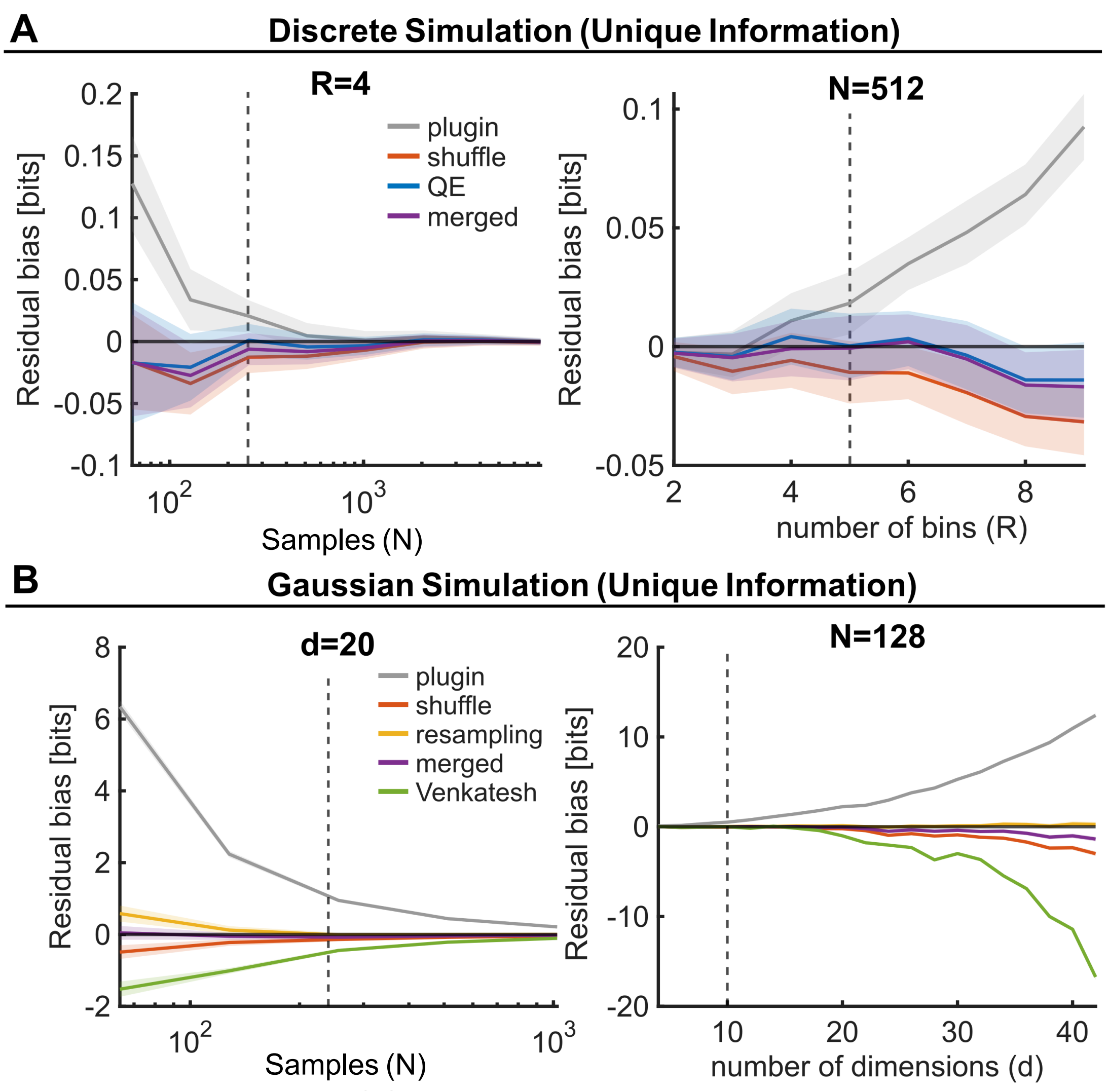
A
Discrete Simulation (Unique Information)
R=4
N=512
plugin
shuffle
QE
merged
Residual bias [bits]
Samples (N)
number of bins (R)
B
Gaussian Simulation (Unique Information)
d=20
N=128
plugin
shuffle
resampling
merged
Venkatesh
Residual bias [bits]
Samples (N)
number of dimensions (d)

Figure S3

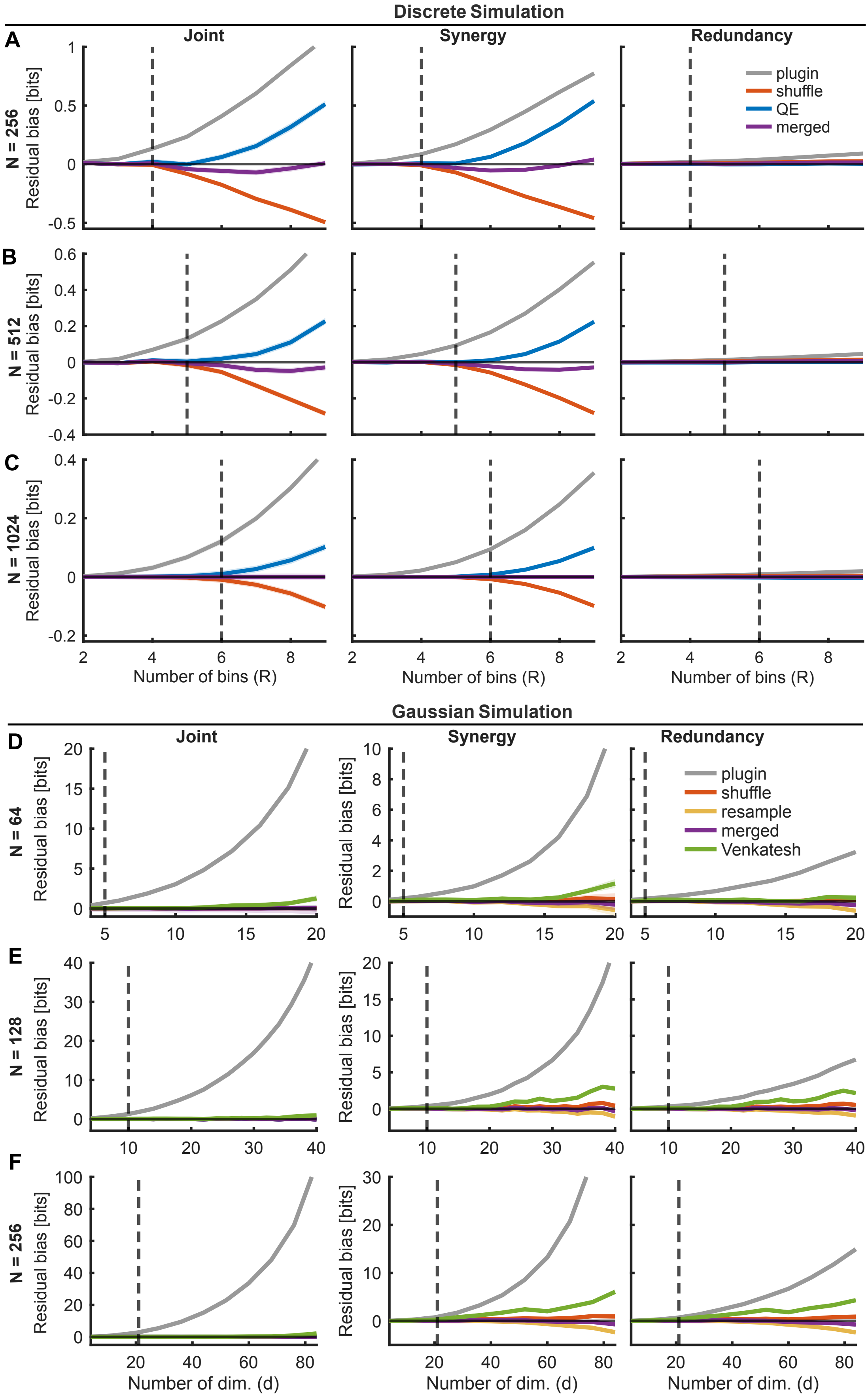

Discrete Simulation
A
B
C
Joint
Synergy
Redundancy
N = 256
N = 512
N = 1024
Residual bias [bits]
plugin
shuffle
QE
merged
Number of bins (R)
Gaussian Simulation
D
E
F
N = 64
N = 128
N = 256
plugin
shuffle
resample
merged
Venkatesh
Number of dim. (d)

Figure S4

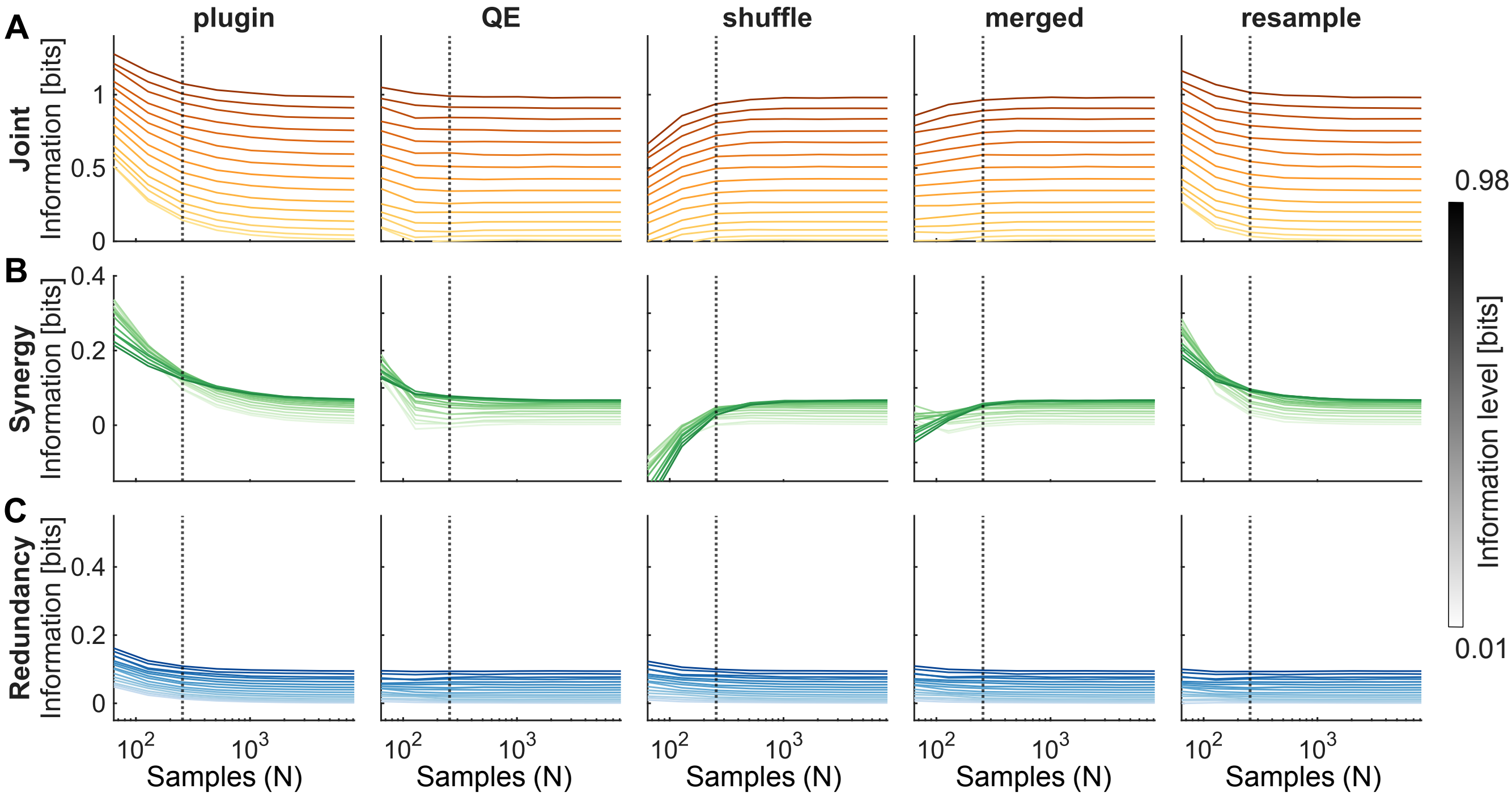

A
B
C
plugin
QE
shuffle
merged
resample
Joint
Information [bits]
Synergy
Information [bits]
Redundancy
Information [bits]
0
0.5
1
0.4
0.2
0
0.4
0.2
0
10^2
10^3
Samples (N)
0.98
0.01
Information level [bits]

Figure S5

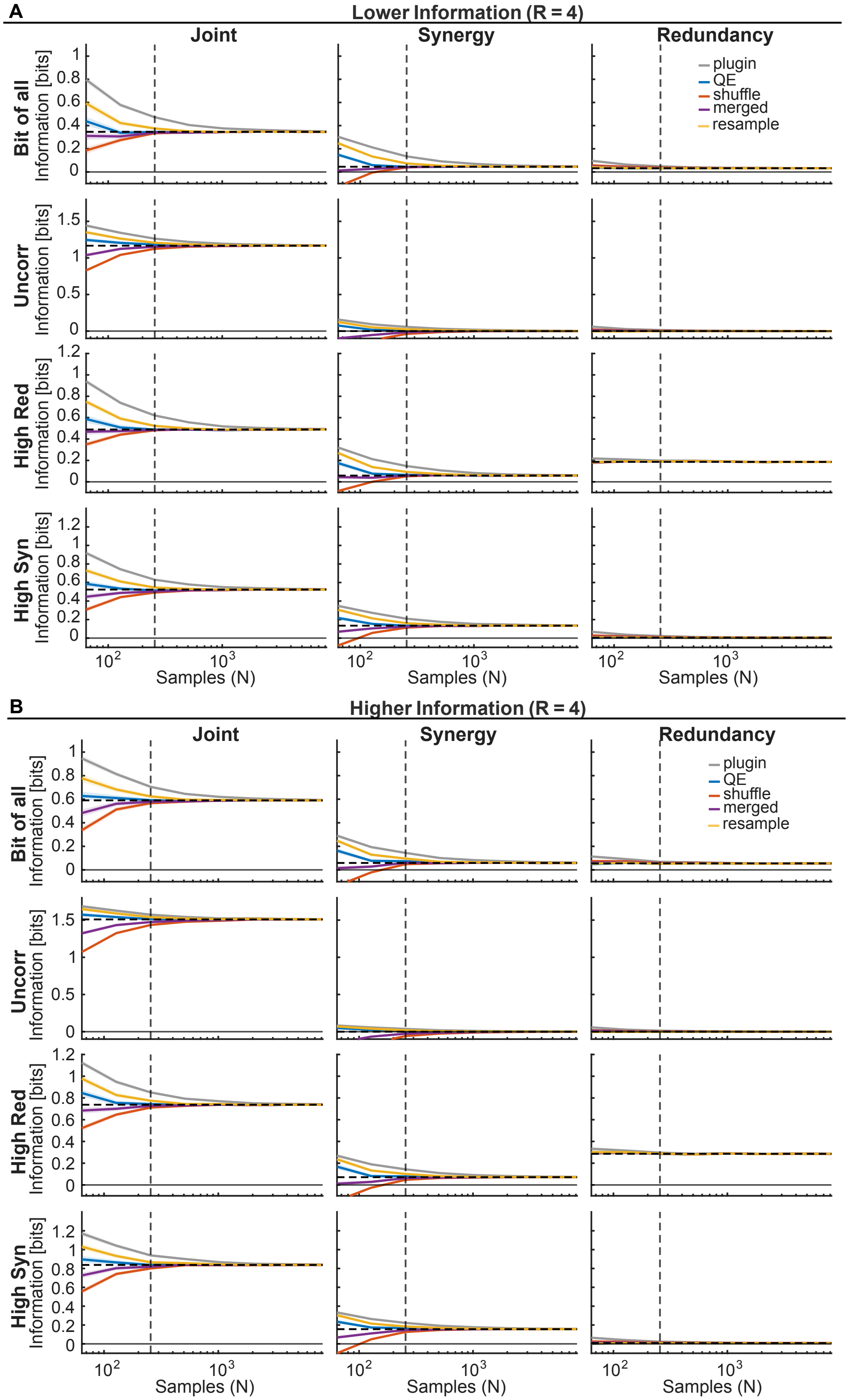

A
Lower Information (R = 4)
Joint
Synergy
Redundancy
plugin
QE
shuffle
merged
resample
Bit of all
Uncorr
High Red
High Syn
Information [bits]
Samples (N)
B
Higher Information (R = 4)

Figure S6

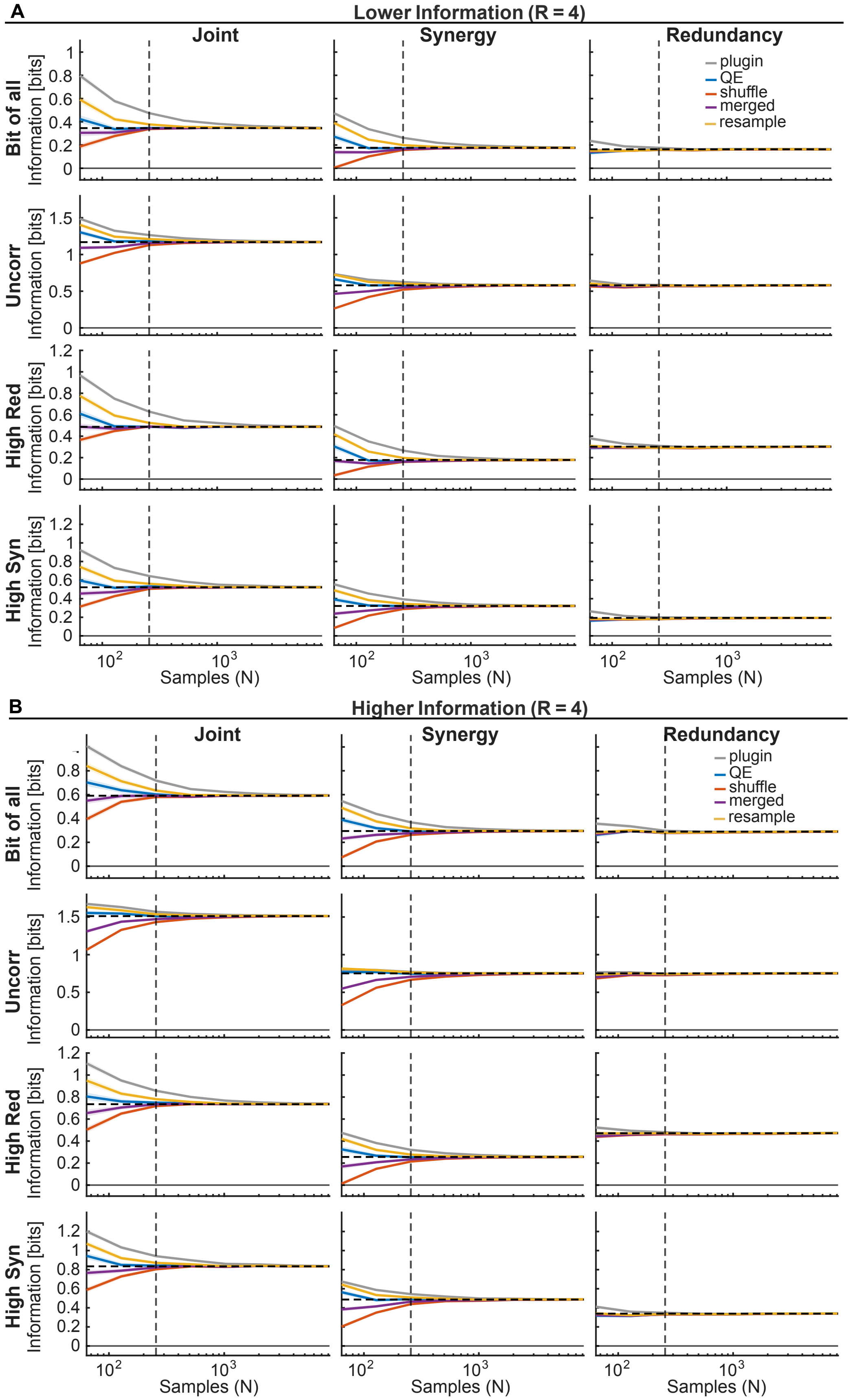
A
Lower Information (R = 4)
Joint
Synergy
Redundancy
plugin
QE
shuffle
merged
resample
Bit of all
Uncorr
High Red
High Syn
Information [bits]
Samples (N)
B
Higher Information (R = 4)

Figure S7

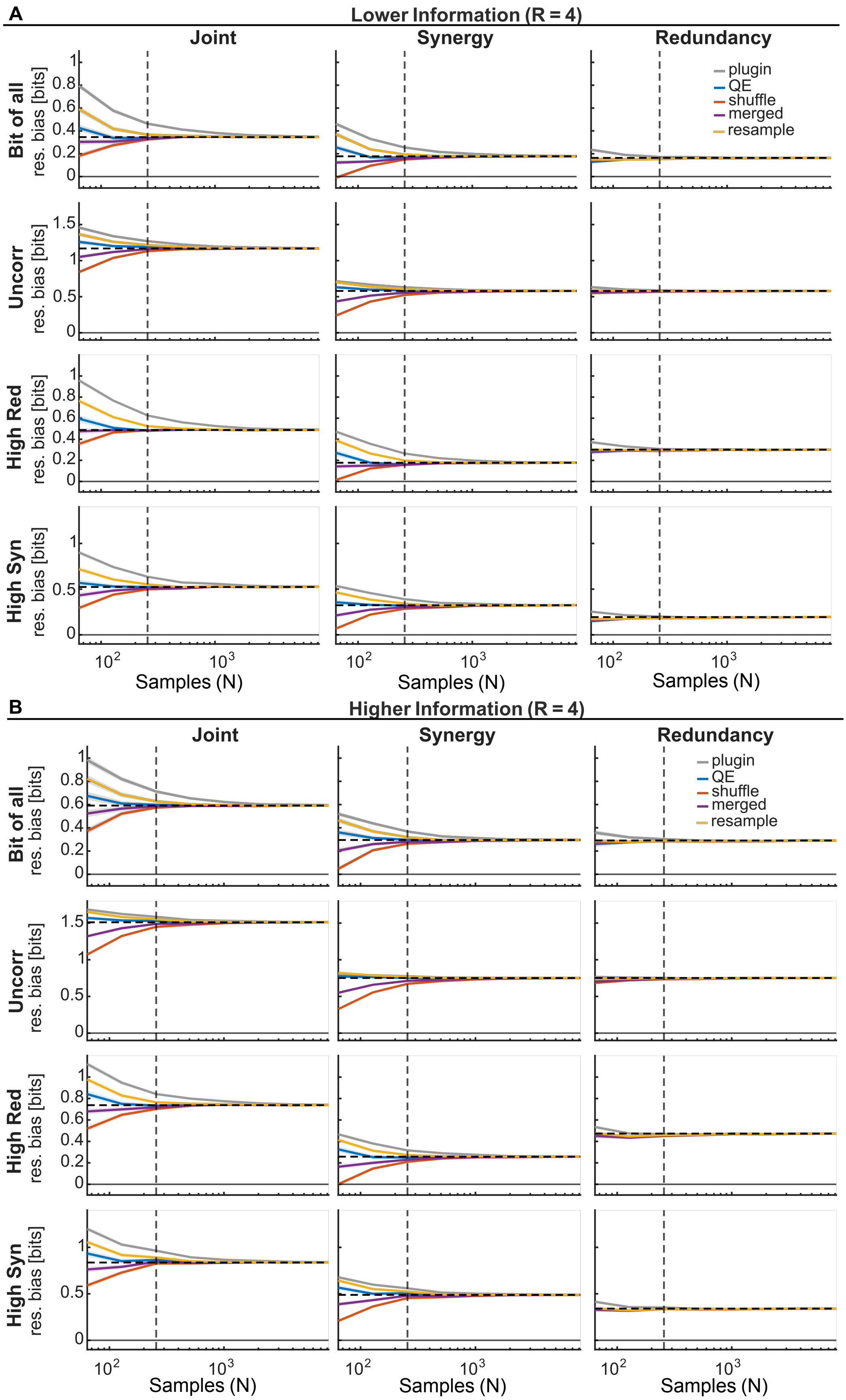
A
Lower Information (R = 4)
Joint
Synergy
Redundancy
plugin
QE
shuffle
merged
resample
Bit of all
Uncorr
High Red
High Syn
res. bias [bits]
Samples (N)
B
Higher Information (R = 4)

Figure S8

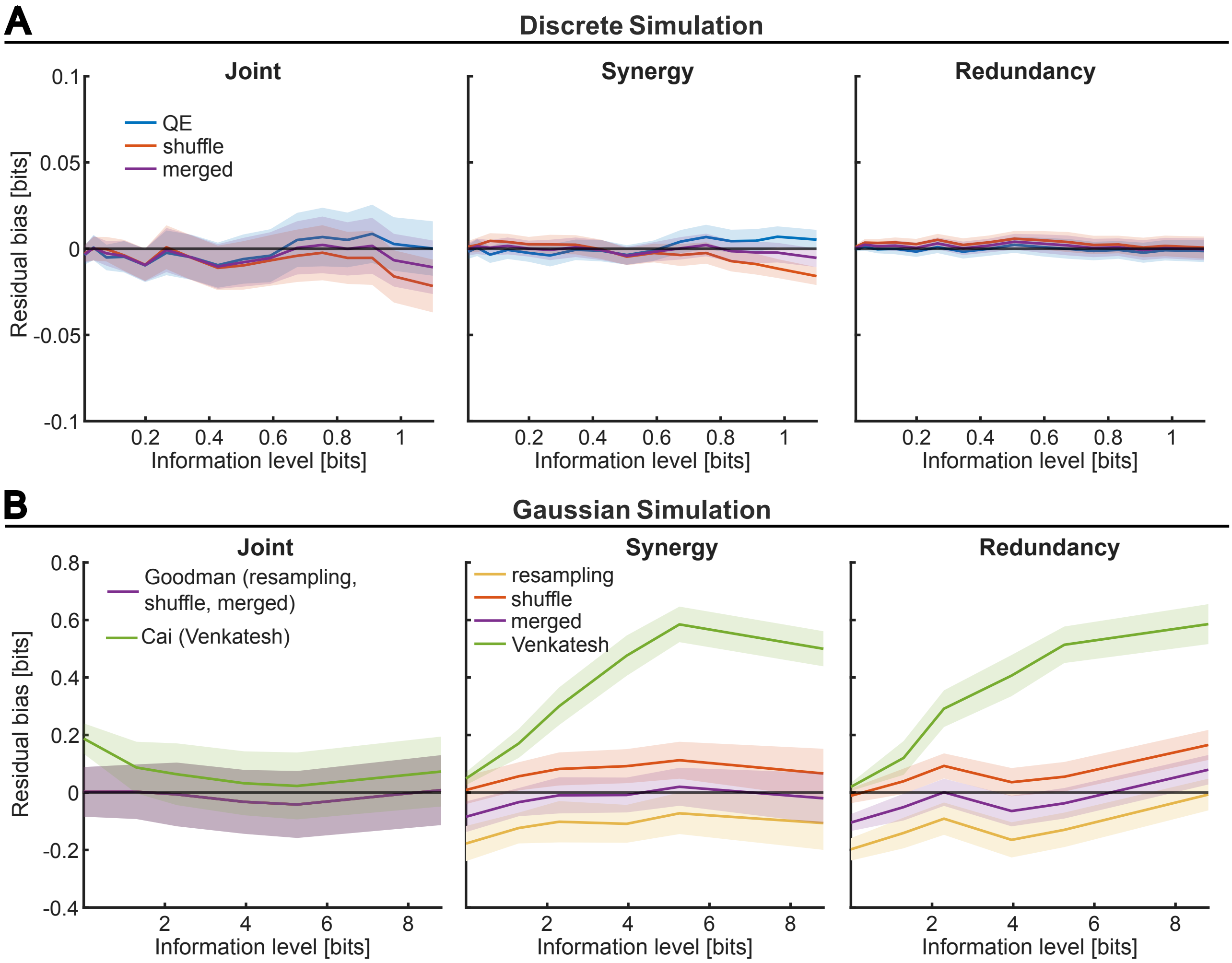

A
Discrete Simulation
Joint
Synergy
Redundancy
QE
shuffle
merged
Residual bias [bits]
0.1
0.05
0
-0.05
-0.1
0.2
0.4
0.6
0.8
1
Information level [bits]
B
Gaussian Simulation
Joint
Synergy
Redundancy
Goodman (resampling, shuffle, merged)
Cai (Venkatesh)
resampling
shuffle
merged
Venkatesh
Residual bias [bits]
0.8
0.6
0.4
0.2
0
-0.2
-0.4
2
4
6
8
Information level [bits]

Figure S9

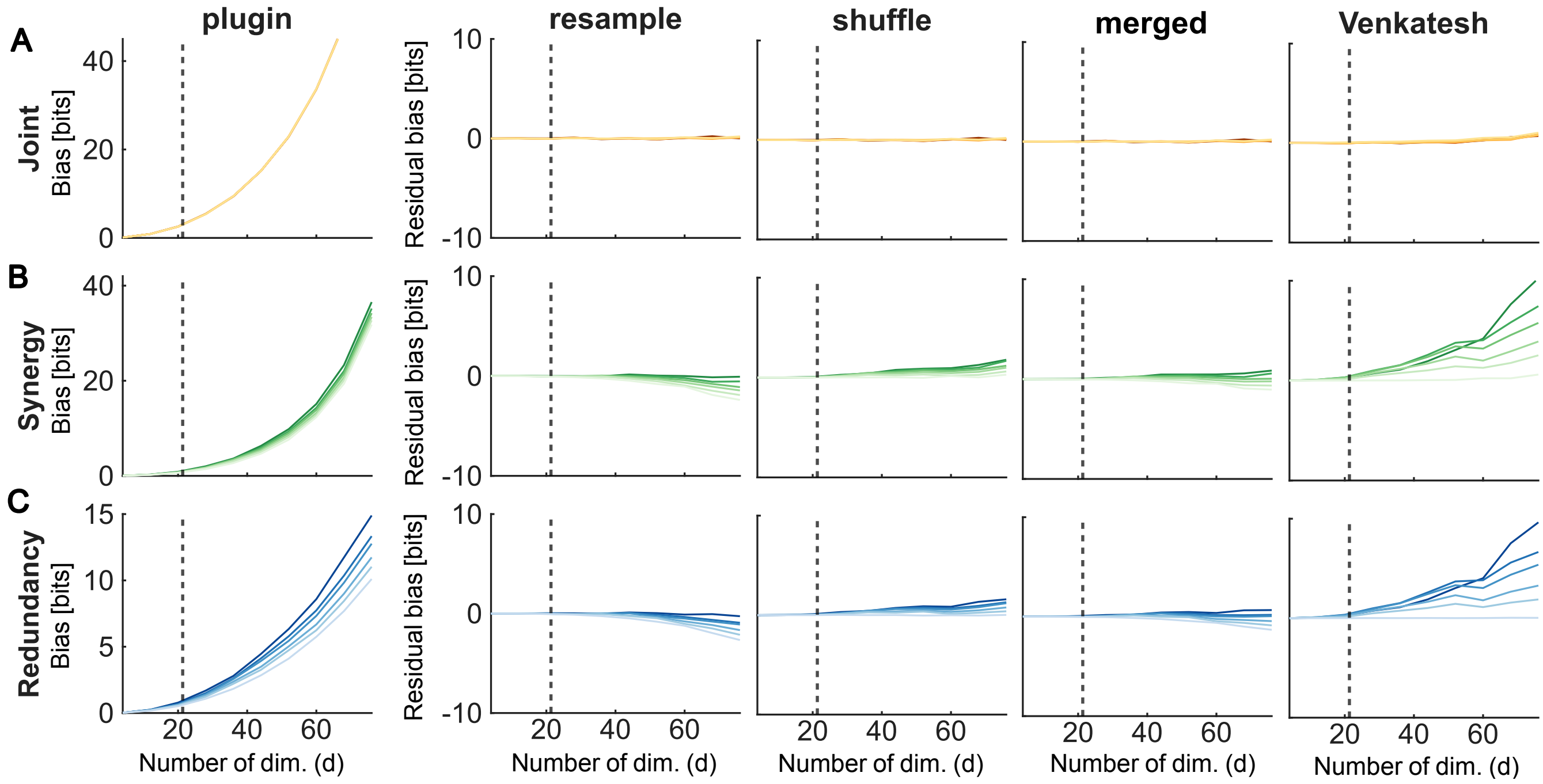

A
B
C
plugin
resample
shuffle
merged
Venkatesh
Joint
Synergy
Redundancy
Bias [bits]
Residual bias [bits]
40
20
0
10
0
-10
15
10
5
20
40
60
Number of dim. (d)

Figure S10

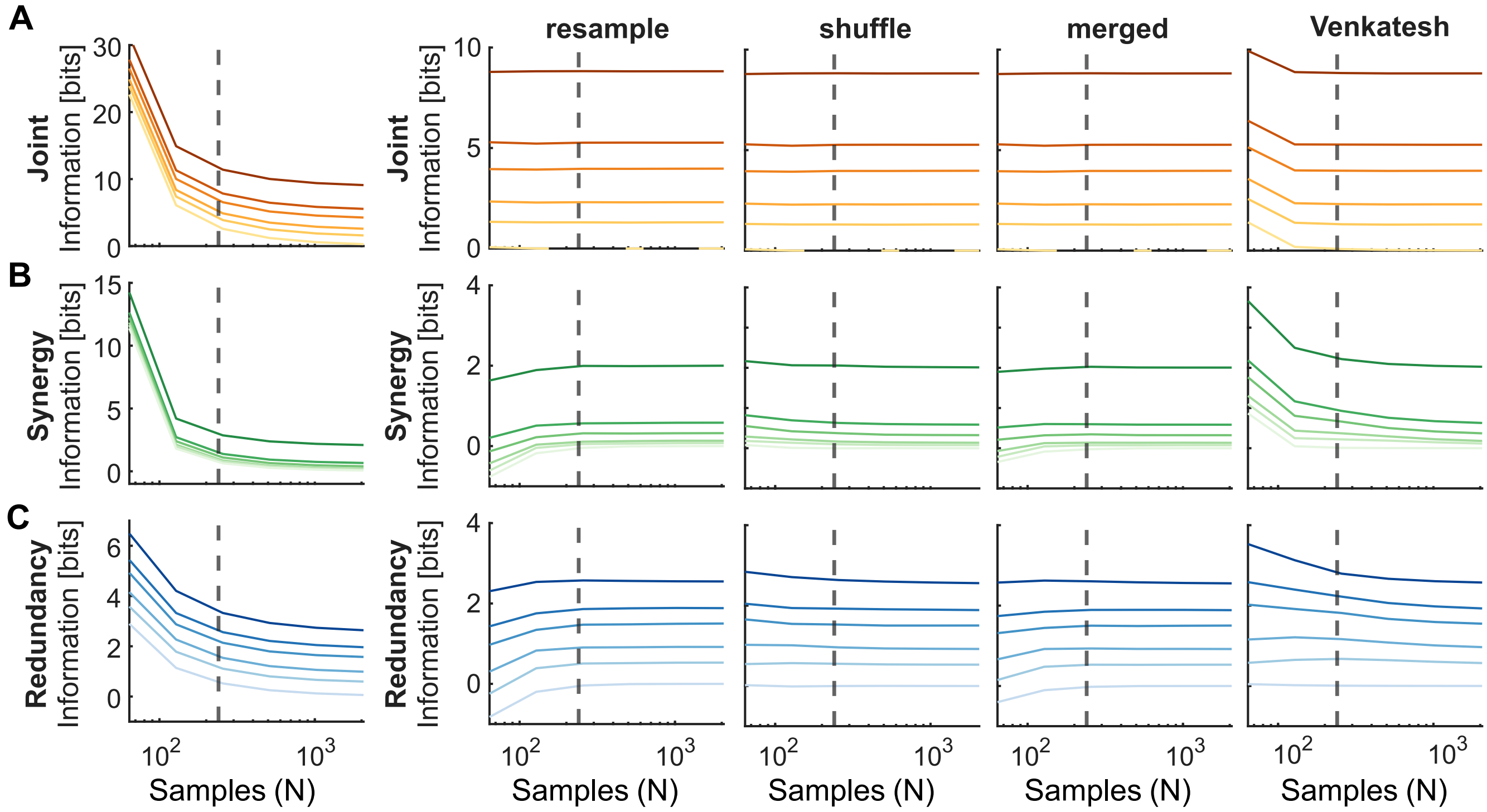

A
B
C
resample
shuffle
merged
Venkatesh
Joint
Information [bits]
Synergy
Redundancy
Samples (N)
10^2
10^3

Figure S11

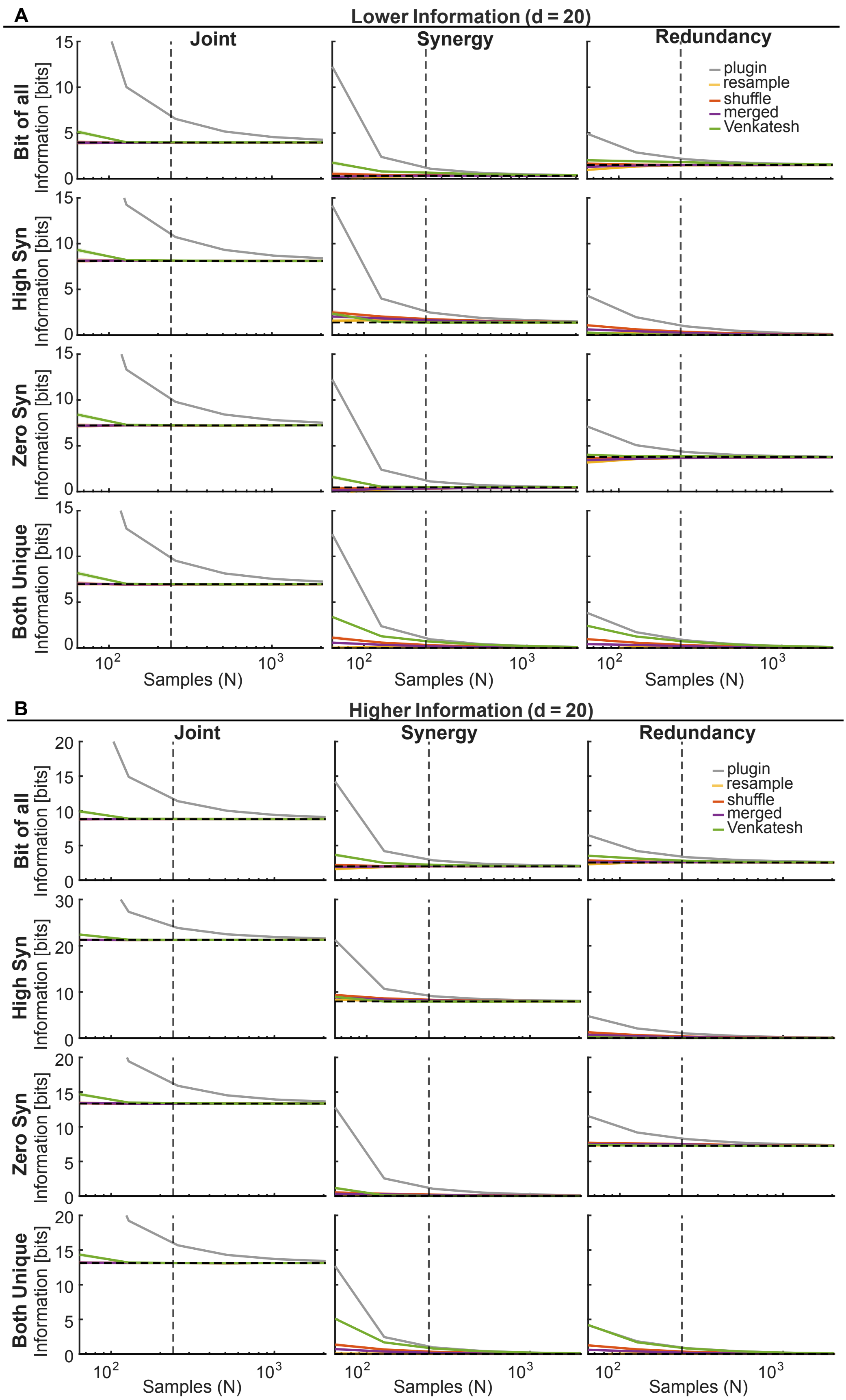

A
Lower Information (d = 20)
Joint
Synergy
Redundancy
plugin
resample
shuffle
merged
Venkatesh
Bit of all
High Syn
Zero Syn
Both Unique
Information [bits]
Samples (N)
B
Higher Information (d = 20)

Figure S12

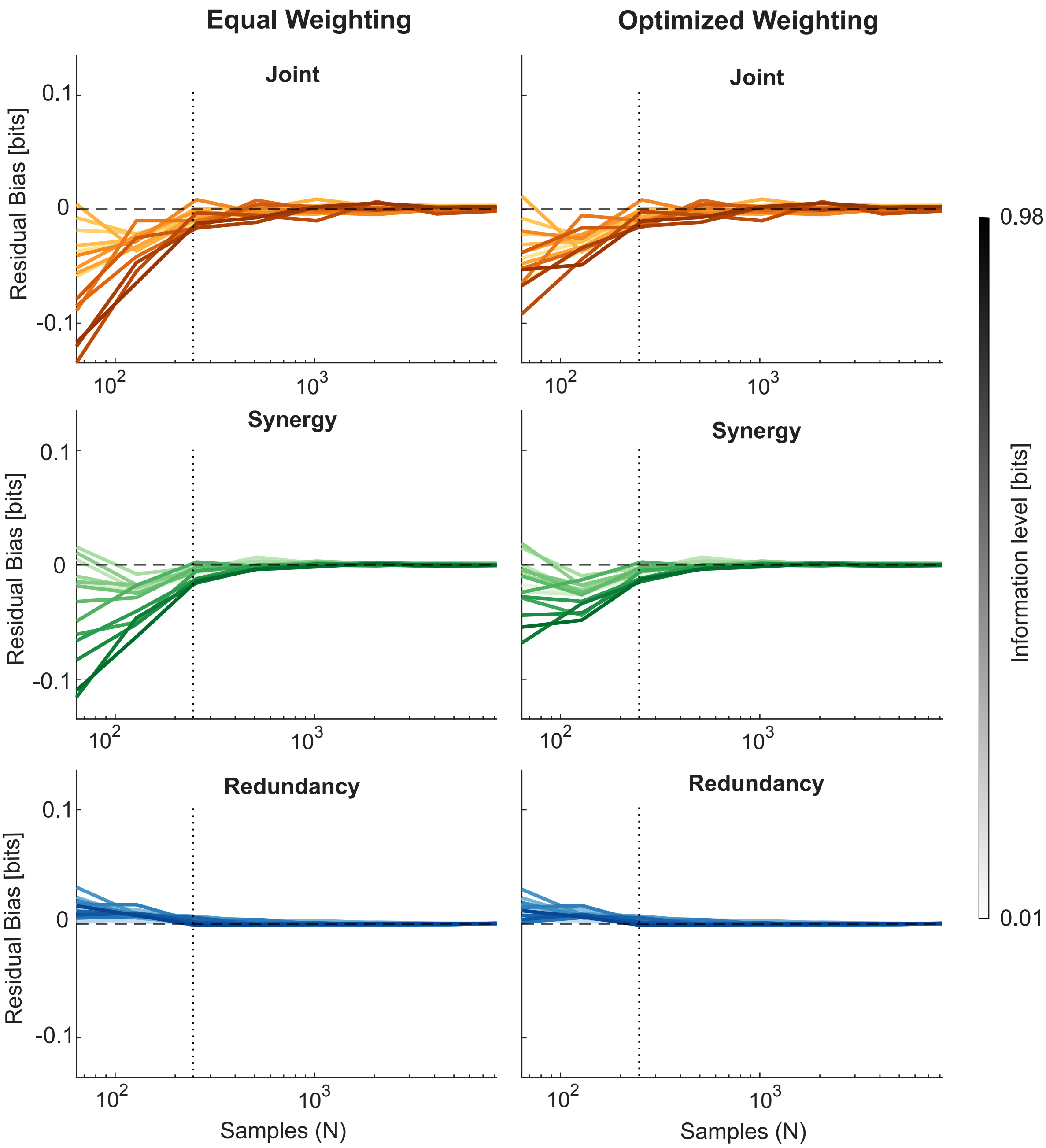
Equal Weighting
Optimized Weighting
Joint
Synergy
Redundancy
Residual Bias [bits]
0.1
0
-0.1
10^2
10^3
Samples (N)
Information level [bits]
0.98
0.01

Figure S13

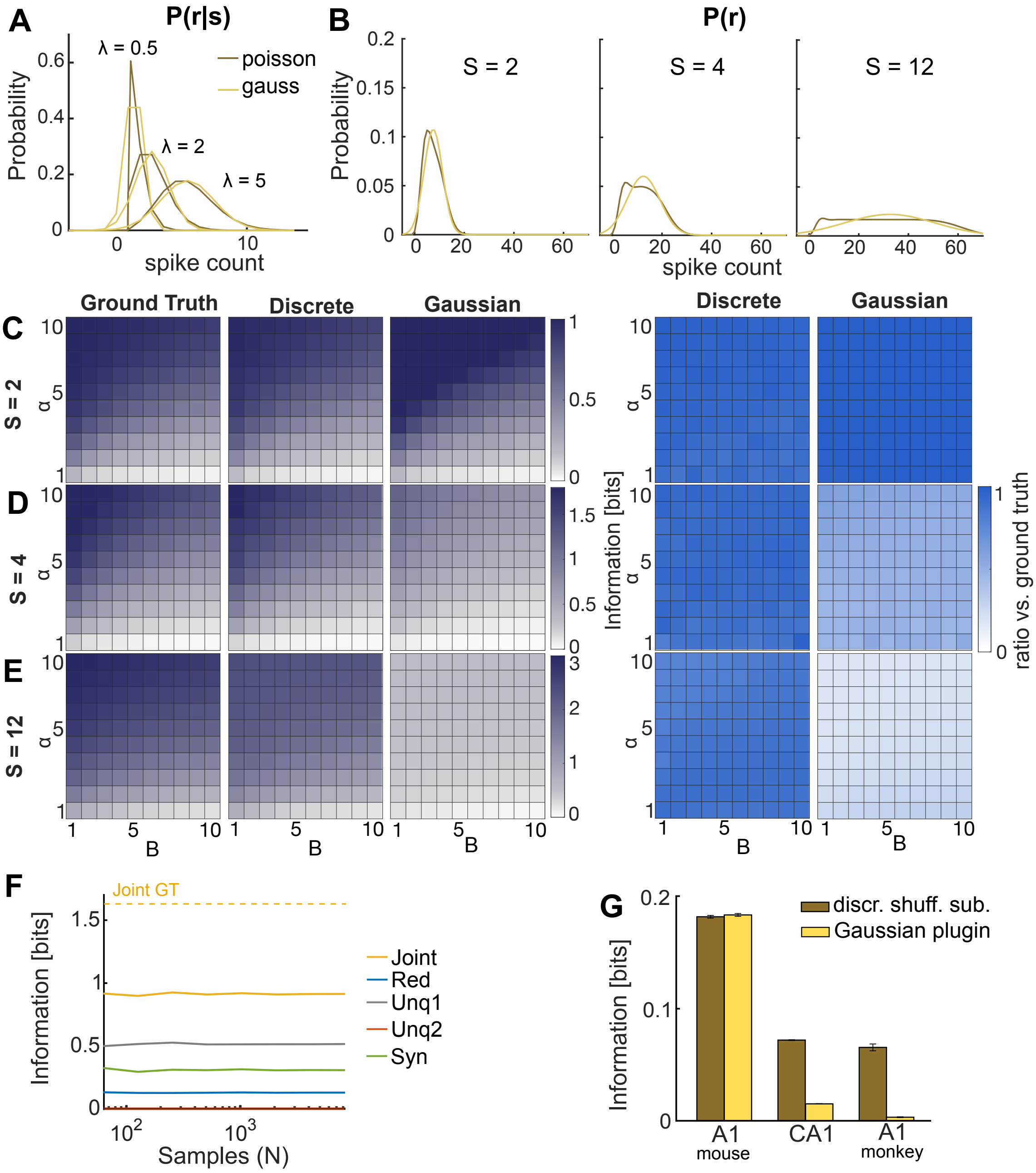

A
P(r|s)
λ = 0.5
λ = 2
λ = 5
poisson
gauss
Probability
spike count
B
P(r)
S = 2
S = 4
S = 12
Probability
spike count
C
D
E
Ground Truth
Discrete
Gaussian
S = 2
S = 4
S = 12
α
B
Information [bits]
ratio vs. ground truth
F
Joint GT
Joint
Red
Unq1
Unq2
Syn
Information [bits]
Samples (N)
G
discr. shuff. sub.
Gaussian plugin
Information [bits]
A1
mouse
CA1
A1
monkey

Figure S14

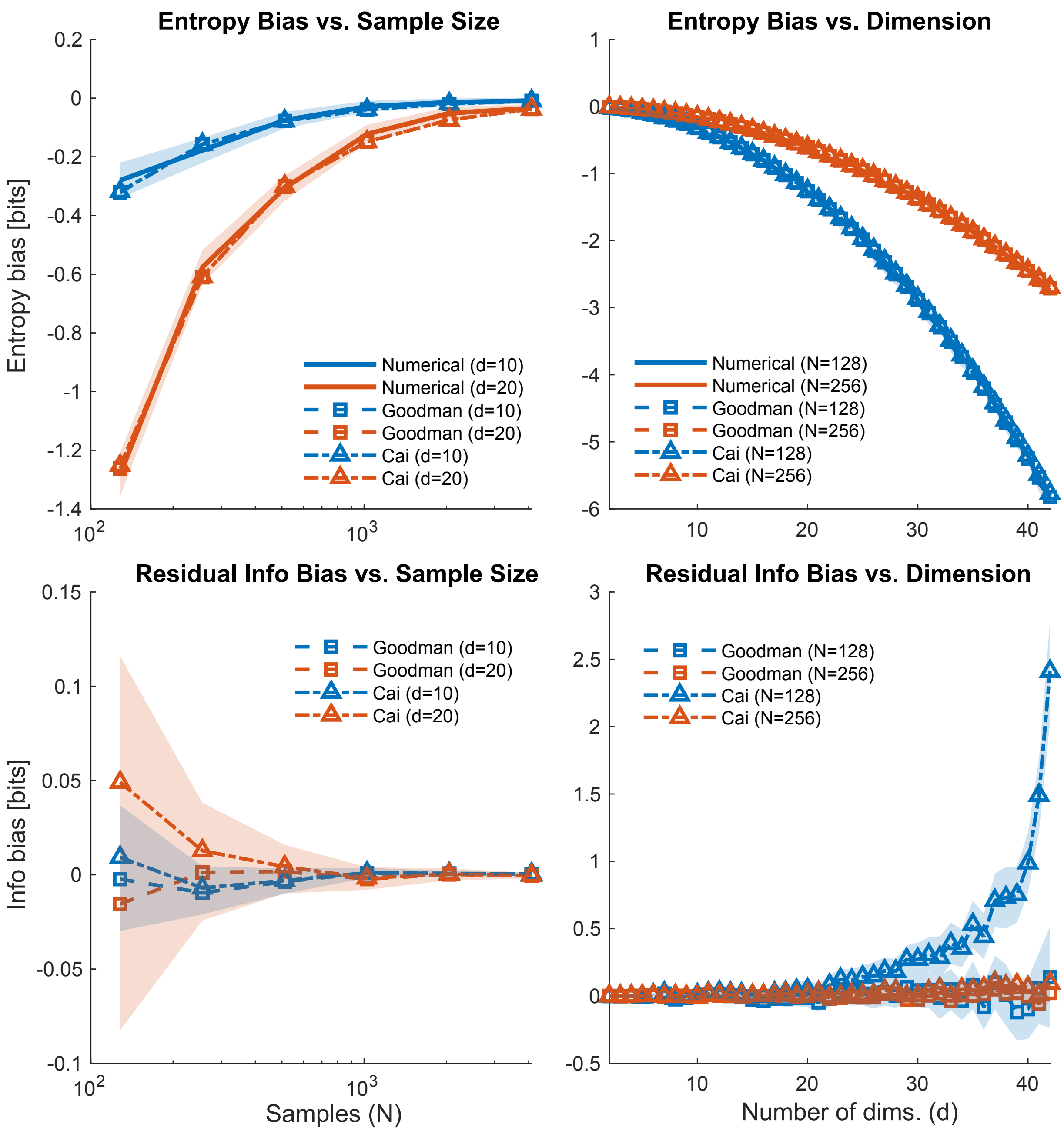

Entropy Bias vs. Sample Size
Entropy bias [bits]
Numerical (d=10)
Numerical (d=20)
Goodman (d=10)
Goodman (d=20)
Cai (d=10)
Cai (d=20)
Entropy Bias vs. Dimension
Numerical (N=128)
Numerical (N=256)
Goodman (N=128)
Goodman (N=256)
Cai (N=128)
Cai (N=256)
Residual Info Bias vs. Sample Size
Info bias [bits]
Goodman (d=10)
Goodman (d=20)
Cai (d=10)
Cai (d=20)
Samples (N)
Residual Info Bias vs. Dimension
Goodman (N=128)
Goodman (N=256)
Cai (N=128)
Cai (N=256)
Number of dims. (d)

Figure S15

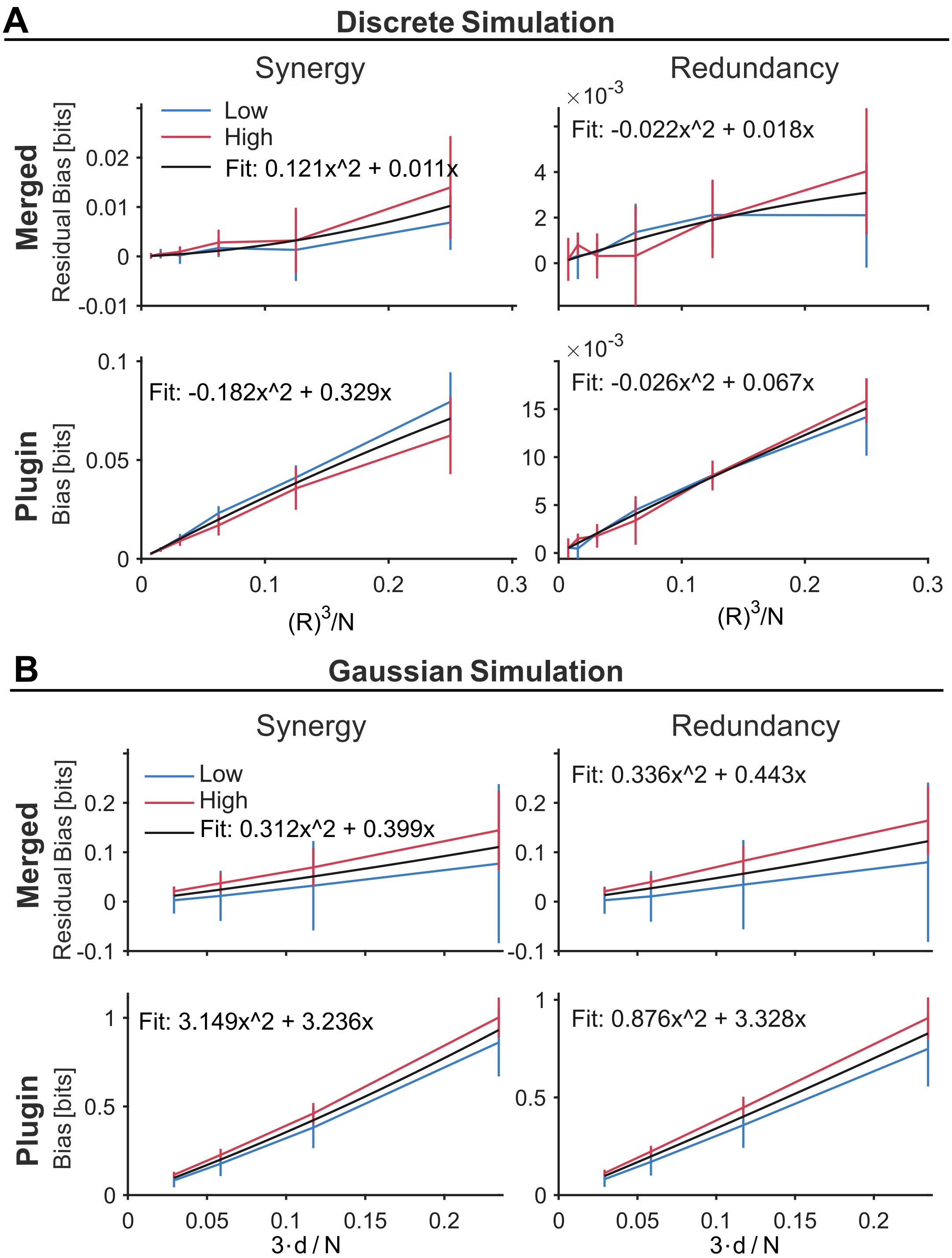

A
Discrete Simulation
Synergy
Redundancy
Merged
Residual Bias [bits]
Low
High
Fit: 0.121x^2 + 0.011x
×10^-3
Fit: -0.022x^2 + 0.018x
Plugin
Bias [bits]
Fit: -0.182x^2 + 0.329x
Fit: -0.026x^2 + 0.067x
(R)^3/N
B
Gaussian Simulation
Synergy
Redundancy
Merged
Residual Bias [bits]
Low
High
Fit: 0.312x^2 + 0.399x
Fit: 0.336x^2 + 0.443x
Plugin
Bias [bits]
Fit: 3.149x^2 + 3.236x
Fit: 0.876x^2 + 3.328x
3·d / N

Figure S16

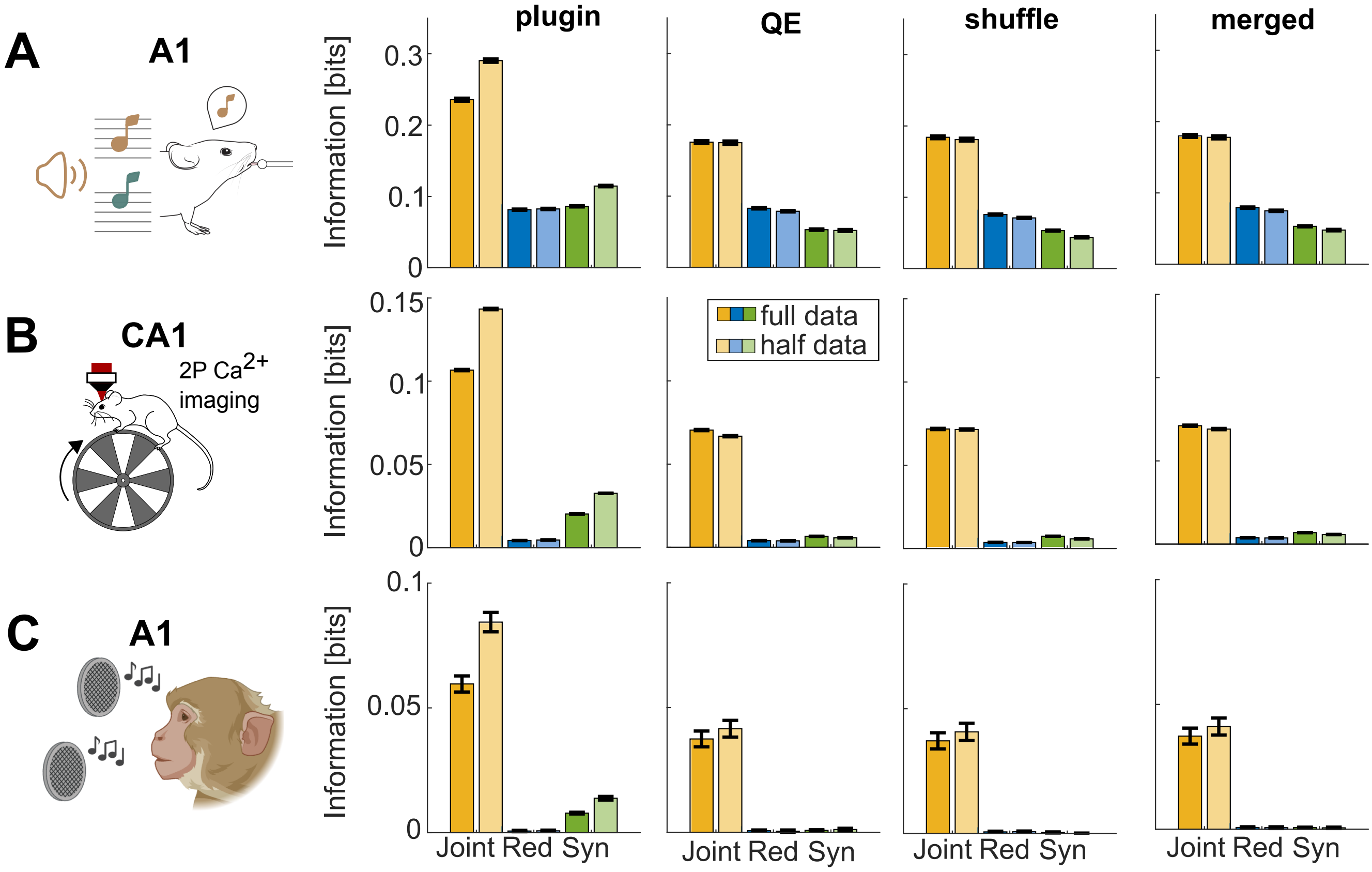
A
A1
B
CA1
2P Ca2+
imaging
C
A1
plugin
QE
shuffle
merged
Information [bits]
0.3
0.2
0.1
0
0.15
0.1
0.05
0
0.1
0.05
0
full data
half data
Joint Red Syn

Figure S17

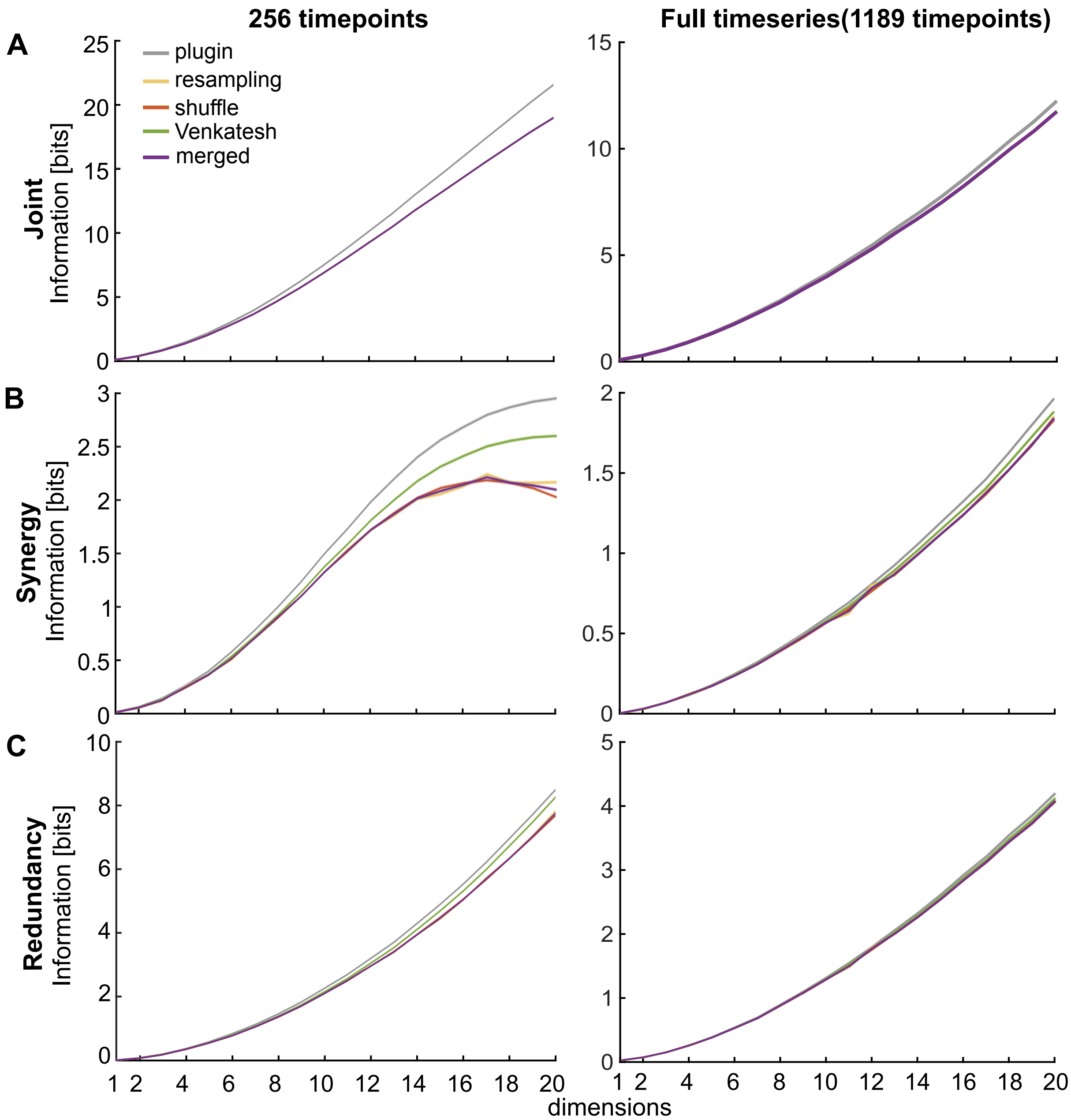
256 timepoints
Full timeseries(1189 timepoints)
A
B
C
plugin
resampling
shuffle
Venkatesh
merged
Joint
Synergy
Redundancy
Information [bits]
dimensions
1 2 4 6 8 10 12 14 16 18 20

Figure S18

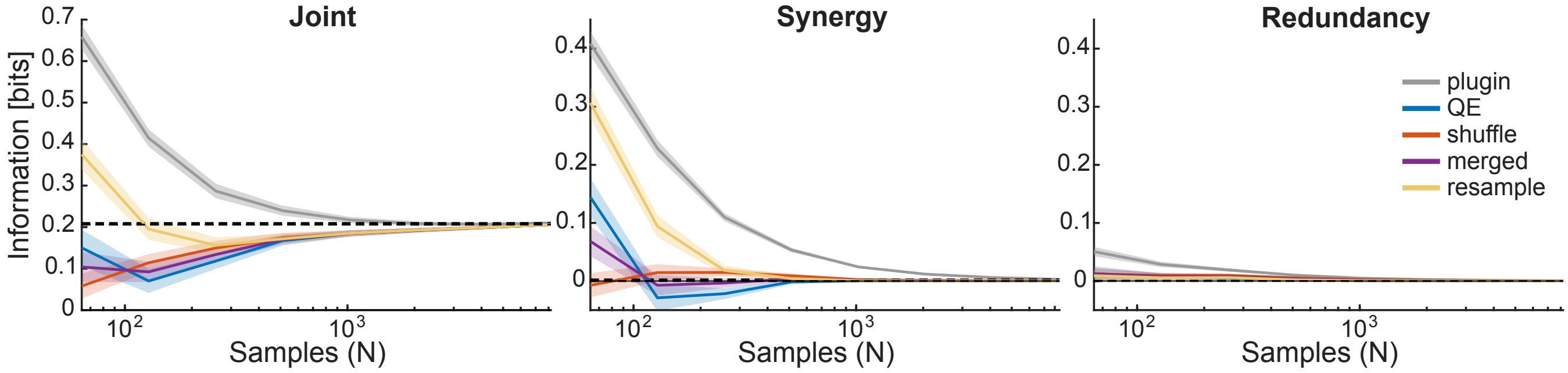

Joint
Synergy
Redundancy
Information [bits]
Samples (N)
Samples (N)
Samples (N)
plugin
QE
shuffle
merged
resample

Supplemental Figure Legends

Figure S1. Schematic of the Partial Information Decomposition with two sources. PID decomposes the joint information that the sources R_1 and R_2 carry about the target S into four components: the redundancy $Red(S : R_1; R_2)$, the two unique information terms $Unq(S : R_1 \setminus R_2)$ and $Unq(S : R_2 \setminus R_1)$, and the synergy $Syn(S : R_1; R_2)$. The colored outlines represent the four linear equations, Eq. (4–5) and (12–13) that relate the four PID components to Shannon information quantities.

Figure S2. Bias properties and bias corrections for the sum of unique information terms Plots show the residual bias (averaged bias-corrected empirical estimates from N samples minus ground-truth values) of the sum of the two unique information terms $Unq(S : R_1 \setminus R_2) + Unq(S : R_2 \setminus R_1)$ as a function of the number of samples N (left panels, on a log x-axis) and as a function of the number of parameters defining the probability distributions (right panels). A: Discrete BROJA PID simulations ($R = 4$, left; $N = 512$, right). Bias correction methods: plugin (no correction), QE, shuffle-subtraction, and merged. B: Gaussian gPID simulations ($d = 20$, left; $N = 128$, right). Bias correction methods: plugin (no correction), shuffle-subtraction, resampling, merged, and Venkatesh. Results in each panel are plotted as mean $\pm$ 3 SEM over $n = 100$ simulations. Dashed vertical lines: boundary of the sufficient sampling regime. Discrete and Gaussian simulations were performed under the bit-of-all scenario (see Methods and Table S2). For more details see Table S1.

Figure S3. Bias of the plugin PID values and residual bias of the bias-corrected PID values as a function of the number of parameters defining the probability distributions. Biases of plugin estimates and residual biases of bias-corrected estimates are computed as the difference between the averaged plugin (no correction) or bias-corrected information empirically measured from N samples and the corresponding ground-truth values. A–C: results for discrete BROJA PID. Bias correction methods: plugin (no correction), shuffle-subtraction, QE, and merged. D–F: results for Gaussian gPID. Bias correction methods: plugin (no correction), shuffle-subtraction, resample, merged, and Venkatesh. Left to right: joint information, synergy, and redundancy. Each row corresponds to a different number of simulated samples (indicated on the far left). Results in each panel show mean $\pm$ 3 SEM over $n = 100$ simulations. Dashed vertical lines: boundary of the sufficient sampling regime. In both cases, simulations were performed under the bit-of-all scenario (see Methods and Table S2). For more details see Table S1.

Figure S4. Dependence of the bias of the discrete BROJA PID on the number of samples N used to compute it. A: Joint information. B: Synergy. C: Redundancy. Each column corresponds to a different bias correction: plugin (no correction), QE, shuffle-subtraction, merged, and resample. Individual lines correspond to different values of the level of joint information, controlled by the simulation parameter α regulating the stimulus-response gain, with information level indicated by line color (lighter to darker colors indicate lower to higher information; see Methods). The x-axis is plotted on a logarithmic scale. We used $R = 4$ discretization bins for each neuron and $S = 4$ different stimulus values. Results in each panel are plotted as mean $\pm$ 3 SEM over $n = 100$ simulations. Dashed vertical lines: boundary of the sufficient sampling regime. Simulations were performed under the bit-of-all scenario (see Methods and Table S2). For more details see Table S1.

Figure S5. Bias correction performance for the discrete BROJA PID. Values of discrete joint information (left), synergy (middle), and redundancy (right) in four different simulated scenarios ('bit-of-all', 'uncorrelated', 'high redundancy', and 'high synergy'; see Methods and Table S2). Plots show the information values as a function of the number of samples N used to compute

them. The x-axis is plotted on a logarithmic scale. Bias correction methods: plugin (no correction), shuffle-subtraction, QE, merged, and resample. A: Results for the case of lower joint information ($\alpha = 7$). B: Results for the case of higher joint information ($\alpha = 10$). We used $R = 4$ discretization bins for each simulated neuron and $S = 4$ stimuli. Results in each panel are plotted as mean $\pm$ 3 SEM over $n = 100$ simulations. Dashed horizontal lines: ground truth information values computed from the true distributions. Dashed vertical lines: boundary of the sufficient sampling regime. For details see Methods and Tables S1 and S2.

Figure S6. Bias correction performance for the discrete I_{min}. Values of discrete joint information (left), synergy (middle), and redundancy (right) in four different simulated scenarios ('bit-of-all', 'uncorrelated', 'high redundancy', and 'high synergy'; see Methods and Table S2). Plots show the information values as a function of the number of samples N used to compute them. The x-axis is plotted on a logarithmic scale. Bias correction methods: plugin (no correction), shuffle-subtraction, QE, merged, and resample. A: Results for the case of lower joint information ($\alpha = 7$). B: Results for the case of higher joint information ($\alpha = 10$). We used $R = 4$ discretization bins for each simulated neuron and $S = 4$ stimuli. Results in each panel are plotted as mean $\pm$ 3 SEM over $n = 100$ simulations. Dashed horizontal lines: ground truth information values computed from the true distributions. Dashed vertical lines: boundary of the sufficient sampling regime. For details see Methods and Tables S1 and S2.

Figure S7. Bias correction performance for the discrete I_{MMI}. Values of discrete joint information (left), synergy (middle), and redundancy (right) in four different simulated scenarios ('bit-of-all', 'uncorrelated', 'high redundancy', and 'high synergy'; see Methods and Table S2). Plots show the information values as a function of the number of simulated trials (i.e. samples) N used to compute them. The x-axis is plotted on a logarithmic scale. Bias correction methods: plugin (no correction), shuffle-subtraction, QE, merged, and resample. A: Results for the case of lower joint information ($\alpha = 7$). B: Results for the case of higher joint information ($\alpha = 10$). We used $R = 4$ discretization bins for each simulated neuron and $S = 4$ stimuli. Results in each panel are plotted as mean $\pm$ 3 SEM over $n = 100$ simulations. Dashed horizontal lines: ground truth information values computed from the true distributions. Dashed vertical lines: boundary of the sufficient sampling regime. For details see Methods and Tables S1 and S2.

Figure S8. Residual bias across information levels. Plots show the residual bias of joint information (left), synergy (middle), and redundancy (right) as a function of the joint information level. Residual biases are computed as the difference between the averaged bias-corrected information empirically measured from N samples and the corresponding ground-truth values. A: Discrete BROJA PID simulations. We vary the level of joint information (plotted on the x-axis) by changing the value of the simulation parameter α regulating the stimulus-response gain (number of samples $N = 512$ and number of bins $R = S = 4$). Bias corrections used for information quantities: QE, shuffle-subtraction, and merged. B: Gaussian gPID simulations. We vary the level of joint information (plotted on the x-axis) by changing the simulation parameter α scaling the diagonal of the covariance matrix ($N = 256$ and number of sources and target dimensions $d = 20$). Bias corrections used for synergy and redundancy: resampling, shuffle-subtraction, merged, and Venkatesh. For joint information we used the Goodman correction in the resampling, shuffle-subtraction, and merged methods as it is an exact correction, and for the Venkatesh correction we used the Cai et al. correction to replicate what was done in Venkatesh et al.[S1]. We plot mean $\pm$ 3 SEM over $n = 100$ simulations. For both discrete and Gaussian PID, simulations were performed under the bit-of-all scenario (see Methods and Table S2). For more details see Table S1.

Figure S9. Bias and performance of bias corrections for the Gaussian gPID as a function

of the number of dimensions d. Biases of plugin (no correction) estimates and residual biases of bias-corrected estimates are computed as difference between the averaged plugin or bias-corrected information empirically measured from N samples and the corresponding ground-truth values. A: Joint information. B: Synergy. C: Redundancy. The far left column plots the bias of the plugin values. Each of the other columns plots the residual bias after application of the bias correction method listed in the panel title. Individual lines correspond to different values of the level of joint information, controlled by the simulation parameter α scaling the diagonal of the covariance matrix, with information level indicated by line color (lighter to darker colors indicate lower to higher information; see Methods; number of samples $N = 256$). Results in each panel are plotted as mean over $n = 100$ simulations. Dashed vertical lines: boundary of the sufficient sampling regime. Simulations were performed under the bit-of-all scenario (see Methods). For more details see Table S1.

Figure S10. Bias and performance of bias corrections for the Gaussian gPID as a function of the number of samples N used to compute them. The x-axis is plotted on a logarithmic scale. A: Joint information. B: Synergy. C: Redundancy. The far left column plots the plugin (no correction) information values. Each of the other columns plots the information values after application of the bias correction method listed in the panel title. Individual lines correspond to different values of the joint information, controlled by the simulation parameter α scaling the diagonal of the covariance matrix, with information level indicated by line color (lighter to darker colors indicate lower to higher information; see Methods). We set the dimensionality of the target and of the sources to $d = 20$. Results in each panel are plotted as mean over $n = 100$ simulations. Dashed vertical lines: boundary of the sufficient sampling regime. Simulations were performed under the bit-of-all scenario (see Methods). For more details see Table S1.

Figure S11. Bias corrections performance for gPID. Values of Gaussian joint information (left), synergy (middle), and redundancy (right) in four different simulated scenarios ('bit-of-all', 'high synergy', 'zero synergy', and 'both unique'; see Methods). Plots show the information values as a function of the number of samples N used to compute them. The x-axis is plotted on a logarithmic scale. Bias correction methods: plugin (no correction), resample, shuffle-subtraction, merged, and Venkatesh. A: Results for the case of lower joint information ($\alpha = 1.5$). B: Results for the case of higher joint information ($\alpha = 1$). We set the number of dimensions of the target and the sources to $d = 20$. Results in each panel are plotted as mean $\pm$ 3 SEM over $n = 100$ simulations. Dashed horizontal lines: ground truth information values computed from the true distributions. Dashed vertical lines: boundary of the sufficient sampling regime. For details see Methods and Table S1.

Figure S12. Comparison of the residual bias of the discrete merged correction using optimized and equal weighting. The residual bias (difference between the averaged values estimated from N samples and the corresponding ground-truth values) of discrete joint information (top row), synergy (middle row), and redundancy (bottom row) as a function of the number of samples N used to compute them. The x-axis is plotted on a logarithmic scale. Columns compare the merged estimator obtained using equal weighting of QE and shuffle-subtracted estimates (left) with the optimized weighted merged estimator (right), where weights were optimized depending on the joint information level. Individual lines correspond to different joint information levels, controlled by the simulation parameter α regulating the stimulus-response gain, with information level indicated by line color (lighter to darker colors indicate lower to higher information; see Methods). Simulations were performed using the BROJA PID under the bit-of-all discrete scenario (see Table S2) with $R = 4$ discretization bins per neuron and $S = 4$ stimulus values. Results in each panel are plotted as mean $\pm$ 3 SEM over $n = 100$ simulations. Dashed vertical lines: boundary of the sufficient sampling regime.

Figure S13. Using a Gaussian PID on discrete neural data. A: Example of stimulus-conditional probability distributions $p(r|s)$ of spike counts of one Poisson neuron whose spike counts follow a Poisson distribution with a given mean λ in response to different stimuli. The brown line denotes the Poisson distribution of the spike counts and the yellow line denotes a Gaussian with the same mean. B: Same as in panel A, but plotting the marginal (stimulus-unconditional) distribution $p(r)$ of the spike counts obtained assuming S uniformly distributed discrete stimuli. C: Discrete and Gaussian information estimates vs ground truth values of uncorrelated Poisson neuron pairs responding to $S = 2$ discrete stimuli (Eq. (26), $\beta = \gamma = 0$). Parameters α (information level) and B (background firing) were varied. GT indicates ground-truth values computed from the exact Poisson probabilities. Discretized and Gaussian (Eq. (21)) estimates are computed numerically from 10000 simulated samples per stimulus. Left: information values for each parameter combination. Right: for each parameter combination, ratios between estimated (discrete or Gaussian) and GT value. D and E: same as C, but neurons respond to $S = 4$ and $S = 12$ discrete stimuli, respectively. F: Bias corrected PID terms computed with the gPID from a discrete simulation ($\alpha = 10$, $B = 5$, $\beta = \gamma = 0$, Eq. (26)). G: Estimated joint information from the three real neural spiking datasets shown in Fig. 6A–C using the discrete shuffle-subtraction bias correction and the Gaussian plugin (no correction) estimator (mean $\pm$ 3 SEM across all neuron pairs). For more details see Table S1.

Figure S14. Comparison of analytical expressions of the bias for the Gaussian entropy, and evaluations of the residual mutual information bias. A: Bias of the entropy of a Gaussian multivariate distribution as a function of the number of samples N (x-axis on a logarithmic scale). Results computed with dimension $d = 10$ and $d = 20$ are plotted with blue and red colors, respectively. B: Bias of the entropy of a Gaussian multivariate distribution as a function of the dimension d computed with $N = 128$ (blue) and $N = 256$ (red) samples, respectively. The Gaussian process used in simulations for panels A–B had a diagonal covariance matrix, with zero mean and unit variance in each of the d dimensions. C–D: Difference between the numerically measured bias and the analytical bias computed with analytical expressions either from Cai et al. or from Goodman, plotted as a function of the number of samples N (C; with x-axis on a logarithmic scale) or the dimension d (D). The Gaussian process used in simulations for panels C–D was made of one Gaussian source and one Gaussian target of the specified dimension d with a diagonal covariance matrix and thus zero mutual information between the target and the source. Results in each panel are plotted as mean $\pm$ 3 SEM over $n = 100$ simulations. Solid line: numerical evaluation from simulated data. Squares: Goodman analytical formula. Triangles: Cai et al. analytical formula. For more details see Table S1.

Figure S15. Tolerance analysis performed on the merged-corrected residual synergy and redundancy bias and on the plugin synergy and redundancy bias. We fitted the absolute value of the residual merged bias or the plugin (no correction) bias values obtained from all data points within the sufficient sampling regime from simulations in Figs. S3A, S4, and S5 to quadratic functions of a quantity that captures the scaling of the leading term of the synergy bias according to theoretical considerations. This quantity (plotted on the x-axis of each panel) is R^3/N for the discrete BROJA PID (panel A) and $3d/N$ for the Gaussian gPID (panel B). In each panel we plot the averages $\pm$ SEM over all simulation cases ('bit-of-all', 'uncorrelated', 'high redundancy', 'high synergy') from Figs. S3A, S4, and S5 with low (blue) and high (red) joint information, respectively.

Figure S16. Comparison of PID bias corrections on each of the three full real neural datasets from Fig. 6A–C and on the same datasets each recomputed with half the trials. Each panel shows mean $\pm$ 3 SEM across all available simultaneously recorded neural pairs. A: mouse auditory cortex (dataset 1), $n = 6209$ pairs. B: mouse hippocampus (dataset 2),

$n = 36158$ pairs. C: monkey auditory cortex (dataset 3), $n = 83$ pairs. Left to right: schematic of each experiment; PID results with plugin (no correction), QE, shuffle-subtraction, and merged bias correction methods, respectively. Darker colors indicate quantities computed from the full datasets (all available trials); lighter colors indicate quantities recomputed with half the trials. *Image credits:* Panel A: mouse sketch adapted from doi.org/10.5281/zenodo.3925985, speaker icon from svgrepo.com, and music note icon from svgrepo.com; all modified and licensed under CC BY 4.0. Panel B: created by the authors. Panel C: Created in BioRender.

Figure S17. Further analyses of the human resting-state fMRI dataset (dataset 4). A: the joint information between the activity of a target set of ROIs and the activity of two source sets of ROIs shifted by one imaging time frame into the past. B: same as in panel A, for synergy. C: same as in panel A, for redundancy. We plot results as a function of dimension d (the number of ROIs within each group) of each multivariate source and target. We report Gaussian gPID estimates not bias-corrected (plugin) or bias-corrected with: resampling, shuffle-subtraction, merged, and Venkatesh. Left: results using 256 time-points (imaging volumes), corresponding to a typical size of an fMRI clinical resting-state dataset. Right: results using the entire dataset with all 1189 time-points available. Results in each panel are plotted as mean $\pm$ 3 SEM over $n = 100$ subjects.

Figure S18. Evaluation of the bias and the performance of bias correction methods when computing discrete BROJA PID of two 10-dimensional sources with an SVM dimensionality reduction. Each source is a different population of 10 uncorrelated Poisson neurons responding to $S = 4$ uniformly distributed stimuli ($\alpha = 1$, $\beta = \gamma = 0$; Eq. (26)). The 10-dimensional responses of each source are compressed to $R = 4$ discrete responses by using a multi-class linear SVM (2-fold cross-validation) to predict the value of the stimulus feature in each trial from the activity of each population. We plot joint information (left), synergy (middle), and redundancy (right) as a function of the number N of simulated trials. The x-axis is plotted on a logarithmic scale. Results in each panel are plotted as mean $\pm$ 3 SEM over $n = 100$ simulations. For more details see Table S1.

Figure	Support size	parameter
1A,B	R=4	N=100,1000
1C,D	d=5	N=20,50
2A	R=4 (left and right), R=2-9 (middle)	N=64-8192 (left),<br>N=512 (middle), N=64 (right)
2B	d=20 (left and right), R=1-42 (middle)	N=64-2048 (left),<br>N=128 (middle and right), $\alpha = 1$
3 top	R=4 (A and B)	N = 64-8192 (A and B),<br>N= 64-2048 (C)
3 bottom	R=2-9 (A and B), d=1-42 (C)	N=128
4A	R=4	N=64-8192
4B	d=20	N=64-2048
5A	R=2-9	N=512
5B	d=1-84	N=128
6,S16, S17	R = 3, S = 2 (A1 mouse),<br>R = 2, S = 12 (CA1),<br>R = 2, S = 399 (A1 monkey),<br>d = 20 (fMRI)	N = 140 (A1 mouse),<br>N = 870 (CA1),<br>N = 108 (A1 monkey),<br>N = 256 (fMRI)
7A	R=2-6	N=2ˆ10-2ˆ14
7B	d=1-15	N=2ˆ6-2ˆ10
8 top	R=3	N=[519-864]
8 bottom	$d_{sources} = [26, 47, 25]$, $d_{target} = 1$	N=[519-864]
S2A	R=4 (left), R=2-9 (right)	N=64-8192 (left),<br>N=512 (right)
S2B	d=20 (left), d=4-42 (right)	N=64-2048 (left),<br>N=128 (right), $\alpha = 1$
S3A, B, C	R = 2–9	N = 256 trials (Fig. S3A),<br>N = 512 trials (Fig. S3B),<br>N = 1024 trials (Fig. S3C)
S3D, E, F	d = 4-84	$\alpha = 1.5$, N = 64 trials (Fig. S3D),<br>N = 128 trials (Fig. S3E),<br>N = 256 trials (Fig. S3F)
S4,S5,S6,S7,S12, S15	R=4	N = 64-8192
S8A	R=4	N=512
S8B	d=20	N=256
S9	d=1-84	N=256
S10, S11	d=20	N=64-2048
S13	R = 4, S = 2 (Fig. S13C),<br>R = 4, S = 4 (Fig. S13D,F),<br>R = 4, S = 12 (Fig. S13E)	N = 20000 (Fig. S13C),<br>N = 40000 (Fig. S13D,F),<br>N = 120000 (Fig. S13E)<br>α = 1-10,<br>B = 1-10, β = 0, γ = 0
S14A,C	d=10,20	N=64-2048
S14B,D	d=1-42	N=128, 256
S18	population size = 10	N = 64–8192,<br>α = 1,<br>B = 10, β = 0, γ = 0

Table S1: **Summary of support sizes and simulation parameters used across all figures, including main text and supplementary figures**. Support size refers to the number of bins (discrete case) or dimensions (Gaussian case). Parameters include the number of trials and, for simulated data, the simulation parameters.

Scenario	Parameters
bit-of-all (discrete)	$B = 5$; $\beta = 0.25$; $\gamma = 17.5$; $\alpha = 0$ (zero info), $\alpha = 7$ (lower info), 10 (higher info)
Uncorrelated (discrete)	$B = 5$; $\beta = 0$; $\gamma = 0$; $\alpha = 0$ (zero info), $\alpha = 7$ (lower info), 10 (higher info)
High redundancy (discrete)	$B = 5$; $\beta = 0.4$; $\gamma = 2$; $\alpha = 0$ (zero info), $\alpha = 7$ (lower info), 10 (higher info)
High synergy (discrete)	$B = 5$; $\beta = 0.1$; $\gamma = 20$; $\alpha = 0$ (zero info), $\alpha = 7$ (lower info), 10 (higher info)
Gaussian Simulations	$\alpha = 10000$ (zero info), $\alpha = 1.5$ (lower info), 1 (higher info)

Table S2: **Overview of simulation parameters for simulated scenarios of discrete and Gaussian simulations**. The values reported in the table are used for Figures 2A, 2B, 3A, 3B, 4A, 5A, S3A-C, S5, S6, S7. For analyses of bias across information levels, we vary α from 1 to 15 in discrete simulations (see Figs. 2A, S4, S8, S12) and use values $\alpha =$1, 1.1, 1.2, 1.5, 2 and 10000 in Gaussian simulations (see Figs. 2B, S8 and S9). Gaussian simulation scenarios were implemented exactly as described in Sec. C.1 (p. 22) of[S1].

Supplementary Notes

Supplementary Note 1: Other discrete PID measures

$\boldsymbol{I_{min}}$**.** In the work introducing PID[S2], Williams and Beer proposed a measure of redundant information about S, called I_{min}, that for two source variables R_1 and R_2 is defined as follows:

$$Red(S : R_1; R_2) = \sum_{s \in S} p(s) \min_{R_i \in \{R_1, R_2\}} I(S = s; R_i) \tag{S1}$$

where $I(S = s; R_i)$ is the *specific information* that source R_i carries about a specific value of the target variable $s \in S$, and is defined as:

$$I(S = s; R_i) = \sum_{r_i \in R_i} p(r_i|s) \Big[\log \frac{p(s|r_i)}{p(s)}\Big] \tag{S2}$$

Intuitively, the redundant information computed as in Eq. (S1) quantifies redundancy as the similarity between R_1 and R_2 in discriminating individual values of the target S.

$\boldsymbol{I_{MMI}}$**.** Minimum mutual information I_{MMI} was introduced in[S3]. This measure is important since both I_{min} and BROJA measures reduce to I_{MMI} for Gaussian systems with an univariate target. This is possibly the simplest PID measure, defining the redundant information that R_1 and R_2 carry about S as follows:

$$Red(S : R_1; R_2) = \min\{I(S; R_1), I(S; R_2)\} \tag{S3}$$

I_{MMI} quantifies redundant information by directly comparing the amount of information encoded individually by each source, and results in at least one component of unique information always being null. (note that in this paper we also compute I_{MMI} with three source variables, and that the definition of MMI redundancy with three sources is provided in Methods).

Supplementary Note 2: Key properties of PID components for two source variables

In this subsection, we review useful key properties of PID measures. In their paper introducing PID for the first time, Williams and Beer[S2] proposed a set of properties that a measure of redundancy $Red(S : R_1, R_2)$ should satisfy. In the following years, other authors introduced new properties which they thought to be important for measures of PID components[S4–6].

Three particularly important properties of PID measures with two source variables are: non-negativity, symmetry and additivity[S1].

- Non-negativity: each PID component should be non-negative, i.e. $Red(S : R_1; R_2) \geq 0$, $Unq(S : R_1 \setminus R_2) \geq 0$, $Unq(S : R_2 \setminus R_1) \geq 0$ and $Syn(S : R_1; R_2) \geq 0$. This property is important since it allows interpreting PID components as fractions of the joint mutual information about the target S encoded by the source variables (i.e., the average reduction of the entropy of S obtained when measuring the two sources simultaneously). All three discrete BROJA, I_{min} and I_{MMI} measures satisfy non-negativity[S2,3,7].

- Symmetry: redundancy and synergy are symmetric under permutation of source variables, i.e.
$Red(S : R_1; R_2) = Red(S : R_2; R_1)$ and $Syn(S : R_1; R_2) = Syn(S : R_2; R_1)$. This property is important since also Shannon information quantities which can be decomposed in terms of redundancy and synergy (including the joint information and the co-information) are symmetric under reordering of R_1 and R_2. All three discrete BROJA, I_{min} and I_{MMI} measures satisfy symmetry[S2,3,7].

- Additivity: given two independent subsystems of random variables (S_1, X_1, Y_1) and (S_2, X_2, Y_2), then

$$\begin{aligned} Red((S_1, S_2) : (X_1, X_2); (Y_1, Y_2)) &= Red(S_1 : X_1; Y_1) + Red(S_2 : X_2; Y_2) \\ Syn((S_1, S_2) : (X_1, X_2); (Y_1, Y_2)) &= Syn(S_1 : X_1; Y_1) + Syn(S_2 : X_2; Y_2) \\ Unq((S_1, S_2) : (X_1, X_2) \setminus (Y_1, Y_2)) &= Unq(S_1 : X_1 \setminus Y_1) + Unq(S_2 : X_2 \setminus Y_2) \\ Unq((S_1, S_2) : (Y_1, Y_2) \setminus (X_1, X_2)) &= Unq(S_1 : Y_1 \setminus X_1) + Unq(S_2 : Y_2 \setminus X_2) \end{aligned} \tag{S4}$$

  This property is important since it guarantees that PID components of two independent subsystems can be computed independently and then summed together to obtain the PID components for the full system. Discrete BROJA satisfies additivity, while I_{min} and I_{MMI} do not[S5].

An additional property satisfied by all three discrete measures is the *pairwise marginals* property[S6], i.e. $Red(S : R_1, R_2)$, $Unq(S : R_1 \setminus R_2)$ and $Unq(S : R_2 \setminus R_1)$ only depend on pairwise marginal distributions between the sources and the target $p(s, r_1)$ and $p(s, r_2)$. This implies that all dependencies between the target S and the sources (R_1, R_2) that go beyond the dependency between the individual sources and the target are quantified as synergy. Even though the pairwise marginals is a fundamental property of the three PID measures, it has been questioned whether PID measures should always satisfy it[S6,8]. For I_{MMI} and I_{min}, which have been defined for more than two source variables, the non-negativity, symmetry and pairwise marginal properties hold for any number of sources.

Venkatesh and colleagues empirically showed, on simulated jointly-Gaussian data, that gPID appears to satisfy the same properties proved for the discrete BROJA decomposition measure[S1]. A formal proof of these properties for gPID is still lacking but would follow immediately by establishing the conjecture that, for every jointly Gaussian distribution $p(s, r_1, r_2)$ the distribution q^* that minimizes the BROJA objective in Eq. (19) is itself Gaussian[S1].

Supplementary Note 3: Illustration of the origin of the bias

Before detailing the procedures for correcting for the limited-sampling bias, we illustrate intuitively the origin of the limited-sampling bias in information calculations.

We first considered the bias for the discrete information calculation. We simulated two neural units (sources for the PID analysis, two spiking neurons in this example) that are completely uninformative about the stimulus (target for the PID analysis) (see Fig. 1A). In this simulation, both the joint and single-neuron information should be zero, but the values we obtain will be different from zero because of limited-sampling of the probabilities. Specifically, we simulated two neurons R_1 and R_2, each responding on each trial with a uniform marginal distribution of spike counts ranging from 1 to 4, regardless of which of $S = 2$ stimuli was presented. The neurons are also uncorrelated, so their joint distribution is uniform across all possible 16 joint responses, and equal between the two stimuli. Examples of marginal and joint stimulus-conditional response probability histograms sampled from a finite number of trials ($N = 100$ trials, that is 50 per stimulus in the top

row and $N = 1000$ trials (that is 500 per stimulus) in the bottom row respectively) are shown in Fig. 1A (responses to stimuli 1 and 2, respectively). Despite all the true response distributions being uniform across response bins and stimuli, a single instantiation of the probabilities from simulated data will have differences across stimuli that are not real but just due to random fluctuations. With smaller numbers of trials (Fig. 1A, top), we can appreciate that the random fluctuations that generate spurious information are larger for the joint probability (the few trials are spread over a larger set of possible responses $R = 16$) than for the marginal probabilities (the few trials are spread over a smaller set of responses $R = 4$). As a result of these spurious differences between stimulus-specific response distributions, the values of information computed from individual simulations with the limited number of trials (Fig. 1B, top) would not be distributed around the true value of zero bits, but around a spurious non-zero value (the bias). Because the redundancy is computed from marginal probabilities and the joint information and the synergy rely instead on the joint probabilities, the bias is smaller for redundancy than for synergy. With larger number of trials (Fig. 1A, bottom), the random fluctuations in the empirical probabilities get smaller (the single instantiation looks more uniform across stimuli, as it should be by ground-truth), and thus the bias is reduced (Fig. 1B, bottom).

We next considered the limited-sampling bias obtained when computing information with the Gaussian method (which assumes that the joint distribution of stimulus and target is a multivariate Gaussian). In this case, the joint information between target and the two sources is just a function of the covariance matrix $\Sigma(S, R_1, R_2)$, see Eq. (21). We can thus understand the limited-sampling bias of the Gaussian information as arising from limited-sampling fluctuations of the covariance matrix. We simulated two source Gaussian signals and one for the target, all of them with $d = 5$ dimensions (see Fig. 1C,D). These simulated data are independently distributed, both across different variables and across different dimensions within the same variable, with zero mean and unit variance. The true covariance matrix should be diagonal with no off-diagonal covariance and thus the information between the target and the single source and the joint information between the target and the two sources should be zero. Examples of empirical full covariance matrices of the stimulus and the neural response sampled from a finite number of trials (20 and 50 trials, respectively in Fig. 1C) show that, however, individual realizations of the covariance matrix sampled from the data show random off-diagonal structure which are treated by the information equation as relationship between the variables and thus give non-zero information values. Similarly to random fluctuations of joint probability values for the discrete case, the off-diagonal entries are larger in absolute value for smaller trial numbers (and would be smaller for marginal covariances, not shown, as they average random fluctuations along the values of the marginalized variable). Thus, each information calculation with a fixed number of trials N would give a positive information value, and thus the average of the information calculation over many realizations with N trials would be larger than zero (rather than equal to zero as the ground truth value should be). Hence the information calculation suffers from an upward limited-sampling bias, which becomes larger for smaller numbers of trials N. Again, because the redundancy and single neuron information depends only on the marginal covariances and the joint information depend on the full joint covariance, the bias of the redundancy is smaller than that of synergy and joint information (Fig. 1D).

Supplementary Note 4: Further results of simulations and bias corrections

Here we present and collect all additional analyses of simulated data reporting the bias properties of the PID including results of the PID bias correction methods on simulated data. In Fig. S3 we show further details of how the bias of the discrete PID varies with R.

In Figs. S4 and S8 we show further details of how the bias of the discrete PID varies for

different information levels.

In Fig. S5 we show further results for simulated scenarios with some discrete PID components having a null ground-truth value.

In Figs. S6, S7 we show how the bias of two different discrete PIDs (I_{min} and I_{MMI}) scales with the number of trials and we confirm the effectiveness of our bias correction procedures with these PIDs.

In Figs. S9 and S10 we show details of how the Gaussian bias varies with d and N.

In Fig. S11 we show further results for simulated scenarios with some Gaussian PID components having a null ground-truth value.

Supplementary Note 5: Application of Gaussian PID to simulated spike count data

In this section, we test with both real data and simulations whether a Gaussian PID can be used for the analysis of spike counts in response to discrete uniformly distributed stimuli. We conclude that using the Gaussian PID on such data is not recommended, as it can give a large underestimation of the information values as well as non-credible synergy and redundancy attribution. In contrast, the discrete PID gives more robust estimates of the information values and credible attribution of synergy and redundancy. We thus recommend the use of the discrete PID for these types of data.

Gaussian PIDs[S1,3] are convenient because they allow analyses of high multivariate responses with relatively low trial numbers. However, they rely on the assumption that sources and targets are distributed according to multivariate Gaussian probabilities. Here we investigated whether these are generally applicable to typical experiments recording the spiking activity of neurons in response to discrete classes of stimuli with uniform distribution of the experimentally presented stimuli, which corresponds to a major class of experimental data. We will investigate this first by simulations under these conditions and then by analyzing real experiments with spiking activity.

It is well documented that the number of spikes emitted by a neuron in response to different stimuli follows approximately a Poisson distribution[S9]. Moreover, the time windows in which neurons process and transmit information are usually short, often of only few tens of milliseconds[S10]. Given that the firing rate of cortical neurons ranges from one or a few spikes per second (when considering the less effective stimuli) to several tens of spikes per second (when considering the most effective stimuli), firing rate distributions relevant for neural information processing can be conceptualized as near-Poisson mean spike counts not exceeding a few spikes. We will thus examine applications of Gaussian PID to simulated neural firing under those conditions.

To explain the effect of using Gaussian approximations on neural data, it is easier to express mutual information as the difference between response entropy $H(R_1, R_2)$ (function of $p(r_1, r_2)$) and noise entropy $H(R_1, R_2|S)$ (function of $p(r_1, r_2|s)$):

$$I(S; R_1, R_2) \quad = -H(R_1, R_2|S) + H(R_1, R_2) \tag{S5}$$

Errors in estimating information with the Gaussian PID will arise from errors in approximating the response probability with Gaussians. Because the Gaussian distribution is the one with the highest entropy across all distributions with a given variance[S11], replacing a true underlying non-Gaussian distribution with a Gaussian with the same mean and variance will overestimate the corresponding entropy. When the Poisson process has a relatively low mean spike count, the probabilities of neural responses conditional to the stimuli $p(r_1, r_2|s)$ are not well approximated by Gaussian distributions (Fig. S13A). This may result in an overestimation of $H(R_1, R_2|S)$ when using a Gaussian. When pooling together responses to individual stimuli using a uniform probability of stimulus presentation to form a marginal probability of responses independent of the stimuli $p(r_1, r_2)$, this probability will differ from a Gaussian, especially for larger values of the

number of uniformly distributed stimuli S (Fig. S13B). This will lead to a large overestimation of $H(R_1, R_2)$. All these considerations are illustrated in Fig. S13A,B with a cartoon of the stimulus-conditional and stimulus unconditional probability distributions of an individual neuron, and similar considerations apply to joint distributions.

To test quantitatively the errors introduced by approximating the discrete distributions with Gaussians on the mutual information estimation (which combines the effects of estimation errors on response and noise entropy), we ran a parameter sweep of a simulation with two uncorrelated Poisson neurons, as described in the 'uncorrelated' scenario of the discrete simulations (see Methods and Table S2). Stimuli were presented with a uniform distribution, as it is often the case with experimental design when using discrete classes of stimuli. We varied the number of stimuli S from 2 to 12 to investigate the effect of this experimental design parameter.

We computed the ground-truth information values from exact Poisson probabilities of spike counts that we used to generate the data. We then compared the information values obtained with the two approaches that can be applied to real data in which the probabilities are unknown. The first is the discrete approach, the one at the basis of the discrete PID. We discretized the responses into $R = 4$ equipopulated bins (as it could be done reliably also in experiments with more limited numbers of trials). In the second approach, we used the single-neuron mutual information with the Gaussian formula (Eq. (20)), which fits a multidimensional Gaussian to the joint distribution of target and sources.

In Fig. S13C-E we plot the values of the ground-truth information as well as those obtained with the discrete and the Gaussian information approach. We simulated neural responses across different values of simulation parameters α regulating the difference in firing rates across stimuli (the higher α, the larger the information) and B regulating the background stimulus unrelated firing (the larger B, the smaller the information). We varied α and B independently from 1 to 10, both in steps of 1. We computed information using a large number (10000) of simulated trials per stimulus to avoid bias issues. For $S = 2$ equiprobable stimuli the Gaussian procedure gave results reasonably close to the ground truth value, but for $S = 4$ and $S = 12$ it severely underestimated the ground truth information. The Gaussian information values were only $\approx 50\%$ and $\approx 20\%$ of the ground-truth values for $S = 4$ and $S = 12$ stimuli, respectively. In contrast, the discrete PID computed with $R = 4$ response bins provided a good approximation to the ground truth information across a wide range of parameters and values of S (the discretized information values were $\approx 90 - 100\%$ of the ground-truth value, with the small information loss due to the data processing inequality as some different spike counts are grouped together when discretizing into $R = 4$ bins).

In addition to checking whether the joint information values are accurate, we also studied whether the results of the PID decomposition were credible. In our simulations, with each neuron uncorrelated with the other and each neuron encoding a different and independent binary stimulus, synergy and redundancy must be zero. We verified that the discretized PID BROJA indeed gave zero synergy and redundancy in these cases (see Fig. S5 case 'Uncorr'). However, when we applied the Gaussian PID to these uncorrelated Poisson simulated data we found high synergy despite the absence of correlations (results are plotted for the $S = 4$ case in Fig. S13F, but we verified that similar results held for $S = 2$ and $S = 12$, not shown). Additionally we found that the unique information of neuron 1 was considerably larger than that of neuron 2, although both neurons were simulated with identical information levels and properties. These not-credible PID decomposition values were not due to numerical errors due to limited sampling as the results were stable across trials, and are thus a consequence of fitting a probability model (the Multivariate Gaussian) to data that are not well described by this parametric distribution. Moreover, we verified that whenever we used uncorrelated processes generated with a true multivariate Gaussian distribution we had zero redundancy and synergy with the gPID (see also[S1] and Fig. S11 case 'both unique').

Finally, to test the Gaussian PID on real spiking data, we tested it on the three spiking datasets. We computed the joint information between one target (stimulus) and two sources (activity of two neurons). As predicted by simulations, we find an excellent approximation to the discrete values for dataset 1 in which $S = 2$, but very poor performance and major underestimations for the datasets with larger S. For dataset 2 (which had $S = 12$), we had a severe underestimation (the Gaussian information was 20% of the discrete one) and for dataset 3 (which had $S = 399$) we had an even poorer underestimation of the discrete information (the Gaussian information was 5% of the discrete one).

Supplementary Note 6: Analytical considerations on the bias of discrete and Gaussian PIDs

Here we describe analytical calculations that account for the bias properties of Shannon information and for the fact that for both discrete and Gaussian PID the synergy suffers from an upward bias larger than that of the redundancy.

Analytical considerations for the discrete PID using an asymptotic expansion

We consider first the discrete PID.

Suppose that we compute the entropy in a certain discrete space X (in the applications of this paper, this space will either be the joint space of pairwise sources and target, or the marginal space of a subset of these quantities) having at our disposal only a finite number N of trials. The entropy of this probability distribution is defined (in bits) as:

$$H(X) = -\sum_{x} p(x) \log_2(p(x)) \tag{S6}$$

The true values of the probabilities are not known a priori in experimental situations. The probabilities could be perfectly computed from the data if we had infinite amounts of experimental trials, but here we consider the setting in which we have only a finite number of trials N. If we have an empirical estimate of the probabilities as the empirical occurrences $p_N(x)$ in the N available trials, we compute the value of the entropy H_N obtained with a finite number of trials N:

$$H_N(X) = -\sum_{x} p_N(x) \log_2(p_N(x)) \tag{S7}$$

From this, we can compute the finite sampling bias of the entropy, defined as the difference between the average value $\langle H_N \rangle$ of H_N over all instantiations with N trials and the true value H obtained from the ground-truth distributions.

Analytical approximations to the bias have been derived[S12–14] in the large N limit, defined formally as the case when $N{\cdot}p(x) \gg 1$. In our applications, this would translate to $N{\cdot}p(r_1, r_2, s) \gg 1$ for each target value s and joint source value r_1, r_2. If this is satisfied for the joint distribution of the three variables then it is also satisfied for the marginal distributions, e.g. $N \cdot p(r_1, r_2) \gg 1$ for each joint response r_1, r_2, and $N \cdot p(r_i, s) \gg 1$ (i = 1,2). While it is unrealistic that this large N limit case will be formally satisfied in practical experiments, this type of derivation has proven very useful to provide approximate expressions of the bias of Shannon information (which are quantitatively precise if N is large enough but are still qualitatively useful for intermediate values of N), to understand its properties and to design strategies to correct for it[S12,13,15]. This previous work has reported an expansion of the bias of the entropy in powers of $1/N$

$$\langle H_N \rangle - H = \frac{C_1}{N} + \frac{C_2}{N^2} + \cdots \tag{S8}$$

with the leading term $1/N$ in the bias being

$$\frac{C_1}{N} = -\frac{(R_x - 1)}{2N\ln(2)} \tag{S9}$$

where R_x denotes the number of relevant events (called responses here with applications to neural activity in mind) for the considered distribution, defined as the number of responses x with probability $p(x) > 0$[S13,16]. Thus, although the bias is not the same for all probability distributions, in the asymptotic sampling regime, its leading term depends on a remarkably simple detail of the response distribution: the number of trials and the number of relevant responses. This makes it possible to derive simple rules of thumb for estimating the bias magnitude and compare the relative bias in different situations. As we will discuss now, this equation can predict effectively how the bias differs across PID terms depending on the number of discrete bins of sources and targets and on the overall information level.

The leading bias terms of the joint information can be computed by writing information as sums and differences between three entropies (Eq. (11)). Using the above leading term for the bias of each entropy term in (Eq. (11)) we obtain the following expression for the bias of the joint information:

$$\langle I_N(S;R_1,R_2)\rangle - I(S;R_1,R_2) = \frac{1}{2N\ln(2)}\left[(\tilde{R}_{s,r_1,r_2} - 1) - (\tilde{R}_{r_1,r_2} - 1) - (S-1)\right] \tag{S10}$$

where in the above $\langle I_N(S;R_1,R_2)\rangle$ denotes the average value of the joint information estimated with a finite number of trials N, taken over all instantiations with N trials. $\tilde{R}_{s,r_1,r_2}$ is the number of bins with non-zero joint probability $p(s,r_1,r_2)$, $\tilde{R}_{r_1,r_2}$ is the number of bins with non-zero marginal probability $p(r_1,r_2)$ and S is the number of values of the target variable (as in most experimental designs, the target variable is the experimentally-presented stimulus, for obvious reasons we assume with this notation that each presented stimulus has a non-zero probability of being presented). Because the absolute value of the entropy bias increases with the number of possible responses, the biggest contribution to the bias of the joint information $I(S;R_1,R_2)$ (Eq. (11)) comes from the bias of the joint entropy of target and two sources $H(S,R_1,R_2)$, whose bias originates from finite sampling fluctuations of the joint probability $p(s,r_1,r_2)$ and thus has a proportionality coefficient of $\tilde{R}_{s,r_1,r_2}$. If all or most bins have non-zero probability, then $\tilde{R}_{s,r_1,r_2} \approx S \times R \times R$.

The leading term of the bias of the single cell information (Eq. (10)) comes instead from $H(S,R_i)$, which depends on the marginal distribution $p(r_i,s)$ of target and one source. The finite sampling fluctuations of the marginal probabilities (Fig. 1A) are smaller than the ones of the joint probabilities and thus its bias has a proportionality coefficient of $\tilde{R}_{s,r_i}$ which scales as $\approx S \times R$ if all or most bins have non-zero probability. Thus, the bias of the single cells is approximately R times smaller than that of the joint information and its leading term in $1/N$ is as follows:

$$\langle I_N(S;R_i)\rangle - I(S;R_i) = \frac{1}{2N\ln(2)}\left[(\tilde{R}_{s,r_i} - 1) - (\tilde{R}_{r_i} - 1) - (S-1)\right] \tag{S11}$$

where $\langle\cdot\rangle$ denotes the average over all instantiations of $I_N(S;R_i)$ computed with N trials.

Importantly, the analytical expression can also explain why the bias of the zero-information shuffled data (and in general of the bias with lower information levels) is larger than the bias of the original distribution and give an idea of the situations in which they are expected to be tight[S13]. Take for example the joint information, for which the bias is mostly dictated by the number of relevant response bins $\tilde{R}_{s,r_1,r_2}$ of the joint distribution. Informative cases will have stimulus-specific distributions that are restricted to a smaller number of relevant bins than the total number of bins (because informative cases have less variability at fixed stimulus so more restricted stimulus-specific distributions) and also the bins that are relevant for a specific stimulus

would not all be relevant for other stimulus-specific distributions (because informative cases will also show diversity of response distributions to different stimuli). When we randomly shuffle the trials combining trials from different stimuli, the shuffling operation will mix up responses and thus create larger stimulus-specific distributions (larger numbers of stimulus-specific relevant bins) which will then increase the bias. This prediction is confirmed by all our simulations, which show larger finite-sampling bias for lower information levels in discrete Shannon information estimates (Figs. 1B, S4, S5). This result is important because it implies that bias corrections based on subtracting shuffled estimators lead to a residual downward bias, which is useful to produce lower-bounded or conservative estimates. It also implies that the shuffled-subtracted bias corrected estimates will be precise and not too conservative when the information level in the original data is low (because in this case the number of relevant bins of the shuffled and original data will be similar) and it will be too conservative when the information levels in the data are high (because in this case the number of relevant bins of the shuffled distribution will be much larger than the corresponding one for the original distribution). This leads to the design of tighter downward biased estimators of information that may be helpful to draw conservative yet sufficiently accurate conclusions.

Having understood the bias of Shannon information measures relevant for PID, we now use this knowledge to evaluate the bias of the PID terms. We focused first on the bias of the synergy as defined in I_{MMI}. We remind that the synergy is defined as a difference between the joint and the Union information (Eq.7), with the I_{MMI} Union information defined as follows:

$$Union = \max(I(S;R_1), I(S;R_2)) \tag{S12}$$

Thus, the bias of the Union information equals the bias (Eq. (S11)) of the single source information $(I(S;R_i))$ with the highest plugin information value of the two single-source information. The bias of the synergy is the difference between the bias of the joint information, which scales as $(S \times R \times R)/N$, and the one of the single-source information which scales as $(S \times R)/N$ (thus R times smaller than the bias of the joint information). Thus the bias of the synergy will be positive and scaling as $(S \times R \times R)/N$. This prediction is confirmed in all numerical simulations with I_{MMI}(Fig. S7).

We then repeated this approach but focusing on the bias of the synergy as defined in BROJA. The BROJA Union information is defined as the result of an optimization problem, minimizing the joint information $I_q(S;R_1,R_2)$ over the set Δ_P of all joint probability distributions $q(s,r_1,r_2)$ that have the same pairwise marginals $q(s,r_1) = p(s,r_1)$ and $q(s,r_2) = p(s,r_2)$ as the original distribution $p(s,r_1,r_2)$ (Eq. (6)). When applied to limited data N, the minimization within the Δ_P space will tend to select a minimum information within the space set by the marginals $p_N(r_1,s)$ and $p_N(r_2,s)$ computed with N trials. It will thus select probability distributions with a low finite sampling information value. Because this bias is by and large positive, we expect that the minimization procedure will select probabilities with lower bias. We therefore conjecture that the distribution q^* minimizing the mutual information over the Δ_P space has a bias similar to the one obtained from the probability distribution $p_{ind}(r_1,r_2,s)$ defined directly from the marginals such that the neural units would have the same single unit probabilities but no interactions at fixed stimulus (stimulus-conditional independence, or lack of noise correlation as it is termed in the neural literature[S17,18])

$$p_{ind}(r_1,r_2,s) = p(r_1|s)p(r_2|s)p(s) \tag{S13}$$

We call the I_q computed on $q = p_{ind}$ the conditionally-independent information $I_{ind}(S;R_1,R_2)$, defined as the joint mutual information with stimulus-conditional probabilities $p_{ind}(r_1,r_2|s)$ and stimulus unconditional probabilities $p_{ind}(r_1,r_2) = \sum_s p_{ind}(r_1,r_2,s)$. For clarity, the conditionally-independent information is the information carried by the population about the target if the single neural unit responses were the same as the original data but there were no noise correlations

(no target-conditional interactions between the neural units), and it has been used as a reference distribution for understanding whether noise correlations increase or decrease information (see[S19,20]). $I_{ind}(S; R_1, R_2)$ can be expressed in terms of entropies as:

$$I_{ind}(S; R_1, R_2) = -H(S, R_1) - H(S, R_2) + H_{ind}(R_1, R_2) + 2H(S) \quad \text{(S14)}$$

Importantly for our PID bias understanding, in I_{ind}, compared to the joint information, we replace the entropy of the two sources and the target $H(S, R_1, R_2)$, whose bias scales as $(R^2 \times S)/N$, with the term $H(S, R_1) + H(S, R_2)$, whose bias scales as $(R \times S)/N$ which is R times smaller. Thus, the bias of the joint information is larger than and grows faster with R than the bias of $I_{ind}(S; R_1, R_2)$. Because the minimal set of finite sampling variations in the Δ_P space is set by the finite sampling fluctuations of the marginals, we assume that the minimization in the Union calculation will select distributions roughly with the same bias as $I_{ind}(S; R_1, R_2)$. To test this, we performed simulations of pairs of neural units. We found (see Fig. 3) across all conditions and trial numbers that the bias of the Union information is very similar to the bias of $I_{ind}(S; R_1, R_2)$. It is actually slightly lower, which is compatible with the understanding that the minimum selects a similar bias and of course smaller statistical fluctuations. The synergy is the difference between the joint and the Union information. Similarly to the cases of the MMI described above, the bias of the joint information is the largest of the two and scales as $(S \times R \times R)/N$, whereas the bias of the Union information is smaller and scales as $(S \times R)/N$. Thus, again, the bias of the synergy will be positive and scale as $(S \times R \times R)/N$ when R increases or N decreases. Because the biggest bias term of the synergy comes from the joint information, which decreases with the information level, the bias of the synergy should decrease with the information level. The above predictions are confirmed in all numerical simulations (Figs. 1B, 2A, S3A-C, S4, S5).

The redundancy is the difference between the sum of single source information and the Union information. The bias of the redundancy is thus a difference of two terms that scale as $(S \times R)/N$. These terms may partly cancel out and none of the two scales with R as fast as the bias of the joint information, which is R times larger. Therefore redundancy is expected to be less biased than synergy, with the gap between the two biases expected to increase steeply as R increases. Once again, these predictions are confirmed in all numerical simulations with both BROJA and I_{MMI} (Figs. 1B, 2A, S3A-C, S4, S5, S7).

While we derived these bias properties for the BROJA and I_{MMI} PID, we expect that the same conclusions would hold for I_{min} and we verified with simulations that these predictions indeed hold with I_{min} (Fig. S6). In fact also for I_{min} the Union information depends on the two marginal probabilities $p(r_1, s)$ and $p(r_2, s)$ because they satisfy the so-called pairwise marginals property. We expect that all the considerations we made about the relative size of the bias of synergy and redundancy hold for any definition of PID satisfying the pairwise marginals property, because in this case our key reasoning (that Union information has bias determined by the finite size fluctuations of the marginals) would hold true.

In sum, the above explains why synergy has a large positive bias, larger than the one of redundancy, and it explains why the bias of synergy gets proportionally bigger for large numbers of possible discretized single cell responses.

Analytical considerations for the Gaussian PID

We consider a multivariate Gaussian variable X with dimensionality d_X and covariance matrix $\Sigma(X)$. The Shannon entropy of this multivariate Gaussian can be expressed as a function of the covariance matrix and of the dimensionality, as follows:

$$H(X) = \frac{1}{2\ln(2)} \ln(\det(2\pi e \Sigma(X))) = \frac{1}{2\ln(2)} (d_X \cdot (1 + \ln(2\pi)) + \ln(\det(\Sigma(X)))) \quad \text{(S15)}$$

As for the discrete case, we consider the setting in which the Gaussian PID and thus the covariance matrix $\Sigma(X)$ are to be estimated by N available trials. The sampled covariance matrix $\Sigma_N(X)$ estimated from the data will have statistical fluctuations from one random realization with N trials to another (Fig. 1C). It will be on average over all instantiations with N trials, equal to the true one. Since the log-determinant is a nonlinear concave function, the average across realizations of the log-determinant of the sampled covariance matrix will be lower or equal to the log-determinant of the average sampled covariance matrix. As a result, the entropy will have a downward bias. This bias has been computed analytically by Goodman[S21] and it takes the form:

$$\text{Bias}\big[H(X)\big] = \frac{1}{2\ln(2)}\left(\langle \ln\det\big(\Sigma_N\big)\rangle - \ln\det\big(\Sigma\big)\right) = \frac{1}{2\ln(2)}\left(d_X \, \ln\Big(\frac{2}{N-1}\Big) + \sum_{k=1}^{d_X}\psi\Big(\frac{N-k}{2}\Big)\right) \tag{S16}$$

where $\psi(x)$ is the digamma function and $\langle \ln\det\big(\Sigma_N\big)\rangle$ indicates the average value of the log-determinant of the sampled covariance matrix taken over all random instantiations with N trials. Another study[S22] provided a more compact, closed-form approximation for this bias in terms of logarithms, namely:

$$\text{Bias}\big[H(X)\big] = \frac{1}{2\ln(2)}\sum_{k=1}^{d_X}\ln\Big(1-\frac{k}{N}\Big) \tag{S17}$$

which is obtained by approximating the digamma function as a logarithm. These two analytical expressions of the Gaussian entropy show that the bias depends only on the sample size N and the dimensionality d_X of the Gaussian variable, not on the structure of the covariance matrix $\Sigma(X)$. In our numerical bias corrections we used the analytical expression of Eq. (S16) bias equation, because it is an exact formula that works slightly better than the approximate one provided by Cai and colleagues (see Fig. S14).

Importantly, the entropy bias is a negative function which decreases in absolute value when increasing N and increases in absolute value when increasing d_X. This is known from the properties of the digamma functions and can be easily appreciated by plots of their values (Fig. S14). Note that the absolute value of the bias increases steeply and supralinearly with d_X at fixed N. Thus the bias of the entropy of a variable with e.g. $2d_X$ dimensions would be more than twice larger in absolute value than the bias of a variable with d_X dimensions (Fig. S14). For example the bias of $H(S, R_1, R_2)$ would be more than three times larger than that of $H(R_1)$, which has three times fewer dimensions.

To understand the bias of the Gaussian information quantities and of the PID components, for simplicity, we assume that both Gaussian sources and the Gaussian target have all the same dimensionality d. We now recall that the joint information between the two sources R_1 and R_2 about the target S can be written as a function of three entropy terms (Eq. (11)). Using Eq. (S16) this leads to an expression of the bias of $I(S; R_1, R_2)$ only in terms of d and N:

$$\text{bias}[I(S;R_1,R_2)] = \frac{1}{2\ln(2)}\left(-\sum_{k=1}^{3d}\psi\left(\frac{N-k}{2}\right) + \sum_{k=1}^{2d}\psi\left(\frac{N-k}{2}\right) + \sum_{k=1}^{d}\psi\left(\frac{N-k}{2}\right)\right) \tag{S18}$$

Plots of the bias of $I(S; R_1, R_2)$ show that its bias is positive because, as for the discrete case, the largest contribution to the bias among the three entropies (Eq. (11)) comes from $-H(S, R_1, R_2)$ whose bias originates from finite sampling fluctuations of the joint probability $p(s, r_1, r_2)$ with covariance matrix $\Sigma(S, R_1, R_2)$ (Eq. (9)). The bias of $I(S; R_1, R_2)$ increases steeply with d and decreases with N (Fig. 2B).

Instead, the single cell information (Eq. (10)) has the biggest bias term coming from $H(S, R_i)$, which depends on the marginal distribution $p(r_i, s)$ of target and one source and thus from the

covariance matrix $\Sigma(S, R_i)$. Using Eq. (S16), the single cell information has the following bias:

$$\text{bias}[I(S;R_i)] = \frac{1}{2\ln(2)}\left(-\sum_{k=1}^{2d}\psi\left(\frac{N-k}{2}\right) + 2\sum_{k=1}^{d}\psi\left(\frac{N-k}{2}\right)\right) \tag{S19}$$

which has the largest contribution from a term with sums of digamma functions up to $2d$ and thus is smaller than the bias of $I(S;R_1,R_2)$ which has the highest contribution from sums of digamma functions up to $3d$. The intuitive reason why the bias is smaller is that finite sampling fluctuations of the marginal probabilities and of the marginal covariance matrices are smaller than the ones of the joint probabilities.

We then considered the bias of the Gaussian PID terms. We first focused on the synergy bias (which is the difference between the joint and the Union information bias) defined in I_{MMI}. The bias of the joint information was discussed above and is dominated by the sum of digamma functions up to $3d$. Because the Union information of I_{MMI} is defined as the maximum between the two single source information (Eq. (S12)), its bias equals the bias of the single source information $(I(S;R_i))$ with the highest information value. In the Gaussian case, both have the same bias, and thus the bias of the Union MMI information equals the one of single source information defined in Eq. (S19). Because the former has a larger positive bias dominated by the sum of digamma functions with terms up to $3d$ and the latter has a smaller positive bias whose larger term is a sum of digamma functions with terms up to $2d$, the synergy has a positive bias that increases steeply with d and decreases with N.

We then repeat this approach but focusing on the bias of the synergy with gPID[S1] (the Gaussian equivalent of BROJA). As for the discrete BROJA case, for the same reasoning we can approximate the bias of the gPID Union information with the one of the conditionally-independent joint information, $I_{ind}(S;R_1,R_2)$, built from $p_{ind}(s,r_1,r_2)$. The fact that the gPID Union information bias can be well approximated by the I_{ind} bias is numerically verified with high accuracy in the simulations (Fig. 3C). Because of the constraints used to construct the independent covariance matrix $\Sigma_{ind}(S,R_1,R_2)$ (see Methods), this covariance matrix has fewer parameters than the full covariance matrix used for the joint information, which suggests that the information $I_{ind}(S;R_1,R_2)$ is easier to sample with finite data. The information $I_{ind}(S;R_1,R_2)$ is obtained by replacing in Eq. (21) the full covariance matrix with the independent one $\Sigma_{ind}(S,R_1,R_2)$. For the sake of understanding its bias, it is useful to decompose $I_{ind}(S;R_1,R_2)$ into a sum of entropies as follows:

$$\begin{aligned} I_{ind} &= -H_{ind}(S,R_1,R_2) + H_{ind}(R_1,R_2) + H(S) = \\ &= -H(S,R_1) - H(S,R_2) + H_{ind}(R_1,R_2) + 2H(S) \end{aligned} \tag{S20}$$

The largest term of the bias of $I_{ind}(S;R_1,R_2)$ is due to $H_{ind}(S,R_1,R_2) = H(S,R_1) + H(S,R_2) - H(S)$ which has a smaller bias than the corresponding entropy $H(S,R_1,R_2)$ entering the synergy and joint information, because the bias of $H_{ind}(S,R_1,R_2)$ has contributions from terms up to dimensionality $2d$ whereas the bias of $H(S,R_1,R_2)$ is larger because it has contributions up to terms of dimensionality $3d$:

$$\text{bias}[H(S,R_1,R_2)] = \frac{1}{2\ln(2)}\left(3d\ln\left(\frac{2}{N-1}\right) + \sum_{k=1}^{3d}\psi\left(\frac{N-k}{2}\right)\right) \tag{S21}$$

$$\text{bias}[H_{\text{ind}}(S,R_1,R_2)] = \frac{1}{2\ln(2)}\left(3d\ln\left(\frac{2}{N-1}\right) + 2\sum_{k=1}^{2d}\psi\left(\frac{N-k}{2}\right) - \sum_{k=1}^{d}\psi\left(\frac{N-k}{2}\right)\right) \tag{S22}$$

Because the bias of the synergy is a difference of the larger bias of the joint information and the smaller of the Union information, and because the difference between the two increases

monotonically with d, the bias of the synergy is positive and grows steeply with d. Again, this is numerically well verified in all simulated cases (Figs. 1C,D, 2B, S3D-F, S9, S10, S11).

The redundancy is the difference between the sum of single neuron information and the Union information. For both MMI and gPID, its bias is a difference of terms that are as biased as the single neuron information, which is expressed by a sum of digamma functions with up to $2d$ terms (rather than $3d$ terms as for the dominant term of the synergy bias). Redundancy is thus less biased than the synergy for large d or small N.

Supplementary Note 7: Study of how to scale up calculations and bias corrections of discrete PID using dimensionality reduction

In this section we report a preliminary investigation of how discrete PID calculations may be scaled up to become feasible and unbiased even with higher dimensionality of the sources (for neurons, for example with large neuronal populations).

We simulate two independent populations of 10 uncorrelated Poisson neurons, responding to four different stimuli with uniform distribution (Eq. (26) with parameters $\alpha = 1$, $B = 10$, $\beta = 0$, $\gamma = 0$). These stimuli represent two binary features, with each pool selectively encoding one of the two features. The two neural pools serve as independent 10-dimensional sources in the Partial Information Decomposition (PID) analysis. Because the two populations are uncorrelated and each population represents only one of the two independent binary stimulus features s_1 and s_2 (see Methods), the PID of the information about the stimulus (the target) and the activity of the two sources (adding up to a total population of 20 neurons) must have zero synergy or redundancy across neurons.

The discrete PID with two 10-dimensional sources cannot be performed directly with reasonably sized datasets. Assuming we discretize each neuron in 4 bins each, we would have that the number of possible discrete values of each source would be $R = 4^{10}$, which would require $N > 4^{10} \times 4^{10} \times 4 \times 4$ trials for the sufficient sampling regime, a number that far exceeds what is feasible experimentally. We thus reduced the dimensionality of each source to four possible discrete responses, by training a one-vs-one multiclass linear SVM (implemented using the MINT toolbox[S23], regularization parameter $C = 1$) to predict the value of the stimulus in a trial from the activity of each source made of a population of 10 neurons. (For this SVM analysis, we used a 2-fold cross-validation, ensuring that the trials were identically split across sources). Reducing the dimensionality of different populations of neurons with SVM to study the effect on information coding of the interactions between neurons is well established in neuroscience[S23–25], although to our knowledge the coupling of SVM to PID in the way described here is a novelty of our approach. These reduced neural representation data of each population are then fed as the value of the source in each trial to the PID routine. Encouragingly, we found (Fig. S18) that when applying our three discrete PID bias correction methods (QE, shuffle subtraction and merged) with an SVM dimensionality reduction, the PID estimates were well bias corrected. The estimation converged to the expected ground truth values of zero synergy and zero-redundancy, even for small trial numbers (results were very accurately corrected for the bias even down to $N = 256$ trials). Without bias corrections, far more trials would have been needed to conclude correctly that synergy and redundancy are zero.

While more work is needed to understand how to optimally scale up discrete PID to large populations, these results encourage us to think that the bias corrections methods developed here will be useful and crucial to devise scalable multidimensional versions of discrete PIDs.

Supplementary Note 8: Analytical derivation of Red_3 and Syn_3 bias

In this supplementary note we derive analytically the leading bias term of the three-way redundancy Red_3 and the three-way synergy Syn_3, for both the discrete and the Gaussian case.

Discrete PID

In this subsection we derive analytically the leading bias term of the three-way redundancy Red_3 and the three-way synergy Syn_3 in the discrete case.

As shown in Supplementary Note 6, in the asymptotic regime, the leading term of the bias of discrete Shannon information quantities scales as the number of parameters defining the highest-dimensional probability distribution entering the definition of the corresponding information quantity.

The bias of Red_3 scales as the bias of the minimum single-source Shannon information quantity (Eq. (30)). Thus, following the arguments of Supplementary Note 6, the bias of Red_3 scales as $(S \times R)/N$.

The bias of Syn_3 is the difference between the bias of the three-source joint information and the bias of the maximum two-source joint information (Eq. (31)). The three-source joint information can be expressed as a function of entropy terms as

$$I(S; R_1, R_2, R_3) = -H(S, R_1, R_2, R_3) + H(R_1, R_2, R_3) + H(S) \tag{S23}$$

Using the leading bias term for entropy as in Eq. (S9), we obtain the following expression for the three-source Shannon information bias:

$$\langle I_N(S; R_1, R_2, R_3)\rangle - I(S; R_1, R_2, R_3) = \frac{1}{2N\ln(2)}\left[\sum_s (\tilde{R}_{s,r_1,r_2,r_3} - 1) - (\tilde{R}_{r_1,r_2,r_3} - 1) - (S-1)\right] \tag{S24}$$

where $\langle I_N(S; R_1, R_2, R_3)\rangle$ denotes the average value of the three-source information estimated with a finite number of trials N, taken over all instantiations with N trials. $\tilde{R}_{s,r_1,r_2,r_3}$ is the number of bins with non-zero joint probability $p(s, r_1, r_2, r_3)$, and $\tilde{R}_{r_1,r_2,r_3}$ is the number of bins with non-zero marginal probability $p(r_1, r_2, r_3)$. If all or most bins have non-zero probability then $\tilde{R}_{s,r_1,r_2,r_3} \approx S \times R^3$, while $\tilde{R}_{r_1,r_2,r_3} \approx R^3$. Thus, in the asymptotic regime the leading term of the $I(S; R_1, R_2, R_3)$ bias scales as $(S \times R^3)/N$. Instead, as discussed in Supplementary Note 6 the leading term of the largest two-source Shannon information scales as $(S \times R^2)/N$. Thus, the bias of Syn_3 is dominated by the bias of the three-source joint information and scales as $(S \times R^3)/N$ in the asymptotic regime.

In summary, in the discrete case, the bias of Syn_3 scales much more quickly than the one of Red_3 (with the difference between them even greater than the one between the synergy and the redundancy of the two-source PID). This prediction is confirmed by all our simulations and experimental results (Fig. 7 and 8).

Gaussian PID

In this subsection we derive analytically the leading bias term of Red_3 and Syn_3 in the MMI Gaussian case (since gPID is only defined for two source variables[S1]).

As shown in Supplementary Note 6, the bias of Gaussian Shannon information quantities is determined by a sum of digamma-function terms whose upper limit is set by the dimensionality of the highest-dimensional entropy term (i.e. the largest covariance matrix) entering the definition of

the corresponding information quantity. Consequently, the upward bias of information estimates increases supralinearly with the dimensionality d of the sources and targets, decreases with the number of trials N, and grows more rapidly for information quantities involving higher-dimensional entropy terms.

The bias of Red_3 scales as the bias of the minimum single-source Shannon information (Eq. (30)). Thus, following the arguments of Supplementary Note 6, for the Gaussian PID the bias of Red_3 is dominated by the sum of digamma functions up to $2d$ dimensions (Eq. (S19)).

The bias of Syn_3 is the difference between the bias of the three-source joint information and the bias of the maximum two-source joint information (Eq. (31)). In Supplementary Note 6 (Eq. (S18)) we already showed that the bias of $I(S;R_i,R_j)$ is dominated by the sum of digamma functions up to $3d$ dimensions. Replacing the terms of Eq. (S23) using the Goodman formula (Eq. (S16)) we find the following analytical expression for the bias of $I(S;R_1,R_2,R_3)$:

$$\text{bias}[I(S;R_1,R_2,R_3)] = \frac{1}{2\ln(2)}\left(-\sum_{k=1}^{4d}\psi\left(\frac{N-k}{2}\right)+\sum_{k=1}^{3d}\psi\left(\frac{N-k}{2}\right)+\sum_{k=1}^{d}\psi\left(\frac{N-k}{2}\right)\right) \tag{S25}$$

Thus, the bias of $I(S;R_1,R_2,R_3)$ is dominated by the sum of digamma functions up to $4d$ dimensions, and it determines the leading term of the bias of Syn_3.

In summary, the Gaussian bias of the three-way synergy Syn_3 is dominated by terms of dimension $4d$ and is much larger than the bias of the three-way redundancy Red_3 (dominated by terms of dimension $2d$). Moreover, the gap between Syn_3 and Red_3 bias grows supralinearly both with increasing the number of dimensions d and with decreasing number of trials N. These predictions are confirmed by all our simulations and experimental results (Fig. 7 and 8).

References

S1. Venkatesh, P., Bennett, C., Gale, S., Ramirez, T., Heller, G., Durand, S., Olsen, S., and Mihalas, S. (2023). Gaussian partial information decomposition: Bias correction and application to high-dimensional data. Advances in Neural Information Processing Systems *36*, 74602–74635. URL: proceedings.neurips.cc/paper_files/paper/2023/file/ec0bff8bf4b11e36f874790046dfdb65-Paper-Conference.pdf.

S2. Williams, P. L., and Beer, R. D. (2010). Nonnegative decomposition of multivariate information. Preprint at arXiv. doi:doi.org/10.48550/arXiv.1004.2515.

S3. Barrett, A. B. (2015). Exploration of synergistic and redundant information sharing in static and dynamical Gaussian systems. Phys. Rev. E *91*, 052802. doi:10.1103/PhysRevE.91.052802.

S4. Harder, M., Salge, C., and Polani, D. (2013). Bivariate measure of redundant information. Phys. Rev. E *87*, 012130. doi:10.1103/PhysRevE.87.012130.

S5. Rauh, J., Banerjee, P. K., Olbrich, E., Montúfar, G., and Jost, J. (2023). Continuity and additivity properties of information decompositions. Int. J. Approx. Reason. *161*, 108979. doi:10.1016/j.ijar.2023.108979.

S6. Kolchinsky, A. (2022). A novel approach to the partial information decomposition. Entropy *24*. doi:10.3390/e24030403.

S7. Bertschinger, N., Rauh, J., Olbrich, E., Jost, J., and Ay, N. (2014). Quantifying unique information. Entropy *16*, 2161–2183. doi:10.3390/e16042161.

S8. Gomes, A. F. C., and Figueiredo, M. A. T. (2024). A measure of synergy based on union information. Entropy *26*, 271. doi:10.3390/e26030271.

S9. Tolhurst, D. J., Movshon, J. A., and Dean, A. F. (1983). The statistical reliability of signals in single neurons in cat and monkey visual cortex. Vision Res. *23*, 775–785. doi:10.1016/0042-6989(83)90200-6.

S10. Panzeri, S., Brunel, N., Logothetis, N. K., and Kayser, C. (2010). Sensory neural codes using multiplexed temporal scales. Trends Neurosci. *33*, 111–120. doi:10.1016/j.tins.2009.12.001.

S11. Cover, T. M., and Thomas, J. A. Elements of Information Theory. Wiley & Sons (2006). doi:10.1002/0471200611.

S12. Treves, A., and Panzeri, S. (1995). The Upward Bias in Measures of Information Derived from Limited Data Samples. Neural Comput. *7*, 399–407. doi:10.1162/neco.1995.7.2.399.

S13. Panzeri, S., and Treves, A. (1996). Analytical estimates of limited sampling biases in different information measures. Network *7*, 87–107. doi:10.1088/0954-898X/7/1/006.

S14. Paninski, L. (2003). Estimation of entropy and mutual information. Neural Comput. *15*, 1191–1253. doi:10.1162/089976603321780272.

S15. Strong, S. P., Koberle, R., De Ruyter Van Steveninck, R., and Bialek, W. (1998). Entropy and information in neural spike trains. Phys. Rev. Lett. *80*, 197. doi:10.1103/PhysRevLett.80.197.

S16. Panzeri, S., Senatore, R., Montemurro, M. A., and Petersen, R. S. (2007). Correcting for the sampling bias problem in spike train information measures. J. Neurophysiol. *98*, 1064–1072. doi:10.1152/jn.00559.2007.

S17. Averbeck, B. B., Latham, P. E., and Pouget, A. (2006). Neural correlations, population coding and computation. Nat. Rev. Neurosci. *7*, 358–366. doi:10.1038/nrn1888.

S18. Panzeri, S., Moroni, M., Safaai, H., and Harvey, C. D. (2022). The structures and functions of correlations in neural population codes. Nat. Rev. Neurosci. *23*, 551–567. doi:10.1038/s41583-022-00606-4.

S19. Schneidman, E., Bialek, W., and Berry, M. J. (2003). Synergy, redundancy, and independence in population codes. J. Neurosci. *23*, 11539–11553. doi:10.1523/JNEUROSCI.23-37-11539.2003.

S20. Pola, G., Thiele, A., Hoffmann, K., and Panzeri, S. (2003). An exact method to quantify the information transmitted by different mechanisms of correlational coding. Network *14*, 35–60. doi:10.1088/0954-898X/14/1/303.

S21. Goodman, N. (1963). The distribution of the determinant of a complex wishart distributed matrix. Ann. Math. Stat. *34*, 178–180. doi:10.1214/aoms/1177704251.

S22. Cai, T. T., Liang, T., and Zhou, H. H. (2015). Law of log determinant of sample covariance matrix and optimal estimation of differential entropy for high-dimensional Gaussian distributions. J. Multivar. Anal. *137*, 161–172. doi:10.1016/j.jmva.2015.02.003.

S23. Lorenz, G. M., Engel, N. M., Celotto, M., Koçillari, L., Curreli, S., Fellin, T., and Panzeri, S. (2025). MINT: a toolbox for the analysis of multivariate neural information coding and transmission. PLOS Comput. Biol. *21*, e1012934. doi:10.1371/journal.pcbi.1012934.

S24. Iurilli, G., and Datta, S. R. (2017). Population coding in an innately relevant olfactory area. Neuron *93*, 1180–1197. doi:10.1016/j.neuron.2017.02.010.

S25. Valente, M., Pica, G., Bondanelli, G., Moroni, M., Runyan, C. A., Morcos, A. S., Harvey, C. D., and Panzeri, S. (2021). Correlations enhance the behavioral readout of neural population activity in association cortex. Nat. Neurosci. *24*, 975–986. doi:10.1038/s41593-021-00845-1.
